# Donkey genomes provide new insights into domestication and selection for coat color

**DOI:** 10.1038/s41467-020-19813-7

**Published:** 2020-12-08

**Authors:** Changfa Wang, Haijing Li, Yu Guo, Jinming Huang, Yan Sun, Jiumeng Min, Jinpeng Wang, Xiaodong Fang, Zicheng Zhao, Shuai Wang, Yanlin Zhang, Qingfeng Liu, Qiang Jiang, Xiuge Wang, Yijun Guo, Chunhong Yang, Yinchao Wang, Fang Tian, Guilong Zhuang, Yanna Fan, Qican Gao, Yuhua Li, Zhihua Ju, Jianbin Li, Rongling Li, Minghai Hou, Guiwen Yang, Guiqin Liu, Wenqiang Liu, Jiao Guo, Shanshan Pan, Guangyi Fan, Wei Zhang, Ruitao Zhang, Jie Yu, Xinhao Zhang, Qi Yin, Chuanliang Ji, Yuanchun Jin, Guidong Yue, Mei Liu, Jiake Xu, Shimin Liu, Jordi Jordana, Antonia Noce, Marcel Amills, Dong Dong Wu, Shuaicheng Li, Xiangshan Zhou, Jifeng Zhong

**Affiliations:** 1 Dairy Cattle Research Center, Shandong Academy of Agricultural Sciences, Ji’nan, 250100 China; 2 Liaocheng Research Institute of Donkey High-Efficiency Breeding and Ecological Feeding, Liaocheng University, Liaocheng, 252059 China; 3 National Engineering Research Center for Gelatin-based Traditional Chinese Medicine, Dong-E-E-Jiao Co. Ltd, Liaocheng, 252201 China; 4 BGI-Shenzhen, Shenzhen, 252201 China; 5 Department of Computer Science, City University of Hong Kong, Hong Kong, 999077 China; 6 College of Life Science, Shandong Normal University, Ji’nan, 250000 China; 7 BGI-Qingdao, BGI-Shenzhen, Qingdao, 266555 China; 8 Jiangsu Key Laboratory for Molecular and Medical Biotechnology, Nanjing Normal University, Nanjing, 210023 China; 9 School of Biomedical Sciences, The University of Western Australia M508, 35 Stirling Hwy, Perth, WA 6009 Australia; 10 Department de Ciència Animal idels Aliments, Facultat de Veterinària, Universitat Autònoma de Barcelona, Bellaterra, 08193 Spain; 11 Department of Animal Genetics, Centre for Research in Agricultural Genomics (CRAG), CSIC-IRTA-UAB-UB, Campus de la Universitat Autònoma de Barcelona, Bellaterra, 08193 Spain; 12 State Key Laboratory of Genetic Resources and Evolution, Kunming Institute of Zoology, Chinese Academy of Sciences, Kunming, 650223 China; 13 Center for Excellence in Animal Evolution and Genetics, Chinese Academy of Sciences, Kunming, 650223 China; 14 State Key Laboratory of Bioreactor Engineering, East China University of Science and Technology, Shanghai, 200237 China; 15 Institute of Animal Science, Jiangsu Academy of Agricultural Sciences, Nanjing, 210014 China

**Keywords:** Population genetics, Evolutionary biology, Next-generation sequencing, Sequence annotation

## Abstract

Current knowledge about the evolutionary history of donkeys is still incomplete due to the lack of archeological and whole-genome diversity data. To fill this gap, we have de novo assembled a chromosome-level reference genome of one male Dezhou donkey and analyzed the genomes of 126 domestic donkeys and seven wild asses. Population genomics analyses indicate that donkeys were domesticated in Africa and conclusively show reduced levels of Y chromosome variability and discordant paternal and maternal histories, possibly reflecting the consequences of reproductive management. We also investigate the genetic basis of coat color. While wild asses show diluted gray pigmentation (Dun phenotype), domestic donkeys display non-diluted black or chestnut coat colors (non-Dun) that were probably established during domestication. Here, we show that the non-Dun phenotype is caused by a 1 bp deletion downstream of the *TBX3* gene, which decreases the expression of this gene and its inhibitory effect on pigment deposition.

## Abstract (web summary)

A new donkey reference genome and comparisons with wild asses yields insights into the evolutionary history of donkey domestication and identifies a genetic variant that results in the non-Dun coat colours of domestic donkeys.

## Introduction

The domestication of donkeys (*Equus asinus*) and horses (*Equus caballus*) dramatically enhanced human mobility and substantially facilitated the trading of goods between distant territories. There is a broad consensus that the domestication of donkeys began in the tropics or subtropics of Africa^1,2^, but the exact timing and precise location of this process remain unknown. Archeological remains of domestic donkeys have been found in the Egyptian predynastic sites of El Omari (6800–6500 years before present, ybp), Maadi (6000–6500 ybp) and Abydos (5000 ybp)^3–6^. However, the exact timeline of donkey domestication is still unclear since archeologists have also identified remains from African wild asses and early domestic donkeys, dating to ~8500 ybp^6,7^, in the site of Ash Shumah (Yemen). On the other hand, the exact locations of the centers of donkey domestication remain controversial. The identification of 5000-year-old ass skeletons in the pharaonic mortuary complex of Abydos, with osteological lesions typical of load carrying, provided evidence that donkeys might have been domesticated by Egyptian villagers in the Nile Valley^2^. Alternatively, it has been proposed that pastoralist peoples from the Horn of Africa domesticated donkeys as a strategy to cope with the consequences of the increasing aridity of the Sahara (7000–6500 ybp). A recent study based on microsatellite data supported this latter hypothesis^8^.

One of the main limitations to reconstructing the history of donkey domestication and dispersal is the lack of archeological remains, as well as of additional historical evidence such as rock paintings, textual records or engravings, particularly in sub-Saharan Africa, where such items are very rare^9^. One possible explanation for this outcome is the low status and prestige of donkeys compared with horses and camels^9^. It is also possible that the spread of donkeys was initially slow and scattered, experiencing a substantial enhancement only with the development of long-distance trade^9^.

Previous research indicated that the critically endangered Nubian wild ass (*Equus africanus africanus*) and an extinct relative of the Somali wild ass (*Equus africanus somaliensis*) (SOM) might be the wild ancestors of domestic donkeys^8^. However, these findings relied exclusively on analysis of the variability of mitochondrial DNA (mtDNA), which represents only the maternal lineage.

All wild asses including Tibetan kiang (*Equus kiang*) (KIA), Onager (*Equus hemionus onager*) (ONA), and Somali wild ass, display a Dun color, which is characterized by a dilution of the pigmentation combined with a few dark-colored body areas termed primitive markings (e.g. dorsal stripe). In contrast, non-Dun coat colors, such as black and chestnut, can be frequently seen in domestic donkeys (*E. asinus*). In horses, the Dun phenotype is explained by mutations impairing the expression of the TBX3 transcription factor.

In this work, we intend to elucidate the history of donkey domestication by using a population genomics approach based on whole-genome sequence data. In addition, we want to investigate the genetic basis of the non-Dun phenotype and seek to ascertain whether the same gene is involved in the determination of the Dun color in donkeys or if, conversely, the genetic basis of this ancient phenotype is completely different in horses and donkeys.

## Results

### Genome assembly and annotation

We constructed a de novo assembly of the donkey genome by using a state-of-the-art approach combining consensus validation with short reads, contig formation with long reads, and scaffolding by Hi–C (Supplementary Notes 1–4, Supplementary Figs. 1–6, Supplementary Tables 1–4, Supplementary Data 1). These combined technologies produced what is, to our knowledge, the most continuous de novo genome assembly of an odd-toed ungulate with chromosome-length scaffolds (Table 1, Supplementary Table 4). We also assembled the sex chromosomes of donkey (Supplementary Note 4). Several assessment approaches indicated the high quality of our assembly (Supplementary Tables 5–7), which exhibits a 24-fold and 6-fold improvement, in the scaffold N50, compared to those of the previously published donkey genomes reported by Huang et al.^10^. and Renaud et al.^11^, respectively. This assembly should facilitate the identification of fine-scale chromosomal rearrangements between horse and donkey with the aim of clarifying the evolutionary history of equine species.

**Table 1:** Quality metrics for the donkey genome assembly generated in the current work and for other donkey and horse genome assemblies published in previous studies.

	Donkey			Horse	
	This study	Renaud et al. (2018)	Huang et al. (2015)	EquCab3.0	EquCab2.0
Total number of scaffolds	43	9021	2167	4701	9687
N50 contigs	7.92 Mb	140.3 kb	66.7 kb	4.50 Mb	112 kb
N50 scaffolds (Mb)	93.37	15.4	3.8	87.23	46.75
Coverage	211×	61.2×	42.4×	88.0×	6.8×
Total bases (Gb)	2.432	2.320	2.391	2.507	2.475
Largest scaffold (Mb)	209.96	84.20	17.06	–	–
Total number of predicted protein-coding genes	21,983	18,984	23,850	–	20,421

According to our results, repetitive sequences accounted for 41.79% of the donkey genome (Supplementary Table 8, Supplementary Data 2). A total of 21,983 protein-coding genes were identified in the present assembly (see “Methods” section and Supplementary Note 4, Supplementary Tables 9–13). Expression profiles of these protein-coding genes in 13 tissues were analyzed via transcriptome sequencing (Supplementary Figs. 5 and 6, Supplementary Data 3). We calculated the proportion of protein-coding genes that are annotated in the horse genome (EquCab2.0)^12^ but not in our donkey assembly. To do so, we considered that each predicted protein-coding gene is represented by a single transcript and vice versa (Supplementary Fig. 7). Approximately 13.2% of the protein-coding genes in our donkey assembly (2914 genes out of 21,980) could not be assigned to the horse genome, a proportion that is higher than that published by Renaud et al.^11^. Moreover, 10.3% of the horse protein-coding genes (2197 genes out of 21,263) could not be assigned to our donkey assembly, a fraction that is lower than that reported by Renaud et al.^11^. The whole set of protein-coding genes identified in our assembly spanned a total of 815.38 Mb, which was ~33.52% of the whole assembly, while the protein-coding regions spanned 33.94 Mb.

We also performed gene function enrichment analysis for horse–donkey orthologs, horse-specific genes, and donkey-specific genes (Supplementary Table 14). The horse–donkey orthologs were enriched in protein-binding functions (adjusted *P*-value = 0.03). In contrast, horse-specific genes were enriched in cellular components including organelle part (adjusted *P*-value = 1.12E−11), intracellular organelle part (adjusted *P*-value = 2.72E−11), intracellular part (adjusted *P*-value = 1.78E−05), and mitochondrial membrane part (adjusted *P*-value = 0.014). Immune response (adjusted *P*-value = 0.0028) and enzyme inhibitor activity (adjusted *P*-value = 0.01) were enriched in the functional and molecular function categories, while donkey-specific genes were not enriched in any cellular, functional, or molecular categories.

Our chromosome-level donkey assembly has a larger scaffold N50 than the donkey assembly previously published by Renaud et al.^11^. To assess the heterozygosity of several representatives of the *Asinus* subgenus, we aligned shotgun sequencing data corresponding to one SOM, two Asian wild asses (AWAs), including an ONA (*E. hemionus onager*) and a KIA (*E. kiang*), and one domestic donkey^9,13,14^ against our donkey genome assembly. We found that the SOM was less heterozygous than the domestic donkey (Supplementary Table 15), which is in accordance with the findings of Renaud et al.^11^. Except for ONA, the heterozygosity rates obtained in our study for the SOM, KIA, and the domestic donkey (Supplementary Table 15) were higher than those reported by Renaud et al.^11^.

### Population genetics

We resequenced, with an average coverage of 10.9×, the genomes of 83 domestic donkeys and two Asian wild asses (two *E. hemionus* and one *E. kiang*) from nine countries (Supplementary Note 5, and Supplementary Figs. 8 and 9, and Supplementary Data 4). We also added to our dataset genome sequences from four Asian wild asses (one *E. hemionus*, one *E. hemionus onager*, and two *E. kiang*), one Somali wild ass (*E. africanus somaliensis*) and 43 domestic donkeys (Supplementary Data 4). A total of 133 genomes of 126 domestic donkeys and seven wild asses from nine countries were used for population genetics analyses. Analysis of these combined data generated a collection of 17.28 million SNPs and 1.5 million indels. Approximately 7.0 million SNPs and 0.66 million indels were detected among the 126 domestic donkeys (Supplementary Tables 16 and 17, Supplementary Data 5).

**Fig. 1:**
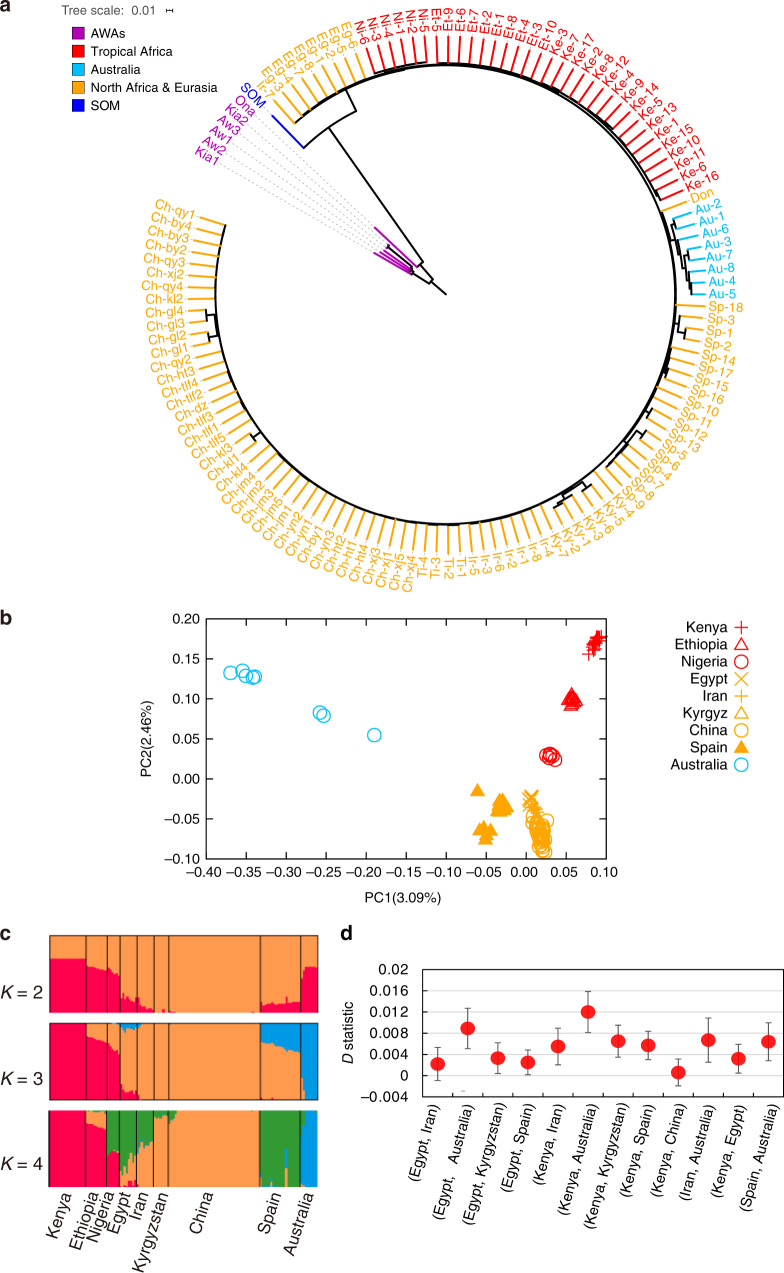
Population structure of domestic donkeys. **a** Neighbor-joining phylogenetic tree of 133 donkey and ass samples constructed using 16,582,014 autosomal SNPs based on pairwise identity-by-state (IBS) genetic distances. The Asian wild asses were set as an outgroup. AWAs Asian wild asses, SOM Somali wild ass. The following acronyms are used: Ke (Kenya), Ch (China), Ni (Nigeria), Ir (Iran), Sp (Spain), Eg (Egypt), Et (Ethiopia), Ti (Tibet), Au (Australia), and Don (the European donkey whose sequence was reported in a previous study^13^). **b** Principal component analysis (PCA) based on 6,825,163 autosomal SNPs identified in 126 domestic donkeys with different geographic origins. **c** Bayesian model-based clustering (from *K* = 2 to *K* = 4) of 126 domestic donkeys. Each vertical bar represents one individual. Each color represents one putative ancestral background, and the *y*-axis quantifies ancestry membership. **d** *D*-statistic tests in the form (((Population 1, Population 2), Somali wild ass), Asian wild ass) where Population 1 (P1) and Population 2 (P2) indicates donkeys from African or Eurasian countries. Data are presented as *D*-statistic ± 1 s.e.m. A positive *D*-statistic indicates that P1 shares more derived alleles with African wild ass than P2 does, while negative *D*-statistic indicates that P2 shares more derived alleles with African wild ass than P1 does. *D*-statistic and *Z* scores for each test can be seen in Supplementary Table 19. Source data are provided as a Source Data file.

Both the phylogenetic tree (Fig. 1a) and the principal component analysis (PCA) (Supplementary Fig. 10) separated the 133 individuals under analysis into two clades, one including all Asian wild asses and the other including the African wild ass and all domestic donkeys, thus confirming previous reports demonstrating that the domestic donkeys originated from African wild asses and not from Asian wild asses^1,2^.

PCA of only domestic donkeys (Fig. 1b, Supplementary Fig. 11) revealed three main clusters: (i) Tropical Africa cluster (Kenya, Ethiopia, and Nigeria), (ii) North Africa and Eurasia cluster (Egypt, Spain, Iran, Kyrgyzstan, and China), and (iii) Australia cluster, roughly corresponding to their continental distribution. Unfortunately, the absence of textual records and archeological remains as well as of pictorial and sculptural representations^9^ makes it very difficult to infer the routes of spread of donkeys across Africa. Blench^9^ proposed several potential paths of diffusion, none of which connect West and East Africa, but this does not necessarily imply the absence of gene flow between these two geographic areas. We have also observed that Australian samples clustered separately from their African, Asian, and European counterparts. The strong differentiation of Australian donkeys might be attributed to the limited number of founder individuals that were brought by the British colonizers to Australia 200 years ago^15^ as well as to the effects of prolonged geographic isolation and high genetic drift.

Individual ancestry coefficients were inferred with Admixture^16^ to further assess population structure (Fig. 1c). With *K* = 2, the genetic ancestry represented by garnet color appears mainly in populations from Kenya, Ethiopia, Nigeria, and Australia. With *K* = 3, which is also the optimal *K*-value (Supplementary Fig. 12, Supplementary Data 6), the third genetic ancestry (in blue) appears mainly in Australian donkeys and Spanish donkeys. When *K* = 4, the fourth genetic ancestry appears mainly in populations from Spain, Iran, Egypt, and Nigeria.

Based on the population structure analyses mentioned previously, domestic donkeys could be subdivided into the Tropical Africa group (including donkeys from Kenya, Ethiopia, and Nigeria) and the North Africa & Eurasia group (including donkeys from Egypt, Spain, Iran, Kyrgyzstan, China, and Australia). Population genetic analyses of the two groups were carried out to infer nucleotide diversities (*π*), Watterson’s estimators (*θ*_W_), minor allele frequency (MAF) distributions, and the linkage disequilibrium (LD) parameters (*r*^2^). The nucleotide diversity of the Tropical Africa group (*π* = 0.697 × 10^−3^) was lower than that of the North Africa & Eurasia group (*π* = 0.857 × 10^−3^). In contrast (Supplementary Table 18), the Watterson’s estimator of the Tropical Africa group (*θ*_W_ = 0.644 × 10^−3^) was higher than that of the North Africa and Eurasia group (*θ*_W_ = 0.576 × 10^−3^). The distribution of MAFs was similar in both groups (Supplementary Fig. 13), while the Tropical Africa group had a higher LD than the North Africa & Eurasia group (Supplementary Fig. 14). These results make it difficult to infer whether there are one or, as proposed by Beja-Pereira et al.^1^, two primary donkey domestication sites.

We used the *D*-statistic^17^ to study the relations between the Somali wild ass and the two domestic donkey groups. The *D*-statistics were calculated in the form of (((Population 1, Population 2), African wild ass), Asian wild ass) where Population 1 (P1) and Population 2 (P2) were represented by donkeys from African or Eurasian countries. A positive *D*-statistic indicates that gene flow existed between P1 and African wild ass while a negative *D*-statistic indicates that gene flow took place between P2 and African wild ass. Among the 12 tests (Fig. 1d, Supplementary Table 19), the form (((Kenya, Australia), African wild ass), Asian wild ass) has the highest *D*-statistic and (((Egypt, Australia), African wild ass), Asian wild ass) has the second highest *D*-statistic, indicating that the Tropical Africa group (including donkeys from Kenya) is genetically closer to the African wild ass than Egyptian donkeys, and that Egyptian donkeys are more closely related to the African wild ass than Eurasian donkeys, suggesting a result consistent with the African domestication of donkeys and their subsequent spread to Europe and Asia.

To detect potential introgression events among domestic donkey populations, we performed a combination of analyses, including *D*-statistic^17^ and TreeMix^18^ analyses. *D*-statistic tests were applied by considering the Asian wild asses as an outgroup. This analysis indicated that introgression events existed between different geographic populations (*Z* scores < −3, Supplementary Table 20), for example, between Ethiopian donkeys and Egyptian donkeys, between Nigerian donkeys and Egyptian donkeys, between Ethiopian donkeys and Nigerian donkeys, and between Spanish donkeys and Egyptian donkeys. For Australian donkeys, a potential gene flow with their Egyptian and Spanish counterparts was detected, but Australian donkeys were genetically closer to Spanish donkeys than to Egyptian donkeys (Supplementary Table 20). TreeMix analysis also confirmed the existence of gene flow between donkey populations from different countries and continents (Supplementary Fig. 15).

### Domestic donkeys have similar historic effective population sizes

**Fig. 2:**
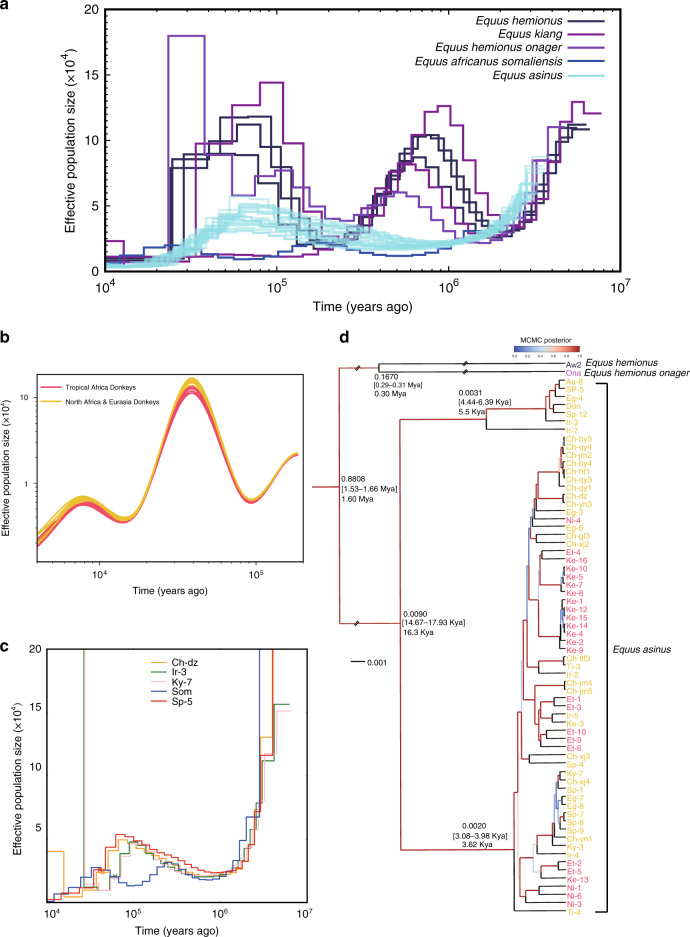
Demographic history of Asian wild ass, Somali wild ass, and domestic donkeys. **a** Demographic trajectories revealed by PSMC. The time scale on the *x*-axis is calculated assuming a mutation rate of 7.242 × 10^−9^ mutations per generation and site, while the assumed generation time is 8 years^11,87^. Bootstrapping confidence intervals for each sample are shown in Supplementary Data 7. **b** Demographic history of Tropical African donkeys and North African & Eurasian donkeys revealed by SMC++. **c** Potential admixture events and corresponding times inferred with a model based on PSMC. Autosomal SNPs from randomly selected domestic donkeys (Ch-dz, Ir-3, Ky-7, and Sp-5) and the Somali wild ass were used in this analysis. The admixture events were indicated by crosses on the curves. **d** Phylogenetic tree based on Y chromosome SNPs from wild asses and domestic donkeys. Sample names in gold represent North African & Eurasian donkeys while sample names in red represent Tropical African donkeys. A total of 13,032 SNPs mapping to the Y chromosome were used to construct the tree. BEAST 2 was applied in this phylogenetic analysis. The parameters for generating the maximum clade credibility (MCC) tree are HKY for the site model, strict clock model for the clock model (clock rate = 1), and Yule model for tree priors. The phylogenetic tree was generated by Bayesian Markov chain Monte Carlo (MCMC) with 1000 simulations. The numbers beside the nodes indicate the estimated node height, the divergence time, and the corresponding 95% confidence intervals. Kya refers to 1000 years ago and Mya refers to million years ago. The node statistical support was assessed by MCMC posterior probability indicated by the edge color linked to the node. In Fig. 1c, d, the following acronyms have been used: Ke (Kenya), Ch (China), Ni (Nigeria), Ir (Iran), Sp (Spain), Eg (Egypt), Et (Ethiopia), Ti (Tibet), Au (Australia), and Don (the European donkey which sequence was reported in a previous study^13^).

By taking into account whole-genome data from single individuals, we utilized the pairwise sequentially Markovian coalescent (PSMC) model^19^ to infer the local time to the most recent common ancestor (TMRCA) as well as to assess changes in the historic effective population size (*N*_e_), measured by the mutation-scaled rate (Fig. 2a, Supplementary Data 7). Our results are consistent with those of Renaud et al.^11^. All the curves of domestic donkeys mixed well, indicating that donkey domestication is a recent event. The ancestral *N*_e_ values of *E. africanus somaliensis* and *E. asinus* diverged ~0.11 million years ago (mya). Much later, donkeys were domesticated about 7000–9000 years ago (kya), as assessed through the analysis of archeological evidence^9^. In addition, we inferred the demographic history of Tropical African donkeys and North African & Eurasian donkeys with SMC++ software^20^. The *N*_e_ curves of these two groups of donkeys were indistinguishable (Fig. 2b), indicating that these donkeys were probably derived from the domestication of one common ancestral group or at least two ancestral groups with a similar biogeography. Our analyses lack enough resolution to infer whether donkeys were domesticated in one or several locations. The study of ancient DNA will be crucial to elucidate this essential question.

We also built a statistical model based on PSMC to detect the most recent possible admixture event time of ancestral species of domestic donkey from autosome-sequencing data (see “Methods” section, Supplementary Fig. 16, Supplementary Data 8). This model indicated that historical admixture events took place ~40 to 60 kya in wild ancestral species of domestic donkeys (Fig. 2c), which was concordant with the variation trend of *N*_e_ estimated with PSMC (Fig. 2a), as the population split and admixture events were expected to generate a hump in the historical population size.

### Donkeys have different paternal and maternal demographic histories

The analysis of 126 domestic donkey genomes showed that the SNP density on the sex chromosomes was dramatically lower than that on the autosomes (Supplementary Table 20). The marked differences in the genetic diversities of the X and Y chromosomes can be attributed to multiple causal factors associated with the mutation rate, *N*_e_, demography, and evolutionary selection^21^. Interestingly, a similar genetic pattern has been observed in horse^22^, which is also a polygynous species with dominant males defending large territories.

To determine the patrilines of donkeys, we constructed a phylogenetic tree for male donkeys with SNPs mapping to the Y chromosome (Fig. 2d). The tree outlined the divergence time for the Y chromosome of wild asses and domestic donkeys. We scaled the results to a real temporal scale by setting the divergence time between *E. africanus somaliensis* and *E. kiang* to 1.61 mya, as previously reported^13^. Our results based on Y chromosome SNPs show that the ancestral species of *E. africanus* and *E. hemionus* separated between 1.55 and 1.66 mya (95% confidence interval) (Fig. 2d and Supplementary Data 9). The common ancestral species of all domestic donkeys (*E. africanus)* separated into two clades ~14.67–17.93 kya (95% confidence interval), and both clades further split into diverse donkey breeds in a window of time from 5.5 to 3.62 kya (Fig. 2d and Supplementary Data 9). We also constructed a phylogenetic tree with SNPs mapping to the mitochondrial genome for all donkeys and asses (Supplementary Fig. 17, Supplementary Data 10), and the result was consistent with the estimated time of donkey domestication. Remarkably, the estimated time at which the ancestral species of *E. africanus* split into two clades according to Y chromosome data (14.67–17.93 kya) does not match the estimated divergence time of the two mitochondrial clades (0.303–0.910 mya) reported by Beja-Pereira et al.^1^ and confirmed in the current work (Supplementary Fig. 17, Supplementary Data 10). These results suggest a different demographic history for maternal and paternal donkey lineages.

### Positive selection of the *TBX3* gene modified the pigmentation patterns of donkeys

**Fig. 3:**
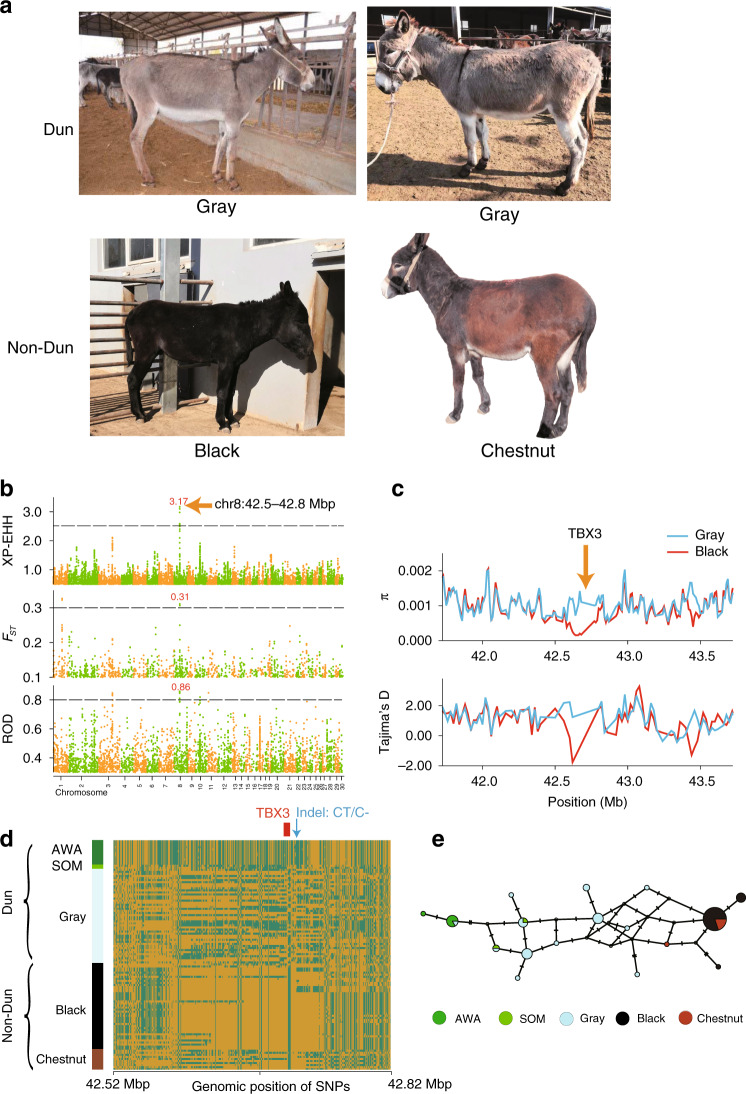
Selective scans for the Dun phenotype of donkeys. **a** Donkeys with Dun (gray) and non-Dun (black and chestnut) coat colors. **b** Whole genome scan with XP-EHH, *F*_ST_, and ROD. The orange arrow indicates the genome position of the strongest selective signal for the Dun phenotype. The numbers in red are the values of the three statistics for the strongest selective signal. The dashed lines represent the empirical threshold values for the three methods: 2.5 for XP-EHH, 0.3 for *F*_ST_, and 0.8 for ROD. **c** Nucleotide diversity and Tajima’s *D* corresponding to the Dun selective sweep on chromosome 8 which encompasses the *TBX3* gene. **d** Plot of the haplotype structure of SNPs around the *TBX3* gene in all donkeys and wild asses. AWA refers to Asian wild asses. SOM refers to Somali wild ass. Genotypes identical or different to those of our donkey assembly are represented with yellow and green colors, respectively. The red rectangle shows the position of the *TBX3* gene (from 42,723,946 bp to 42,735,174 bp). The blue arrow indicates the position (42,742,556 bp) of the candidate causal 1 bp deletion for the Dun phenotype. **e** Median-joining haplotype network based on the SNPs from the 5′ end of the *TBX3* gene (42,723,946 bp) to its downstream 20,000 bp (42,743,946 bp) on chromosome 8. Each circle represents one haplotype, and the size of the circle indicates the number of individuals harboring the haplotype. Lines with dashes between circles represent the mutational steps between haplotypes. One dash represents one mutation and two dashes represent two mutations. Only SNPs with minor allele frequencies ≥0.1 were considered in this analysis.

Coat color is one of the main traits that were selected during the domestication process. The base color of donkeys can be black or red (chestnut)^23^. There is a dominant *Dun* allele causing strong dilution of the pigmentation, which becomes gray or light chestnut/rose Dun, in contrast to the recessive *non-Dun* allele (black or chestnut, as mentioned previously). The Dun coat is considered to be the ancestral wild type^24^, while the non-Dun coat is found exclusively in domestic animals. In this study, we compared 25 Dun donkey samples and 23 non-Dun donkey samples from different geographic regions to identify the genomic region responsible for this coloration pattern (Supplementary Data 11). We used three methods to search for the causal mutations, including the fixation index for diversity differentiation (*F*_ST_), extended haplotype homozygosity (XP-EHH) test and reduction of diversity (ROD). The overlap of the selective signals consistently detected with the three methods revealed that the most significant region was located on the 42–43 Mb interval of chromosome 8 (Fig. 3a). The analysis based on *π* and Tajima’s *D* supported a strong selective signal in this region (Fig. 3b). By plotting the phased SNPs, the selective sweep was finely mapped to the 42,587,636–42,781,262 bp interval (Fig. 3c), which contains the T-Box 3 gene (*TBX3*, EAS0007835 in the Ensembl database). The only *TBX3* polymorphism fully associated with the Dun phenotype was a 1 bp deletion (*chr8:g.42742556 CT*>*C−*). This deletion showed homozygous or heterozygous genotypes (*CT*/*CT* or *CT*/*C−*) in all Dun donkeys, while all non-Dun donkeys were homozygous for the deletion (*C−*/*C−*) (Supplementary Table 21). These results indicate that the non-Dun pigmentation emerged as a result of the loss of 1 bp in the *TBX3* gene. This 1 bp deletion (*chr8:g.42742556 CT*>*C−*) is located ~18.6 kb downstream of the transcription start site of the *TBX3* gene.

**Fig. 4:**
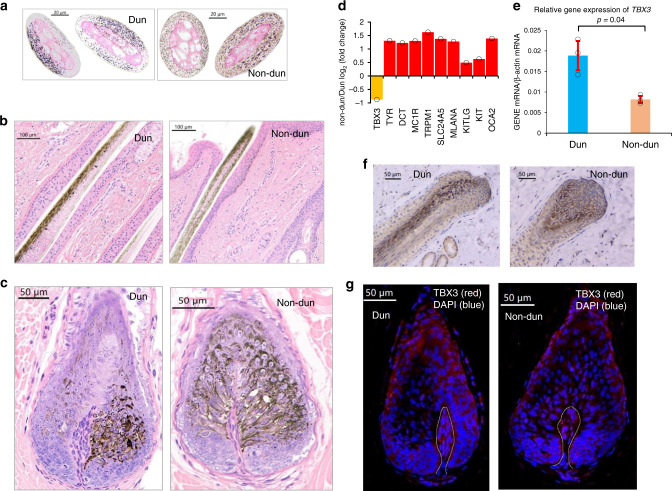
Phenotypic characterization and differential gene and protein expression in the croup skin of Dun and non-Dun donkeys. **a** Micrographs of cross-sections of hairs from Dun and non-Dun donkeys, images representative of three experiments **b** Micrographs of skin and hair sections from Dun and non-Dun donkeys stained with hematoxylin and eosin, images representative of three experiments. **c** Micrographs of sections of anagen hair follicles from Dun and non-Dun donkeys stained with hematoxylin and eosin, images representative of three experiments. **d** Bar plot of differential mRNA expression (log_2_-transformed fold change) of 10 pigmentation-related genes expressed in the skin of non-Dun (*n* = 3) versus Dun (*n* = 3) donkeys. Upregulated and downregulated genes are indicated with red and orange bars, respectively. Black circles indicate the data points. **e** Relative croup skin *TBX3* mRNA levels (mean ± s.e.m.) as assessed by quantitative RT-PCR in Dun (*n* = 3) and non-Dun (*n* = 3) donkeys (statistical significance of a two-tailed *t*-test is indicated). Black circles indicate the data points. Source data are provided as a Source Data file. **f** Micrographs of immunohistochemistry of the *TBX3* protein in sections of hair follicles from the croup of Dun and non-Dun donkeys, images representative of three experiments. **g** Micrographs of immunofluorescence analysis of the *TBX3* protein (red) in sections of anagen hair follicles from the croup of Dun and non-Dun donkeys, images representative of three experiments. DAPI staining is indicated with blue color, and white lines show the basement membrane. Scale bars were defined in each image.

We investigated the biochemical basis of the Dun phenotype in donkeys. Hematoxylin and eosin (H&E) staining of cross-sections of croup hairs revealed that hair pigment is differentially deposited in Dun and non-Dun donkeys (Fig. 4a). In this manner, hair pigment is evenly distributed in the hair cortex of non-Dun donkeys. Conversely, a radial and asymmetrical distributions of pigment in the hair cortex of Dun donkeys is observed. As expected, pigmentation in Dun donkeys is markedly diluted. Pigment granules in hairs from the croup of the Dun donkey are limited to approximately less than half of the cortex (diluted hairs). This result is consistent with observations made by Imsland et al.^25^ in horses. Histological sections of croup skin indicated that in Dun donkeys, pigments are densely distributed on the outward side of the hair follicle and sparsely distributed on the inward side. In contrast, in non-Dun donkeys, pigments are evenly distributed on both sides of the hair follicle (Fig. 4b). Histological sections of anagen hair follicles revealed that in Dun donkeys, the uneven distribution of pigments starts in the hair bulb, while in non-Dun donkeys, this asymmetric pattern of hair pigmentation is disrupted (Fig. 4c, Supplementary Fig. 18).

To understand the role of the 1 bp deletion presumably involved in the determination of the non-Dun phenotype, we compared the expression levels of *TBX3* and other genes regulating melanocyte pigment production in Dun and non-Dun donkeys. We first assessed the genotype of the 1 bp deletion in three Dun and three non-Dun donkeys by Sanger sequencing. This analysis demonstrated that the genotype of the 1 bp deletion was *CT/C*− in all Dun donkeys and *C−/C*− in all non-Dun donkeys. Then, we performed transcriptome sequencing of croup skin from three Dun and three non-Dun donkeys. Among the differentially expressed genes, we found that *TBX3* mRNA was downregulated 1.8-fold in non-Dun skin (Fig. 4d, Supplementary Data 12), and this observation was confirmed by quantitative RT-PCR (downregulated by 2.3-fold in non-Dun skin, Fig. 4e). We also identified nine genes (*DCT*, *KIT*, *KITLG*, *MC1R*, *MLANA*, *OCA2*, *SLC24A5*, *TRPM1*, and *TYR*) that are known to regulate pigment production in melanocytes. These genes showed upregulated expression in non-Dun croup skin (Fig. 4d, Supplementary Data 12). This result is consistent with the increased pigment content in non-Dun hair. We also compared the expression of melanogenic or melanocyte regulatory genes in the dorsal stripe skin of Dun donkeys and skin from the corresponding location in non-Dun donkeys, but we did not obtain evidence of differential expression (Supplementary Table 22).

To illustrate the relationship between pigment deposition patterns and the profile of *TBX3* mRNA expression, we carried out immunohistochemical and immunostaining assays of *TBX3* expression in croup skin sections of Dun and non-Dun donkeys. Immunohistochemistry of a Dun croup skin section showed that *TBX3* is expressed on only one side of the longitudinal axis of the hair follicle and that pigment is distributed on the other side (Fig. 4f). In the section of anagen hair follicles from the Dun donkey, *TBX3* immunostaining was observed in a subset of keratinocytes in the developing hair cortex and in the outer cuticular layer. In contrast, in the non-Dun donkey, immunostaining was observed only in the outer cuticular layer of the hair follicle (Fig. 4g, Supplementary Fig. 18). These results suggest that the expression of *TBX3* in cortical keratinocytes suppresses the deposition of pigment, thus generating the asymmetrical distribution pigmentation pattern characteristic of Dun donkeys. It is likely that the 1 bp deletion inhibits, to some extent, the expression of *TBX3* in the hair follicles of non-Dun donkeys, thus suppressing the abrogating effect of *TBX3* on pigment deposition. In addition, we analyzed the selective sweep in Asian wild asses, Somali wild ass and chestnut donkeys. The gray-colored Asian wild asses and Somali wild ass were homozygous for the allele *CT*, suggesting that *CT/CT* is the ancestral genotype. The chestnut and black donkeys were both *C−/C−* and showed similar phased haplotypes in the selective sweep. The median-joining haplotype network (Fig. 3d) for the *TBX3* region (from 42,726,257 to 42,743,062) encompassing the short indel, indicated that the chestnut and black coats were more likely derived from gray-colored domestic donkeys than directly from wild donkeys.

### Convergent selection for the Dun phenotype in horses and donkeys

In horses, two variants associated with the non-Dun phenotype, i.e., the G>T mutation at chr8:18,227,267 (horse *non-Dun1* allele) and the 1609 bp deletion (horse *non-Dun2* allele), are also located ~18 kb downstream of the transcription start site of *TBX3*^25^. The alignment of sequences containing the *non-Dun1* allele of horse with the donkey sequence that harbors the 1 bp deletion mentioned previously indicated that these sequences are homologous (Supplementary Fig. 19). Moreover, the 1 bp deletion is located 25 bp downstream of the *non-Dun1* causal substitution (Supplementary Fig. 19). Careful inspection of both Dun and non-Dun donkey *TBX3* sequences indicated that there was no polymorphism equivalent to the horse *non-Dun2* allele (Supplementary Fig. 19). Likewise, the horse *non-Dun1* allele identified in horses did not segregate in donkeys. Moreover, the 1 bp deletion detected in the current work was located within the 1609 bp deleted region corresponding to the horse *non-Dun2* allele. These results indicate that the same causal gene (*TBX3*) is involved, but the causative mutations for the non-Dun phenotype in donkeys and horses occurred independently after the divergence of these two species. Imsland et al.^25^ inferred that the binding sites for NF-Y and NF-I, which are CCAT box-binding transcription factors, were altered in horses harboring the *non-Dun1* allele. The binding sites for ALX4 and MSX2, which are known transcription factors participating in hair follicle development^26–28^, were deleted in horses carrying the *non-Dun2* allele. Transcription factor affinity prediction with TRAP^29^ indicated that the short deletion in the *TBX3* gene of non-Dun donkeys involves the suppression of one binding site for the transcription factor NFIC, which is essential for TGF-β-dependent hair follicle cycling^30^. In Dun donkeys, the binding site for NFIC is CTGGC, while in non-Dun donkeys, the 1 bp deletion generated a motif (CGGC) that cannot bind NFIC (Supplementary Fig. 19). Moreover, comparative mapping indicated that the region 1000 bp upstream and downstream of the *CT/C*− indel corresponds to the 114,663,952–114,689,575 interval on human chromosome 12 (version GRCh38), which contains an enhancer (GH12F114663, chr12:114,663,952–114,689,575).

In horses, the Dun phenotype, which is also ancestral, is characterized by a generalized dilution of the pigmentation except in certain areas of the body, e.g., the dark dorsal stripe. Dun coloration is assumed to enhance camouflage from predators^25^. As previously stated, the non-Dun phenotype (no color dilution) is predominant in most domestic horses and is explained by regulatory mutations in the *TBX3* gene. In the non-Dun skin of horses, the expression of the *TBX3* gene is also downregulated. We detected the upregulated expression of several pigmentation genes in the skin of non-Dun donkeys (Supplementary Data 12, Supplementary Note 6), thus showing that the molecular mechanism of pigment dilution is very similar in horses and donkeys.

## Discussion

The history of donkey domestication is controversial. Previous mitochondrial data of donkey indicated the existence of two highly differentiated clades. Our mitochondrial data agree well with the existence of two clades that diverged 0.303–0.910 mya, but population structure data based on the analysis of whole-genome sequences indicate that modern Tropical African donkeys and North African & Eurasian donkeys coalesced before 6000 ybp. Interestingly, analysis of extensive mitochondrial data sets has shown that Somali wild asses are distinct from Nubian wild asses and domestic donkeys from clades 1 and 2^31^. These results suggest that both clades might have a Nubian-like ancestry^31^. Interestingly, wall paintings and other iconography of domestic donkeys began in Egypt in the fourth millennium BC, while no representations of wild asses or domestic donkeys have been found in the Horn of Africa^9^. Moreover, the analysis of ancient Egyptian wall paintings has shown that the process of differentiation of donkeys from their wild relatives can be tracked through the progressive disappearance, during the transition from the Old Kingdom to the Middle Kingdom, of the dark shoulder stripe of the wild ass from donkeys^9^. Such depictions of the gradual transformation of wild asses into domestic donkeys are completely absent in other parts of Africa^9^. The scarcity of archeological remains and other sources of historical information reinforces the need to sequence a broad collection of ancient and modern African samples from wild asses and domestic donkeys to clearly ascertain the geography and timing of donkey domestication.

We also observed a limited level of variability in the donkey Y chromosome, a result that is consistent with previous findings in horses^32^. While horses display high levels of mitochondrial diversity, the study of Y chromosome data has evidenced low variation^32^. Until recently, it was thought that the decline in horse Y chromosome variability began 5500 years ago as a result of founder effects associated with the domestication process, i.e., most likely, a limited number of stallions were incorporated into the domestic stock^33^. However, the analysis of horse ancient DNA revealed that stallion Y chromosome diversity remained high until at least 2000 ybp^34,35^ with a steady reduction then^35^. Indeed, Byzantine horses (287–861 CE) and horses from the Great Mongolian Empire (1206–1368 CE) already displayed reduced variation (although larger than the current variation)^35^. More recently, in the post-Renaissance period, the predominance of certain stallion lines caused a 3.8- to 10.0-fold reduction in Y chromosome diversity^35^, exemplifying the strong impact of breeding and artificial selection on equine paternal diversity. The most prevalent view is that the limited paternal variability observed in modern horse breeds was mainly produced by the reproductive strategies employed during the last two millennia, which relied on the extensive use of a limited number of specific stallion lines that became predominant^35^. This is illustrated by the dominance of an ~1000- to 700-year-old oriental haplogroup in most modern studs^34^. The analysis of ancient donkey genomes will be fundamental for determining whether jack Y chromosome diversity was high at the initial stages of domestication, and then declined as a result of mating practices similar to those implemented in horse breeding.

Livestock species were domesticated at different locations and historical times, but, compared to their wild ancestral species, domestic animals display a few common features (increased tameness, existence of multiple coat colors, increased reproduction, earlier onset of sexual maturity, etc.) that were established through artificial selection^36^. Currently, there is much debate about whether this phenotypic convergence is explained by common mechanisms leading to genetic convergence (homologous causal genes or mutations) or by the action of distinct sets of genes^37^. According to Glémin and Bataillon^38^ and Martínez-Ainsworth and Tenaillon^39^, genetic convergence is a rare phenomenon in cultivated plants, and similar domestication traits are generally controlled by different loci. However, the generalized lack of knowledge about the genes involved in the domestication process makes it difficult to assess whether genetic convergence is the rule or the exception. In the current work, we demonstrated that the dilution of the coloration typical of the Dun phenotype displays very similar microscopic and macroscopic features in horses and donkeys. More importantly, we were able to demonstrate that the same gene, *TBX3*, is responsible for the Dun pattern of pigmentation in both species, and we also showed that the causal mutation of the non-Dun phenotype in donkeys is a 1 bp deletion with a probable regulatory effect. Similarly, in horses, the non-Dun phenotype is explained by two deletions with regulatory effects^25^. In another study, Vickrey et al.^40^ provided evidence that head crests in domestic rock pigeons (*Columba livia*) and ring neck doves (*Streptopelia risoria*) are produced by different mutations in the ephrin receptor B2 gene. Moreover, the analysis of the genomes of sheep, goats, and their wild ancestral species demonstrated that approximately half of the genes showing selection signatures in *Ovis*, show congruent signatures in *Capra*^41^. Here, we showed that the *TBX3* gene was convergently selected in horses and donkeys. Although this particular case cannot be generalized to other phenotypes, it emphasizes the need to precisely clarify the role of convergent evolution in the fixation of Mendelian phenotypes that have been recurrently targeted by selection in multiple domestic species.

## Methods

Animal care and research procedures were carried out in accordance with the guiding principles for the care and use of laboratory animals and were approved by the Institutional Animal Care and Use Committee of Shandong Academy of Agricultural Sciences (SAAS).

### Genome sequencing

#### Sample information

A purebred DZ donkey was used for genome sequencing (Supplementary Note 1). Genomic DNA was extracted from blood by using the Puregene Tissue Core Kit A (Qiagen, Beijing, China).

#### Illumina short-read sequencing

Genomic DNA was sequenced on an Illumina HiSeq 2000 sequencing platform (Illumina, CA, USA). We constructed nine different short-insert (170, 250, 500, 800 bp) and mate-pair (2, 5, 10, 20, 40 kb) libraries and they were sequenced on 27 lanes.

#### PacBio library construction and sequencing

The preparation and sequencing of the SMRTbell DNA library were performed according to the manufacturer’s protocols (Pacific Biosciences, CA, USA). The SMRTbell library with 20 kb insert size was generated with the BluePippin system (Sage Scientific, MA, USA). Eleven SMRT cells were loaded and run on the Sequel System, which utilizes single molecule, real-time (SMRT) sequencing with fluorescently labeled nucleotides^42^.

#### Hi-C library construction and sequencing

To map the chromatin contacts of the DZ donkey genome, an in situ Hi-C protocol was developed as described in Rao et al. (2014)^43^ with customized adjustments: two to five million cells were crosslinked with 1% formaldehyde for 10 min at room temperature; nuclei were permeabilized; DNA was digested with 100 units of MboI, and the ends of restriction fragments were labeled using biotinylated nucleotides and ligated in a small volume; after reversal of cross-links, ligated DNA was sheared to a size of 200–400 bp with a Covaris instrument LE220 (Covaris, MA, USA), and the biotin-containing fragments were captured on streptavidin-coated beads. The resulting library was sequenced by using a BGISEQ-500 instrument to yield 100 bp paired-end sequence reads, thus providing an approximate 56× coverage of the donkey genome.

### Genome assembly

A hybrid de novo assembly was built using both Illumina short reads and PacBio long reads. A schematic diagram of the assembly pipeline is shown in Supplementary Figs. 2 and3. Initially, Illumina paired-end reads were assembled with SOAPdenovo v2.04.4 to construct short but accurate contigs^44^. The resulting Illumina contigs were combined with the PacBio reads to perform hybrid assembly with the DBG2OLC genome assembler^45^. The Sparc module of the DBG2OLC assembler^46^ was utilized to generate a consensus sequence in which long and highly accurate overlapping sequences were produced by correcting errors in the longest reads using shorter reads from the same library. Subsequently, the genome assembly was polished by considering sequence information from Illumina paired-end reads. The Illumina reads were mapped into the hybrid contigs using bwa^47^, and the alignment was subsequently used to further correct the assembly with Pilon v1.22^48^. Finally, scaffolding was carried out with BESST v2.2.7^49^ by using Illumina mate pair reads. To obtain the final assembly, PBJelly, a module of the PBSuite package v15.8.24^50^, was used to close or shrink gaps using PacBio reads.

The HiC-Pro (v2.8.0) pipeline^51^ was used for Hi-C data quality control using Bowtie 2^52^ with options (–very-sensitive -L 30–score-min L,−0.6,−0.2 --end-to-end -reorder) and the following parameters: MIN_INSERT_SIZE = 50; MAX_INSERT_SIZE = 1500; MIN_FRAG_SIZE = 100; MAX_FRAG_SIZE = 100,000; IGATION_SITE = GATC. Then, valid reads were extracted according to the HiC-Pro results. The de novo assembly of the chromosome-length donkey genome was produced using the open-source tools Juicer (v1.5)^53^ and 3d-dna (v170123)^54^ to generate a Hi-C contact matrix at a fine resolution (*r* = 100 kb) and pseudochromosomes with parameters (-m haploid -s 0 -c 5). The 3d-dna software was used to assemble the chromosome-length genome, combined with the PacBio assembly draft genome. By combining the Hi-C data and information from the collinearity analysis among donkey, horse, and human genomes^55^, we were able to infer that the donkey genome is distributed in 30 autosomes, two sexual chromosomes (X and Y) and one mitochondrial circular chromosome.

### Scaffold anchoring

Yang et al.^55^ utilized a complete set of chromosome-specific painting probes for horse to determine the regions of homology between human and donkey, and horse and donkey. In this manner, they established a genome-wide homology map of these three mammalian species. We used BLASTZ to anchor our assembled donkey scaffolds to the corresponding chromosomes^56^. By using the homology map of human, horse, and donkey as a reference for genomic coordinates, we were able to reliably assign scaffolds to donkey chromosomes. For instance, we inferred that donkey chromosome 11 is homologous to horse chromosome 17 and to human chromosome 13. Since the BLASTZ alignment results indicated that one of the donkey scaffolds maps to both horse chromosome 17 and human chromosome 13, we concluded that this scaffold can be safely assigned to donkey chromosome 11.

### Sex chromosome determination and assessment

The X chromosome assembly was built by considering collinearity mapping with horse X scaffolds. To assemble the donkey Y chromosome, we mapped 20 public donkey Y chromosome markers to our scaffolds^22^. We used our resequenced mapping data to determine the average sequencing depth of sex chromosomes. The sequencing depth of autosomes was defined as “normal”. If the average sequencing depth of sex chromosomes was approximately half of the average sequencing depth of autosomes, the sequencing depth was defined as “half”. If the average sequencing depth of sex chromosomes was nearly zero, the sequencing depth was defined as “zero”. For all 126 domestic donkeys, the average sequencing depths of X in female individuals were “normal” and in male accessions were “half”; meanwhile, the average sequencing depths of the Y chromosome in female accessions were “zero” and in male accessions were “half”. These results confirmed the high quality of our sex chromosome assembly. The nonrecombining region of the Y chromosome (NRY) was identified according to two criteria: (i) it cannot be found in female genomes (depth = 0) and (ii) its sequencing depth must be half for at least 80% of the jacks.

### Assessment of the genome assembly

To assess the completeness of our genome assembly, ~145.76 Gbp of Illumina reads generated from short-insert libraries (250, 500, 800 bp) were mapped to the donkey genome assembly by using BWA software with default parameters^47^. Subsequently, we used BamDeal-0.19 (https://github.com/BGI-shenzhen/BamDeal) to calculate sequencing depth. To check the completeness of coding regions, all transcriptome sequences from 13 tissues (brain, heart, kidney, liver, lung, muscle, skin, spleen, stomach, blood, cecum, epididymis, and testis) were assembled into unigenes with Trinity^57^. Next, unigenes were mapped to the assembled genome sequence with BLAT, and the coverage rate was duly assessed.

### Genome annotation

#### Repeat annotation

To identify repeat sequences in the donkey genome, we searched for both tandem repeats and transposable elements (TEs). Tandem repeats were detected using Tandem Repeats Finder 4.04 software^58^ with the following settings: Match = 2, Mismatching penalty = 7, Delta = 7, PM = 80, PI = 10, Minscore = 50, MaxPeriod = 2000. TEs were identified using a combination of homology-based and de novo approaches. The homology-based approach used standard databases for known repetitive sequences (e.g., RepBase17.01) and predicted TEs at both the DNA and protein levels^59^. At the DNA level, RepeatMasker (v4.0.4) was applied by considering data from the Repbase library (http://www.repeatmasker.org/), while at the protein level we used RepeatProteinMask by considering information from the Repbase library. For de novo prediction of TEs, RepeatModeler 1.05 software (www.repeatmasker.org/RepeatModeler) was employed. In the RepeatModeler analysis, we used two de novo repeat-finding programs, i.e., RECON (1.08)^60^ and REPEATSCOUT (v1.0.6)^61^, which employ complementary computational methods for identifying repeat element boundaries and family relationships from sequences. The results obtained with RepeatModeler were merged into a library, which was used by RepeatMasker to find homologous repeats in the donkey genome and to categorize them. In addition, we used LTR_finder^62^ to identify long terminal repeat (LTR) sequences in the donkey genome.

#### Gene annotation

To predict mRNA-encoding genes in the donkey genome, we performed both de novo and homology-based predictions. For the homology-based predictions, we used protein data from five species (*Homo sapiens*, *Bos Taurus*, *E. caballus*, *Sus scrofa*, and *Mus musculus*) that were retrieved from the Ensembl (release 64) database and mapped to the donkey genome with TBLASTN (version 2.2.26)^63^ by considering an *E*-value of 1e−5 as the threshold of significance. Subsequently, we selected the most homologous protein for each genomic locus showing multiple matches. To make de novo predictions, we used AUGUSTUS (version 2.5.5)^64^ and GENSCAN (version 1.0)^65^. The gene model parameters for AUGUSTUS were trained using homologous horse protein sequences, while the parameters for GENSCAN were derived from human studies. Genes with coding lengths smaller than 150 bp were filtered out to reduce false positives. GLEAN software (http://sourceforge.net/projects/glean-gene/) was employed to integrate evidence from the AUGUSTUS and GENSCAN predictions to generate a consensus gene set. To aid gene prediction, samples from 13 tissues (brain, heart, kidney, liver, lung, muscle, skin, spleen, stomach, blood, cecum, epididymis, and testis) retrieved from three donkeys were used to construct a normalized cDNA library. Transcriptome sequencing was performed with on the HiSeq 2000 platform. Transcriptome reads were mapped onto the genome to refine gene structures and in combination with GLEAN to yield the final protein-encoding gene set.

#### Gene function annotation

Gene functions were assigned according to the best match of the BLASTP alignment to the SwissProt and Translated EMBL Nucleotide Sequence Data Library (TrEMBL) databases (UniProt release 2011-01)^66^. Motifs and domains in the protein-coding genes were determined with InterProScan (v55.0)^67^, which searches several protein databases, including ProDom (ProDom, RRID:SCR_006969), PRINTS (PRINTS, RRID:SCR_003412), HAMAP (HAMAP, RRID:SCR_007701), Pfam (Pfam, RRID:SCR_004726), PIRSF (PIRSF, RRID:SCR_003352), PANTHER (PANTHER, RRID:SCR_004869), TIGRFAM (TIGRFAM, RRID:SCR_005493), SMART (SMART, RRID:SCR_005026), SUPERFAMILY (SUPERFAMILY, RRID:SCR_007952), Gene3D (Gene3D, RRID:SCR_007672), PROSITE (PROSITE, RRID:SCR_003457), COILS (COILS, RRID:SCR_008440), SignalP (RRID:SCR_015644), Phobius (PHOBIUS, RRID:SCR_015643) and TMHMM (http://www.cbs.dtu.dk/services/TMHMM/). Gene Ontology identifiers^68^ for each gene were obtained from the corresponding InterPro entry. We also mapped the reference genes to the KEGG pathway database^69^ and identified the best match for each gene.

### Transcriptome sequencing

#### Animal material and RNA isolation

We collected 17 samples corresponding to 13 distinct tissues (Supplementary Fig. 5) from three donkeys to perform RNA-seq (Supplementary Table 16). Samples were trimmed and cut into small pieces, cleaned with RNase-free and DNase-free water and immediately frozen in liquid nitrogen for storage until RNA isolation. Total RNA from each tissue sample was extracted by using TRIzol reagent (Thermo Fisher Scientific, CA, USA) according to the manufacturer’s instructions. The quality of RNA samples was evaluated with an Agilent 2100 Bioanalyzer (Agilent Technologies, CA, USA). RNA samples with a minimum RIN > 7.0 and a 28S/18S ratio > 1.0 were selected for sequencing.

#### Construction and sequencing of cDNA libraries

First, 200 ng of total RNA were purified by using oligo-dT beads, and then, poly(A)-containing mRNA was fragmented into small pieces with Fragmentation Buffer (Ambion, Thermo Fisher Scientific, CA, USA). First-strand cDNA was generated by employing First Strand Master Mix and Super Script II reverse transcriptase (Thermo Fisher Scientific). The reaction conditions were as follows: 25 °C for 10 min; 42 °C for 50 min; and 70 °C for 15 min. Then, Second Strand Master Mix (Thermo Fisher Scientific) was added to synthesize the second-strand cDNA (16 °C for 1 h). The purified fragmented cDNA was combined with End Repair Mix (Thermo Fisher Scientific) and incubated at 30 °C for 30 min. The end-repaired DNA was purified with AMPure XP Beads (Beckman Coulter, CA, USA). Then, A-Tailing Mix (ThermoFisher Scientific) was added and incubated at 37 °C for 30 min. Subsequently, poly(A)-containing mRNA was fragmented into small pieces with fragmentation buffer (Thermo Fisher Scientific). The 3′-adenylated DNA, RNA Index Adapter and Ligation Mix (Thermo Fisher Scientific) were mixed and incubated at 30 °C for 10 min. The end-repaired DNA was purified with AMPure XP Beads (Beckman Coulter). Several rounds of PCR amplification with PCR Primer Cocktail and PCR Master Mix (Thermo Fisher Scientific) were performed to enrich the cDNA fragments. Then, the PCR products were purified with AMPure XP Beads (Beckman Coulter, USA). The resulting library was quantitated in two ways: the average molecule length was determined using an Agilent 2100 Bioanalyzer (Agilent Technologies, CA, USA), and the library was quantified by real-time quantitative PCR (TaqMan Probe). Quantified libraries that passed the quality control were first amplified within the flow cell on the cBot instrument for cluster generation (HiSeq 4000 PE Cluster Kit, Illumina). Subsequently, the clustered flow cell was loaded onto the HiSeq 4000 Sequencer for paired-end sequencing (HiSeq^®^ 4000 SBS Kit, Illumina) with a recommended read length of 100 bp.

#### Gene expression

Transcript reads were mapped to the reference genome with Bowtie (version 2.2.5)^70^. Gene expression levels were calculated with RSEM (v1.2.12)^71^.

### Resequencing and identification of polymorphisms

#### Collection of samples

We collected blood samples from 83 domestic donkeys and two Asian wild asses encompassing four continents (Africa, Europe, Oceania, and Asia) (Supplementary Table 19). In addition, we obtained resequencing data for three Asian wild asses (accession numbers: SRR1562345, ERR650932–ERR650969, and ERR654542–ERR654612), one Somali wild ass (accession numbers: ERR650540–ERR650547, and ERR650570–ERR650703) and one domestic donkey (accession number: SRA082086) reported in previous studies^10,13,14^ and for 42 domestic donkeys and one kiang donkey accessible from the National Genomics Data Center (https://bigd.big.ac.cn/bioproject/browse/PRJCA001131). In total, genome sequences from 133 individuals were available to carry out population genomics analyses.

#### Sequencing, mapping, and variant calling

Genomic DNA was extracted from fresh blood and sequenced on the HiSeq 4000 platform. We cleaned the Illumina NGS raw data to remove adaptors, trim low-quality bases and remove “N” sites with Trimmomatic (V0.36)^72^. Subsequently, clean reads were mapped to our donkey genome using BWA (version: 0.7.10-r789)^47^. High-quality mapped reads (mapped, nonduplicated reads with mapping quality ≥ 20) were selected with SAMTools (version 1.3.1) and the following commands: “-view -F 4 -q 20” and “rmdup”^73^. For all 133 samples, mapping statistics based on high-quality mapped reads of each accession included (I) the coverage depth of each chromosomal position (SAMTools command “-depth”) and (II) the proportion of the donkey genome covered by different read depths. For autosomal and X chromosomes, the region covered by at least two reads in ≥80% of all donkey accessions was defined as the “effectively covered region” of the donkey genome.

Only high-quality mapped reads were used for variant calling with GATK (version: 3.3-0-g37228af)^74^. BAM files were sorted and marked as PCR duplications with Picard (version: 1.117, http://broadinstitute.github.io/picard/). There is no well-annotated SNP and short-indel database for donkeys, so it was not feasible to use the “Base Quality Score Recalibrator” (BQSR) and “IndelRealigner” options of GATK. To carry out variant calling, we used the command “HaplotypeCaller”, which calls SNPs and indels simultaneously via local de novo assembly of haplotypes in an active region. Applying the “hard filtering” method, we obtained an initial set of high-confidence SNPs and indels. The parameters of “hard filtering” were set by default, i.e., for SNPs we used QD < 2.0, FS > 60.0, MQ < 40.0, and MQRankSum < −12.5, while for short indels, we considered QD < 2.0, FS > 200.0, and ReadPosRankSum < −20.0. Subsequently, the original BAM files were analyzed with the BQSR and Indel Realigner options of GATK by using the set of high-confidence SNPs and indels. This dataset included 133 accessions (six Asian wild asses, one Somali wild ass, and 126 domestic donkeys) and it was considered the raw confidence variant set.

To obtain high-quality variant sets, the raw confidence variants were filtered based on well-established criteria. For autosomal and X chromosomes, (i) only variants present within the “effective covered region” were kept; (ii) only biallelic variants were taken into consideration; (iii) genotype calls were deemed successful if the read depth was ≥2 and ≤80 (otherwise they were classified as missing); (iv) positions with more than 80% heterozygous calls or more than 20% missing genotype calls were discarded; and (v) both alleles of each variant were required to be present in the homozygous state in at least one individual. In contrast, the NRY variants of 68 male accessions were filtered according to the following criteria: (i) only variants in NRY were kept; (ii) only biallelic variants were considered; (iii) genotype calls were considered successful if the read depth was ≥2 and ≤50 (otherwise they were classified as missing); (iv) all of the genotype calls needed to be homozygous; and (v) positions with more than 20% missing genotype calls were discarded. After these filtering steps, the remaining variants segregated in our data set were considered high-confidence variants. The polymorphic variants of 126 domestic donkey accessions were directly extracted from the high-confidence data set. To assess SNP quality, 10 primers were designed with Primer-BLAST (https://www.ncbi.nlm.nih.gov/tools/primer-blast/index.cgi?LINK_LOC = BlastHome). Seven accessions were used for Sanger sequencing. The annotation of these variants using our donkey gene set as a reference was carried out with an in-house Perl script and ReSeqTools (https://github.com/BGI-shenzhen/Reseqtools).

### Population structure analysis

#### Autosomes

Pairwise clustering based on identity by state (IBS) was calculated with Plink (version 1.90)^75^ by using high-quality SNP data from 133 samples. Based on the pairwise IBS genetic distance matrix, we constructed the neighbor-joining phylogenetic tree with Fneighbor (http://bioinfo.nhri.org.tw/cgi-bin/emboss/fneighbor). The tree was rooted on Asian wild asses and visualized with iTOL (http://itol.embl.de/)^76^. The PCA of 133 samples was performed with GCTA (v1.92.4)^77^. Ancestry and population structure were analyzed with ADMIXTURE (v1.23)^16^. An LD pruning step was performed with Plink (v1.90)^75^ with the following parameters: “--indep-pairwise 50 10 0.1”. Fifteen replicate runs, from *K* = 1 to *K* = 10, were performed with a random seed (1–999,999,999). The cross-validation errors of 20 replicate runs were plotted with the Gnuplot “boxplot” (version 5.2, http://www.gnuplot.info/). The 20 replicate ADMIXTURE runs were combined with CLUMPP (v1.1.2)^78^ and plotted with Distruct (v1.1)^79^.

#### Sex chromosomes

SNPs located in the NRY portion of the Y chromosome were used. Male samples with more than 20% missing genotype calls were removed. The neighbor-joining phylogenetic tree was constructed with MEGA (version 6.06)^80^, rooted on Asian wild asses and visualized with iTOL (http://itol.embl.de/).

#### Comparing the genetic diversity of the autosomal and sex chromosomes

The single-site nucleotide diversity (*π*)^81^ was calculated for autosomal and X chromosomes (only females were taken into consideration) with VCFtools (v0.1.13)^82^. In contrast, for NRY, we used an in-house Perl script. For each chromosome, the sum of the per-site *π* divided by the value of the “effective covered region” yielded the average chromosomal *π* (per-bp *π*). The *π* calculation was based on the SNPs of 126 domestic donkeys.

#### Analysis of the genetic diversity within each genetic group

Based on the population structure data (phylogenetic tree, PCA, and Admixture), all 126 domestic donkeys were divided into three groups: Tropical African donkeys, North African & Eurasian donkeys, and Australian donkeys. We took into consideration that the Australian sample was smaller than those representing the other two groups and, moreover, that it had a recent European origin, so the Australian group was not used in the diversity analysis. The *π* values of Tropical Africa and North Africa & Eurasia groups were inferred with the method explained previously. Watterson’s estimator (*θ*_w_) was calculated as defined in ref. ^83^. The magnitude of the LD was estimated with PopLDdecay software (v3.40)^84^.

#### Calculation of the *D*-statistic

To study the genetic relationship between SOM and domestic donkeys, we computed *D*-statistics^85^. First, we selected non-admixed samples (Admixture analysis) from Kenya (which is close to the geographic distribution of SOM), Iran, and Australia. The *D*-statistic calculation was performed with ADMIXtools (v5.1)^86^. The SNP matrix was converted to EIGENSOFT format by using the fcGENE and CONVERTF bioinformatic tools^86^. The *D*-statistics were calculated in the form of (((Population 1, Population 2), African wild ass), Asian wild ass) where Population 1 (P1) and Population 2 (P2) were represented by donkeys from African or Eurasian countries.

### Demographic history of donkeys and asses

The demographic history of the Asian wild asses, Somali wild ass, and domestic donkeys was inferred by using the PSMC model^19^. In the case of domestic donkeys, one sample per country (those with the maximum coverage fold) was selected for PSMC analysis. The parameters were set as follows: -N30 -t15 -r5 -p “4 + 25*2 + 4 + 6”. The generation time was set to 8 years, and the neutral mutation rate *μ* was set to 7.242 × 10^−9^ mutations per generation and site in accordance with previous reports^11,87^. Bootstrapping was performed 100 times for each sample (Supplementary Data 1).

The sequential Markov coalescent implemented in SMC++ software (V1.13)^20^ was used to estimate the demographic history of donkeys belonging to the Tropical Africa and North Africa & Eurasia groups. For each group, 10 random samples were selected and 10 replicated runs were performed. The non-WGS effective covered regions were masked by using the parameter “vcf2smc -m”. A mutation rate of 7.242 × 10^−9^ per site per generation, and a constant generation time of 8 years were assumed^11,87^ to convert coalescence generations into a time-scale.

### Phylogenetic tree of the Y chromosome

We transformed the genotypes of the Y chromosome to NEXUS format to perform phylogenetic tree inference with BEAST 2 (v2.5.2)^88^. BEAST 2 software was run with default parameters and the Yule Process^89^ as tree prior.

### Potential admixture events in ancestral species of donkeys and asses

To estimate the admixture event time in the donkey population, we performed a PSMC analysis^19^ and captured the hump structure in the estimated population size, which indicates when the admixture event took place. We verified the method with 70 experiments of simulated data generated with ms software^90^. For each experiment, we assumed an 8-year generation time. The ancestral population split into two populations with equal sizes at 200 kya. The two resulting populations merged into a single population at an admixture time ranging from 5 to 100 kya. The parameters for ms command lines, real admixture time and estimated admixture time are shown in Supplementary Table 23.

### Selection scan for coat color

Photos and records^91^ were used to determine the coat color of 44 donkeys, which were divided into two groups, i.e., Dun (23 gray donkeys) and non-Dun (21 black donkeys). The selection analysis involved these two groups and included the calculation of three statistics: (i) cross population XP-EHH^92^; (ii) *F*_ST_^93^; (iii) ROD_Dun_non-Dun_ (1 − *π*_non-Dun_/*π*_Dun_). The windows were set to 20 kb with a 10 kb step. The *F*_ST_, Tajima’s *D*^94^, and *π* were calculated using VCFtools^82^. In contrast, ROD_Dun_non-Dun_ was computed using an in-house Perl script, and the XP-EHH metric was estimated with selscan^95^. The empirical thresholds of selective signal values for XP-EHH, *F*_ST_, and ROD were empirically set as 2.5, 0.3, and 0.8, respectively. The overlap of the data sets generated with the three methods indicated that the strongest signal was located on chromosome 8 (~42.6 Mb). Other signals above the thresholds indicated previously were considered as secondary candidate regions. In the two compared groups (Dun vs. non-Dun), we calculated *π* and Tajima’s *D* in the major selective sweep mapping to chromosome 8 (~ 42.6 Mb). The SNPs and the indels mapping to this region were compared between Dun and non-Dun donkeys, and we found only one indel CT/C− located at chr8:42,742,556 (CT was the genotype of the reference genome), which segregated perfectly with the non-Dun phenotype. The SNPs around the indel were phased with SHAPEIT (v2.r790)^96^ to investigate haplotype structure in Dun and non-Dun donkeys. Median-joining haplotype networks based on the phased SNPs (only SNPs with MAF ≥ 0.1 were considered) from the 5′ end of the *TBX3* gene (chr8:42,723,946) to the 20,000 bp downstream (chr8:42,743,946) were built with PopART (v1.7)^97^. The genomic segments located 1000 bp upstream and downstream of the CT/C− indel position (chr8:42,742,556) were mapped to the human genome with BLAST (https://blast.ncbi.nlm.nih.gov/Blast.cgi). Enhancers located within this region were identified with GeneCards (http://www.genecards.org).

### Histological characterization of the *Dun* phenotype in donkeys and elucidation of the molecular basis

#### Sample collection

We selected three healthy non-Dun donkeys that were ~18 months old (Supplementary Fig. 18) from the Black Donkey Research Institute, Shandong Province. We also selected three healthy Dun donkeys, which were also ~18 months old, from a farm located in the Inner Mongolia Autonomous Region. We collected 5 ml of blood from each of the six donkeys via venipuncture into tubes with anticoagulant, and samples were stored at 4 °C until DNA extraction. We collected croup skin samples from these six donkeys with a minimally invasive procedure (Supplementary Fig. 18), and each sample was divided into two parts: one part was stored in liquid nitrogen for RNA extraction, and the other part was fixed in 4% paraformaldehyde for histological sectioning. We also collected skin samples from the dorsal strip line of Dun donkeys and the corresponding anatomical region of non-Dun donkeys.

#### Genotyping of the 1 bp deletion in the six donkeys

DNA was extracted from blood samples with the RelaxGene Blood DNA System (TIANGEN, Beijing, China). The 1 bp deletion was genotyped by Sanger sequencing on an ABI 3730xl DNA Analyzer (Applied Biosystems, USA) according to the manufacturer’s instructions. The primer sequences used were as follows: forward primer 5′-TTAGGGTCCAGCTCTCTCCA-3′ and reverse primer 5′-AAAGATGCACCCTGCCCATA-3′.

#### Transcriptome sequencing of the six croup skin samples

RNA extraction and transcriptome sequencing were performed according to the approach described in the part of Transcriptome sequencing. Differentially expressed genes between Dun and non-Dun donkeys were identified with the DESeq2 package (v1.28.1) from Bioconductor^98^. Genes were identified as differentially expressed if their expression level differed by two-fold. Gene expression is represented in fragments per kilobase million (FPKM).

#### RT-qPCR for the TBX3 gene

One microgram of RNA from each of the six croup skin samples was used to synthesize first-strand cDNA with the Transcript First-Strand cDNA Synthesis SuperMix Kit (TransGen, Beijing, China). Quantitative RT-PCR was performed in a Roche LightCycler 480 II Real-Time PCR Detection System (Roche, Swiss) device by using the Takara SYBR Premix Ex Taq II Kit (TAKARA, Dalian, China). The β-actin locus was used as the reference gene, and relative expression was calculated with the 2^−ΔΔCT^ method^99^. The primer sequences used for qRT-PCR analysis of *TBX3* expression were as follows: forward primer 5′-GAGGCCAAAGAACTTTGGGAT-3′ and reverse primer 5′-GGCATTTCAGGATCTGCCTTA-3′.

#### Histology and immunofluorescence

Croup skin samples stored in 4% paraformaldehyde were washed in phosphate buffered saline (PBS), dehydrated with a gradient of alcohol solutions (50% → 70% → 80% → 95% → 100% → 100%), cleared with xylene and infiltrated with paraffin wax. Samples were embedded in paraffin and sectioned into 4-micrometer-thick tissue sections. We also collected hair from the croup skin samples of Dun and non-Dun donkeys and made transverse sections of the samples. Both the hair and croup skin sections were stained with hematoxylin and eosin (H&E). Immunohistochemistry and immunofluorescence were carried out with a rabbit antibody against TBX3 (Bioss, China, bs-10266R), diluted at 1:200, by following a protocol involving the removal of wax from sections, antigen retrieval with Tris–EDTA (pH 8.0) in a microwave oven, and quenching autofluorescence. Sections were incubated with 4′,6-diamidino-2-phenylindole (DAPI) to stain the nucleus and were imaged with a fluorescence microscope.

### Reporting summary

Further information on research design is available in the Nature Research Reporting Summary linked to this article.

## Supplementary information

Supplementary Information

Reporting Summary

Description of Additional Supplementary Files

Supplementary Data 1

Supplementary Data 2

Supplementary Data 3

Supplementary Data 4

Supplementary Data 5

Supplementary Data 6

Supplementary Data 7

Supplementary Data 8

Supplementary Data 9

Supplementary Data 10

Supplementary Data 11

Supplementary Data 12

## Data Availability

Data from whole-genome sequencing, resequencing and transcriptome sequencing have been deposited in the GenBank database under BioProject accession PRJNA431818. Sanger sequences of the *Dun*, *non-dun1*, and *non-dun2* alleles from horse can be accessed with accessions KT896508, KT896509 and KT896510, respectively. In addition, we obtained resequencing data for 3 Asian wild asses (accession numbers: SRR1562345, ERR650932-ERR654612, ERR669419-ERR669469), one Somali wild ass (accession numbers: ERR650540-ERR650547, and ERR650570-ERR650703), one domestic donkey (accession number: SRR873443-SRR873445), and for 42 domestic donkeys and one kiang donkey accessible from the National Genomics Data Center (https://bigd.big.ac.cn/bioproject/browse/PRJCA001131). All relevant data are available from the authors. Source data are provided with this paper.

## Source data

Source Data

## Code availability

Custom Perl scripts were deposited into Github (https://github.com/JINPENG-WANG/scripts-for-donkey-genomes-sequencing).
