# Author Correction: Donkey genomes provide new insights into domestication and selection for coat color

**DOI:** 10.1038/s41467-021-21014-9

**Published:** 2021-01-26

**Authors:** Changfa Wang, Haijing Li, Yu Guo, Jinming Huang, Yan Sun, Jiumeng Min, Jinpeng Wang, Xiaodong Fang, Zicheng Zhao, Shuai Wang, Yanlin Zhang, Qingfeng Liu, Qiang Jiang, Xiuge Wang, Yijun Guo, Chunhong Yang, Yinchao Wang, Fang Tian, Guilong Zhuang, Yanna Fan, Qican Gao, Yuhua Li, Zhihua Ju, Jianbin Li, Rongling Li, Minghai Hou, Guiwen Yang, Guiqin Liu, Wenqiang Liu, Jiao Guo, Shanshan Pan, Guangyi Fan, Wei Zhang, Ruitao Zhang, Jie Yu, Xinhao Zhang, Qi Yin, Chuanliang Ji, Yuanchun Jin, Guidong Yue, Mei Liu, Jiake Xu, Shimin Liu, Jordi Jordana, Antonia Noce, Marcel Amills, Dong Dong Wu, Shuaicheng Li, Xiangshan Zhou, Jifeng Zhong

**Affiliations:** 1 Dairy Cattle Research Center, Shandong Academy of Agricultural Sciences, Ji’nan, China; 2 Liaocheng Research Institute of Donkey High-Efficiency Breeding and Ecological Feeding, Liaocheng University, Liaocheng, China; 3 National Engineering Research Center for Gelatin-based Traditional Chinese Medicine, Dong-E-E-Jiao Co. Ltd, Liaocheng, China; 4 BGI-Shenzhen, Shenzhen, China; 5 Department of Computer Science, City University of Hong Kong, Hong Kong, China; 6 College of Life Science, Shandong Normal University, Ji’nan, China; 7 BGI-Qingdao, BGIShenzhen, Qingdao, China; 8 Jiangsu Key Laboratory for Molecular and Medical Biotechnology, Nanjing Normal University, Nanjing, China; 9 School of Biomedical Sciences, The University of Western Australia M508, Perth, WA Australia; 10 Department de Ciència Animal idels Aliments, Facultat de Veterinària, Universitat Autònoma de Barcelona, Bellaterra, Spain; 11 Department of Animal Genetics, Centre for Research in Agricultural Genomics (CRAG), CSIC-IRTA-UAB-UB, Campus de la Universitat Autònoma de Barcelona, Bellaterra, Spain; 12 Leibniz-Institute for Farm Animal Biology (FBN), Dummerstorf, 18196 Germany; 13 State Key Laboratory of Genetic Resources and Evolution, Kunming Institute of Zoology, Chinese Academy of Sciences, Kunming, China; 14 Center for Excellence in Animal Evolution and Genetics, Chinese Academy of Sciences, Kunming, China; 15 State Key Laboratory of Bioreactor Engineering, East China University of Science and Technology, Shanghai, China; 16 Institute of Animal Science, Jiangsu Academy of Agricultural Sciences, Nanjing, China

**Keywords:** Population genetics, Evolutionary biology, Next-generation sequencing, Sequence annotation

Correction to: *Nat. Commun.* 10.1038/s41467-020-19813-7, published online 8 December 2020.

The original version of this Article contained an error in the author contributions, which incorrectly omitted the following: ‘J.W. led the experiments to study the genetic mechanism underlying the emergence of the non-Dun coat colors of donkeys, including performing RNA-seq of croup skin samples of Dun and non-Dun donkeys, performing the immunohistochemistry and immunofluorescence assays against donkey TBX3 protein and bioinformatics analysis of the 1 bp deletion downstream the TBX3 gene. J.W. also contributed to the plotting of Figures’.

The original version of this Article also contained an error in the author affiliations. The affiliation of Antonia Noce with Leibniz-Institute for Farm Animal Biology (FBN), Dummerstorf 18196, Germany, was inadvertently omitted.

These have now been corrected in both the PDF and HTML versions of the Article.

