# Supplementary material for: Donkey genomes provide new insights into domestication and selection for coat color
Source: Nat Commun. 2020 Dec 8;11:6014. doi: 10.1038/s41467-020-19813-7 (PMC7723042; doi:10.1038/s41467-020-19813-7)
Supplement: Supplementary file 1 — Supplementary Information [file 41467_2020_19813_MOESM1_ESM.pdf]

Supplementary Information for

Donkey genomes provide new insights into domestication and selection for coat color

Wang et al.

Supplementary Note 1. Sample Information.

DZ donkey is one of the five largest-type native breeds, which was formed mainly at the northwest of Shandong Province (like Dezhou City and Binzhou City) of China. Its average withers' height is generally 140.2 ± 3.8 cm for males and 135.0 ± 4.8 cm for females. The DZ breed has two coat color patterns, *i.e.*, completely black or with white (abdomen and area around the nose and eyes) and black pigmentation. From the perspective of traditional Chinese medicine, donkey-hide glue (*Asini Corii Collas*) made from black donkey is regarded as the best quality glue. For genome sequencing, a 6-month-old male DZ black donkey (its father, mother, grandfather, and grandmother were all black) from the Black Donkey Research Institute (Dong'E County, Shandong Province, China) was selected. This individual was healthy and did not have any body injury or infection. Blood samples were collected from the jugular vein by venipuncture into 5 ml tubes with anticoagulant. Approximately 60 ml of blood was obtained from this animal on September 14, 2014, and stored at -80°C for DNA extraction.

Supplementary Note 2. Data processing for Illumina HiSeq 2000 data.

Filtering. To obtain high-quality data, we applied several criteria to filter the raw data: 1) Filter out reads in which the proportion of N exceeds 2% and the proportion of poly(A) exceeds 5% or 10%. 2) Filter out low-quality reads: short-insert library reads that have 40% bases with quality scores ≤ 7; large-insert library reads that have more than 30% or 40% bases with quality scores ≤ 7. 3) Filter out reads with adapter contamination: reads with more than 10 bp aligned to the adapter sequence (allowing several mismatches lower or equal to 3 bp) were filtered out. 4) Filter out short-insert-size reads in which read 1 and read 2 overlapped ≥ 10 bp, allowing 10% mismatch. Read 1 and read 2 are both ends of a given paired end read. When read 1+ read 2 + 30 > insert size, we did not execute the short insert-size filtering. 5) Filter out PCR duplicates. When read 1 and read 2 of two paired end reads are identical, these reads are considered duplicates.

Error correction. For deep sequencing, the correct K-mers appear multiple times in the reads set, while randomly sequenced error-containing K-mers have a low frequency. We used K-mer frequency information to correct the short-insert size (170 bp, 250 bp, 500 bp, 800 bp) library data, and the K value was set to 17. We built a hash table to store the frequency of all 17-mers. Then, for each read, we started from high-frequency regions and extended across both sides to infer potential erroneous sites of low-frequency (< 10) 17-mers. For each inferred erroneous site, we tested the impact of changing to any of the other three allele types, and these changes were

picked up as candidates if all 17-mers containing the allele had a frequency equal to or higher than 10. When we obtained no candidates that satisfied these criteria, we did not change the base of the erroneous site; otherwise, the allele was replaced by that with the highest 17-mer frequency. A dynamic programming algorithm was used to find the optimal solution with minimal changes. To increase speed, we used threaded parallelization to split read sets and handled them in parallel by sharing the same 17-mer hash table.

Supplementary Note 3. Estimate the Genome Size with K-mer.

A K-mer refers to a sequence with k base pairs. We can obtain K-mers from the short-insert-size (insert size < 1 kb) reads with just one bp slide and calculate the frequency of each K-mer. The K-mer frequency follows a Poisson distribution when a certain amount of data is present. The genome size can be estimated with Genome Size equals K-mer number/peak_depth). We used 17-mer to estimate genome size. The K-mer number was 67,200,000,252, and the peak depth was 25. Genome size was estimated to be 2,688 Mbp (Fig. S1).

Supplementary Note 4. Construction of a high-quality reference genome.

One black male donkey from the Dezhou (DZ) breed was selected for genome sequencing. In the last two generations (at least), all ancestors of this individual belonged to the black DZ breed. We illustrated the pipeline of genome assembly in Fig. S2 and Fig. S3. First, four short-insert and five long-insert libraries were constructed and sequenced in an Illumina HiSeq 2000 platform, yielding 515.7 Gbp clean data with a sequence depth of 211.4 × (Supplementary Table 1). Sequencing data generated from short-insert libraries produced a draft assembly of 2,472,592 contigs, with a contig N50 of 5,346 bp. To link these short contigs, single molecule, real-time (SMRT) sequencing applied on a PacBio platform was utilized to generate 76.43 Gbp of data (Supplementary Table 1). The PacBio sequencing data improved the contig N50 to 7.23 Mb and decreased the contig number to 2,375. Joining of data from long-insert libraries again improved the contig N50 to 7.92 Mb, generated a scaffold N50 of 34.1 Mb, and decreased the scaffold number to 1,430 (Supplementary Table 2), which made the assembly more continuous than the previous donkey assembly published by Renaud et al. (*1*). With the aim to generate a chromosome-level donkey genome assembly, the Hi-C sequencing technology was utilized to anchor scaffolds belonging to the same chromosome. A total of 24.88 Gbp valid data were obtained after filtering (Supplementary Table 1), making it possible to assemble a draft reference genome into 43 super scaffolds with contig N50 to be 7.92 Mbp, scaffold N50 to be 93.37 Mbp, and 99.88% coverage of the donkey genome (Table 1, Supplementary Table 2). To anchor the scaffolds to their corresponding chromosomes, we mapped previously published chromosome

markers (*2, 3, 4*) to our super scaffolds and assembled donkey chromosomes by assuming collinearity between the chromosomes of horses and donkeys (Supplementary Data 1). To assemble the donkey Y chromosome, we also mapped the 20 donkey Y chromosome markers to our donkey reference genome by using BLAT (*5*), and these 20 markers were also mapped to four PacBio contigs (Supplementary Table 3). We also identified contigs belonging to Y chromosome using re-sequencing data (Methods and Fig. S10). Finally, 99.83% of scaffolds were anchored to 32 chromosomes (including the X and Y chromosomes), with only 565,467 additional bp not anchored to any chromosome and requiring further investigation (Supplementary Table 4). This novel and high-quality donkey genome assembly has been denominated as EquDZ1.0. Comparison of these results with those obtained by Huang et al. (*6*) and Renaud et al. (*1*) showed a 24-fold and 6-fold improvement in the scaffold N50, respectively.

Analysis of our sequencing results indicated that 99.78% of the reads with a short insert size could be successfully mapped to the assembled genome (Supplementary Table 5). Moreover, the reads covered 98.35% of the assembled genome (Supplementary Table 4). To verify the completeness of the sequences of the coding regions, we assembled unigenes with Trinity 2.15 based on transcriptome sequence data from thirteen tissues. Alignment results indicated that 99.78% of uni−genes were covered by the EquDZ1.0 reference genome (Supplementary Table 6). We further evaluated the quality and completeness of our genome assembly with the Benchmarking Universal Single-Copy Orthologs (BUSCO 2.16) data sets (*7*). Of the total 4,104 BUSCO ortholog groups (mammalia_odb9), 3,937 (96.0%) ortholog groups were searched in the donkey assembly genome, and 3,910 (95.3%) BUSCO genes were matched to the "complete single-copy" category, 27 (0.7%) were "complete duplicated," 96 (2.3%) were "fragmented," and 71 (1.7%) were "missing" (Supplementary Table 7).

We predicted that repeat sequences cover 41.79% of our genome assembly (Supplementary Table 8). We also calculated the proportions of different types of transposable elements (TEs) in the donkey genome (Supplementary Data 2, Fig. S4). With *de novo* prediction and homologous alignment tools, we predicted 21,983 protein-encoding genes in our assembly (Supplementary Tables 9-13). After analyzing their annotation status in the InterPro, GO, KEGG, SwissProt and TrEMBL databases, 19,927 genes were successfully annotated at the functional level, while 2,056 genes remained unannotated (Supplementary Table 11). We also evaluated the quality and completeness of gene sequences with BUSCO 3.0.2 data sets. Of the total 4,104 BUSCO orthologous groups (mammalia_odb9), 3,675 (89.5%) matched protein-encoding genes, 3,632 (88.5%) were present as a complete single-copy in our assembled genome, 43 (1.0%) had

complete duplicated copies, 280 (6.8%) were fragmented, and 149 (3.6%) were missing (Supplementary Table 12). We generated RNA-Seq data for 13 types of tissues (Fig. S5) from three donkeys and determined the gene expression levels of 20,769 (94.48%) protein-encoding genes (Supplementary Data 3). The circular visualization graph depicting the genomic distribution of TEs and protein-coding genes was built with Circos and is displayed in Fig. S6.

Supplementary Note 5. Resequencing of donkey populations

In this work, we also collected DNA samples from 83 donkeys with a broad geographic distribution covering Africa, Europe, Asia, and Australia (Fig. S8, Supplementary Data 4). We constructed and sequenced libraries with 500 bp or 300 bp insert sizes using Illumina HiSeq 2000 sequencing technology. After filtering out the low-quality reads, sequence alignment indicated an average of 93% sequence coverage and an average of 10.9-fold depth for each sample respectively to our donkey reference genome (Fig. S10, Supplementary Data 4). In addition, we also downloaded sequence data of four Asian wild asses, one Somali wild ass and 43 domesticated donkeys, making a total of 133 individual genomes that were subjected to downstream analyses. The SNP and indel calling algorithms exclusively utilized uniquely aligned reads, and strict filtering criteria were applied (Methods). In total, we detected 17.28 million SNPs and 1.5 million indels in the 133 individuals. However, only 7.0 million SNPs and 0.66 million indels were detected in the 128 donkeys (Supplementary Table 16). We validated the SNP calling accuracy of Illumina sequencing by Sanger sequencing of 9 randomly selected SNPs in 7 individuals (Supplementary Data 5). Approximately 35.2% of the SNPs and 35.4% of the indels resided in gene regions. About 0.94% and 0.30% of genic SNPs and indels were distributed in exons, respectively (Supplementary Table 17). The proportion of exonic SNPs and indels was much lower than that observed in intergenic regions, reflecting the role of purifying selection in limiting exonic variability. The counts of nonsynonymous and synonymous mutations were 36,010 and 33,417, respectively.

Supplementary Note 6. Candidate genes in genomic regions differentiating Dun and non-Dun donkeys.

We also investigated candidate genes in other genomic regions differentiating Dun and non-Dun phenotypes (Supplementary Table 23). Ten genes were identified, but none of them was related to pigmentation. We compared their expression level in croup skins between Dun and non-Dun donkeys, and found no significant differential mRNA expression. Gene expression profiles of

Dun and non-Dun horses also revealed no significant differential mRNA expression for those genes (Supplementary Table 23).

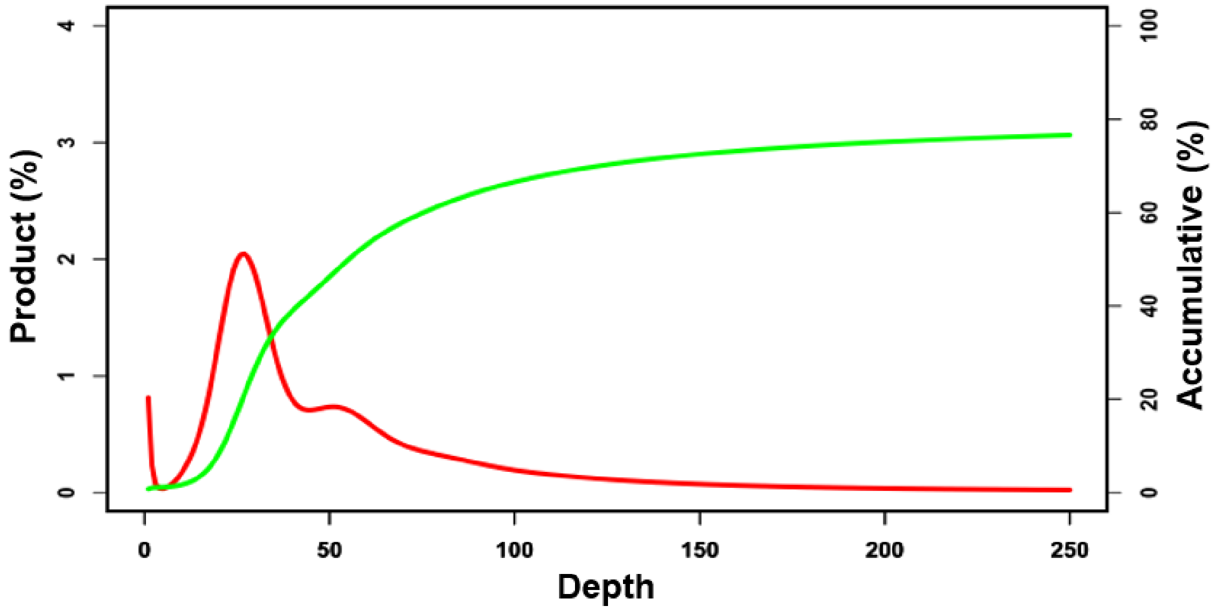

Supplementary Fig. 1. Statistics of 17-mer for calculating genome size. We used 17-mer to estimate the genome size (genome size equals K-mer number/peak depth), the K-mer number was 67,200,000,252, the peak depth was 25, and the genome size was estimated to be 2688.00 Mbp.

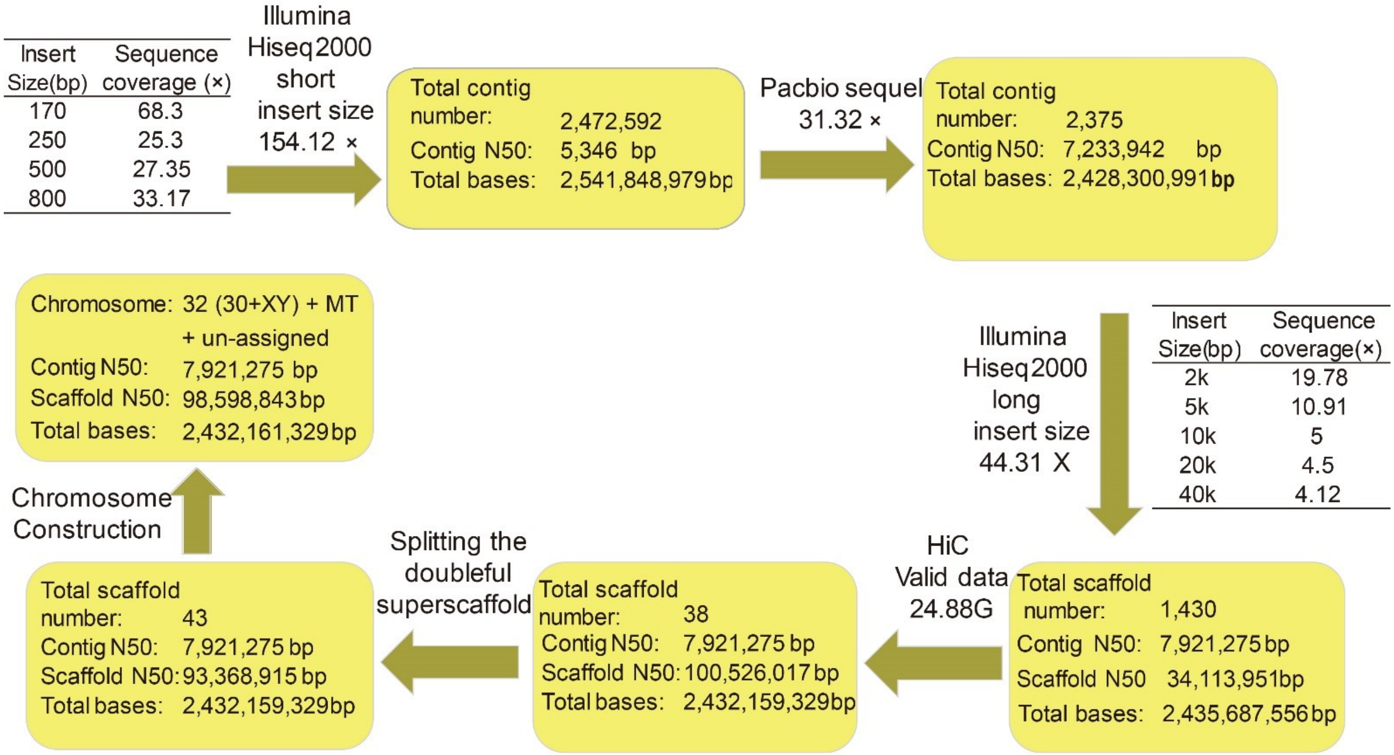

Supplementary Fig. 2. The pipeline of donkey genome assembly using Hiseq, PacBio and Hi-C sequencing technologies. The values in the yellow boxes were representative statistics obtained in each step of the genome assembly.

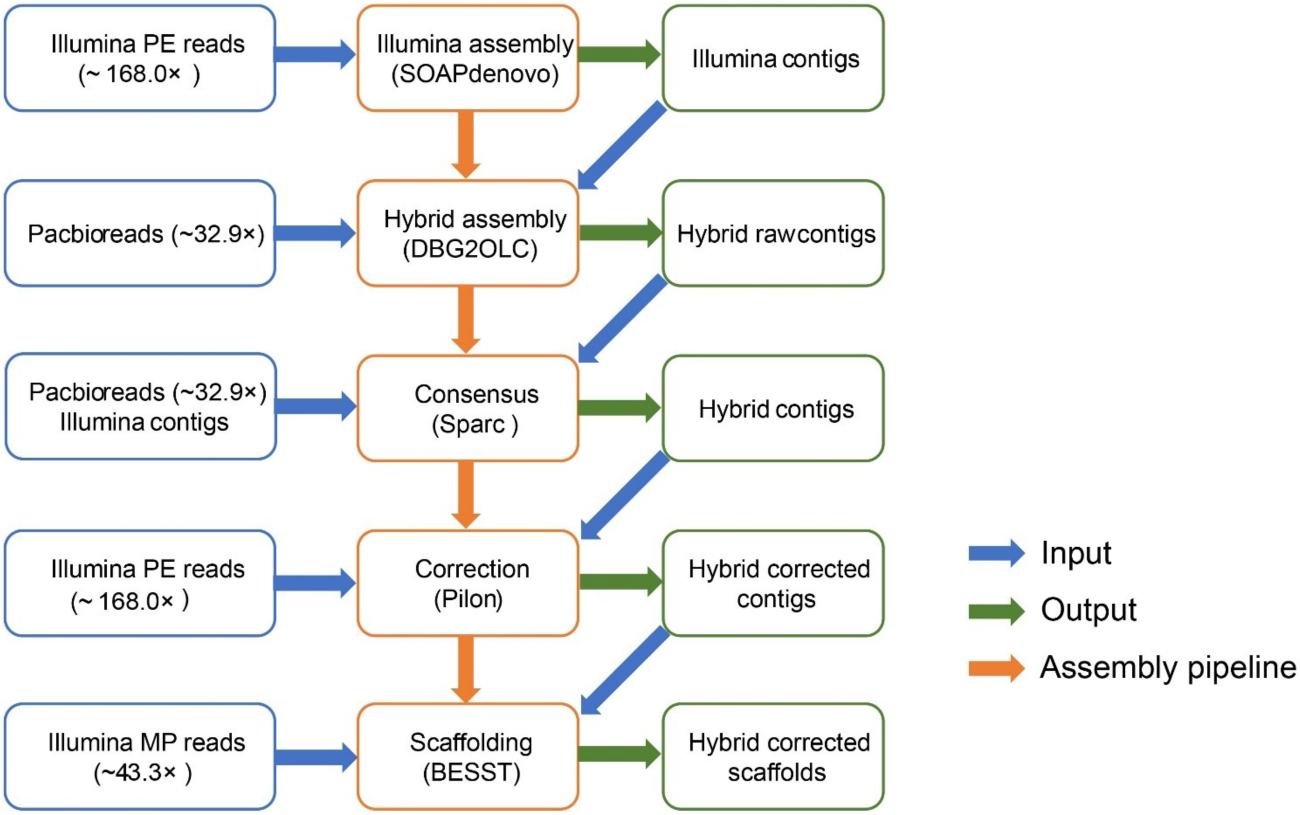

Supplementary Fig. 3. A schematic diagram of the pipeline used to assemble the donkey genome based on Illumina Hiseq 2000 and PacBio sequencing data.

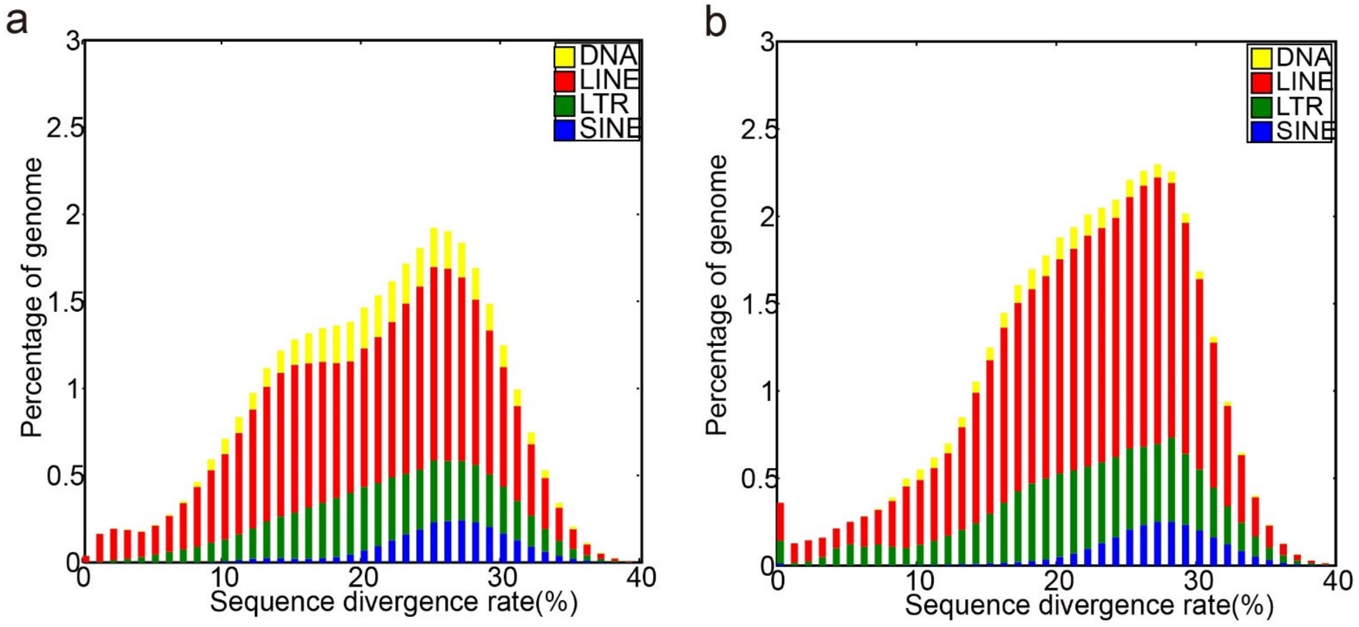

Supplementary Fig. 4. Distribution of the sequence divergence rate of each type of transposable element (TE). (a) The divergence rate was calculated across the TE elements identified in the donkey genome by using the homology-based method and the consensus sequence implemented in the RepBase17.01. (**b)** The divergence rate was calculated across the TE elements identified in the genome by using the *de novo* method and the consensus sequence implemented in the predicted TE library.

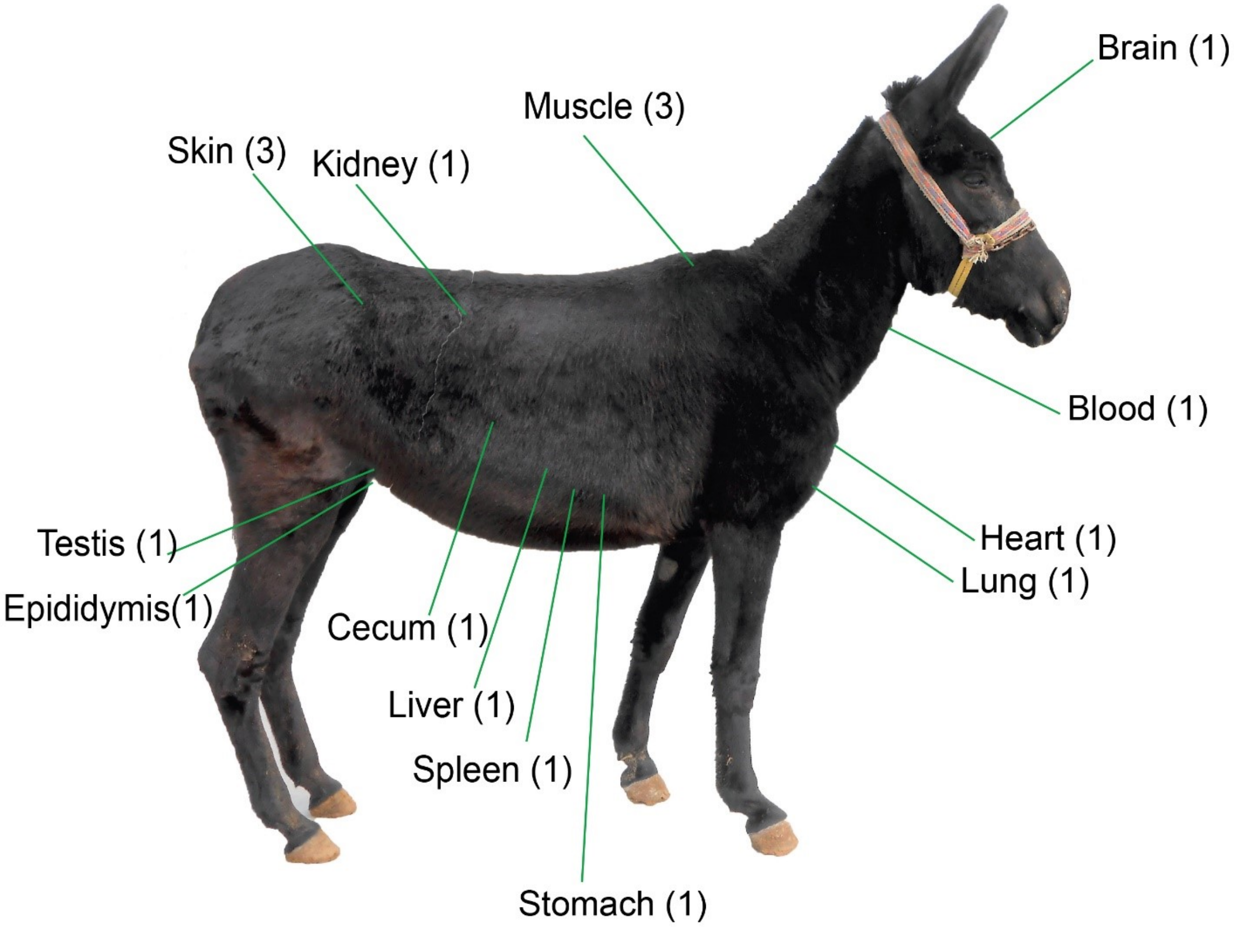

Supplementary Fig. 5. Diagram of the anatomic locations of tissues analyzed by transcriptome sequencing. Thirteen organs were sampled, and the numbers between parentheses indicate the number of samples for each specific organ. The photograph shown in this figure was taken by Haijing Li, one of the co-authors of the manuscript.

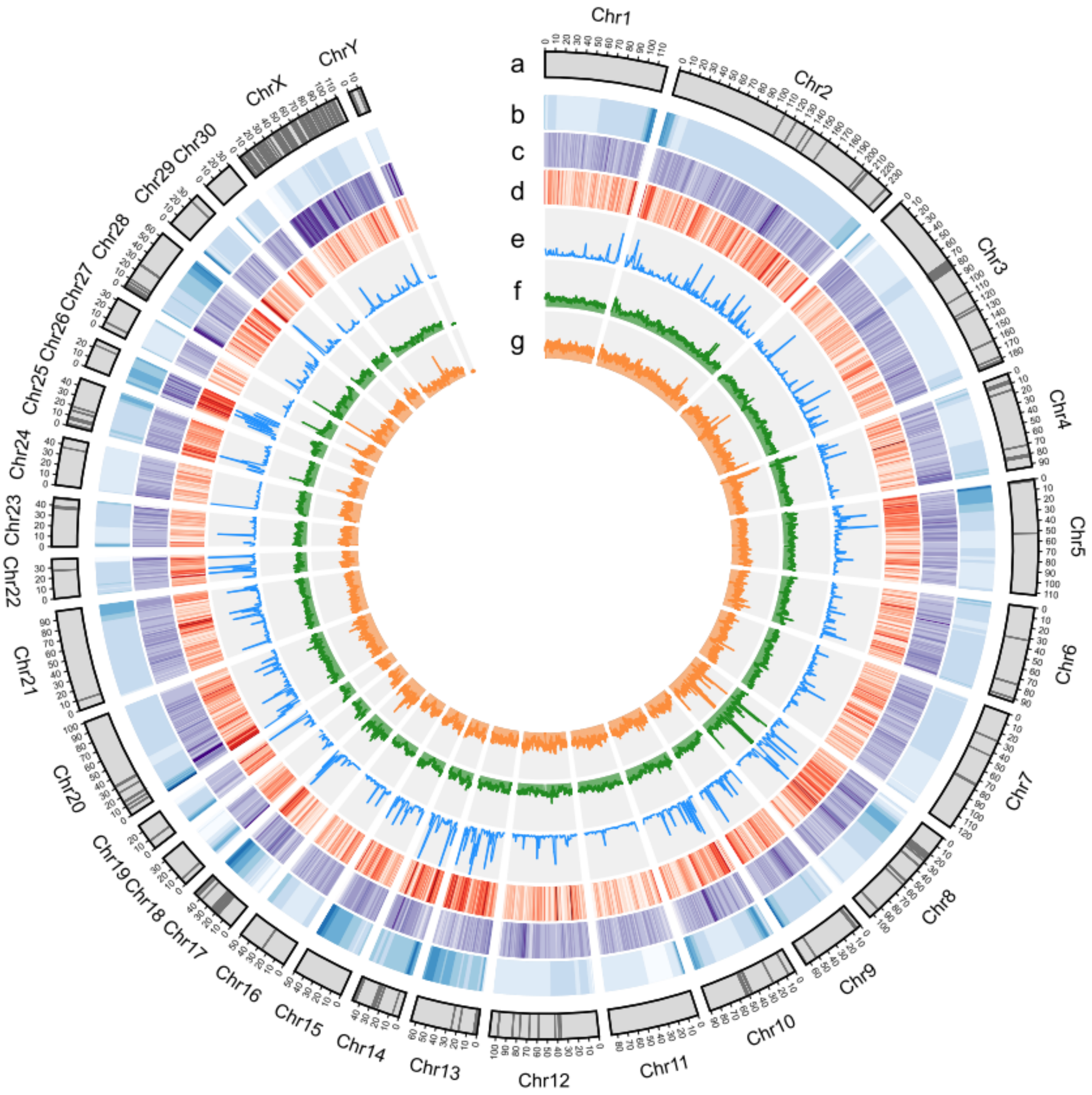

Supplementary Fig. 6. Circular visualization graph depicting distinct features of the genome of one Dezhou Donkey. Track **a** corresponds to 32 donkey chromosomes (30 autosomes and X, Y) measured in Megabases. The positions of the linkage scaffold are shown as vertical gray lines. Track **b** shows the GC% content. Track **c** indicates the density distribution of transposable elements. Track **d** displays the density distribution of protein coding genes. Track **e** shows the average expression levels of protein-coding genes. Track **f** indicates the density distribution of SNPs. Track **g** displays the density distribution of indels.

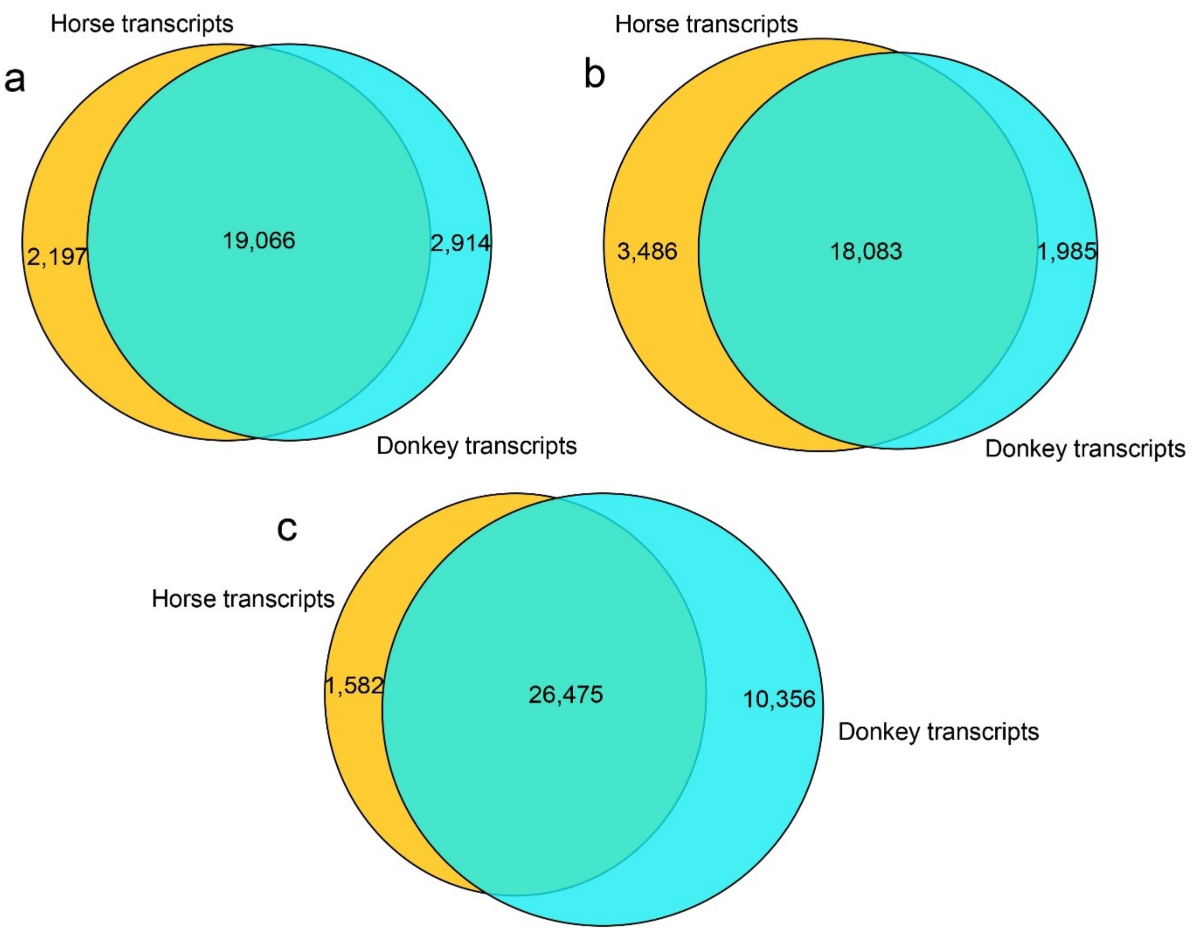

Supplementary Fig. 7. Venn diagrams of the protein-coding genes that were annotated in three different donkey genomes versus the protein-coding gene annotation for horses. (**a**) The sharing of mRNA transcripts between our assembly and horse. (**b**) The sharing of mRNA transcripts between the donkey assembly published by Renauld et al. (*1*) and horse. (**c**) The sharing of mRNA transcripts between the donkey assembly published by Huang et al. (*6*) and horse. The reference for the horse (*Equus caballus*) genome was EquCab2.0, and Ensembl Genes (version 86) were used. The circles with gold color refer to horse transcripts, while the circles with cyan color refer to transcripts of donkey. The comparison to the horse annotation was performed using a single transcript per predicted protein-coding gene.

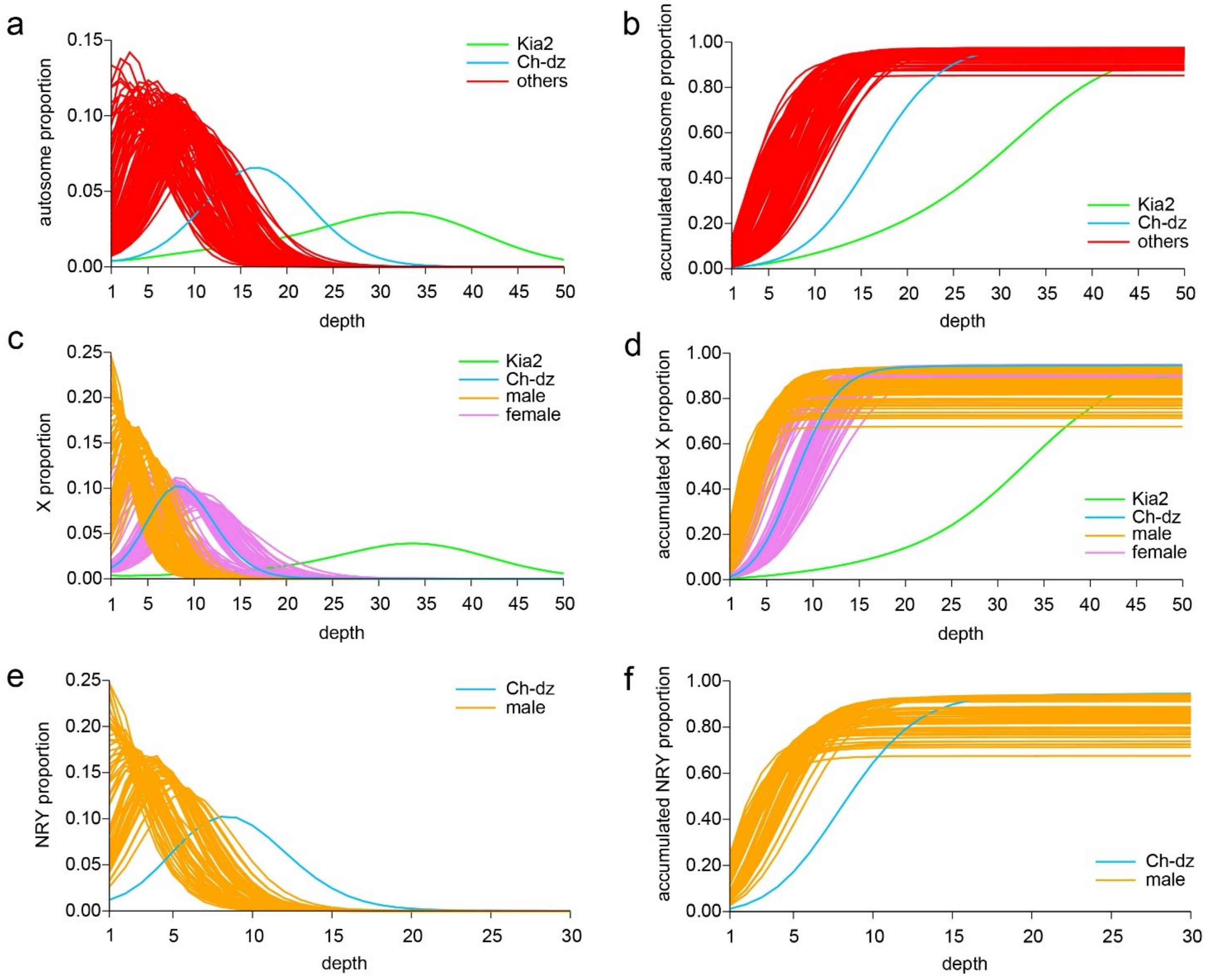

Supplementary Fig. 8. Proportion of donkey genome (a, c, e) and accumulated proportion of donkey genome (b, d, f) covered by different read depths of each accession. Only high-quality mapped reads (mapped, non-duplicated reads with mapping quality ≥ 20) were used for the statistics. (**a, b**) The autosome; (**c, d**) the X chromosome; (**e, f**) the non-recombining region of the Y chromosome (NRY). Kia2 (~ 44 ×) and Ch-dz (~ 19 ×) were samples with high sequencing depth. Kia refers to Kiang, Ch-dz refers to Dezhou donkey of China.

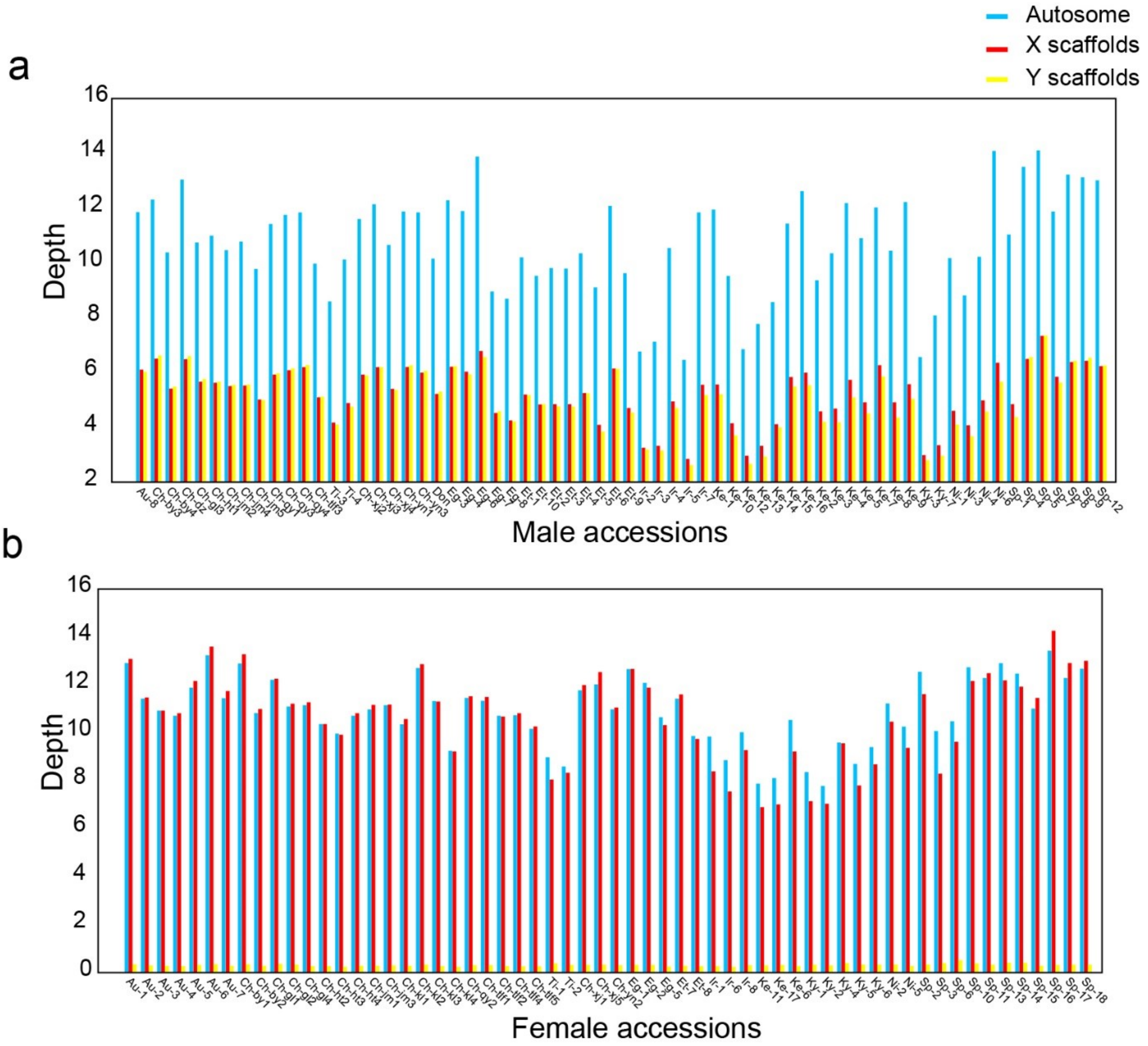

Supplementary Fig. 9. Plot of re-sequencing depth of male (a) and female (b) donkeys for autosomes as well as for the X and Y chromosomes. For male donkeys, sequencing depth of X and Y chromosomes were approximately half of that of autosomes. For female donkeys, sequencing depth of the X chromosome was almost the same obtained for autosomes, while the sequencing depth of the Y chromosome was almost zero.

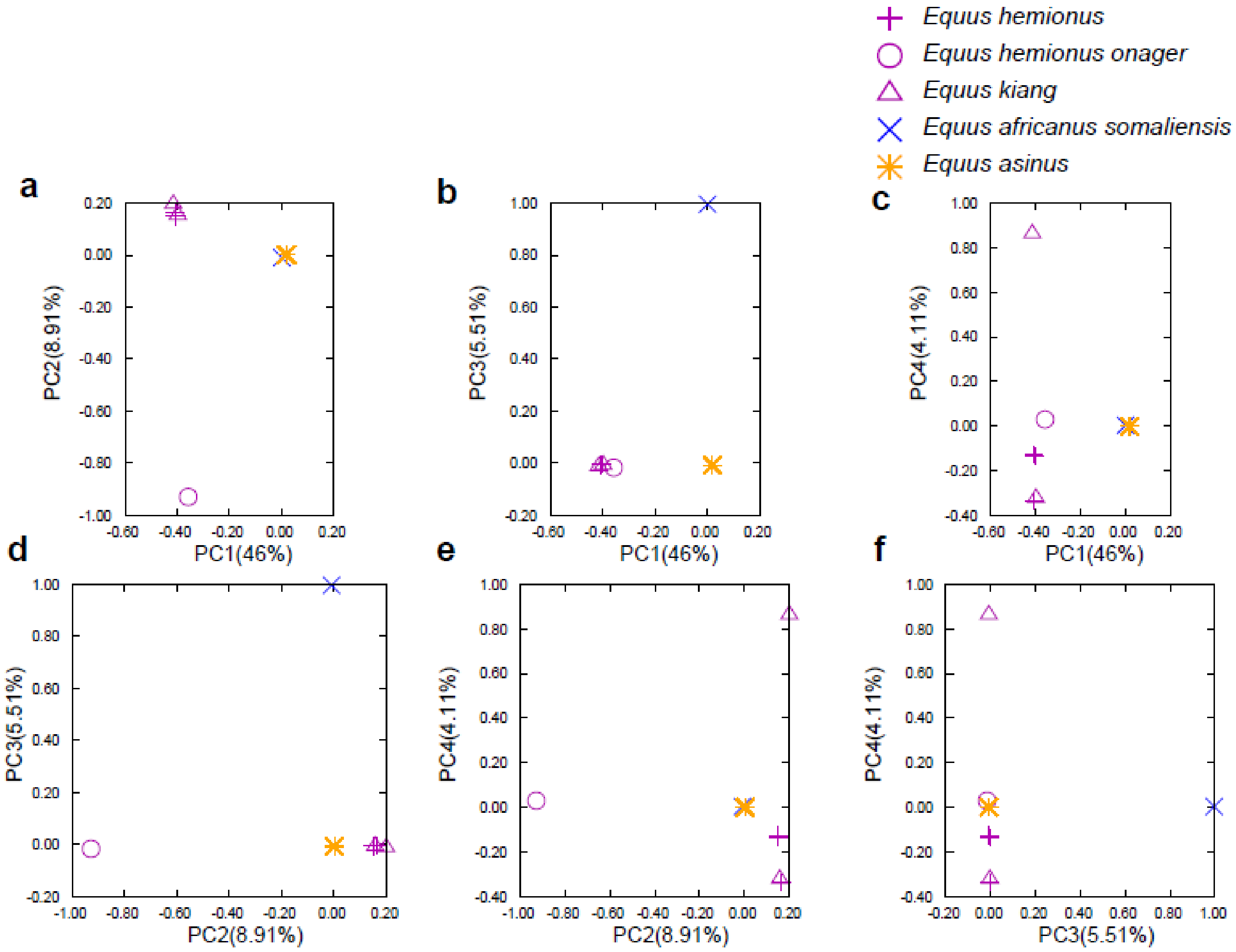

Supplementary Fig. 10. Principal component analysis (PCA) of wild asses and domestic donkeys from different countries based on 16,582,014 autosomal SNPs. Pictures were plotted with PC1 against PC2 (**a**), PC1 against PC3 (**b**), PC1 against PC4 (**c**), PC2 against PC3 (**d**), PC2 against PC4 (**e**), and PC3 against PC4 (**f**). Variances explained by principal components are presented in parentheses.

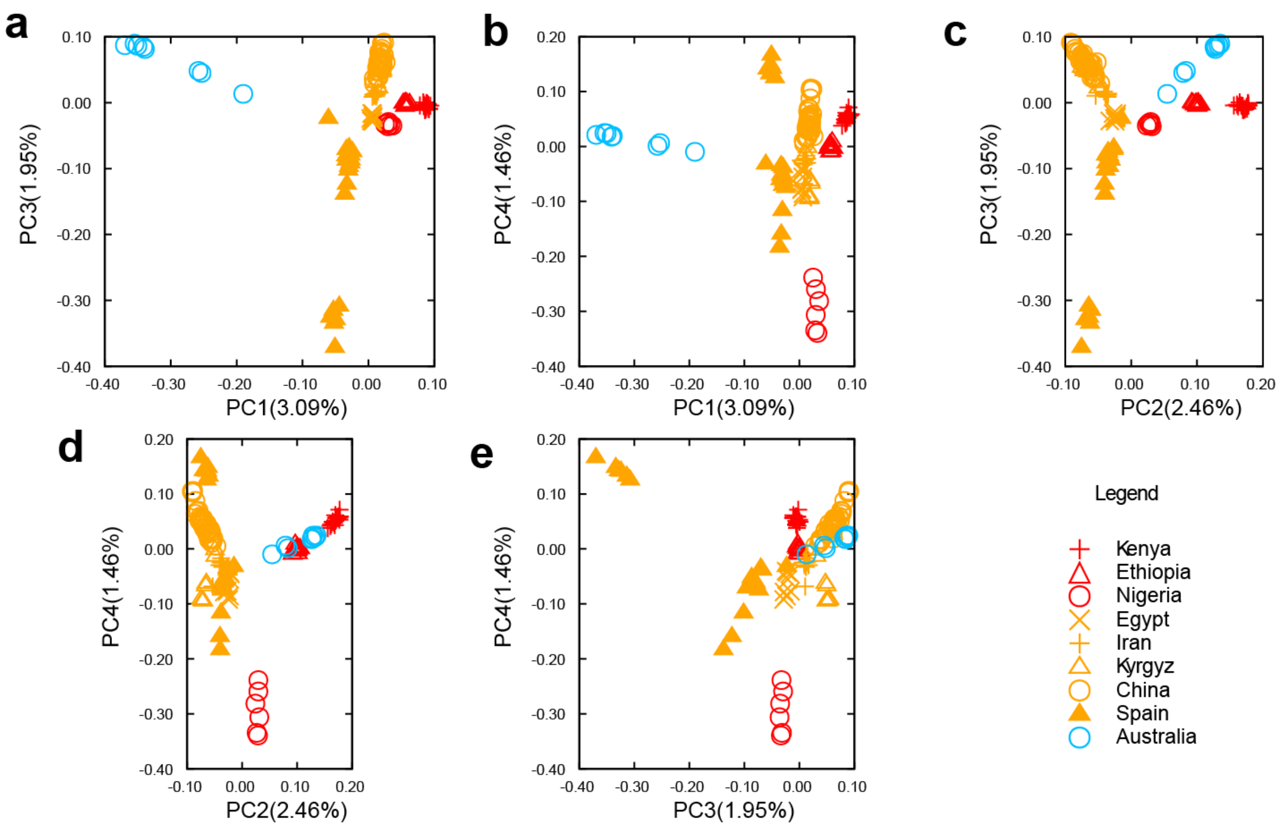

Supplementary Fig. 11. Principal component analysis (PCA) of domestic donkeys from different countries using 6,825,163 autosomal SNPs. Pictures were plotted by considering PC1 against PC3 (**a**), PC1 against PC4 (**b**), PC2 against PC3 (**c**), PC2 against PC4 (**d**), and PC3 against PC4 (**e**). Donkeys clustered mainly according to their geographical distribution. Variances explained by principal components are presented in parentheses.

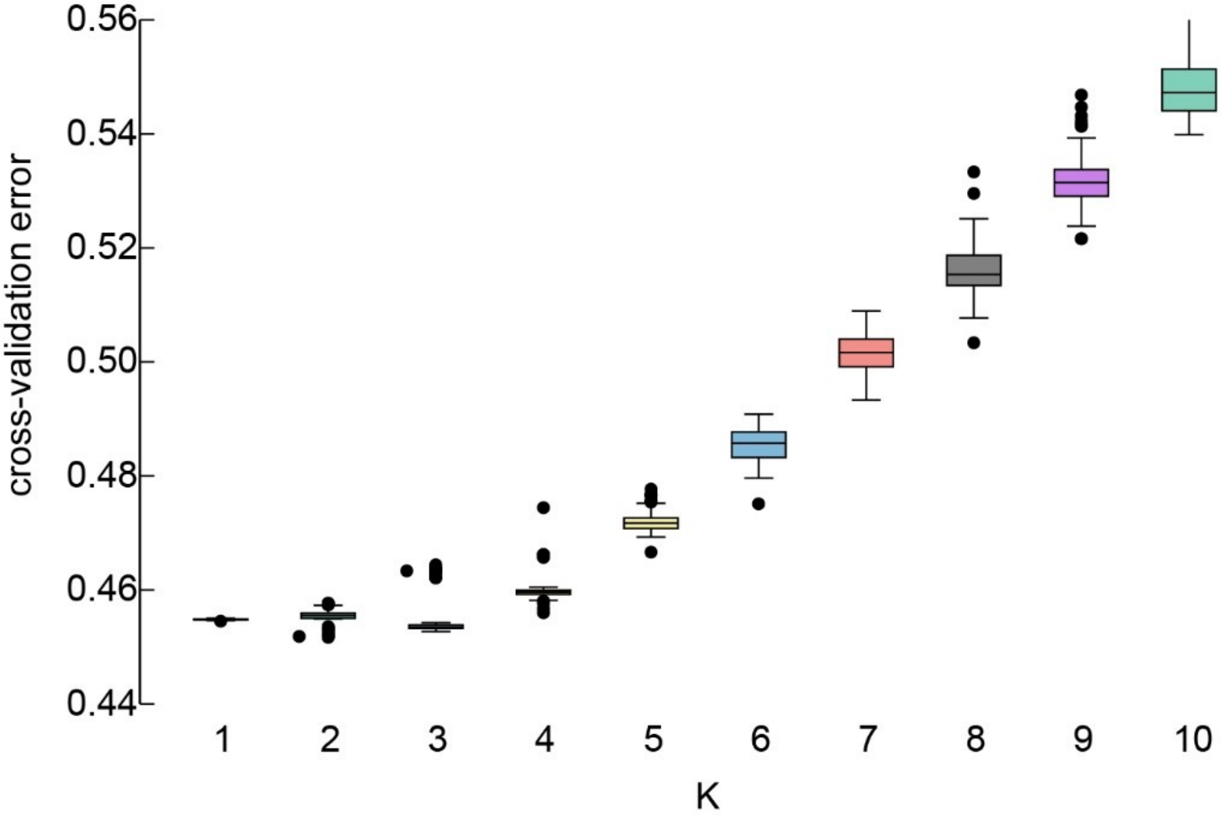

Supplementary Fig. 12. Plot of cross validation of different K values for ADMIXTURE analysis. Box plots indicate median (middle line), 25th, 75th percentile (box) and 5th and 95th percentile (whisker) as well as outliers (single points) with *n* = 20 replicate runs. Source data are provided as a Source Data file.

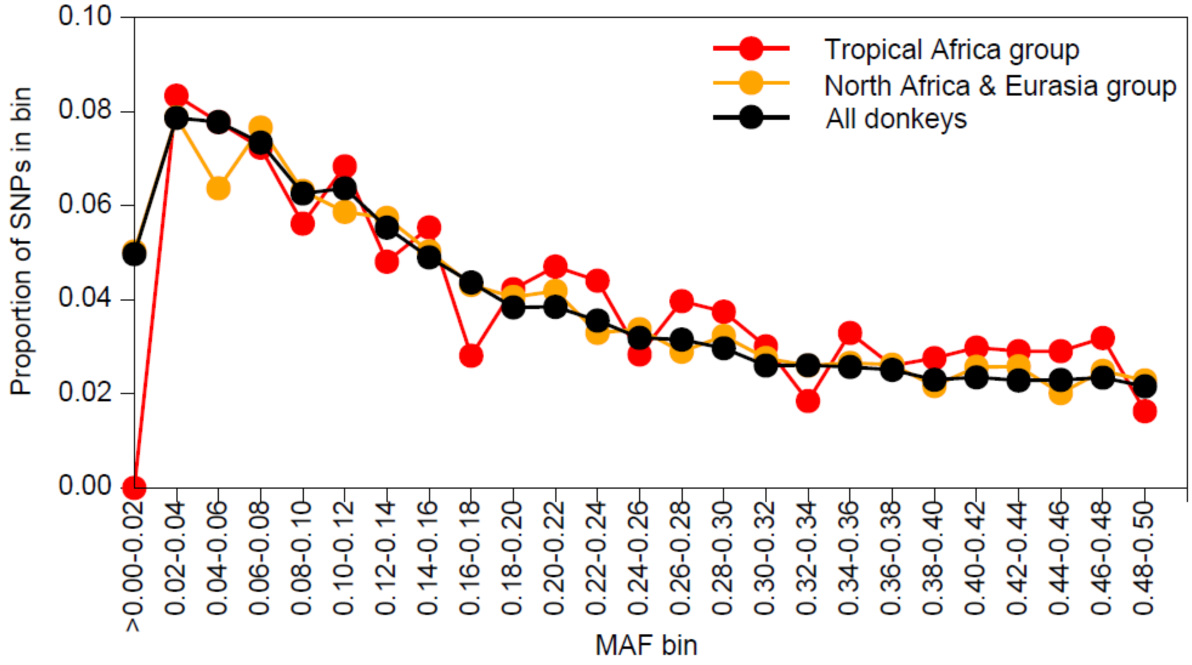

Supplementary Fig. 13. Minor allele frequency (MAF) distributions of SNPs in the two main donkey groups. Monomorphic SNP sites (MAF equals 0) in each donkey group were not taken into consideration.

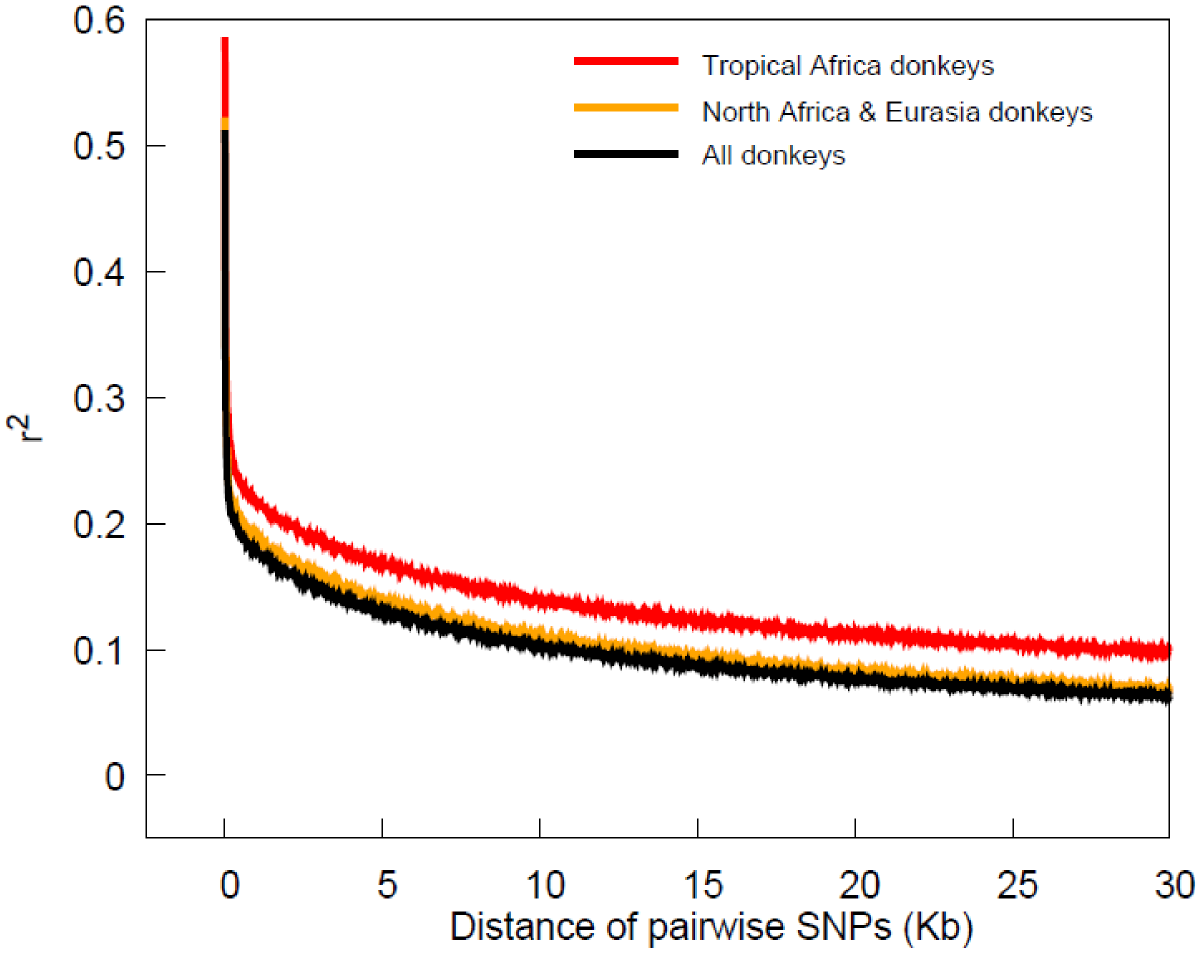

Supplementary Fig. 14. Linkage disequilibrium (LD) decay vs distance (Kb) in Tropical Africa donkeys and North Africa & Eurasia donkeys. The LD decays to half of its maximum within 0.5 Kb.

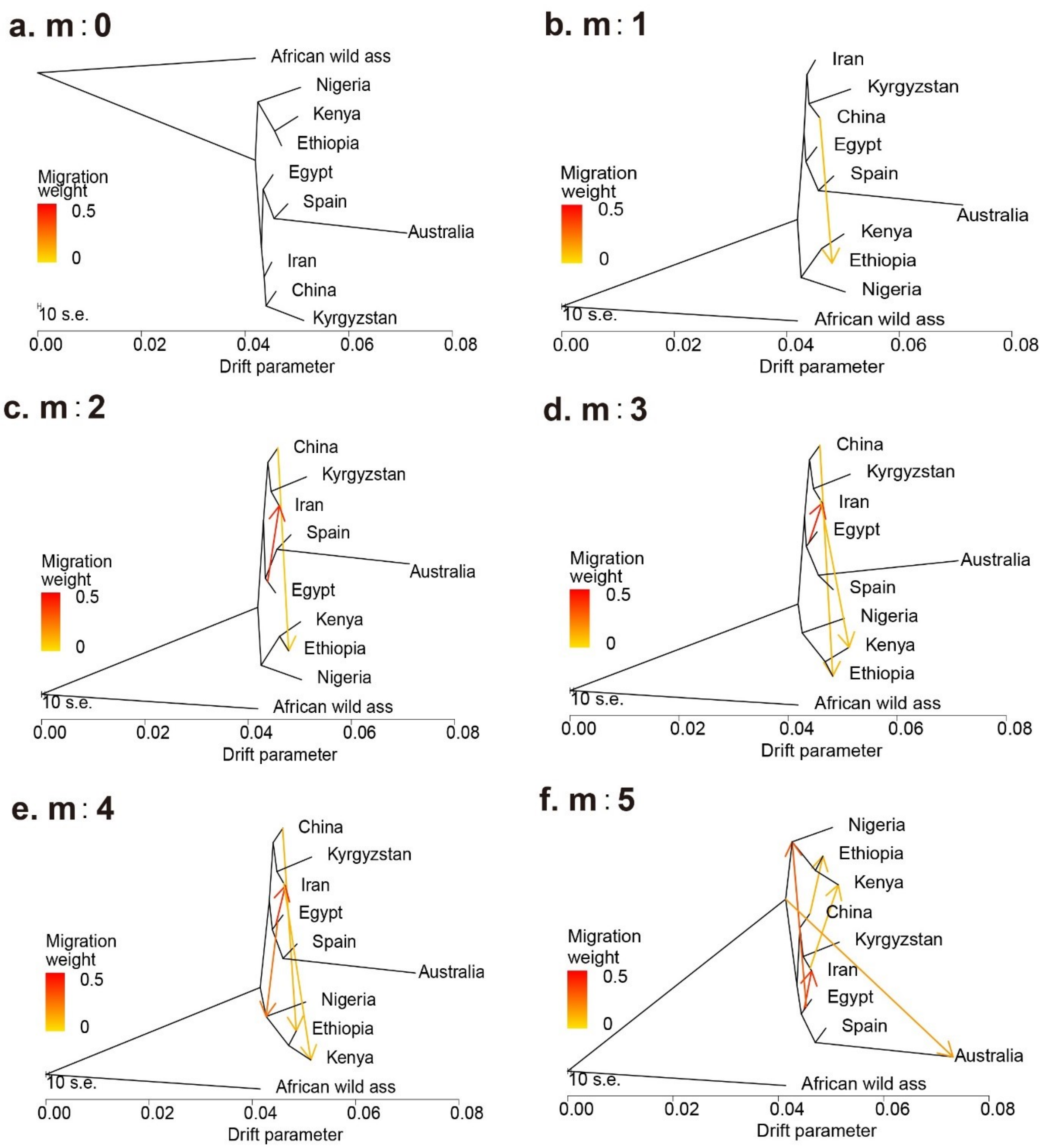

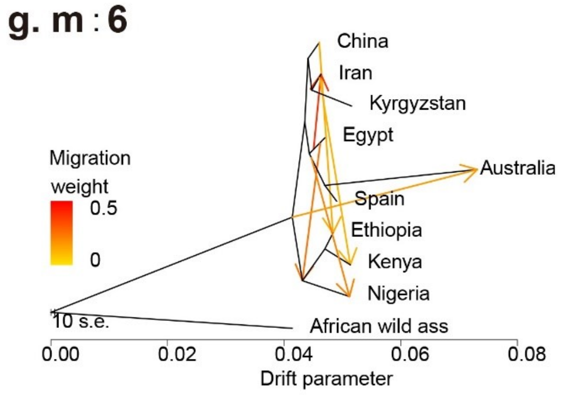

Supplementary Fig. 15. Gene flow between domestic donkey populations inferred with TreeMix (*8*). Up to 6 potential migration events (m) (from zero to six) are presented. The color of migration edges indicates the migration weight.

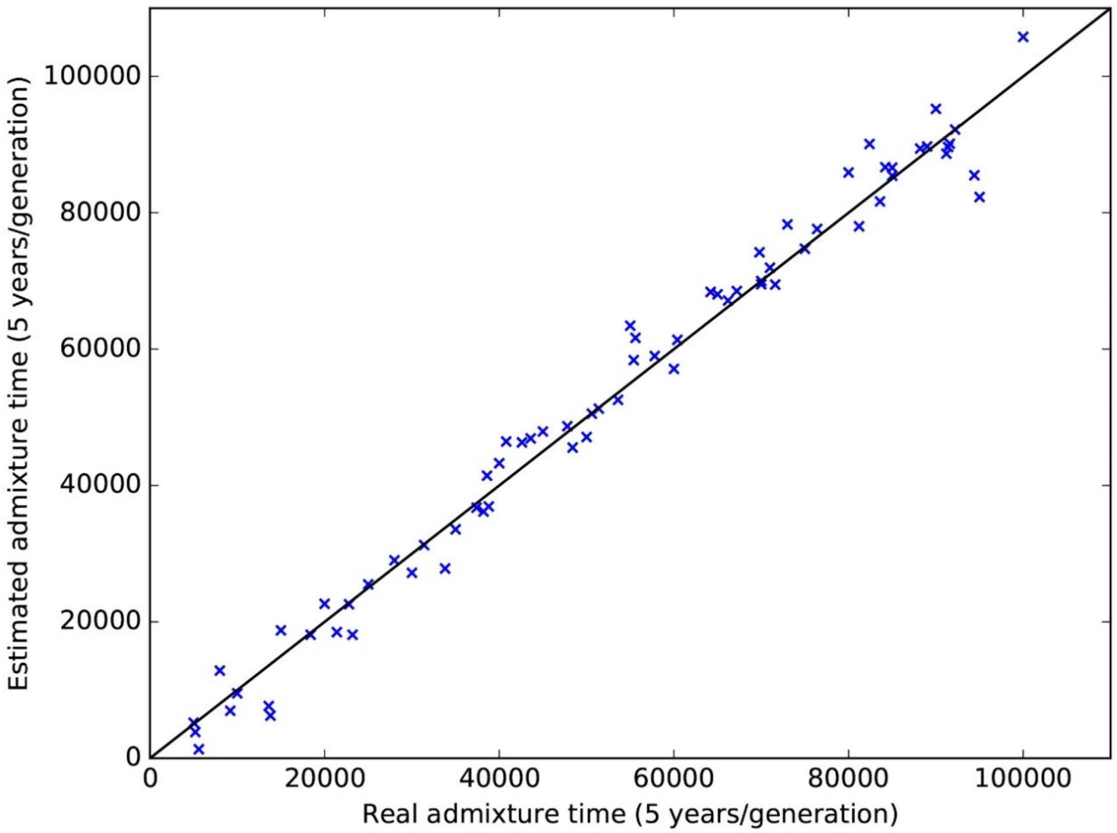

Supplementary Fig. 16. The estimated admixture time versus simulated admixture time. The simulated data was generated by ms (*9*) to simulate a population split into two equal-sized sub-populations that subsequently were admixed. The parameters of the simulation are shown in Supplementary Fig. 12. The x-axis is the simulated admixture time and the y-axis is the estimated admixture time.

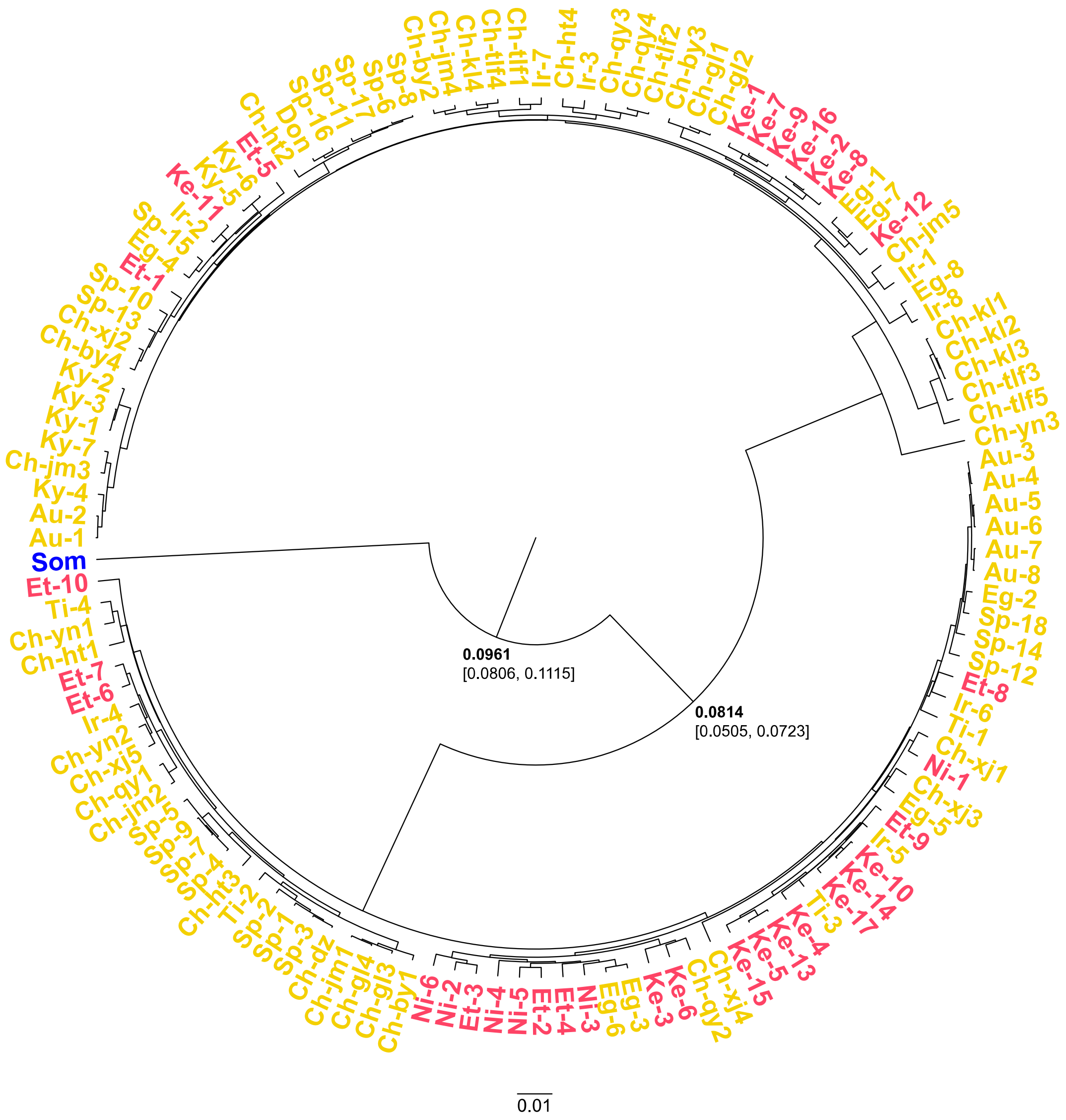

Supplementary Fig. 17. Phylogenetic tree based on SNPs mapping to the mitochondrial genome of Somali wild ass and domestic donkeys. Sample names in gold color represent North Africa & Eurasia donkeys, while sample names in red color represent Tropical Africa donkeys. The numbers aside the nodes are the estimated node heights and their 95% confidence intervals. A total of 953 mitochondrial SNPs were used to construct the tree. The BEAST 2 software (*10*) was used in the phylogenetic analysis.

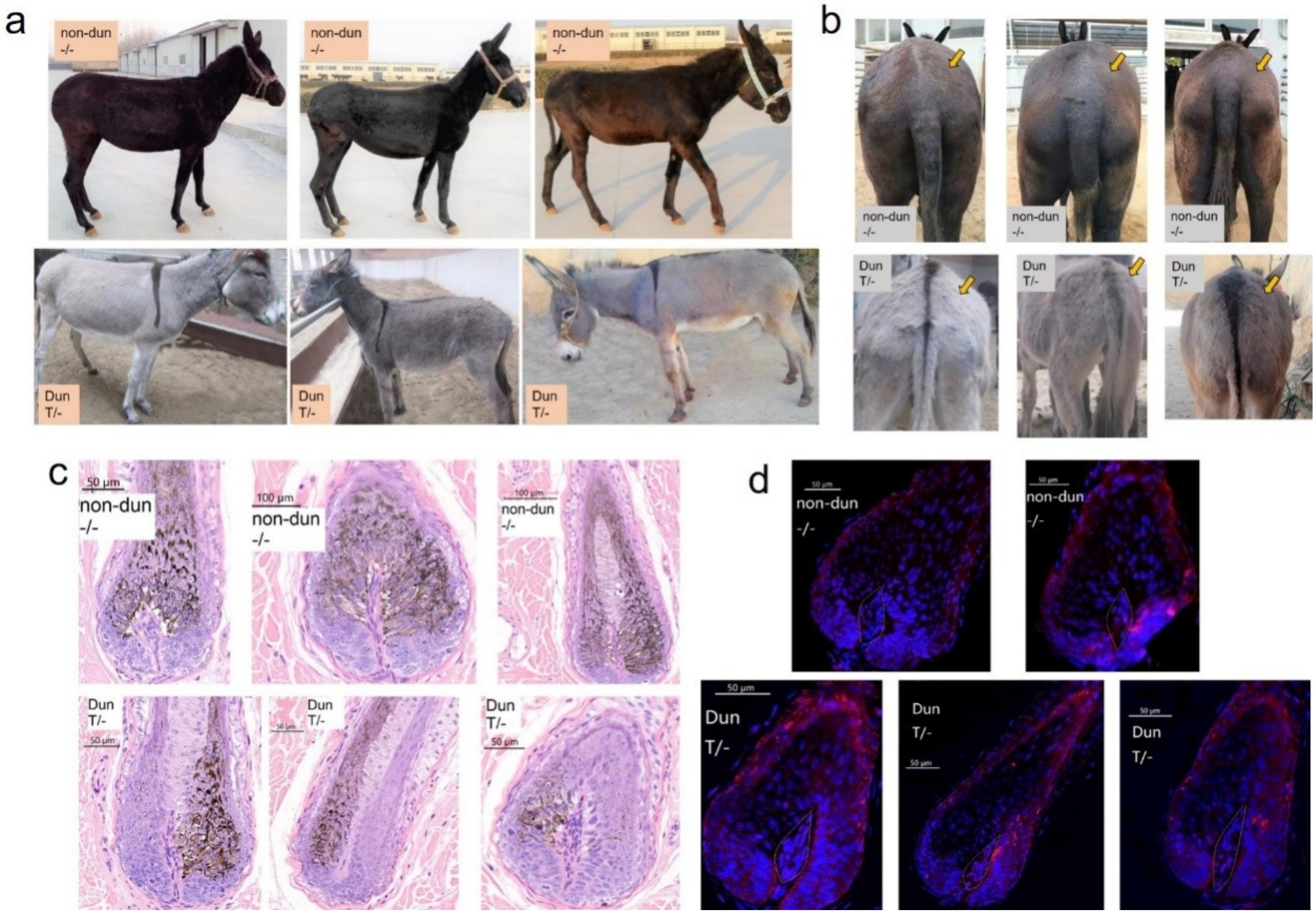

Supplementary Fig. 18. Phenotypic characterization and TBX3 protein expression in the croup skin of Dun and non-Dun donkeys. (a) Three non-Dun and three Dun donkeys were used to determine the expression of *TBX3* mRNA and protein in the croup skin. Sanger sequencing revealed that the genotype of the 1bp deletion detected by us in three non-Dun donkeys was "-/-". In contrast, Dun donkeys harbored the "T/-" genotype. (**b)** The croup skin samples were obtained from the location indicated with yellow arrows via minimally invasive sampling. (**c)** Micrographs of sections of hair follicles from non-Dun and Dun donkeys stained with hematoxylin and eosin, images representative of three experiments. Scale bars were defined in each image. (**d)** Micrographs of immunofluorescence for TBX3 (red) in sections of hair follicles from the croup skin of non-Dun and Dun donkeys. Scale bars were defined in each image. DAPI staining is depicted in blue, and white lines indicate the basement membrane, images representative of three experiments. The photos of non-Dun and Dun donkeys were taken by Haijing Li and Qiang Jiang, respectively, who are both coauthors of our paper.

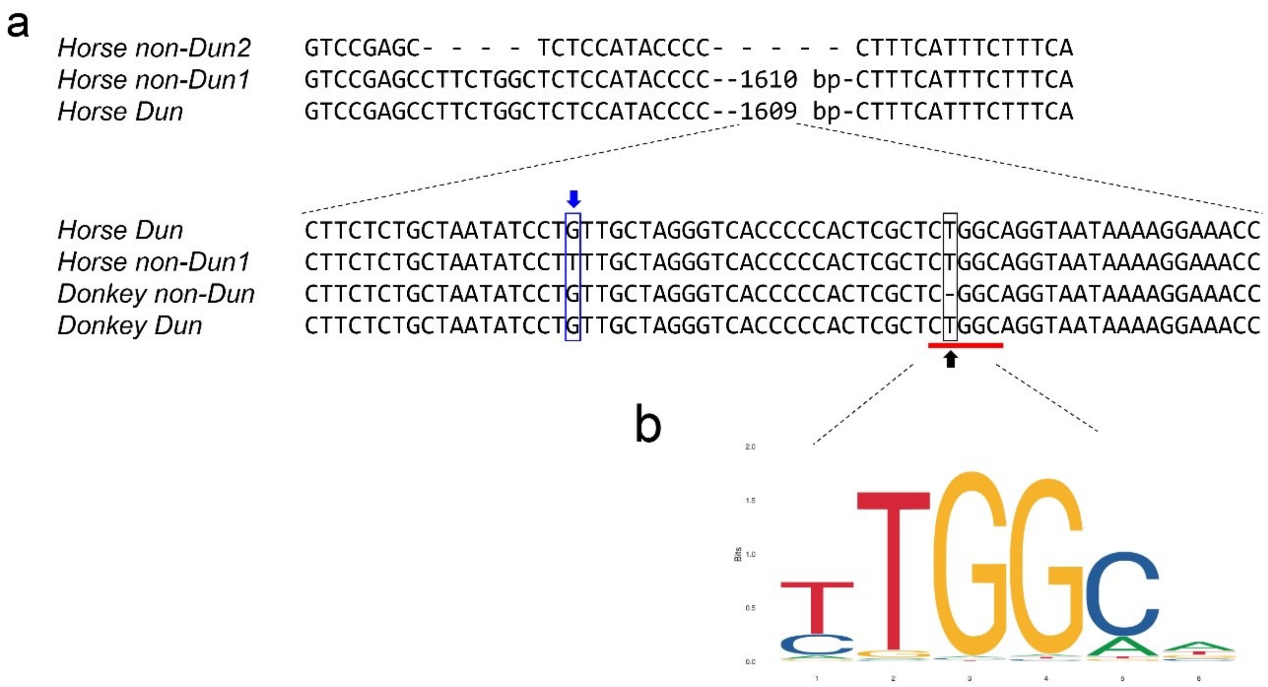

Supplementary Fig. 19. Positional comparison of the donkey *non-Dun* allele in relation to horse *Dun* and *non-Dun* alleles and depiction of the binding site for transcription factor NFI-C. (a) Comparison of horse *Dun* and *non-Dun* alleles and donkey *Dun* and *non-Dun* alleles. Compared to horse *Dun* allele, one deletion of 8 bp and another deletion of 1609 bp are present in the horse *non-Dun2* allele. The horse *non-Dun1* allele is in the deleted region of 1609 bp characteristic of the horse *Dun* allele. The blue arrow indicates the position of the horse *non-Dun1* allele, which involved a G>T substitution compared to horse *Dun* allele. Neither horse *non-Dun1* nor horse *non-Dun2* alleles were detected in the donkey genome. The black arrow indicates the position of the donkey *non-Dun* allele, which is also absent from the horse genome. The red line indicates the binding site sequence for the transcription factor NFI-C: CTGGC. **(b)** The sequence logo of matrix profile MA0161.1 for NFIC, which was obtained from JASPAR database (http://jaspar.genereg.net/matrix/MA0161.1/).

Supplementary Tables

Supplementary Table 1. Statistics of sequencing data corresponding to the *Equus asinus* genome and obtained with Illumina Hiseq 2000, PacBio Sequel and Hi-C sequencing technologies.

Sequencing Platform	Library name	Reads Length	Raw		After filtering	
			Data size (Gbp)	genome coverage (×)	Data size (Gbp)	genome coverage (×)
Illumina Hiseq 2000	insert size 170bp	paired 100 bp	193.3	74.35	177.59	68.3
	insert size 250bp	paired 150 bp	99.4	38.23	65.79	25.3
	insert size 500bp	paired 100 bp	75.09	28.88	71.1	27.35
	insert size 800bp	paired 100 bp	103.51	39.81	86.24	33.17
	insert size 2 kbp	paired 49 bp	82.21	31.62	51.43	19.78
	insert size 5 kbp	paired 49 bp	60.82	23.39	28.37	10.91
	insert size 10 kbp	paired 49 bp	53.79	20.69	12.74	5
	insert size 20 kbp	paired 49 bp	52.32	20.12	11.73	4.5
	insert size 40 kbp	paired 49 bp	38.77	14.91	10.71	4.12
	Sum	/	759.21	292	515.7	211.35
PacBio Sequel	cell 1	10,602	4.18	1.74		
	cell 2	11,099	5.21	2.17		
	cell 3	11,349	8.01	3.34		
	cell 4	12,408	8.11	3.38		
	cell 5	10,425	5.88	2.45		
	cell 6	11,511	7.08	2.95		
	cell 7	12,104	7.14	2.98		
	cell 8	11,828	7.3	3.04		
	cell 9	10,782	7.04	2.93		
	cell 10	10,026	6.8	2.83		
	cell 11	7,940	4.63	1.93		
	cell 12	8,945	5.05	2.1		
	Sum	/	76.43	31.84		
Hi-C	Lane 1	/	127.7	/	24.88	/

Supplementary Table 2. Statistics of the current donkey genome assembly in its primary version (using Hiseq 2000 and PacBio data) and in its final version (using Hiseq 2000, PacBio, and Hi-C data).

		Contig		Scaffold	
		Size (bp)	Number	Size (bp)	Number
Assembly based on Illumina Hiseq 2000 and PacBio data	N90	1,266,011	351	5,068,599	88
	N80	2,935,512	229	11,157,146	57
	N70	4,638,592	164	16,922,917	41
	N60	6,495,461	121	27,133,929	30
	N50	7,921,275	87	34,113,951	22
	Longest	31,532,978	/	119,294,037	/
	Genome Size	2,432,738,602	/	2,435,687,556	/
	Total Number (≥ 100 bp)	/	2,191	/	1,430
	Total Number (≥2 kbp)	/	2,190	/	1,429
Final assembly with Hiseq2000, PacBio and Hi-C data	N90	1,319,152	1,969	37,455,888	43
	N50	7,921,275	1,969	93,368,915	43
	longest	31,532,978	/	208,619,324	/
	Genome Size	2,428,624,875	/	2,432,159,329	/
	GC content (%)	41.76	/	41.76	/

Supplementary Table 3. Chromosome Y markers used to anchor super scaffolds to chromosomes. Information about these markers was obtained from Lindgren, et al (2004) (*11*).

Donkey Y chromosome specific sequences obtained from Lindgren et al. (2004)				The present donkey genome reference			
GeneBank accession number	length (bp)	start position	end position	PacBio Contig	chromosome	start position	end position
AY532878	452	1	452	Contig1025	Y	31,406	31,858
AH013682	4,183	1	4,183	Contig122	Y	1,268,482	1,273,598
AY532833	710	1	710	Contig122	Y	309,273	309,983
AY532834	539	1	539	Contig122	Y	435,166	435,705
AY532835	579	1	579	Contig122	Y	348,874	349,453
AY532836	323	1	323	Contig122	Y	419,072	419,395
AY532837	435	1	435	Contig122	Y	349,105	349,540
AY532838	341	1	341	Contig122	Y	415,507	415,848
AY532839	353	1	353	Contig122	Y	355,863	356,216
AY532840	381	1	381	Contig122	Y	366,422	366,803
AY532843	342	1	342	Contig122	Y	319,696	320,038
AY532844	426	1	426	Contig122	Y	319,299	319,725
AY532876	450	1	450	Contig122	Y	349,383	349,833
AH013675	563	1	563	Contig238	Y	3,359,614	3,360,096
AY532805	437	1	437	Contig238	Y	4,043,351	4,043,788
AY532807	468	1	468	Contig238	Y	3,252,443	3,252,911
AY532809	392	1	392	Contig238	Y	4,702,801	4,703,193
AY532810	215	1	215	Contig238	Y	4,702,228	4,702,443
AH013678	1,820	1	1,820	Contig454	Y	518,410	549,989
AY532808	445	1	445	Contig454	Y	633,581	634,026

Supplementary Table 4. The length and GC content of each chromosome in our donkey genome assembly, and the coverage of reads from small insert size libraries. MT refers to the mitochondrial genome.

Chromosome	Length (bp)	GC content	Covered Base (bp)	Coverage (%)	Mean Depth (×)
Chr01	119,294,037	39.69%	118,679,311	99.48	59.89
Chr02	238,844,183	41.94%	237,420,621	99.4	60.15
Chr03	183,772,800	39.23%	174,785,478	95.11	57.22
Chr04	92,923,151	39.57%	91,326,084	98.28	56.28
Chr05	112,293,617	43.11%	110,968,949	98.82	59.59
Chr06	93,368,415	41.30%	92,678,420	99.26	60.1
Chr07	123,523,216	41.26%	122,769,434	99.39	59.86
Chr08	104,246,046	42.65%	102,815,436	98.63	58.18
Chr09	64,683,936	41.89%	63,980,400	98.91	60.44
Chr10	90,667,400	42.11%	89,615,606	98.84	59.35
Chr11	85,788,095	37.72%	85,507,560	99.67	58.99
Chr12	106,346,232	40.41%	105,556,057	99.26	59.97
Chr13	64,922,896	45.26%	62,884,798	96.86	57.31
Chr14	47,677,399	46.61%	45,321,861	95.06	58.73
Chr15	50,236,165	45.26%	50,010,397	99.55	60.44
Chr16	50,732,815	39.59%	50,518,295	99.58	60.22
Chr17	47,651,721	41.49%	45,919,301	96.36	68.99
Chr18	33,166,870	41.39%	32,880,316	99.14	61.05
Chr19	26,998,044	43.60%	26,673,575	98.8	59.73
Chr20	100,526,017	42.67%	99,389,442	98.87	59.08
Chr21	98,598,843	41.87%	98,185,553	99.58	60.43
Chr22	38,360,130	42.04%	38,113,696	99.36	59.26
Chr23	47,379,281	40.71%	46,617,448	98.39	59.21
Chr24	46,610,899	38.32%	46,438,617	99.63	59.36
Chr25	47,158,557	42.53%	46,115,180	97.79	58.51
Chr26	28,693,288	47.43%	28,323,928	98.71	60.28
Chr27	32,167,772	39.07%	31,979,436	99.41	59.69
Chr28	63,897,334	43.75%	62,577,138	97.93	135.87
Chr29	37,455,388	42.13%	37,245,422	99.44	59.61
Chr30	30,283,642	40.32%	30,082,259	99.34	58.73
ChrX	110,613,827	39.32%	103,958,604	93.98	28.99
ChrY	12,677,250	39.14%	12,329,286	97.26	31.23
MT	36,596	42.06%	25,890	70.75	571.2
Un-assigned	565,467	44.30%	274,144	48.48	33.05
Total	2,432,161,329	41.76%	2,391,967,942	98.35	59.93

Supplementary Table 5. Evaluation of the genome assembly for *Equus asinus* using 166 Gb small insert-size reads (250bp, 500bp and 800bp).

Library	Reads Length (bp)	Total Reads	Mapped Reads	Mapped Reads Ratio (%)	Properly Paired Reads	Properly Paired Reads Ratio (%)
250 bp-lane1	150	236,184,965	235,750,940	99.82	217,647,594	92.15
250 bp-lane2	150	233,336,657	232,904,496	99.81	215,346,836	92.29
500 bp-lane1	100	326,710,835	325,930,772	99.76	309,814,306	94.83
500 bp-lane2	100	337,969,194	337,173,375	99.76	321,882,676	95.24
800 bp-lane1	100	295,372,535	294,682,315	99.77	278,540,896	94.30
Sum	/	1,429,574,186	1,426,441,898	99.78	1,343,232,308	93.76

Supplementary Table 6. Results of the evaluation of the *Equus asinus* genome assembly based on uni-genes.

Dataset	Number	Total length (bp)	Bases covered by assembly (%)	Sequences covered by assembly (%)	With > 90% sequence in one scafffold		With > 50% sequence in one scafffold	
					Number	Percent (%)	Number	Percent (100%)
All	103,431	157,508,690	98.80	99.73	99,722	96.41	103,032	99.61
> 200 bp	103,431	157,508,690	98.80	99.73	99,722	96.41	103,032	99.61
> 500 bp	58,780	144,082,075	98.78	99.87	56,207	95.62	58,618	99.72
> 1000 bp	42,558	132,571,066	98.75	99.93	40,495	95.15	42,464	99.78

Supplementary Table 7. Assessment of assembly completeness using BUSCO (run in mode of Genome) and comparison of the present donkey assembly with previous equine assemblies.

	Donkey assembly						Horse assembly	
BUSCO types	This study		Renaud et al. (2018)		Huang et al. (2015)		EquCab3.0	
	Number	ratio	Number	ratio	Number	ratio	Number	ratio
Complete BUSCOs (C)	3,937	96.00%	3,933	95.90%	3,953	96.30%	3,869	94.30%
Complete and single-copy BUSCOs (S)	3,910	95.30%	3,905	95.20%	3,920	95.50%	3,825	93.20%
Complete and duplicated BUSCOs (D)	27	0.70%	28	0.70%	33	0.80%	44	1.10%
Fragmented BUSCOs (F)	96	2.30%	104	2.50%	85	2.10%	150	3.70%
Missing BUSCOs (M)	71	1.70%	67	1.60%	66	1.60%	85	2.00%

Supplementary Table 8. General statistics of repeat sequences detected with several methods in our donkey genome assembly.

Methods used	Repeat size (bp)	Proportion of the genome (%)
TRF	20,004,628	0.82
RepeatMasker	840,976,871	34.53
RepeatProteinMask	263,007,299	10.8
De novo	855,173,102	35.11
Total	1,018,018,280	41.79

Supplementary Table 9. General statistics of predicted protein-coding genes. Average transcript length without considering the untranslated regions (UTR). Two approaches were employed in gene prediction: Homolog (*H. sapiens, M. musculus, E. caballus, S. scrofa, B. taurus, C. hircus*) and De novo (GENSCAN, AUGUSTUS), which can be consolidated using the program GLEAN. CDS refers to coding sequence.
*Information about homologs in the previous genome reference was obtained from Huang, J. et al (2015) (*6*).

	Methods used	Number	Average transcript length (bp)	Average CDS length (bp)	Average exon per gene	Average exon length (bp)	Average intron length (bp)
De novo	AUGUSTUS	22,709	50,143	1,449	8.82	164.28	6,226
	GENSCAN	24,786	40,324	1,739	10.37	167.64	4,117
Homolog	*Equus asinus**	20,033	26,509	1,563	8.74	178.77	3,221
	Mus musculus	17,160	26,627	1,596	8.56	186.38	3,309
	Equus caballus	18,885	25,281	1,500	8.58	174.63	3,134
	Sus scrofa	19,250	19,919	1,328	7.21	184.06	2,992
	Homo sapiens	18,010	26,821	1,601	8.67	184.65	3,288
	Capra hircus	18,332	23,723	1,438	8.00	179.69	3,182
	Bos taurus	17,840	25,478	1,577	8.61	183.26	3,142
Final set		21,983	37,092	1,544	8.94	172.49	4,472

Supplementary Table 10. Summary of evidence for the *GLEAN* gene models. P: ab initio prediction; H: homology-based. The evidence for the GLEAN gene model was further separated into single (with one gene source) and more (with two or more gene sources) according to the number of gene sources. The overlap threshold refers to the CDS region of GLEAN genes.

	⩾ 20% overlap		⩾ 50% overlap		⩾ 80% overlap	
	Number	Percentage (%)	Number	Percentage (%)	Number	Percentage (%)
P(single)	0	0.0	408	2.0	2,260	12.5
P(more)	2,290	10.7	2370	11.4	2,064	11.4
H(single)	6	0.0	30	0.1	254	1.4
H(more)	573	2.7	712	3.4	1,446	8.0
P+H	19,113	88.9	18,458	88.4	15,694	87.0

Supplementary Table 11. Statistics of the functional annotation of protein-encoding genes. Five protein databases were chosen to predict gene function. (InterPro, Gene ontology, KEGG, Swissprot and TrEMBL) and the numbers of genes matched to each database is shown.

		Number	Percent
Annotated	InterPro	18,485	84.1%
	GO	14,643	66.6%
	KEGG	17,298	78.7%
	Swissprot	19,575	89.0%
	TrEMBL	19,862	90.4%
Unannotated		2,056	9.4%

Supplementary Table 12. Assessment of the completeness of protein-encoding genes by running BUSCO in protein mode.

Types	Number	Percentage (%)
Complete BUSCOs (C)	3675	89.5
Complete and single-copy BUSCOs（S）	3632	88.5
Complete and duplicated BUSCOs (D)	43	1.0
Fragmented BUSCOs (F)	280	6.8
Missing BUSCOs (M)	149	3.6
Total BUSCO groups searched (n)	4104	

Supplementary Table 13. Comparison of statistics for protein-encoding genes in the current donkey genome assembly vs the genomes of six additional mammalian species.

Species	Gene number	Average mRNA length (bp)	Average CDS length (bp)	Average exon per gene	Average exon length (bp)	Average intron length (bp)
Equus asinus	21,983	37,092	1,544	8.94	172.5	4,472
Bos taurus	19,970	35,400	1,611	9.64	167.0	3,908
Capra hircus	22,172	29,969	1,385	8.23	168.4	3,956
Equus caballus	20,419	32,157	1,538	9.29	165.5	3,692
Homo sapiens	21,375	47,027	1,660	9.55	173.7	5,301
Mus musculus	22,927	35,443	1,551	8.64	179.3	4,430
Sus scrofa	21,577	27,367	1,375	8.46	162.5	3,482

Supplementary Table 14. Gene function enrichment using Gene Ontology for horse specific genes and horse-donkey orthologous genes. Unpaired *t* test, two tailed were used for data analysis. Adjustments were made for multiple comparisons with a false discovery rate (FDR) approach. (*12*).

Type	GO_ID	GO_Term	GO_Class	P value	Adjusted P value (FDR)
Horse specific genes	GO:0044422	organelle part	CC	6.92E-14	1.12E-11
	GO:0044446	intracellular organelle part	CC	1.68E-13	2.72E-11
	GO:0043228	non-membrane-bounded organelle	CC	1.38E-10	2.24E-08
	GO:0043229	intracellular organelle	CC	6.09E-09	9.87E-07
	GO:0044424	intracellular part	CC	1.10E-07	1.78E-05
	GO:0006955	immune response	BP	1.72E-05	2.79E-03
	GO:0043234	protein complex	CC	2.10E-05	3.41E-03
	GO:0004857	enzyme inhibitor activity	MF	7.22E-05	1.17E-02
	GO:0044455	mitochondrial membrane part	CC	9.12E-05	1.48E-02
	GO:0005787	signal peptidase complex	CC	1.72E-04	2.79E-02
	GO:0033177	proton-transporting two-sector ATPase complex, proton-transporting domain	CC	1.77E-04	2.87E-02
	GO:0031975	envelope	CC	1.83E-04	2.97E-02
	GO:0031090	organelle membrane	CC	2.04E-04	3.31E-02
Horse-donkey orthologous genes	GO:0005515	protein binding	MF	9.30E-05	3.24E-02

Supplementary Table 15. Comparison of heterozygosity rates for domestic donkeys and wild asses by aligning them to the current donkey assembly, the donkey assembly published by Renaud et al. (2018) (*1*) and the horse assembly (EquCab 2.0) reported by Wade et al (2009) (*13*).

Species name	Binomial nomenclature	Heterozygosity rate (aligned to the present donkey assembly)	Heterozygosity rate (aligned to the donkey assembly published by Renaud et al.)	Heterozygosity rate (aligned to the horse assembly published by Wade et al.)
Somali wild ass	*Equus africanus somaliensis*	0.07294%	0.05747%	0.08122%
Onager	*Equus hemionus onager*	0.17182%	0.18138%	0.21042%
Kiang	*Equus kiang*	0.11098%	0.10419%	0.12814%
Domestic donkey	*Equus asinus*	0.07798%	0.06814%	0.11371%

Supplementary Table 16. Chromosome distribution of high-quality variants and nucleotide diversity of each donkey chromosome. Density: variant sites counts per 1000 bp. Density equals to (variant counts/variants used region) × 1000. NRY refers to the non-recombining portion of the Y chromosome.

Chromosome	effective covered regions	effective covered regions proportion	SNPs		Short InDels		π (1×10^{-3})	θ_W (1×10^{-3})
			Count	Density	Count	Density		
1	111,458,806	93.4%	328,523	2.9	32,886	0.30	0.806	0.487
2	222,898,805	93.3%	698,508	3.1	66,165	0.30	0.851	0.517
3	160,737,176	87.5%	512,812	3.2	51,858	0.32	0.869	0.527
4	82,637,037	89.1%	254,689	3.1	25,632	0.31	0.841	0.509
5	103,934,485	92.6%	328,266	3.2	30,580	0.29	0.853	0.521
6	86,929,515	93.2%	273,297	3.1	26,135	0.30	0.856	0.519
7	115,629,073	93.7%	360,445	3.1	34,598	0.30	0.823	0.514
8	94,210,208	90.5%	423,281	4.5	37,791	0.40	1.182	0.742
9	60,013,828	92.8%	180,603	3.0	17,556	0.29	0.833	0.497
10	83,339,204	92.0%	268,094	3.2	24,434	0.29	0.878	0.531
11	79,665,214	92.9%	261,888	3.3	26,926	0.34	0.887	0.544
12	99,158,427	93.3%	294,257	3.0	28,566	0.29	0.799	0.490
13	57,453,710	88.6%	167,727	2.9	15,723	0.27	0.776	0.482
14	41,254,786	86.7%	136,927	3.3	12,255	0.30	0.904	0.548
15	47,243,892	94.1%	152,321	3.2	13,077	0.28	0.867	0.532
16	47,509,008	93.7%	136,639	2.9	13,692	0.29	0.79	0.475
17	40,871,542	85.9%	164,773	4.0	15,125	0.37	1.059	0.666
18	30,601,207	92.3%	104,172	3.4	9,589	0.31	0.928	0.562
19	25,071,840	92.9%	87,979	3.5	7,914	0.32	0.93	0.579
20	91,931,664	91.5%	306,366	3.3	28,721	0.31	0.891	0.550
21	93,088,626	94.4%	272,906	2.9	26,199	0.28	0.806	0.484
22	35,657,514	93.0%	108,641	3.0	10,607	0.30	0.833	0.503
23	43,272,448	91.4%	132,759	3.1	12,636	0.29	0.857	0.507
24	43,461,268	93.2%	134,620	3.1	13,569	0.31	0.831	0.512
25	42,693,163	90.6%	127,926	3.0	12,312	0.29	0.792	0.494
26	25,469,740	88.9%	95,251	3.7	8,246	0.32	0.99	0.618
27	29,902,531	93.0%	107,379	3.6	10,278	0.34	0.97	0.593
28	57,591,390	90.2%	191,311	3.3	16,746	0.29	0.869	0.548
29	34,858,052	93.1%	119,612	3.4	10,857	0.31	0.912	0.566
30	27,915,461	92.2%	99,881	3.6	9,670	0.35	0.972	0.591
total autosome	2,116,459,620	91.7%	6,831,853	3.2	650,343	0.31	0.871	0.533
X	85,568,695	78.9%	173,113	2.0	14,086	0.16	0.479	0.337
NRY	2,888,055	23.0%	981	0.3	85	0.03	0.035	0.073

Supplementary Table 17. Annotation of high confidence variants. Flanking refers to regions ± 2 kb upstream and downstream of genes. The "non-sy/sy" term refers to non-synonymous SNPs divided by synonymous SNPs. "Percentages" refers to the proportions of SNPs or Indels divided by the total number of SNPs or Indels.

Regions of the genome where SNPs were located	Number of SNPs	Percentages of SNPs	Regions of the genome where Indels were located	Number of Short Indels	Percentages of short Indels
CDS	65,941	0.94%	CDS	2,089	0.30%
Intron	2,222,953	31.77%	Intron	222,589	32.43%
Flanking	174,936	2.50%	Flanking	17,959	2.62%
Intergenic	4,534,038	64.79%	Intergenic	443,693	64.65%
non-sy/sy	33062/32879 is1.01		Frameshift	1,545	0.23%

Supplementary Table 18. Nucleotide diversity (π, 1×10^{-3}) and Watterson's estimator (θ_W, 1×10^{-3}) per bp of the polymorphism of Tropical Africa and North Africa & Eurasia donkeys.

	Tropical Africa donkeys	North Africa & Eurasia donkeys	All donkeys
π	0.697	0.857	0.867
θ_W	0.644	0.576	0.552

Supplementary Table 19. D statistics obtained through the comparison of different donkey populations in the form of (((P1, P2), African wild ass), Asian wild ass).

P1	P2	P3 (African wild ass)	P4 (Asian wild ass)	D statistic	Standard error	Z-value
Kenya	Iran	African wild ass	Asian wild ass	0.0055	0.003458	1.58
Kenya	Australia	African wild ass	Asian wild ass	0.012	0.003874	3.102
Kenya	Kyrgyzstan	African wild ass	Asian wild ass	0.0065	0.00301	2.169
Kenya	Spain	African wild ass	Asian wild ass	0.0057	0.002668	2.138
Kenya	China	African wild ass	Asian wild ass	0.0006	0.002527	0.237
Kenya	Egypt	African wild ass	Asian wild ass	0.0032	0.002715	1.189
Iran	Australia	African wild ass	Asian wild ass	0.0067	0.00417	1.615
Spain	Australia	African wild ass	Asian wild ass	0.0064	0.00357	1.804
Egypt	Iran	African wild ass	Asian wild ass	0.0022	0.003128	0.711
Egypt	Australia	African wild ass	Asian wild ass	0.0089	0.003807	2.333
Egypt	Kyrgyzstan	African wild ass	Asian wild ass	0.0033	0.002895	1.137
Egypt	Spain	African wild ass	Asian wild ass	0.0025	0.002331	1.082

Supplementary Table 20. Results of the D statistics tests performed to detect admixture events between Tropical Africa donkeys and North Africa & Eurasia donkeys. The term **"**O" refers to the outgroup. Negative D statistics indicate that gene flow has occurred between population A and population B, and positive D statistics indicate that gene flow has occurred between population A and population C. If the absolute value of Z-value is bigger than three, the test is significant.

O	A	B	C	D statistic	Z-value
Asian wild ass	Ethiopia	Kenya	Kyrgyzstan	-0.053	-28.288
Asian wild ass	Spain	Australia	Kenya	-0.0382	-19.599
Asian wild ass	Spain	Australia	Nigeria	-0.0317	-13.769
Asian wild ass	Spain	Egypt	Ethiopia	-0.0188	-12.263
Asian wild ass	Iran	Egypt	Nigeria	-0.0204	-11.451
Asian wild ass	Iran	Egypt	Ethiopia	-0.018	-11.226
Asian wild ass	Spain	Egypt	Nigeria	-0.0196	-10.993
Asian wild ass	Iran	Kyrgyzstan	Nigeria	-0.0192	-9.982
Asian wild ass	Iran	China	Nigeria	-0.0158	-8.671
Asian wild ass	Ethiopia	Nigeria	Kyrgyzstan	-0.0151	-7.68
Asian wild ass	Iran	Spain	Nigeria	-0.014	-7.43
Asian wild ass	Nigeria	Ethiopia	Kyrgyzstan	-0.0165	-7.312
Asian wild ass	Ethiopia	Egypt	Kyrgyzstan	-0.0099	-6.039
Asian wild ass	Nigeria	Kenya	Kyrgyzstan	-0.0118	-5.746
Asian wild ass	Spain	China	Nigeria	-0.0082	-4.955
Asian wild ass	Ethiopia	China	Kyrgyzstan	-0.0064	-4.675
Asian wild ass	Nigeria	Egypt	Kyrgyzstan	-0.0083	-4.302
Asian wild ass	Spain	Kyrgyzstan	Nigeria	-0.006	-3.476
Asian wild ass	Ethiopia	Spain	Kyrgyzstan	-0.0051	-3.313
Asian wild ass	Nigeria	Spain	Kyrgyzstan	-0.0059	-3.155
Asian wild ass	Spain	Iran	Nigeria	-0.0053	-2.725
Asian wild ass	Iran	Australia	Nigeria	-0.0045	-1.844
Asian wild ass	Iran	Ethiopia	Nigeria	-0.0023	-1.187
Asian wild ass	Iran	Kenya	Nigeria	-0.001	-0.542
Asian wild ass	Spain	Ethiopia	Nigeria	-0.0006	-0.344
Asian wild ass	Ethiopia	Australia	Kyrgyzstan	0.0001	0.062
Asian wild ass	Nigeria	China	Kyrgyzstan	0.0013	0.797
Asian wild ass	Ethiopia	Iran	Kyrgyzstan	0.0019	1.236
Asian wild ass	Nigeria	Iran	Kyrgyzstan	0.0028	1.507
Asian wild ass	Nigeria	Australia	Kyrgyzstan	0.0042	1.584
Asian wild ass	Spain	Kenya	Nigeria	0.0067	3.872
Asian wild ass	Australia	China	Egypt	0.0072	4.103
Asian wild ass	Australia	China	Spain	0.0316	19.724
Asian wild ass	Australia	Egypt	Ethiopia	-0.0119	-6.222
Asian wild ass	Australia	Egypt	Kenya	-0.0261	-12.143
Asian wild ass	Australia	Egypt	Spain	0.0245	13.869
Asian wild ass	Australia	Ethiopia	Spain	0.0361	18.821
Asian wild ass	Australia	Kyrgyzstan	Spain	0.0385	19.135

Supplementary Table 21. Genotype distribution of the short deletion at chromosome 8 (chr8:g.42742556 CT>C-) in Dun and non-Dun donkeys. The term “Photo” means that a photographic record of the coat color was taken; “record” means coat color was not photographed but annotated. “Genotype” means the genotype of the Indel (chr8: 42742556) in the downstream region of the *TBX3 g*ene EAS0007835 (*TBX3*, chr8: 42723946-42735174) , which is associated with coat color in donkeys.

Dun donkey			non-dun donkey		
accessions	photo/record	genotype	accessions	photo/record	genotype
Ch-xj1	photo	CT/C-	Ch-dz	photo	C-/C-
Ch-xj2	photo	CT/C-	Ch-by1	photo	C-/C-
Ch-xj3	photo	CT/C-	Ch-by2	photo	C-/C-
Ch-xj4	photo	CT/C-	Ch-by3	photo	C-/C-
Ch-xj5	photo	missing	Ch-by4	photo	C-/C-
Ch-yn1	photo	CT/C-	Ch-gl1	photo	C-/C-
Ch-yn2	photo	CT/C-	Ch-gl2	photo	C-/C-
Ch-yn3	photo	CT/C-	Ch-gl3	photo	C-/C-
Ch-kl1	photo	CT/C-	Ch-gl4	photo	C-/C-
Ch-kl2	photo	CT/C-	Eg-4	photo	C-/C-
Ch-kl3	photo	CT/C-	Sp-1	record	C-/C-
Ch-kl4	photo	CT/CT	Sp-2	record	C-/C-
Ch-ht1	photo	CT/C-	Sp-3	record	C-/C-
Ch-ht2	photo	CT/CT	Sp-10	record	C-/C-
Ch-tlf4	photo	CT/C-	Sp-11	record	C-/C-
Au-1	photo	CT/CT	Sp-12	record	C-/C-
Eg-2	photo	CT/C-	Sp-13	record	C-/C-
Et-1	photo	CT/C-	Sp-14	record	C-/C-
Et-2	photo	CT/C-	Sp-15	record	C-/C-
Et-3	photo	CT/C-	Sp-16	record	C-/C-
Et-7	photo	CT/C-	Sp-17	record	C-/C-
Et-8	photo	CT/C-	/	/	/
Et-9	photo	CT/C-	/	/	/

Supplementary Table 22. Gene expression measured in fragments per kilobase millions (FPKM) of ten melanogenic or melanocyte regulatory genes in the dorsal stripe skin of non-Dun and Dun donkeys. Three columns are included for each detected gene, displaying the average expression level in non-Dun and Dun donkeys, as well as the $\log_2$ fold-change of expression (an absolute value higher than 1 indicates a significant change in gene expression).

Gene ID	Gene symbol	Mean FPKM across non-Dun donkeys	Mean FPKM across Dun donkeys	$\log_2$ (non-Dun/Dun)
EAS0019088	*DCT*	30.96	32.95	-0.09
EAS0012237	*KIT*	6.21	5.93	0.07
EAS0020811	*KITLG*	0.16	0.22	-0.46
EAS0012784	*MC1R*	11.28	12.6	-0.16
EAS0013342	*MLANA*	32.8	31.68	0.05
EAS0006134	*OCA2*	10.97	10.78	0.03
EAS0015035	*SLC24A5*	22.81	23.13	-0.02
EAS0006150	*TRPM1*	11.47	10.86	0.08
EAS0018171	*TYR*	36.11	36.44	-0.01

Supplementary Table 23. Genes mapping to selective sweeps detected in the comparison of non-Dun and Dun donkeys and their expression in the croup skin. Homologous genes in horse and their mRNA expression fold-change between Dun and non-Dun croup skin are also indicated (data obtained from Imsland et al. (*14*). FPKM refers to fragments per kilobase millions.

Species	chromosome	Gene ID	Gene symbol	Mean FPKM across non-Dun donkeys	Mean FPKM across Dun donkeys	$\log_2$ (non-Dun/Dun)
Donkey	chr03	EAS0006038	*LCORL*	0.27	0.25	0.09
	chr03	EAS0006039	*NCAPG*	6.66	5.91	0.17
	chr03	EAS0006040	*DCAF16*	3.51	2.94	0.26
	chr03	EAS0006041	*FAM184B*	0.16	0.12	0.38
	chr03	EAS0006042	*MED28*	21.06	23.2	-0.14
	chr10	EAS0008231	*SLC44A1*	30.3	31.66	-0.06
	chr10	EAS0008232	*RDH14*	0.02	0.07	-1.65
	chr10	EAS0008233	*ABCA1*	4.23	4.24	0
	chr10	EAS0008234	*NIPSNAP3A*	12.38	12.16	0.03
Horse	/	ENSECAG00000000648	*LCORL*	/	/	-0.35
	/	ENSECAG00000007470	*NCAPG*	/	/	-0.14
	/	ENSECAG00000002410	*DCAF16*	/	/	0.13
	/	ENSECAG00000013661	*FAM184B*	/	/	-0.07
	/	ENSECAG00000017465	*MED28*	/	/	-0.07
	/	ENSECAG00000002233	*SLC44A1*	/	/	0.09
	/	ENSECAG00000008852	*ABCA1*	/	/	-0.18
	/	ENSECAG00000006103	*NIPSNAP3A*	/	/	-0.1

Supplementary References

1. Renaud, G. et al. Improved de novo genomic assembly for the domestic donkey. *Sci. Adv.* **4**, eaaq0392 (2018).
2. Yang, F. et al. Refined genome-wide comparative map of the domestic horse, donkey and human based on cross-species chromosome painting: insight into the occasional fertility of mules. *Chromosome Res*. **12**, 65-76 (2004).
3. Swinburne J. E. et al. Single linkage group per chromosome genetic linkage map for the horse, based on two three-generation, full-sibling, crossbred horse reference families. *Genomics* **87**, 1-29 (2006).
4. Piras, F. M. et al. Phylogeny of horse chromosome 5q in the genus Equus and centromere repositioning. *Cytogenet. Genome Res.* **126**, 165-172 (2009).
5. Kent, W. J. BLAT—the BLAST-like alignment tool. *Genome Res.* **12**, 656-664 (2002).
6. Huang, J. et al. Donkey genome and insight into the imprinting of fast karyotype evolution. *Sci. Rep*. **5**, 14106 (2015).
7. Simão, F. A., Waterhouse, R. M., Ioannidis, P., Kriventseva, E. V. & Zdobnov, E. M. BUSCO: assessing genome assembly and annotation completeness with single-copy orthologs. *Bioinformatics* **31**, 3210-3212 (2015).
8. Pickrell, J. K. & Pritchard, J. K. Inference of population splits and mixtures from genome-wide allele frequency data. P*LoS Genet.* **8**, e1002967 (2012).
9. Hudson, R. R. Generating samples under a Wright-Fisher neutral model of genetic variation. *Bioinformatics* **18**, 337-338 (2002).
10. Suchard, M. A. et al. Bayesian phylogenetic and phylodynamic data integration using BEAST 1.10. *Virus Evol.* **4**, vey016 (2018).

11. Lindgren, G. et al. Limited number of patrilines in horse domestication. *Nat. Genet.* **36**, 335-336 (2004).

12. Benjamini, Y. & Hochberg, Y. Controlling the false discovery rate: a practical and powerful approach to multiple testing. *J. R. Stat. Soc. B.* **57**, 289-300 (1995).

13. Wade, C. M. et al. Genome sequence, comparative analysis, and population genetics of the domestic horse. *Science* **326**, 865-867 (2009).

14. Imsland, F. et al. Regulatory mutations in *TBX3* disrupt asymmetric hair pigmentation that underlies Dun camouflage color in horses. *Nat. Genet.* **48**, 152-158 (2016).
