# Supplementary material for: Donkey genomes provide new insights into domestication and selection for coat color
Source: Nat Commun. 2020 Dec 8;11:6014. doi: 10.1038/s41467-020-19813-7 (PMC7723042; doi:10.1038/s41467-020-19813-7)
Supplement: Supplementary file 2 — Reporting Summary [file 41467_2020_19813_MOESM2_ESM.pdf]

nature research

Corresponding author(s): Jifeng Zhong

Last updated by author(s): Oct 10, 2020

Reporting Summary

Nature Research wishes to improve the reproducibility of the work that we publish. This form provides structure for consistency and transparency in reporting. For further information on Nature Research policies, see our Editorial Policies and the Editorial Policy Checklist.

Statistics

For all statistical analyses, confirm that the following items are present in the figure legend, table legend, main text, or Methods section.

n/a	Confirmed	
☐	☒	The exact sample size (n) for each experimental group/condition, given as a discrete number and unit of measurement
☐	☒	A statement on whether measurements were taken from distinct samples or whether the same sample was measured repeatedly
☐	☒	The statistical test(s) used AND whether they are one- or two-sided<br>*Only common tests should be described solely by name; describe more complex techniques in the Methods section.*
☒	☐	A description of all covariates tested
☐	☒	A description of any assumptions or corrections, such as tests of normality and adjustment for multiple comparisons
☐	☒	A full description of the statistical parameters including central tendency (e.g. means) or other basic estimates (e.g. regression coefficient) AND variation (e.g. standard deviation) or associated estimates of uncertainty (e.g. confidence intervals)
☐	☒	For null hypothesis testing, the test statistic (e.g. F, t, r) with confidence intervals, effect sizes, degrees of freedom and P value noted<br>*Give P values as exact values whenever suitable.*
☐	☒	For Bayesian analysis, information on the choice of priors and Markov chain Monte Carlo settings
☐	☒	For hierarchical and complex designs, identification of the appropriate level for tests and full reporting of outcomes
☒	☐	Estimates of effect sizes (e.g. Cohen's d, Pearson's r), indicating how they were calculated

Our web collection on statistics for biologists contains articles on many of the points above.

Software and code

Policy information about availability of computer code

Data collection	No software was used to collect data.
Data analysis	Software used in the study was as follows: SOAPdenovo (v2.04.4), DBG2OLC, Pilon (v1.22), BESST (v2.2.7), PBSuite (v15.8.24), HiC-Pro (v2.8.0), Bowtie 2, Juicer (v1.5), 3d-dna (v170123), BLASTZ, BWA (v0.7.10-r789), BamDeal-0.19, Tandem Repeats Finder (v4.04), RepeatMasker (v4.0.4), RepeatModeler (v1.05), RECON (v1.08), REPEATSCOUT (v1.0.6), LTR_finder, TBLASTN (v2.2.26), AUGUSTUS (v.2.5.5), GENSCAN (v1.0), BLASTP, InterProScan (v55.0), Trimmomatic (v0.36), SAMtools (v1.3.1), GATK (v3.3.0-g37228af), Picard (v1.117), Primer-BLAST, PLINK (v1.90), Fneighbor, iTOL, GCTA (v1.92.4), ADMIXTURE (v1.23), CLUMPP (v1.1.2), Distruct (v1.1), MEGA (v6.06), VCFtools (v0.1.13), PopLDdecay (v3.40), ADMIXtools (v5.1), EIGENSOFT (v6.0.1), PSMC (v0.6.5), SMC++ (v1.13), BEAST (v2.5.2), ms, PopART (v1.7), SHAPEIT (v2.r790), and DESeq2 (v1.28.1).<br>Custom perl scripts were deposited into Github (https://github.com/JINPENG-WANG/scripts-for-donkey-genomes-sequencing).

For manuscripts utilizing custom algorithms or software that are central to the research but not yet described in published literature, software must be made available to editors and reviewers. We strongly encourage code deposition in a community repository (e.g. GitHub). See the Nature Research guidelines for submitting code & software for further information.

Data

Policy information about availability of data

All manuscripts must include a data availability statement. This statement should provide the following information, where applicable:

- Accession codes, unique identifiers, or web links for publicly available datasets
- A list of figures that have associated raw data
- A description of any restrictions on data availability

Data from whole-genome sequencing, resequencing and transcriptome sequencing have been deposited in the GenBank database under BioProject accession

PRJNA431818. Sanger sequences of the Dun, non-dun1, and non-Dun2 dun2 deletion alleles from horse have been submitted to GenBank undercan be accessed with accessions KT896508, KT896509 and KT896510, respectively-KT896515. In addition, we obtained resequencing data for 3 Asian wild asses (accession numbers: SRR1562345, ERR650932-ERR654612, ERR669419-ERR669469), one Somali wild ass (accession numbers : ERR650540-ERR650547, and ERR650570-ERR650703), one domestic donkey (accession number: SRR873443-SRR873445), and for 42 domestic donkeys and one kiang donkey accessible from the National Genomics Data Center (https://bigd.big.ac.cn/bioproject/browse/PRJCA001131). All relevant data are available from the authors. Supplementary Information and Source Data data files are available in the online version of the paperprovided with this paperSanger sequences of the non-Dun2 deletion have been submitted to GenBank under accessions KT896508-KT896515. In addition, we obtained resequencing data for 3 Asian wild asses (accession numbers: SRR1562345, ERR650932-ERR650969, and ERR654542-ERR654612), one Somali wild ass (accession numbers : ERR650540-ERR650547, and ERR650570-ERR650703), one domestic donkey (accession number: SRA082086), and for 42 domestic donkeys and one kiang donkey accessible from the National Genomics Data Center (https://bigd.big.ac.cn/bioproject/browse/Data from whole-genome sequencing, resequencing and transcriptome sequencing have been deposited in the GenBank database under BioProject accession PRJNA431818 [https://www.ncbi.nlm.nih.gov/bioproject/?term=prjna431818]. Sanger sequences of the Dun, non-dun1, and non-dun2 alleles from horse can be accessed with accessions KT896508 [https://www.ncbi.nlm.nih.gov/nuccore/KT896508.1], KT896509 [https://www.ncbi.nlm.nih.gov/nuccore/KT896509.1] and KT896510 [https://www.ncbi.nlm.nih.gov/nuccore/KT896510.1], respectively. In addition, we obtained resequencing data for 3 Asian wild asses (accession numbers: SRR1562345 [https://trace.ncbi.nlm.nih.gov/Traces/sra/?run=SRR1562345], ERR650932-ERR654612 [https://www.ncbi.nlm.nih.gov/Traces/study/?page=2&acc=SAMEA2802530&o=acc_s%3Aa], ERR669419-ERR669469 [https://www.ncbi.nlm.nih.gov/Traces/study/?acc=SAMEA2802529&o=acc_s%3Aa]), one Somali wild ass (accession numbers : ERR650540-ERR650547 [https://www.ncbi.nlm.nih.gov/Traces/study/?acc=SAMEA2802531&o=acc_s%3Aa], and ERR650570-ERR650703 [https://www.ncbi.nlm.nih.gov/Traces/study/?acc=SAMEA2802531&o=acc_s%3Aa]), one domestic donkey (accession number: SRR873443-SRR873445 [https://www.ncbi.nlm.nih.gov/Traces/study/?acc=SAMN02179859&o=acc_s%3Aa]), and for 42 domestic donkeys and one kiang donkey accessible from the National Genomics Data Center (https://bigd.big.ac.cn/bioproject/browse/PRJCA001131). All relevant data are available from the authors. Source data are provided with this paper.

Field-specific reporting

Please select the one below that is the best fit for your research. If you are not sure, read the appropriate sections before making your selection.

- [ ] Life sciences
- [ ] Behavioural & social sciences
- [x] Ecological, evolutionary & environmental sciences

For a reference copy of the document with all sections, see nature.com/documents/nr-reporting-summary-flat.pdf

Ecological, evolutionary & environmental sciences study design

All studies must disclose on these points even when the disclosure is negative.

Study description	We de novo assemble the genome of Dezhou donkey, Chinese important donkey breed. We also want to infer the domestication history of domestic donkey. We collected donkey samples from nine countries. By re-sequencing the whole genome of them, we can tell the genetic relationship of donkeys and infer their domestication site or spread route. We also intend to identify the underlying gene controlling the coat color of domestic donkey.
Research sample	To comprehensively infer the demographic history of domestic donkey, we collected donkey samples from nine countries across Africa, Europe, and Asia. As donkeys were reported to be domesticated in Africa and then spread to Europe and Asia, we sampled donkey samples from the potential domestication centers (African countries including Egypt, Ethiopia, etc.) and along the spread routine to European and Asian countries. As donkeys were reported to be imported to Australia from European countries, we also sampled donkeys from Australia to determine the genetic divergence between Australian donkeys and donkeys from Old World. To infer the domestication history, we also collect wild ass samples from zoo to measure the divergence between wild asses and domestic donkeys. The samples were representatives of the main donkey breeding countries.<br>As we intended to infer paternal and maternal history of donkeys, we sampled both male and female donkeys. For population analysis, we did not consider the age of donkey samples as the age of donkey samples had no effect on the results. For the transcriptome sequencing, three male donkeys aged at 2-2.5 years old were sampled.
Sampling strategy	For historic effective population size inferring analyses, sample size has little effect on the result as the analyses involved bootstrapping analysis which usually run 100-1000 times that would support enough statistical support. We sample about ten samples from each country, which still support enough statistical support when calculating nucleotide diversity.
Data collection	For the de novo sequencing, sequencing data was collected by Illumina Hiseq sequencing, PacBio sequencing, and Hi-C sequencing. Jiumeng Min was responsible for this part.<br>For the resequencing data, Illumina Hiseq sequencing platform was used and Yu Guo was responsible for this.<br>For the quantification of the expression level of the TBX3 gene which was determined by qPCR procedure, the data collection was supervised by Jinpeng Wang.
Timing and spatial scale	For the de novo sequencing, the data collection started at Jan 12, 2015 and ended at April 13, 2015. For the resequencing, the data collection started at Feb 15, 2015 and ended at May 16, 2015. For the transcriptome sequencing, the collection started at Feb 16, 2015 and ended at May 20, 2015. All above data was collected at BGI company, Shenzhen, China. As the sequencing data was collected at fully automated machines, the data collection was finished at one-shot.
Data exclusions	No data was excluded.
Reproducibility	When determining the gene expression level, at least three samples were used to make the data to be reproducibility. All attempts at replication were successful. In addition, immunofluorescence was replicated three times and all attempts at replication were also successful.

Randomization	When determining the expression level of TBX3 gene and relevant pigmentation deposition genes, the black donkeys and gray donkeys were sampled randomly from a big donkey population which has about 1000 individuals.
Blinding	When determining the expression level of TBX3 gene , the gene name and individual names were re-coded and the experiment was performed by a laboratory technician who did not know the experiment design.

Did the study involve field work? ☐ Yes ☒ No

Reporting for specific materials, systems and methods

We require information from authors about some types of materials, experimental systems and methods used in many studies. Here, indicate whether each material, system or method listed is relevant to your study. If you are not sure if a list item applies to your research, read the appropriate section before selecting a response.

Materials & experimental systems

n/a	Involved in the study	
☐	☒	Antibodies
☒	☐	Eukaryotic cell lines
☒	☐	Palaeontology and archaeology
☐	☒	Animals and other organisms
☒	☐	Human research participants
☒	☐	Clinical data
☒	☐	Dual use research of concern

Methods

n/a	Involved in the study	
☒	☐	ChIP-seq
☒	☐	Flow cytometry
☒	☐	MRI-based neuroimaging

Antibodies

Antibodies used	Anti-TBX3 (bs-10266R) diluted at 1:200.
Validation	It can react with TBX3 proteins from Human, Mouse, Rat, Dog, Pig, Cow, Horse, Rabbit as indicated by the manufacturers. The validation was carried out in mouse brain samples as followed: Paraformaldehyde-fixed, paraffin embedded (Mouse brain); Antigen retrieval by boiling in sodium citrate buffer (pH6.0) for 15min; Block endogenous peroxidase by 3% hydrogen peroxide for 20 minutes; Blocking buffer (normal goat serum) at 37°C for 30min; Antibody incubation with (TBX3) Polyclonal Antibody, Unconjugated (bs-10266R) at 1:400 overnight at 4°C, followed by operating according to SP Kit(Rabbit) (sp-0023) instructionsand DAB staining.

Animals and other organisms

Policy information about studies involving animals; ARRIVE guidelines recommended for reporting animal research

Laboratory animals	We used Dezhou donkey (Equus asinus), which is one major donkey breed in China, for de novo sequencing. This sample was a 6-month-old male donkey.
Wild animals	We collected blood samples of two Asian wild asses from zoo, one was male, 4-year-old and the other was female, 5-year-old.
Field-collected samples	This study did not involve samples collected from field.
Ethics oversight	Animal care and research procedures were carried out in accordance with the guiding principles for the care and use of laboratory animals, being approved by the Institutional Animal Care and Use Committee at Shandong Academy of Agricultural Sciences (SAAS).

Note that full information on the approval of the study protocol must also be provided in the manuscript.
