# Supplementary material for: Donkey genomes provide new insights into domestication and selection for coat color
Source: Nat Commun. 2020 Dec 8;11:6014. doi: 10.1038/s41467-020-19813-7 (PMC7723042; doi:10.1038/s41467-020-19813-7)
Supplement: Supplementary file 3 — Description of Additional Supplementary Files [file 41467_2020_19813_MOESM3_ESM.pdf]

Description of Additional Supplementary Data

Supplementary Data 1

Assignment of assembled scaffolds to the corresponding chromosomes. The first column is our assembled scaffolds. The second clolumn is the corresponding horse chromosome of specific scaffold indicated by lastz alignment result. The third column is the corresponding human chromosome of specific scaffold indicated by lastz alignment result. The forth column is the assigned donkey chromosome of each scaffold. The fifth column is the assignment remarks of some complex situations.

Supplementary Data 2

The subtype of transposable elements (TEs) content in the assembled donkey genome.

Supplementary Data 3
The mRNA expression profiles of 17 samples from 13 tissues. Skin and muscle were sampled from three different donkeys separately. Gene expressions were represented in FPKM (fragments per kilobase million).

Supplementary Data 4
Information of all re-sequenced donkey samples and statistics of sequencing data and mapping data.

Supplementary Data 5
Experimental conditions and results of the SNP confirmation experiment carried out by Sanger sequencing.

Supplementary Data 6
The Admixture result when K=3. We defined the African, Eurasian and Australian group based on the Admixture result.

Supplementary Data 7
Bootstrap confidence intervals (100 replicates) around PSMC estimates for 133 samples (including six Asian wild asses, one African wild ass, and 126 domestic donkeys). The following acronyms have been used: Ke (Kenya), Ch (China), Ni (Nigeria), Ir (Iran), Sp (Spain), Eg (Egypt), Et (Ethiopia), Ti (Tibetan), Au (Australia), and Don (the European donkey). The generation time was set to eight years (g=8) and the neutral mutation rate μ was set to $7.242\times10\text{-}9$ mutations per generation and site.

Supplementary Data 8
Parameters used for ms command lines.

Supplementary Data 9
Phylogenetic tree based on Y chromosome SNPs from wild asses and domestic donkeys. Sample names in gold color represent North Africa & Eurasia donkeys while sample names in red represent Tropical Africa donkeys. A total of 13,032 SNPs mapping to the Y chromosome were used to construct the tree. FigTree v1.4.4 (http://tree.bio.ed.ac.uk/software/figtree/) was used to display the tree. To make the tree more readable, the branches of the tree were transformed to be cladogram. BEAST2 was applied in this phylogenetic analysis. The parameters for generating the maximum clade credibility (MCC) tree are: HKY for site model, strict clock model for clock model (clock rate = 1), Yule model for tree priors. The

phylogenetic tree was generated by Bayesian Markov chain Monte Carlo (MCMC) with 1000 simulations. Statistical support of each node was assessed by MCMC posterior probability indicated by the number aside each node. The following acronyms have been used: Ke (Kenya), Ch (China), Ni (Nigeria), Ir (Iran), Sp (Spain), Eg (Egypt), Et (Ethiopia), Ti (Tibetan), Au (Australia), and Don (the European donkey). Both AW2 and Ona refers to Asian wild asses.

Supplementary Data 10
Phylogenetic tree based on SNPs mapping to the mitochondrial genome of Somali wild ass and domestic donkeys. Sample names in gold color represent North Africa & Eurasia donkeys while sample names in red represent Tropical Africa donkeys. A total of 953 mitochondrial SNPs were used to construct the tree. FigTree v1.4.4 (http://tree.bio.ed.ac.uk/software/figtree/) was used to display the tree. To make the tree more readable, the branches of the tree were transformed to be cladogram. BEAST2 software (90) was applied in this phylogenetic analysis. The parameters for generating the maximum clade credibility (MCC) tree are: HKY for site model, strict clock model for clock model (clock rate = 1), Yule model for tree priors. The phylogenetic tree was generated by Bayesian Markov chain Monte Carlo (MCMC) with 1000 simulations. Statistical support of each node was assessed by MCMC posterior probability indicated by the number aside each node. The following acronyms have been used: Ke (Kenya), Ch (China), Ni (Nigeria), Ir (Iran), Sp (Spain), Eg (Egypt), Et (Ethiopia), Ti (Tibetan), Au (Australia), and Don (the European donkey).

Supplementary Data 11
Phased variants mapping to a chromosome 8 region going from the beginning of TBX3 (position 42,723,946) to 20,000bp downstream (position 42,743,946). Only SNPs with MAF>=0.1 were considered.

Supplementary Data 12
Results of a RNA-Seq differential gene expression study comparing the croup skin transcriptomes from Dun and non-Dun donkeys. Three columns are included for each detected gene, displaying the average expression level in non-Dun and Dun donkeys, as well as the log2 fold-change between them. FPKM refers to fragments per kilobase million.
