# Supplementary material for: Donkey genomes provide new insights into domestication and selection for coat color
Source: Nat Commun. 2020 Dec 8;11:6014. doi: 10.1038/s41467-020-19813-7 (PMC7723042; doi:10.1038/s41467-020-19813-7)
Supplement: Supplementary file 10 — Supplementary Data 7 [file 41467_2020_19813_MOESM10_ESM.pdf]

**Supplementary Data 7**. Bootstrap confidence intervals (100 replicates) around PSMC estimates for 133 samples (including six Asian wild asses, one African wild ass, and 126 domestic donkeys). The following acronyms have been used: Ke (Kenya), Ch (China), Ni (Nigeria), Ir (Iran), Sp (Spain), Eg (Egypt), Et (Ethiopia), Ti (Tibetan), Au (Australia), and Don (the European donkey). The generation time was set to eight years (g=8) and the neutral mutation rate $\mu$ was set to $7.242\times10^{-9}$ mutations per generation and site.

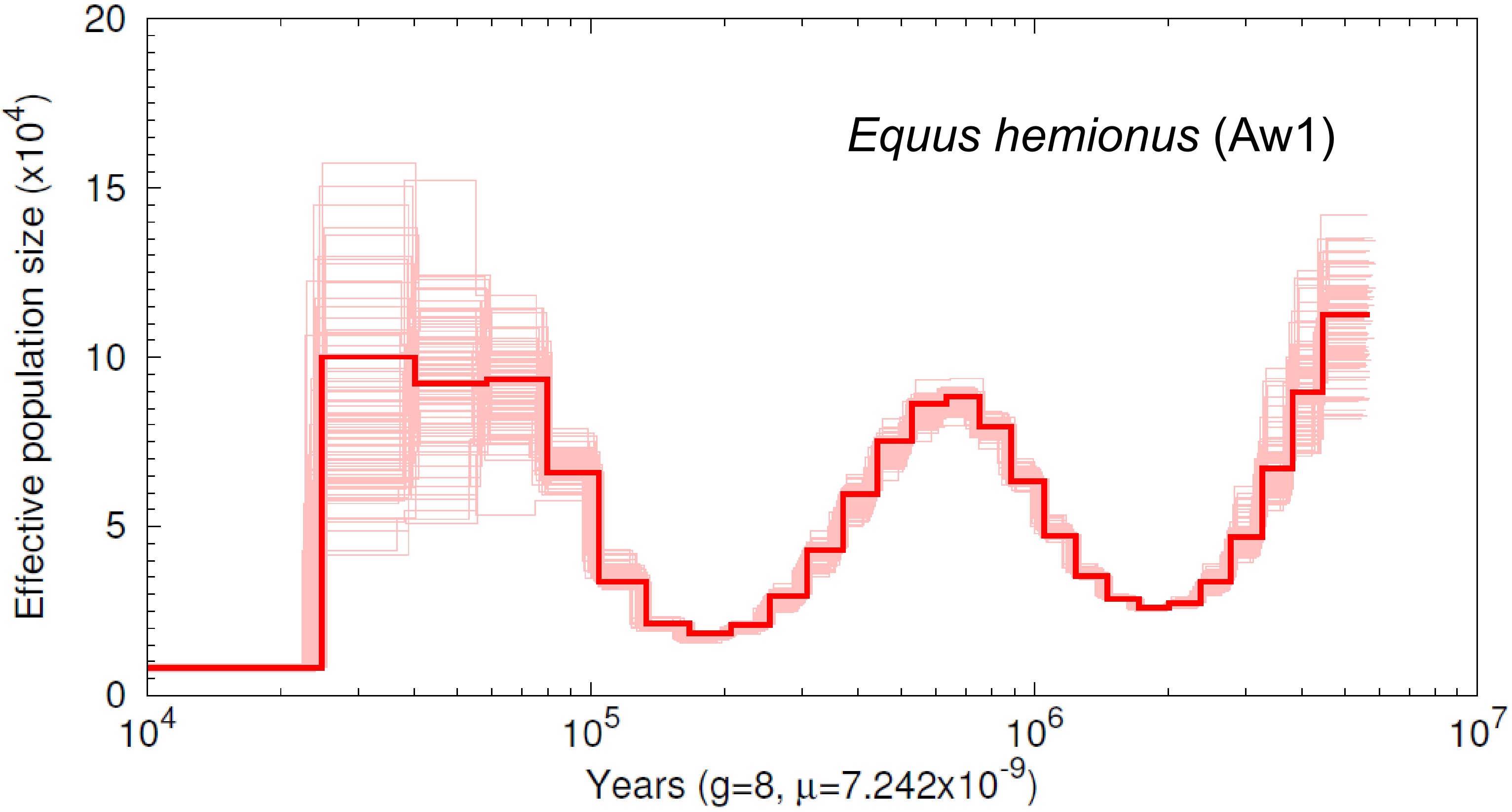
Equus hemionus (Aw1)
Effective population size (x10^4)
20
15
10
5
0
10^4
10^5
10^6
10^7
Years (g=8, μ=7.242x10^-9)

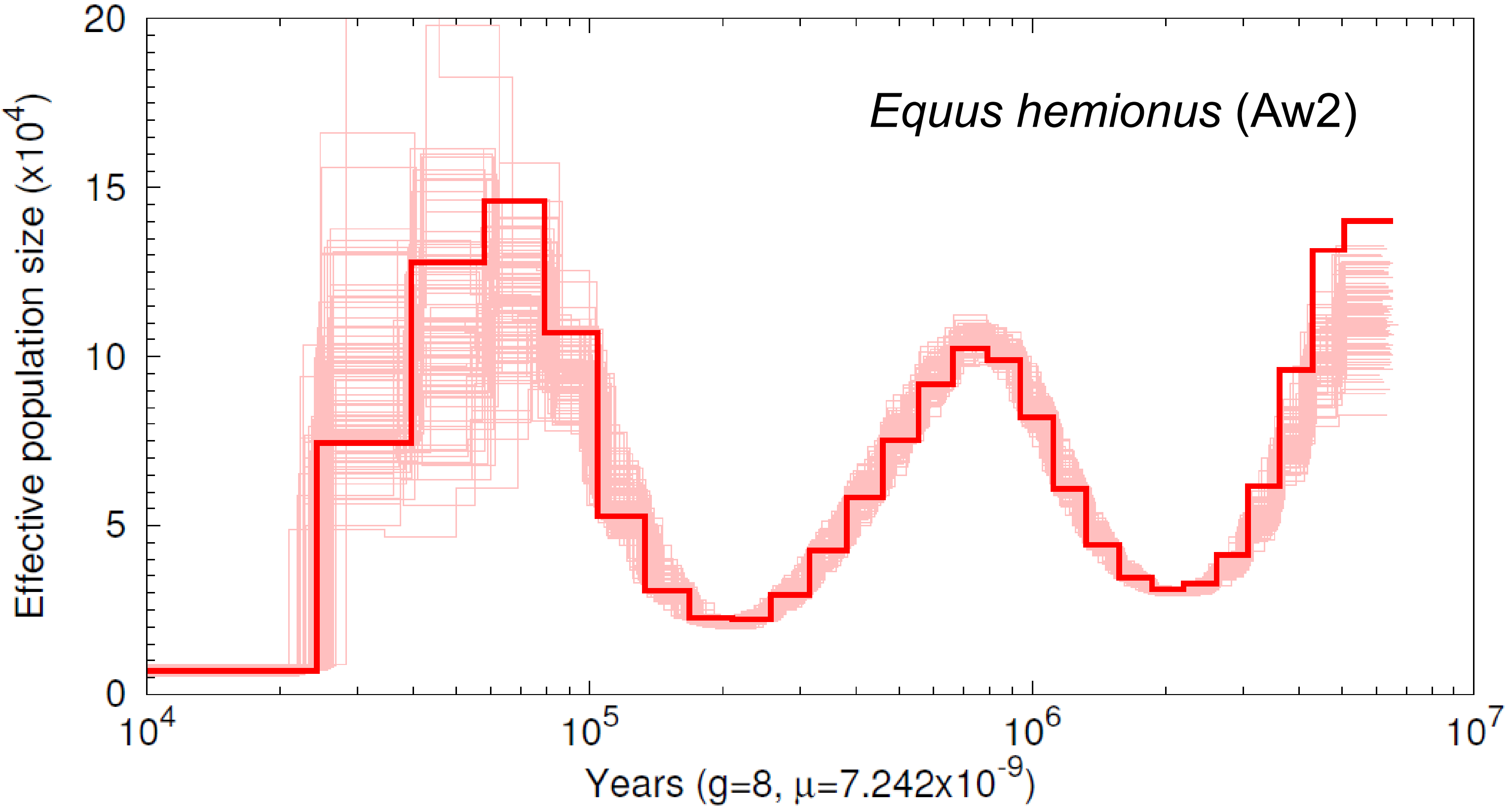
Equus hemionus (Aw2)
Effective population size (x10^4)
20
15
10
5
0
10^4
10^5
10^6
10^7
Years (g=8, μ=7.242x10^-9)

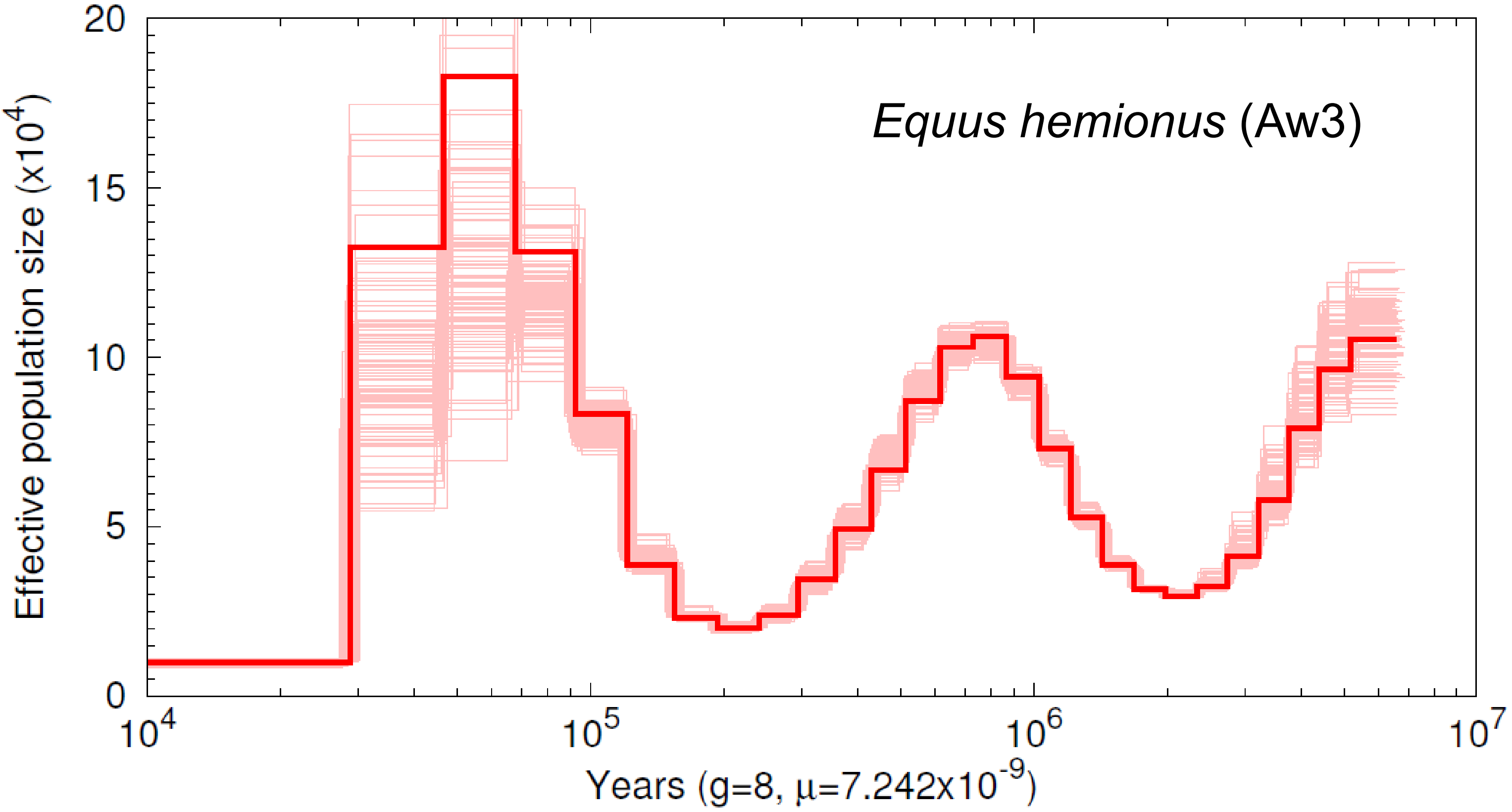
Equus hemionus (Aw3)
Effective population size (x10^4)
Years (g=8, μ=7.242x10^-9)
0
5
10
15
20
10^4
10^5
10^6
10^7

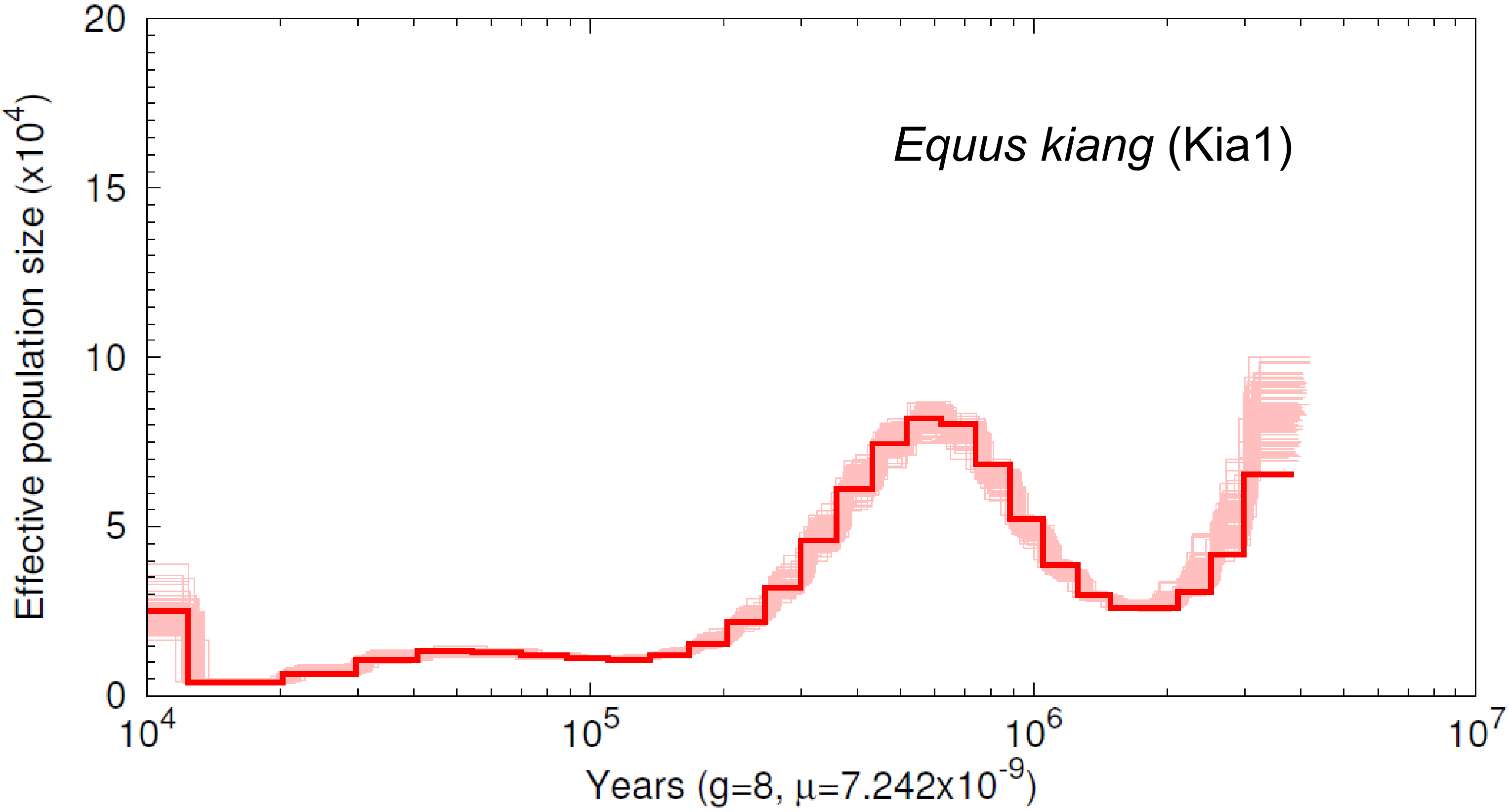
Equus kiang (Kia1)
Effective population size (x10^4)
Years (g=8, μ=7.242x10^-9)
0
5
10
15
20
10^4
10^5
10^6
10^7

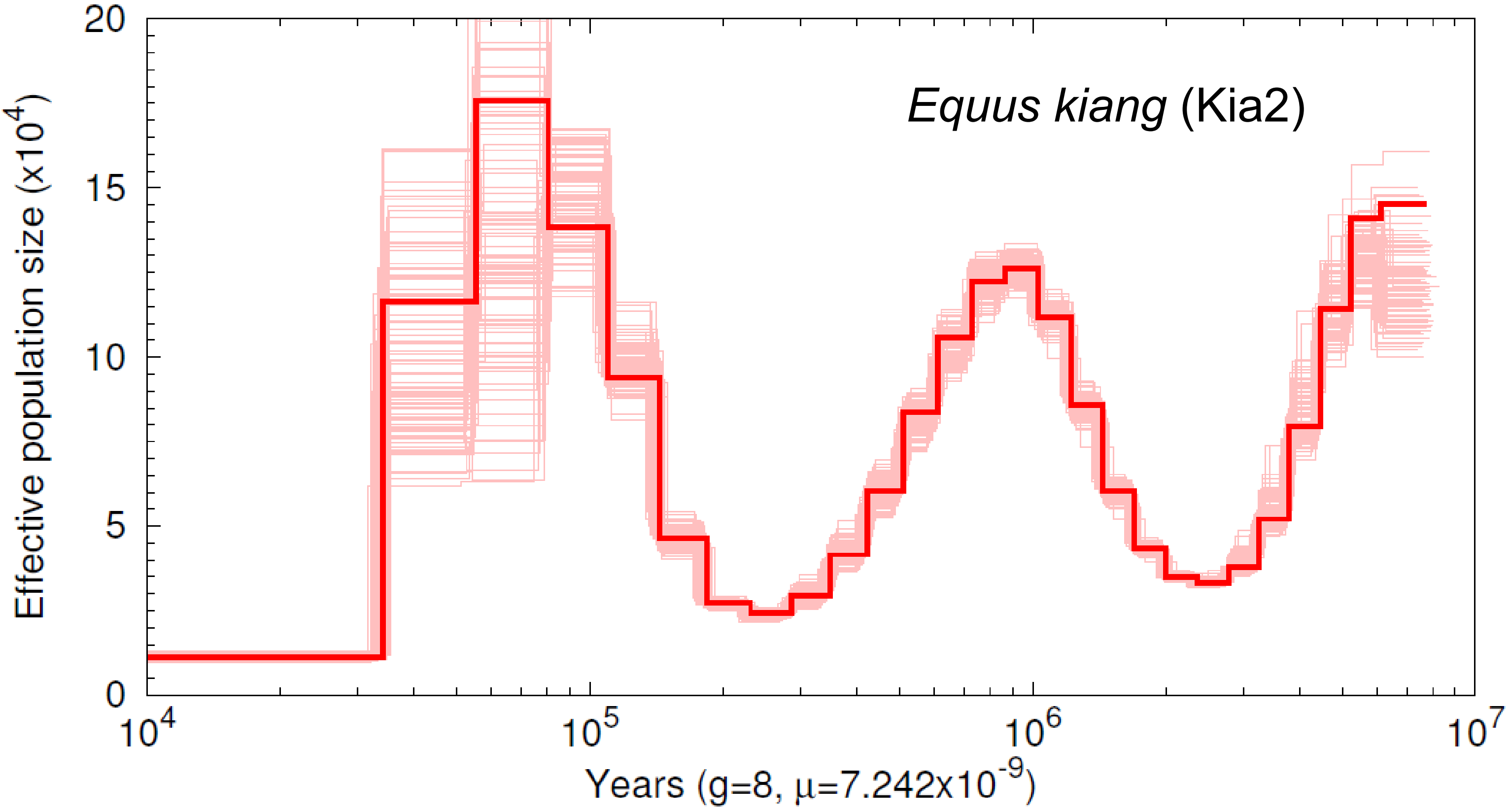
Equus kiang (Kia2)
Effective population size (x10^4)
20
15
10
5
0
10^4
10^5
10^6
10^7
Years (g=8, μ=7.242x10^-9)

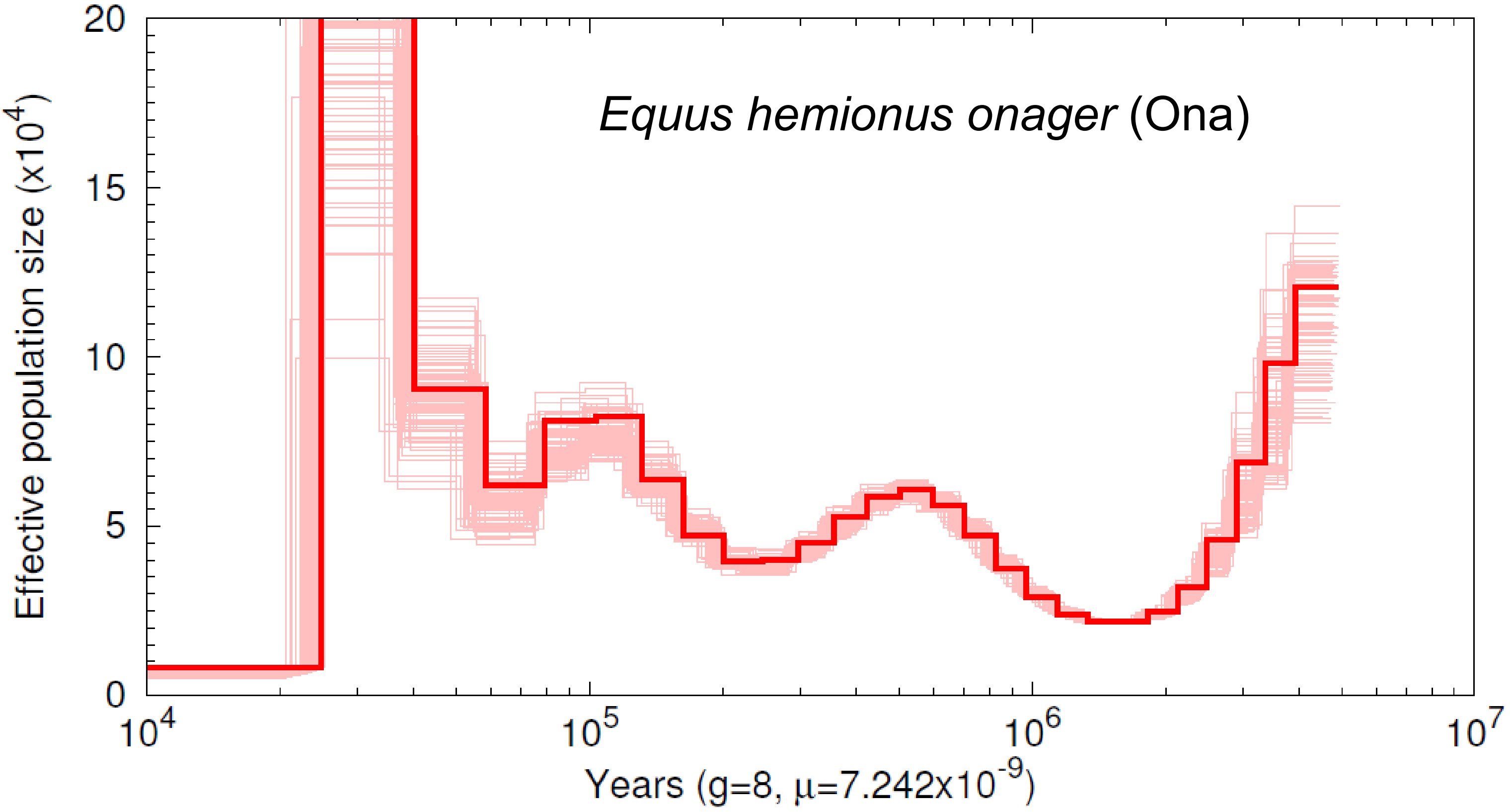
Equus hemionus onager (Ona)
Effective population size (x10^4)
20
15
10
5
0
10^4
10^5
10^6
10^7
Years (g=8, μ=7.242x10^-9)

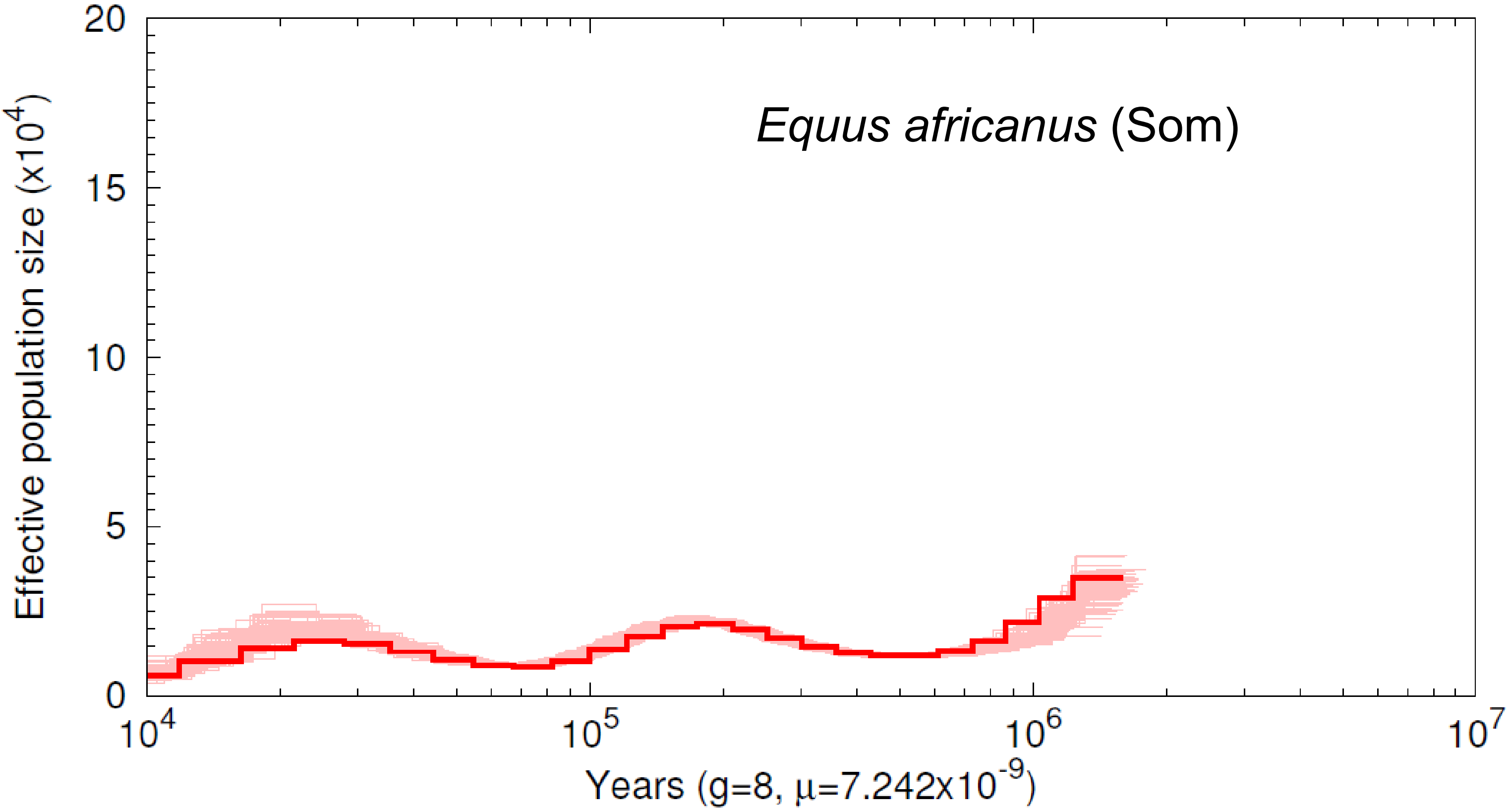

Equus africanus (Som)
Effective population size (x10^4)
20
15
10
5
0
10^4
10^5
10^6
10^7
Years (g=8, μ=7.242x10^-9)


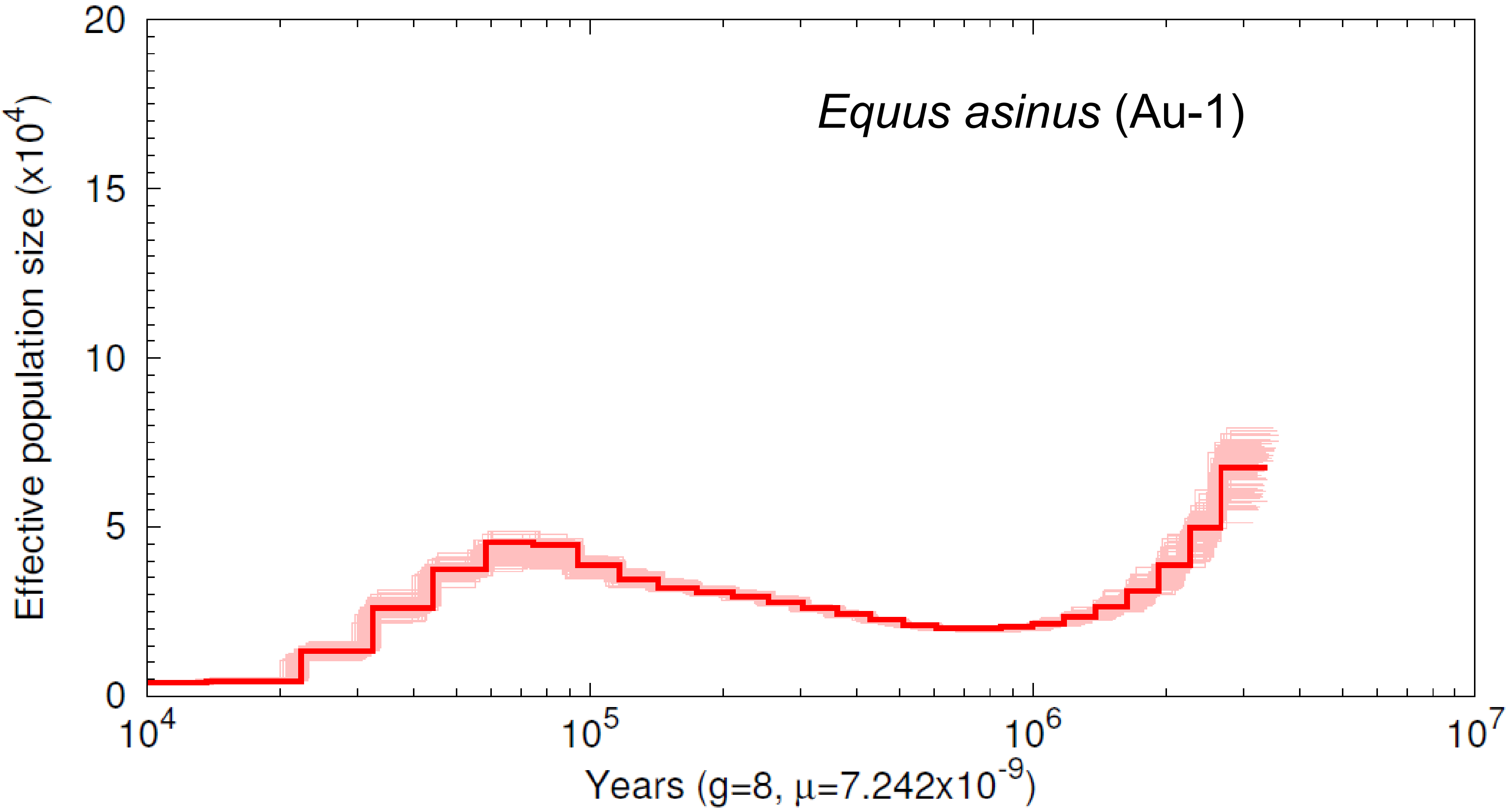

Equus asinus (Au-1)
Effective population size (x10^4)
20
15
10
5
0
10^4
10^5
10^6
10^7
Years (g=8, μ=7.242x10^-9)

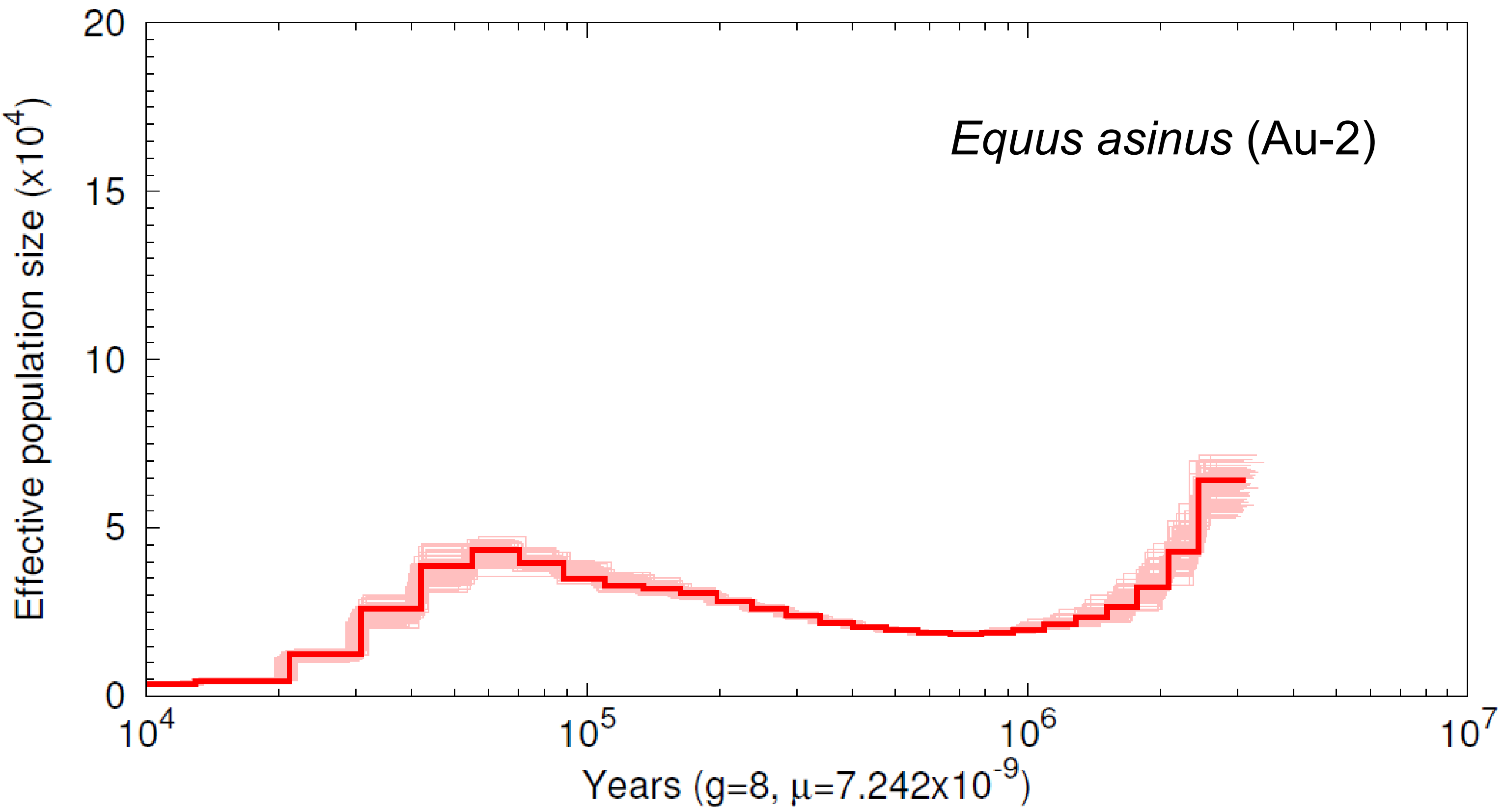
Equus asinus (Au-2)
Effective population size (x10^4)
Years (g=8, μ=7.242x10^-9)
20
15
10
5
0
10^4
10^5
10^6
10^7

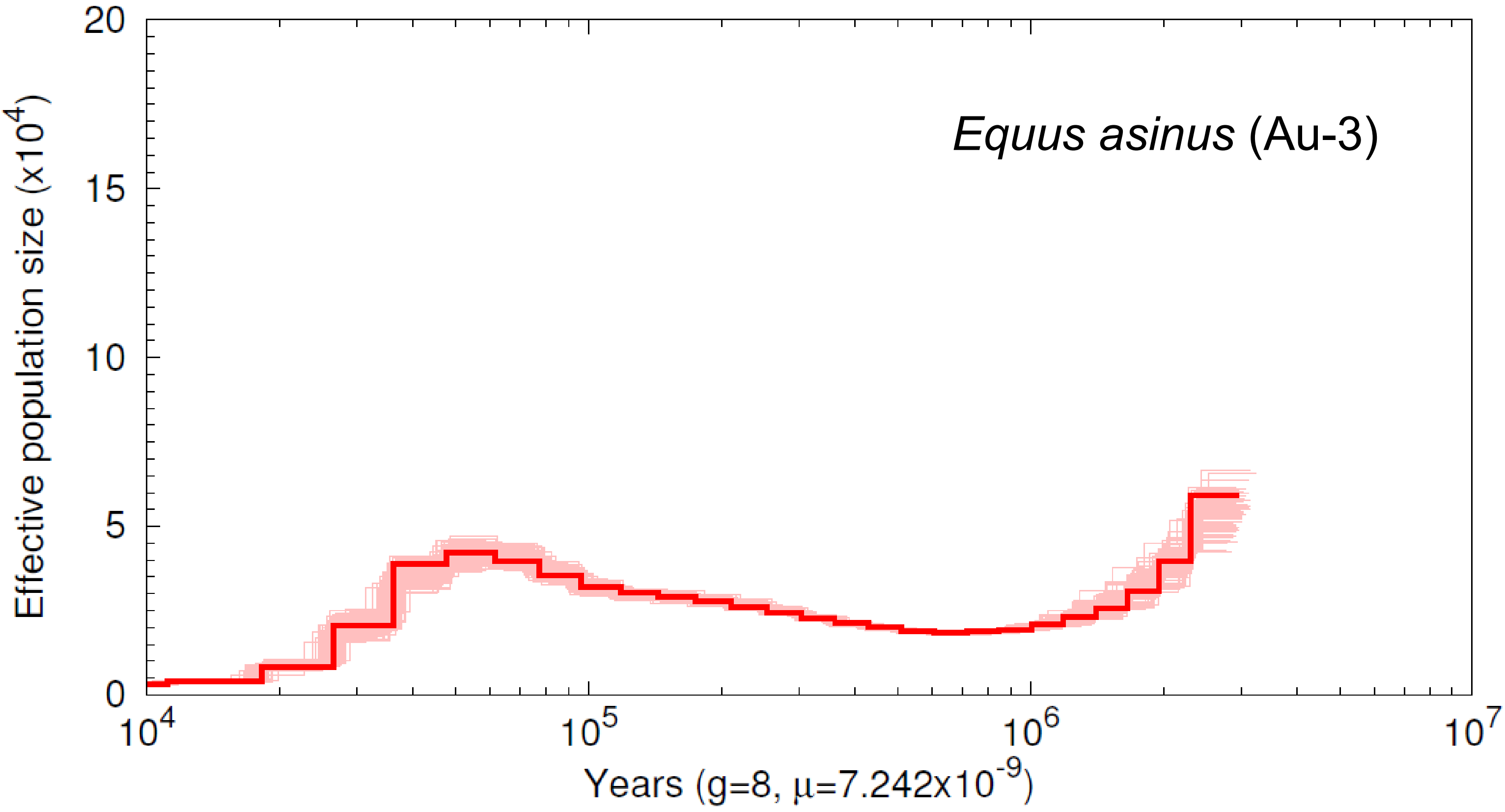
Equus asinus (Au-3)
Effective population size (x10^4)
Years (g=8, μ=7.242x10^-9)
20
15
10
5
0
10^4
10^5
10^6
10^7

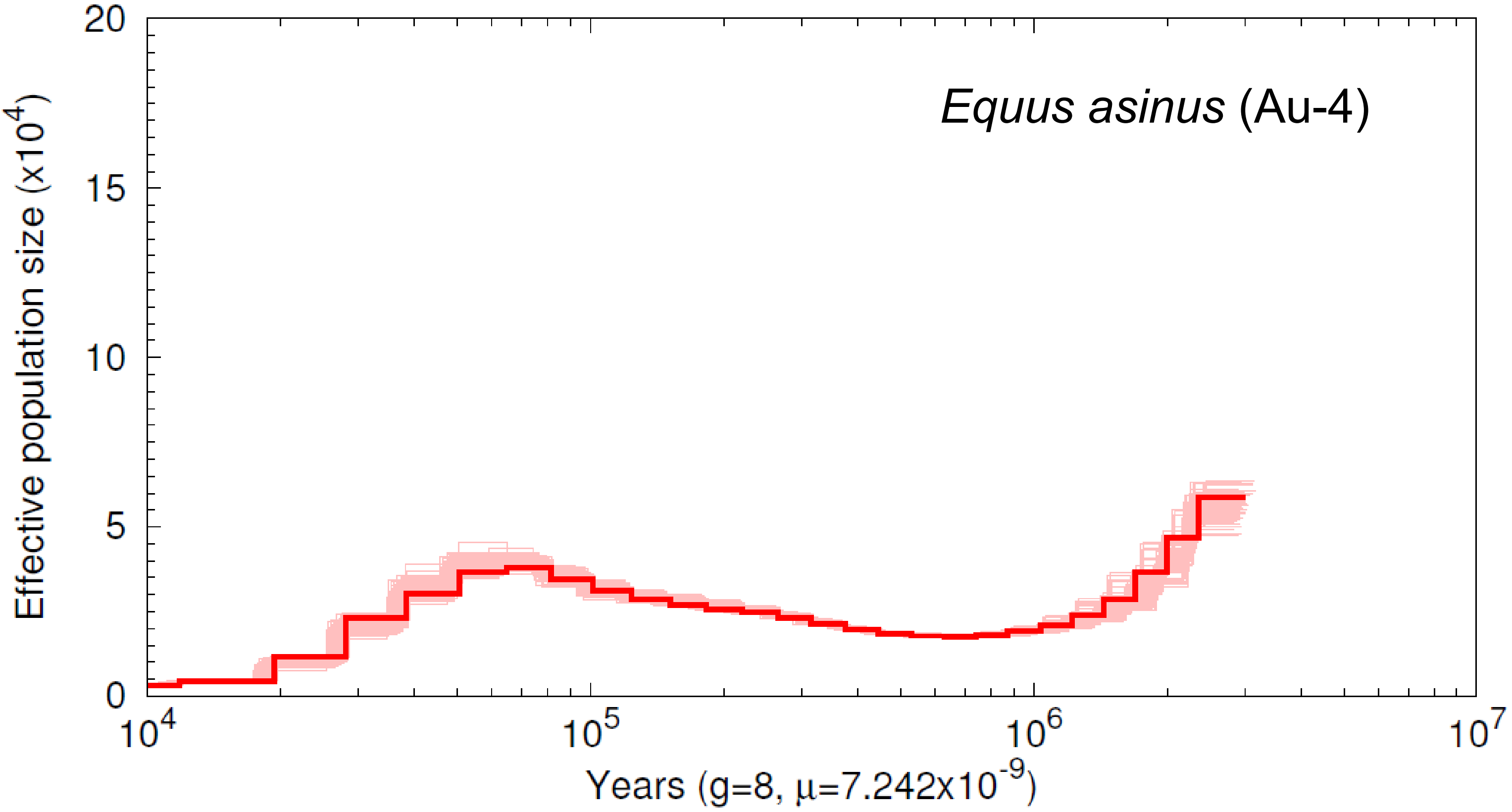
Equus asinus (Au-4)
Effective population size (x10^4)
20
15
10
5
0
10^4
10^5
10^6
10^7
Years (g=8, μ=7.242x10^-9)

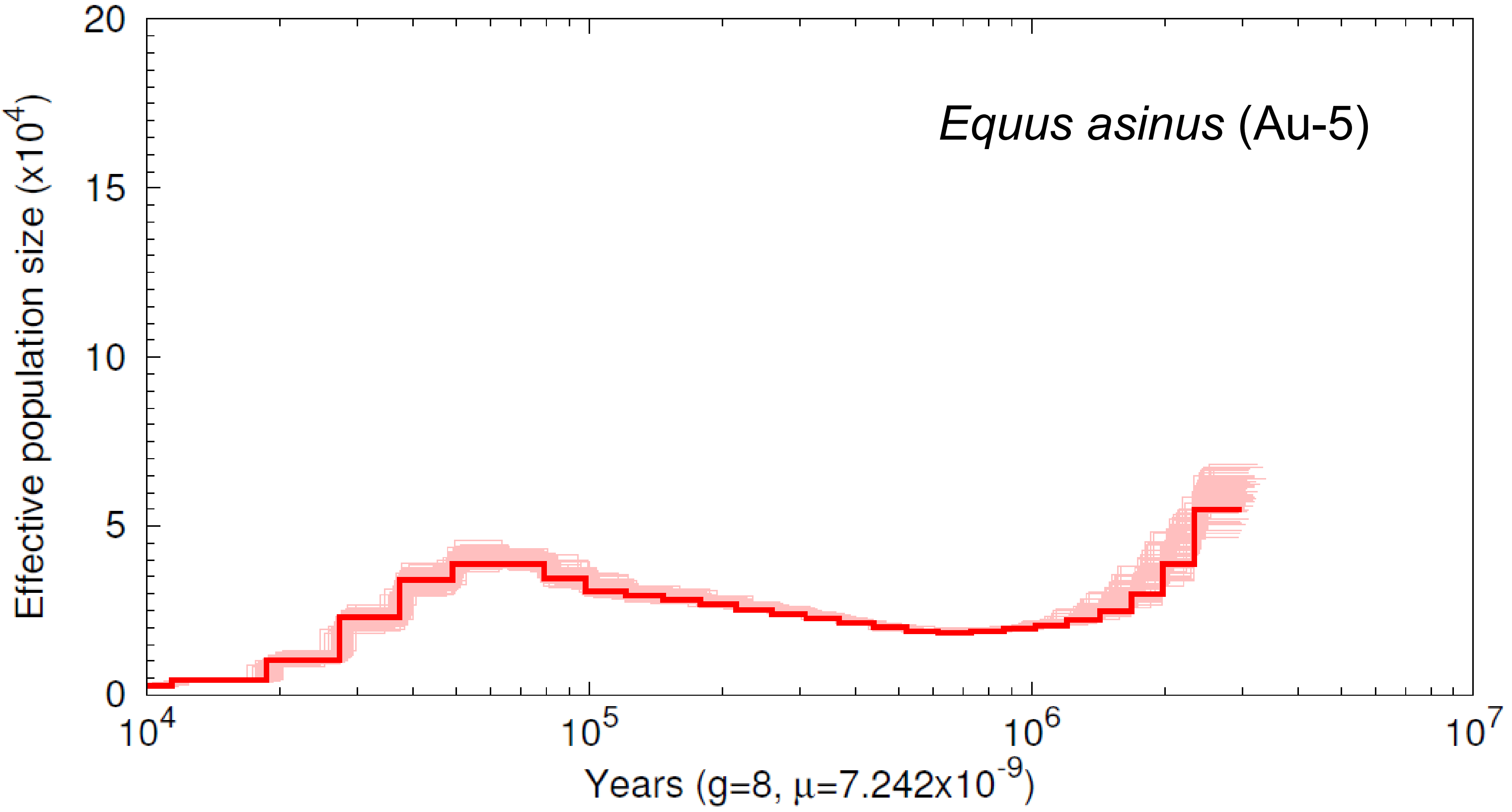
Equus asinus (Au-5)
Effective population size (x10^4)
20
15
10
5
0
10^4
10^5
10^6
10^7
Years (g=8, μ=7.242x10^-9)

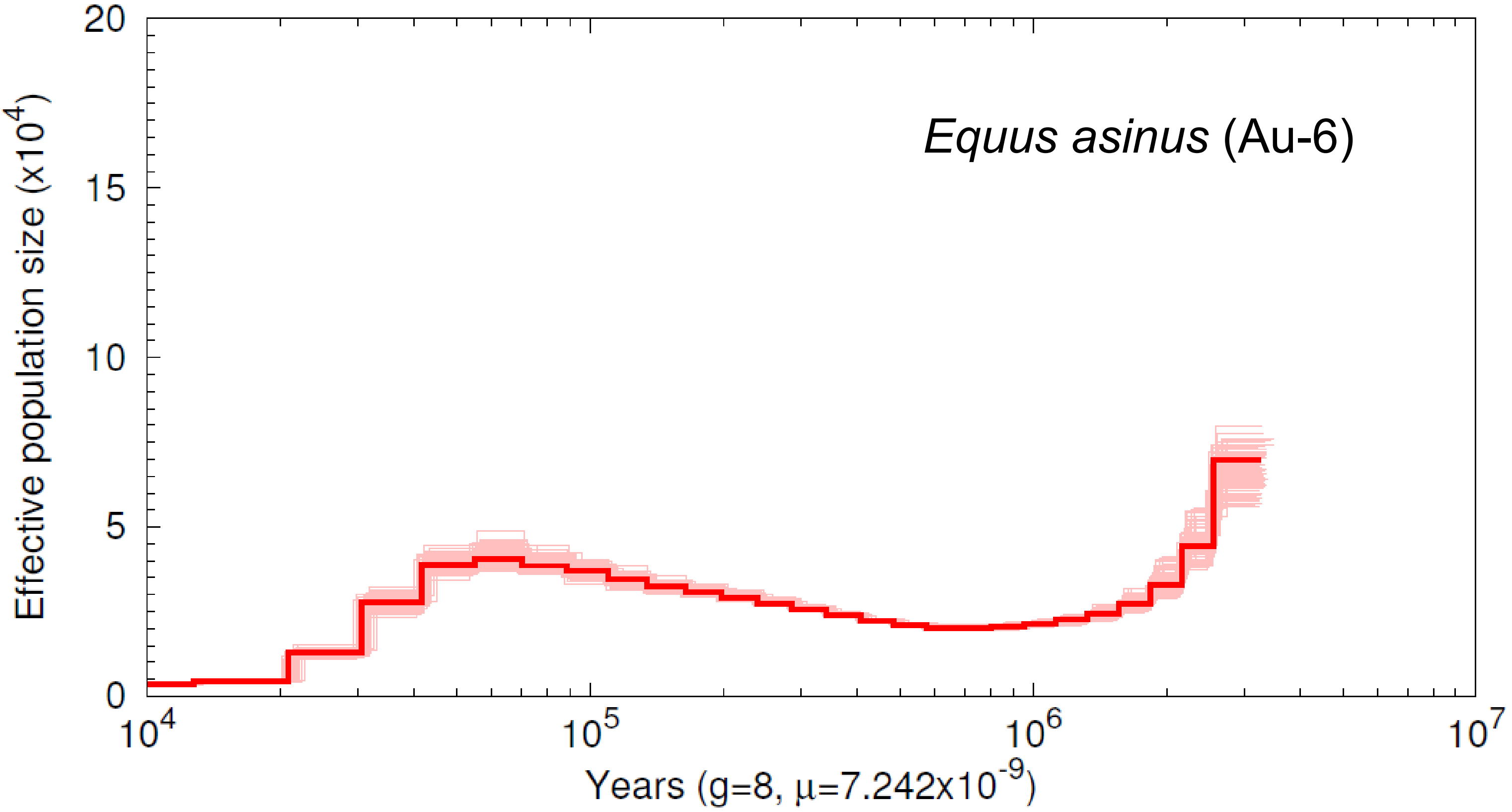
Equus asinus (Au-6)
Effective population size (x10^4)
20
15
10
5
0
10^4
10^5
10^6
10^7
Years (g=8, μ=7.242x10^-9)

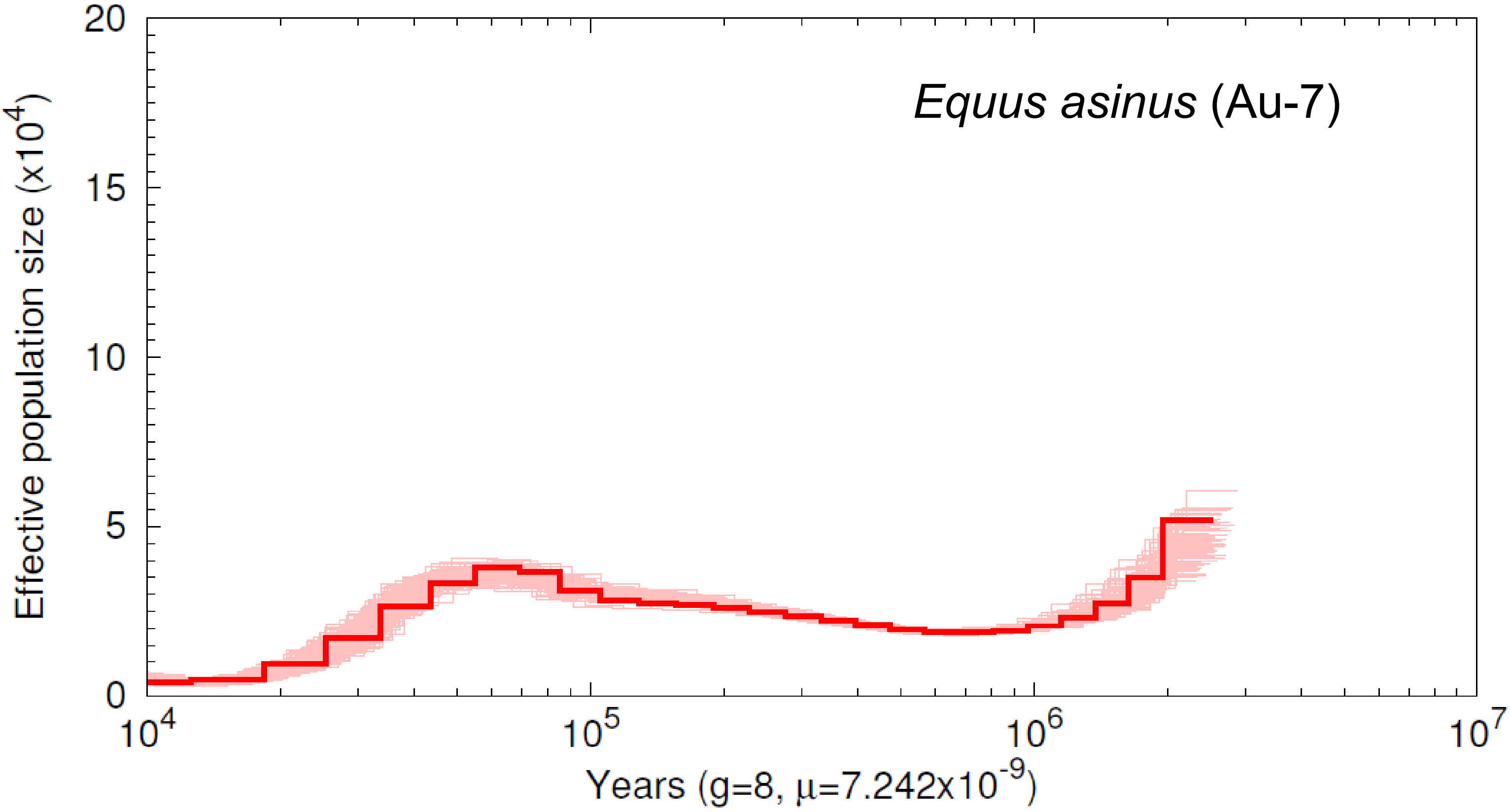
Equus asinus (Au-7)
Effective population size (x10^4)
20
15
10
5
0
10^4
10^5
10^6
10^7
Years (g=8, μ=7.242x10^-9)

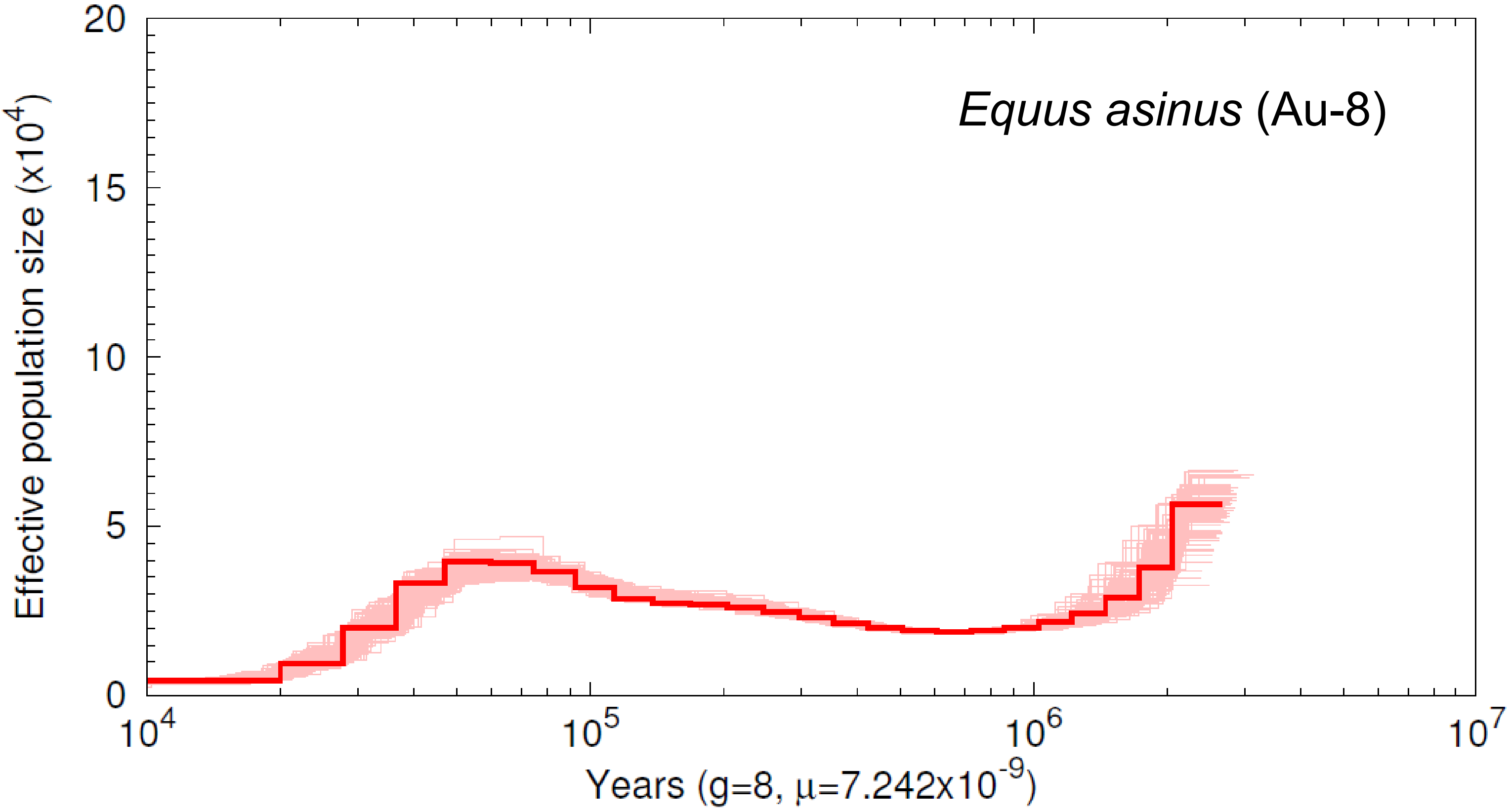

Equus asinus (Au-8)
Effective population size (x10^4)
20
15
10
5
0
10^4
10^5
10^6
10^7
Years (g=8, μ=7.242x10^-9)


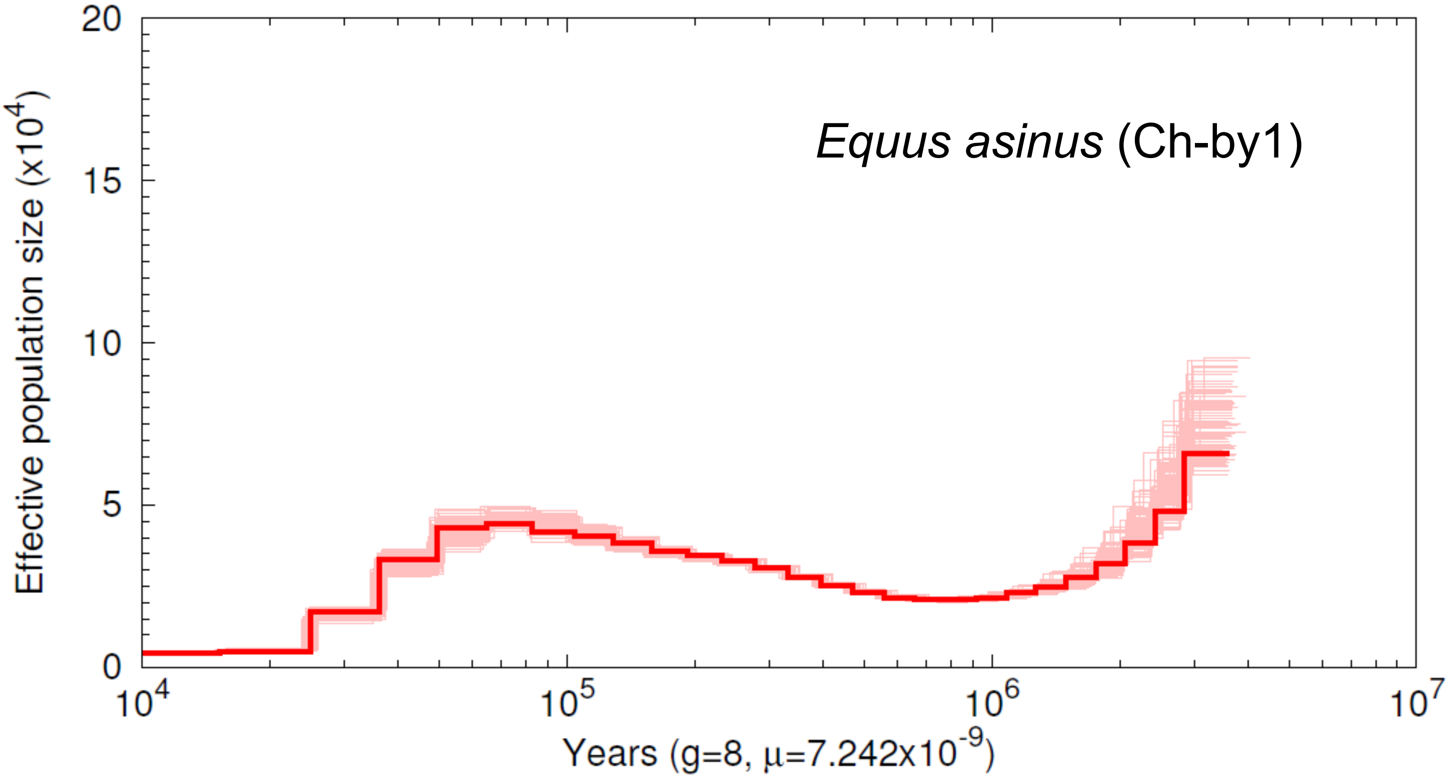

Equus asinus (Ch-by1)
Effective population size (x10^4)
20
15
10
5
0
10^4
10^5
10^6
10^7
Years (g=8, μ=7.242x10^-9)

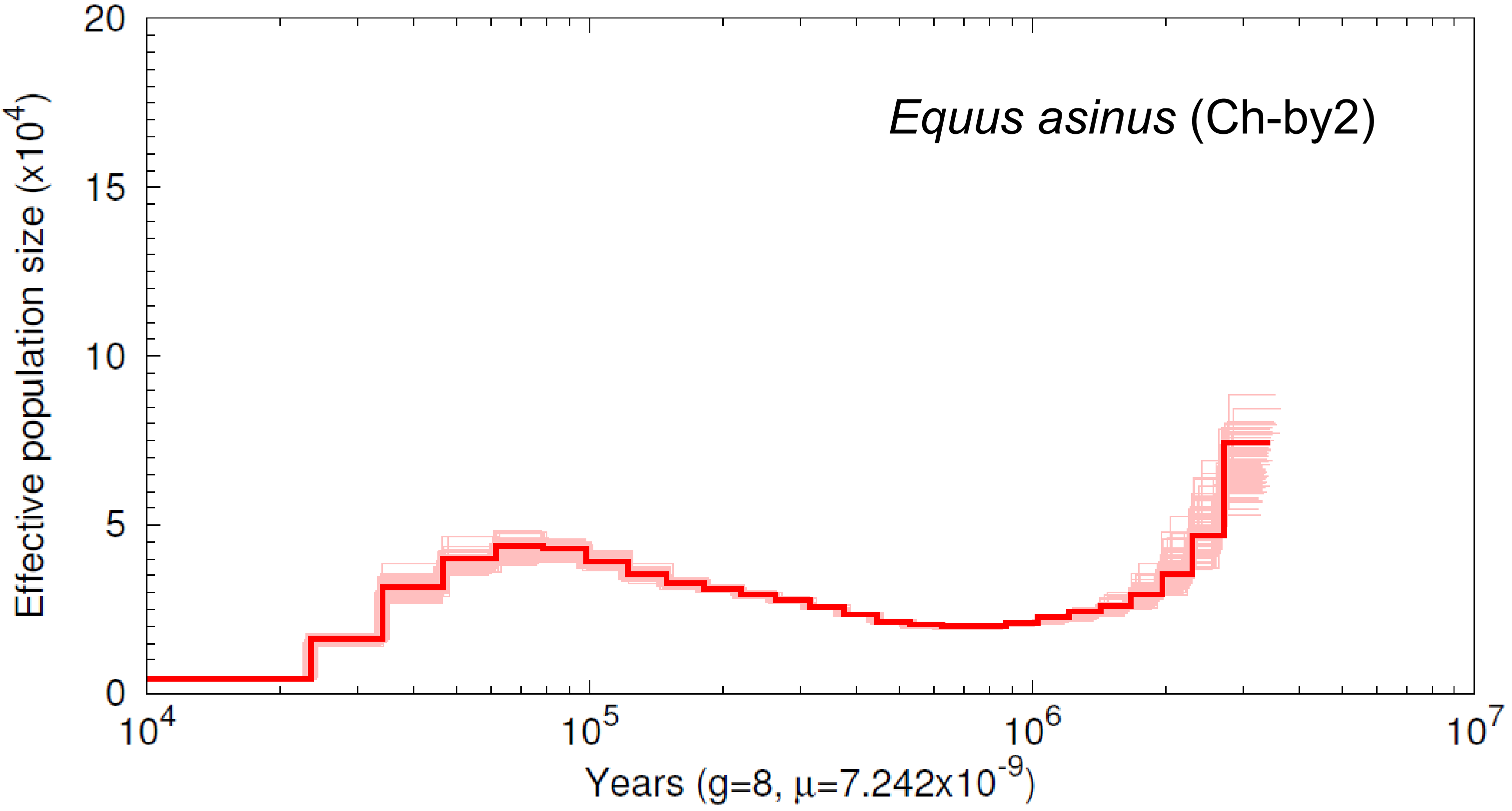
Equus asinus (Ch-by2)
Effective population size (x10^4)
Years (g=8, μ=7.242x10^-9)
20
15
10
5
0
10^4
10^5
10^6
10^7

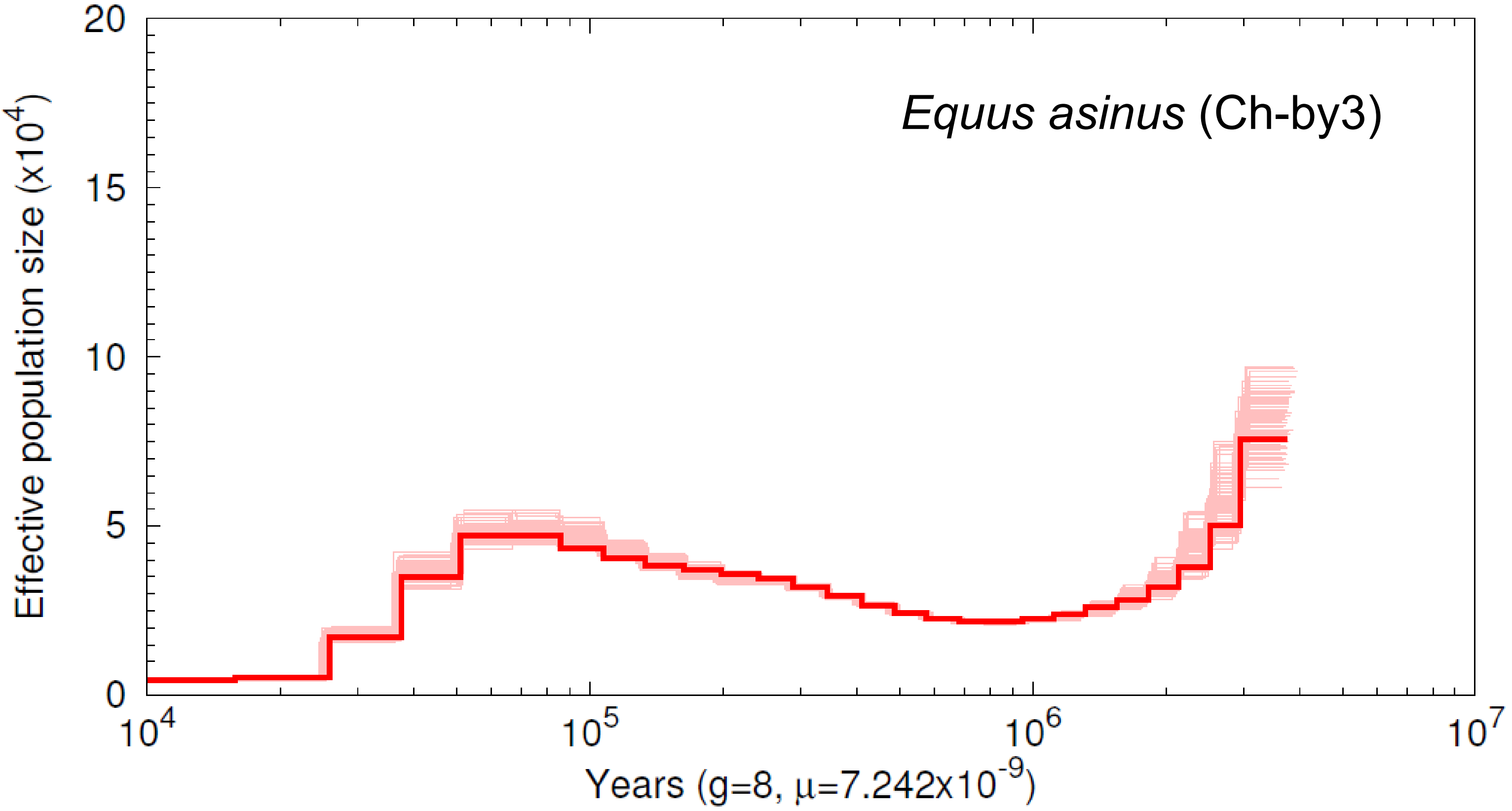
Equus asinus (Ch-by3)
Effective population size (x10^4)
Years (g=8, μ=7.242x10^-9)
20
15
10
5
0
10^4
10^5
10^6
10^7

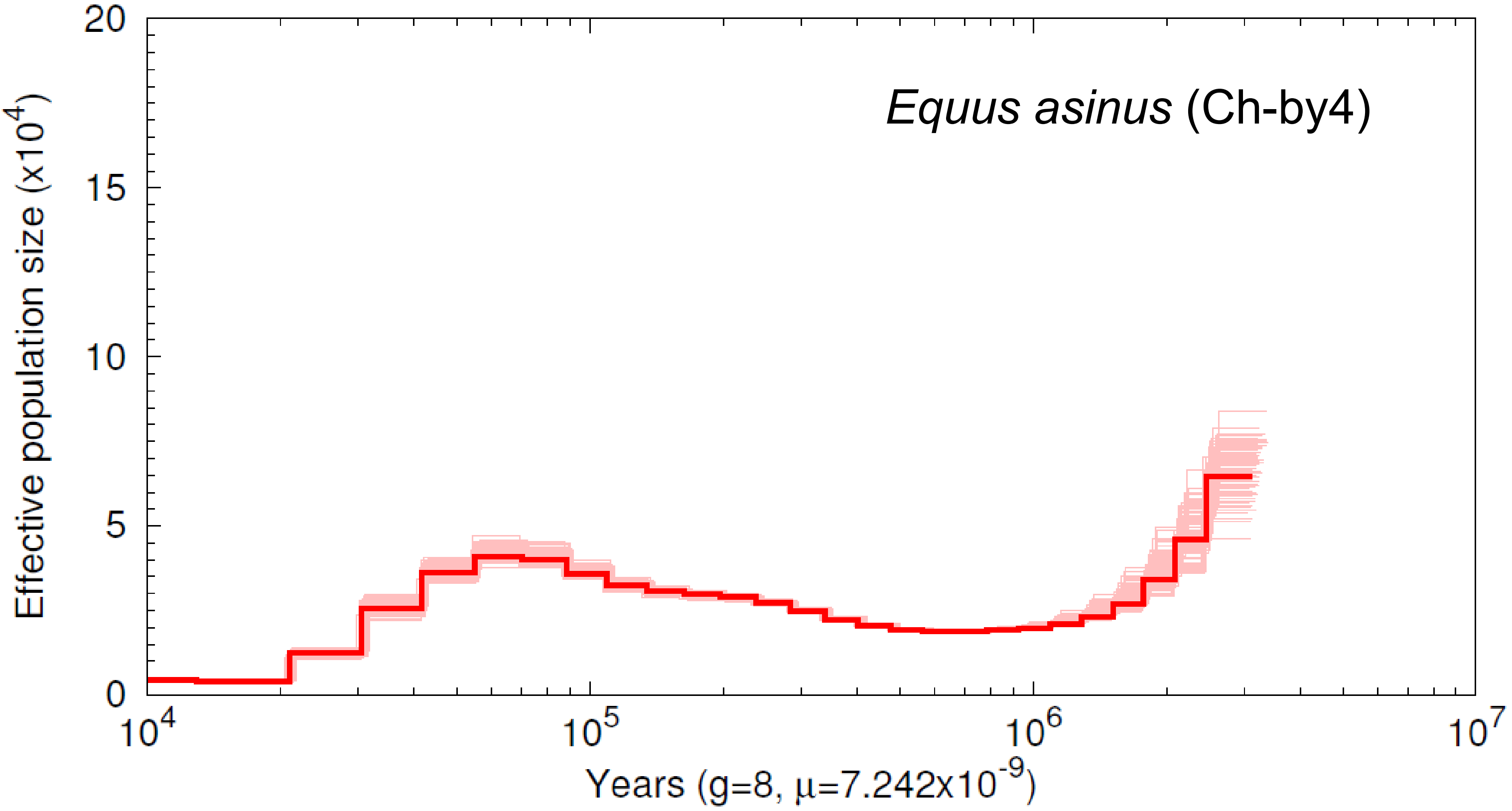
Equus asinus (Ch-by4)
Effective population size (x10^4)
0
5
10
15
20
10^4
10^5
10^6
10^7
Years (g=8, μ=7.242x10^-9)

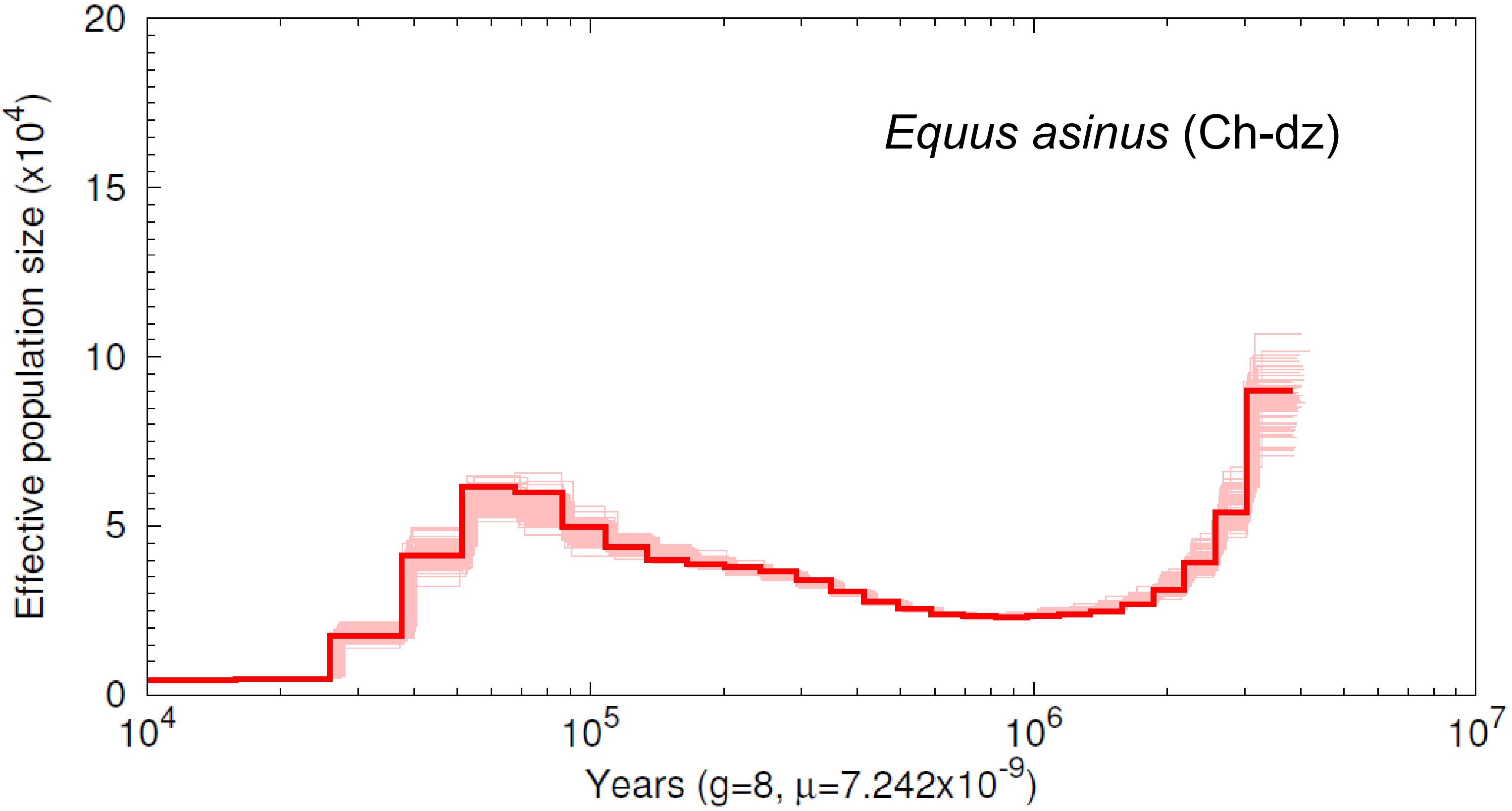
Equus asinus (Ch-dz)
Effective population size (x10^4)
0
5
10
15
20
10^4
10^5
10^6
10^7
Years (g=8, μ=7.242x10^-9)

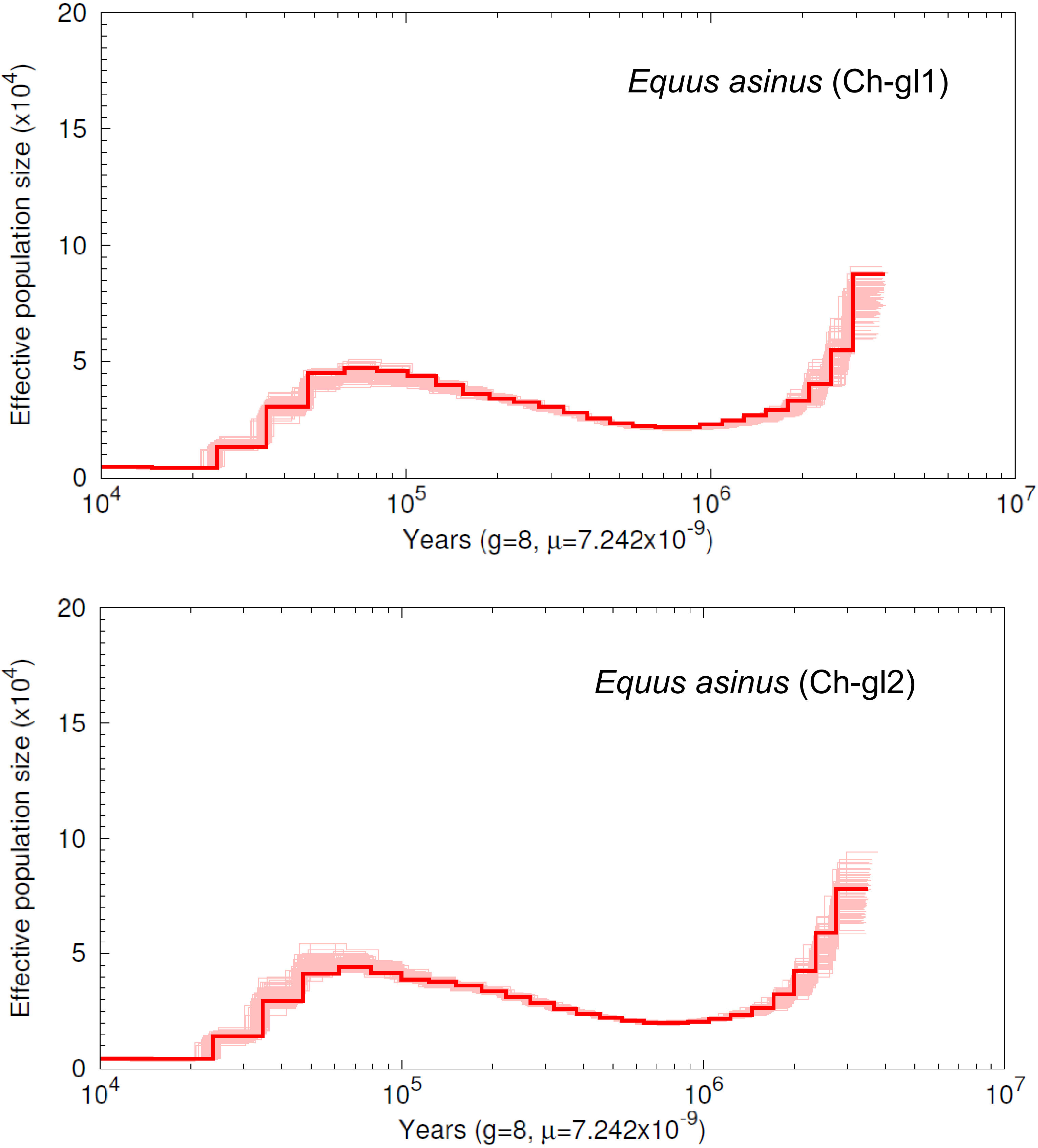

Equus asinus (Ch-gl1)
Effective population size (x10^4)
Years (g=8, μ=7.242x10^-9)
20
15
10
5
0
10^4
10^5
10^6
10^7
Equus asinus (Ch-gl2)
Effective population size (x10^4)
Years (g=8, μ=7.242x10^-9)
20
15
10
5
0
10^4
10^5
10^6
10^7

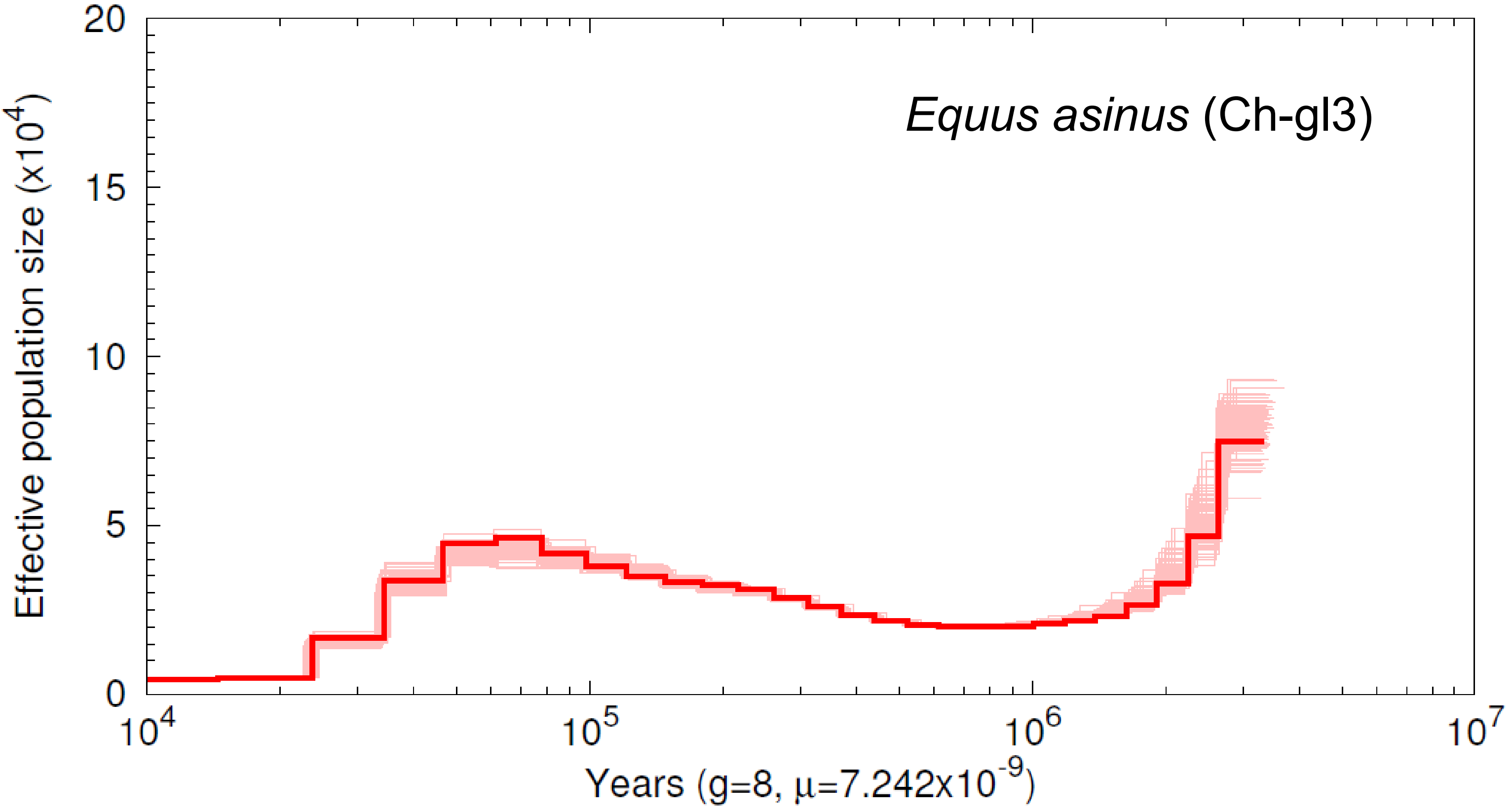

Equus asinus (Ch-gl3)
Effective population size (x10^4)
0
5
10
15
20
10^4
10^5
10^6
10^7
Years (g=8, μ=7.242x10^-9)


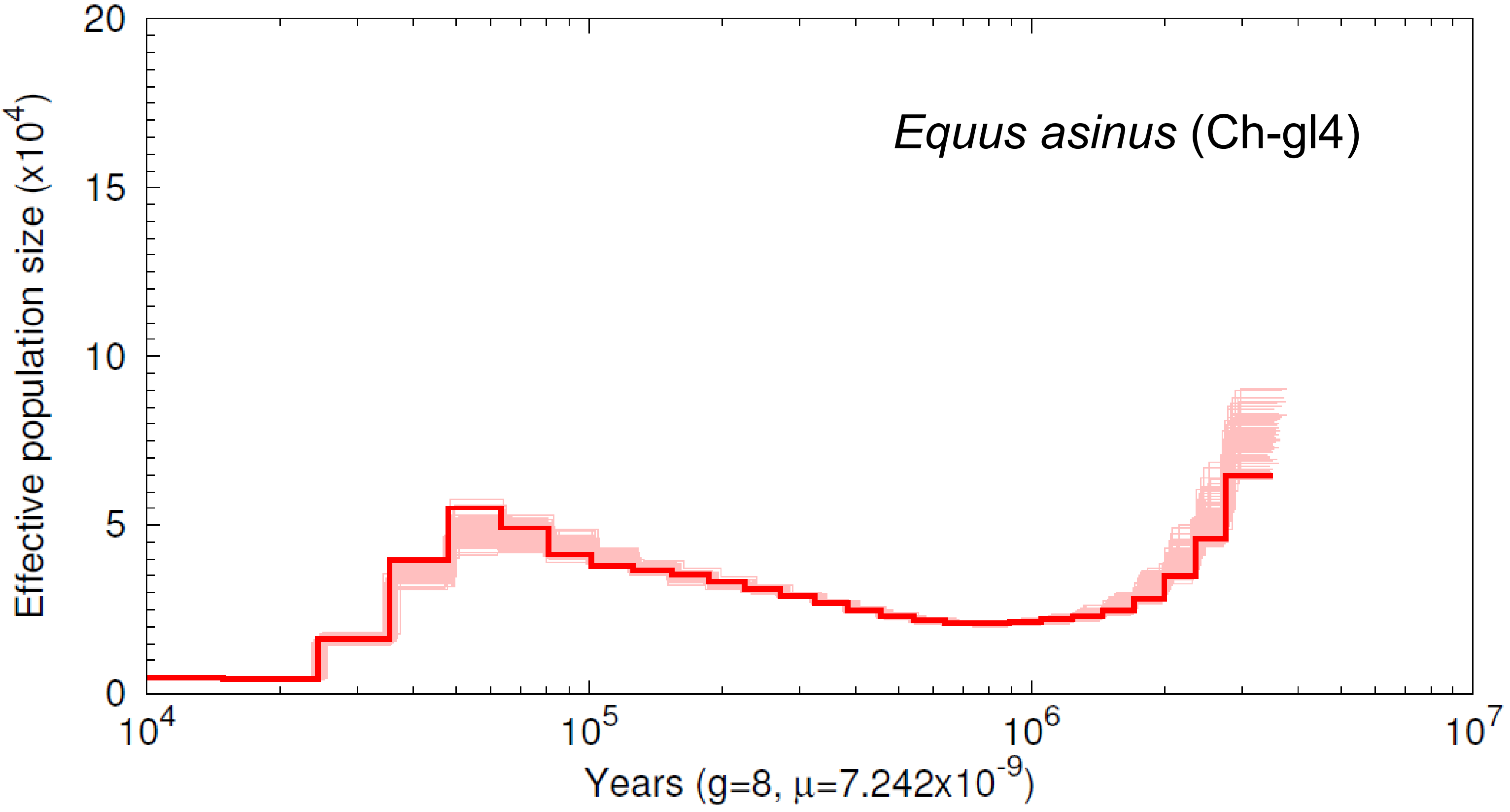

Equus asinus (Ch-gl4)
Effective population size (x10^4)
0
5
10
15
20
10^4
10^5
10^6
10^7
Years (g=8, μ=7.242x10^-9)

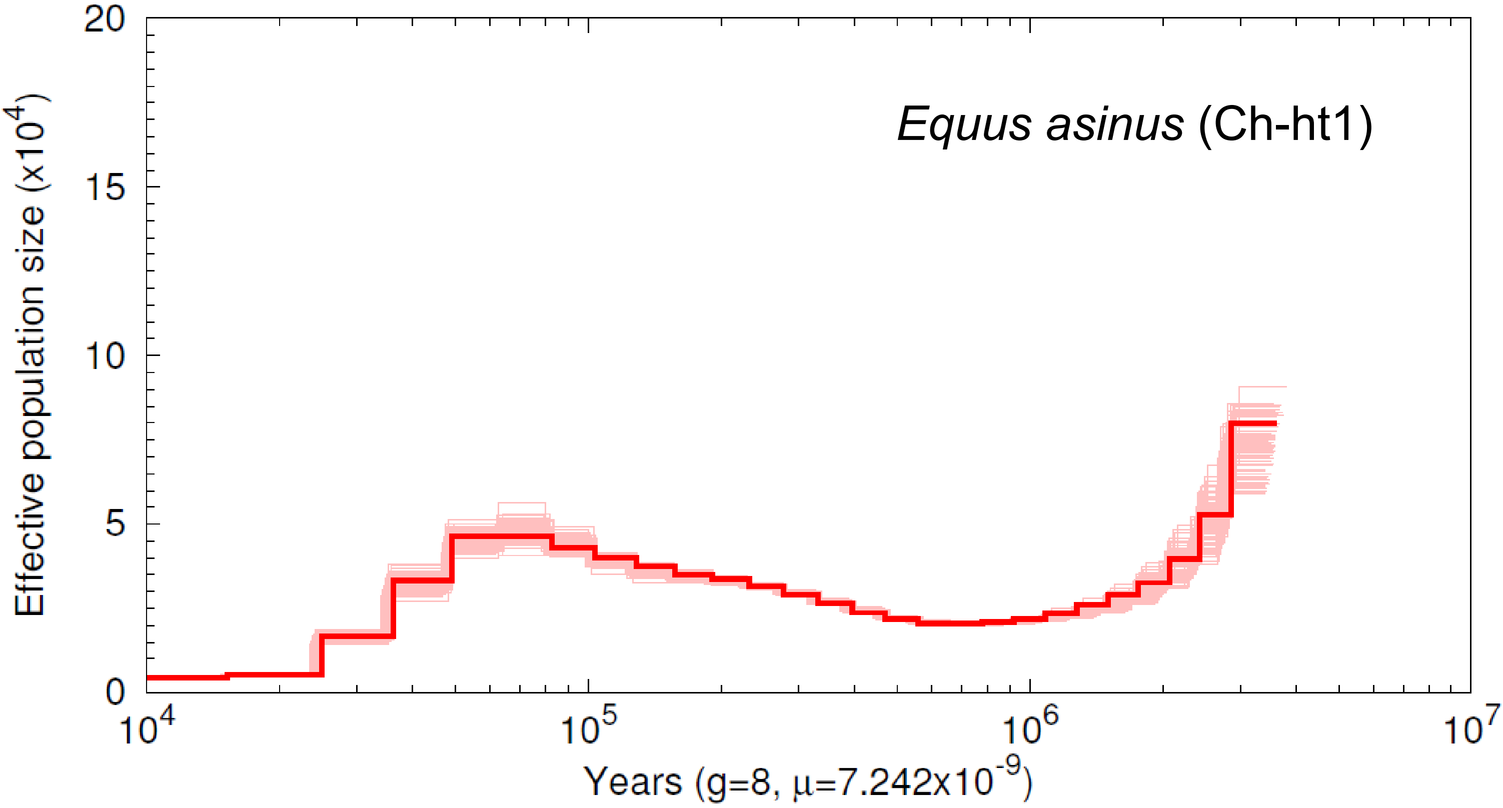
Equus asinus (Ch-ht1)
Effective population size (x10^4)
20
15
10
5
0
10^4
10^5
10^6
10^7
Years (g=8, μ=7.242x10^-9)

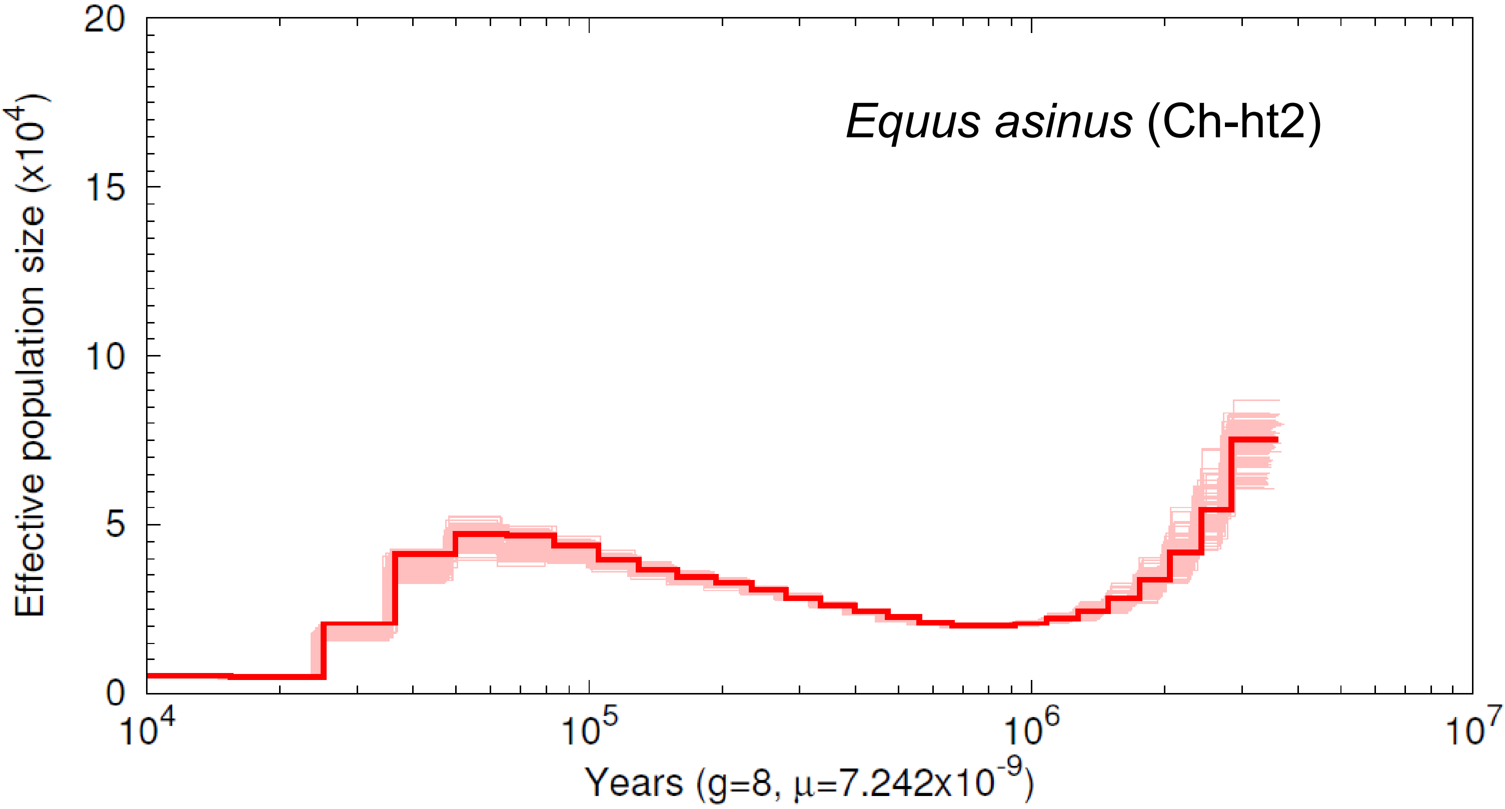
Equus asinus (Ch-ht2)
Effective population size (x10^4)
20
15
10
5
0
10^4
10^5
10^6
10^7
Years (g=8, μ=7.242x10^-9)

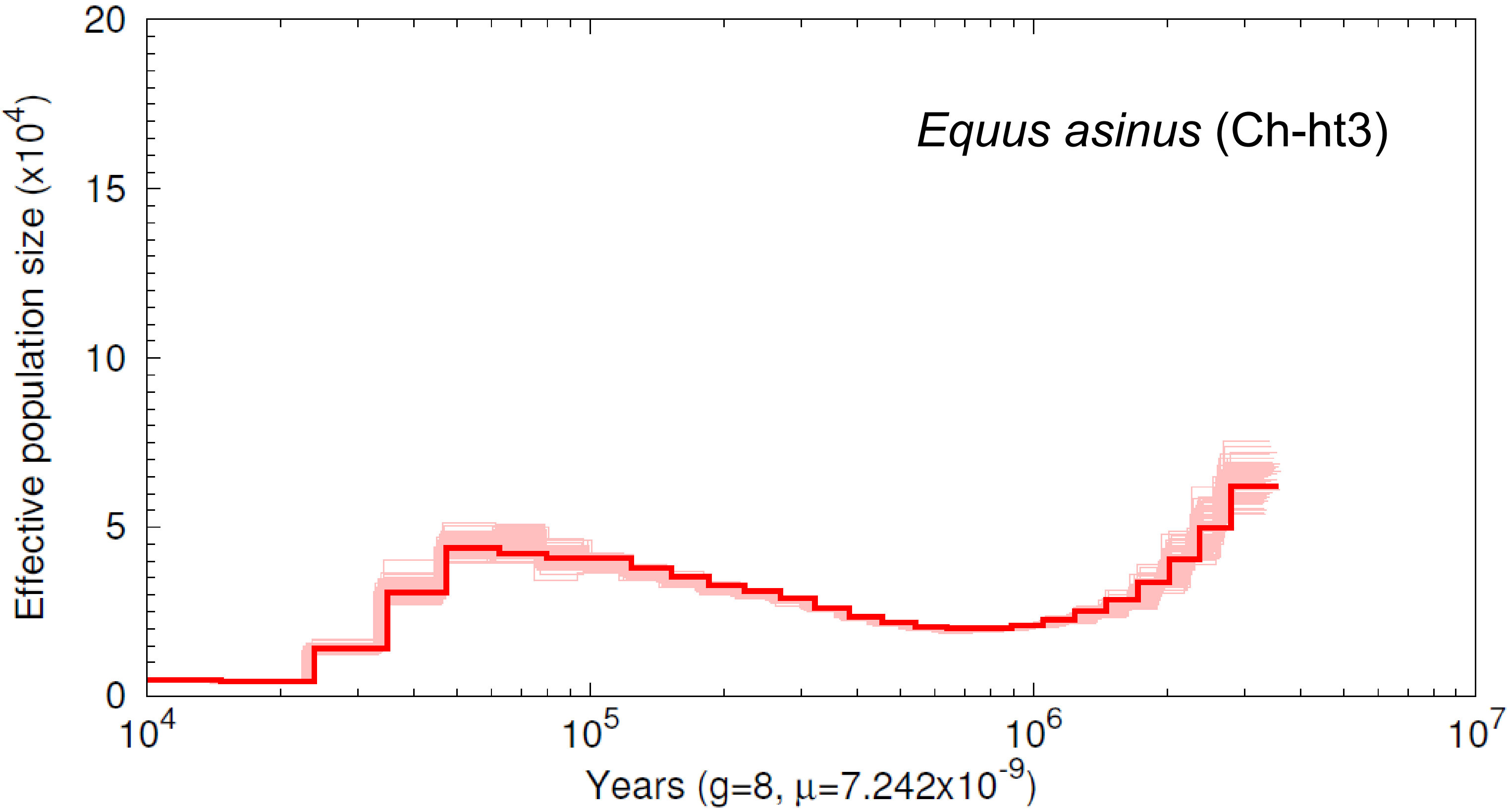

Equus asinus (Ch-ht3)
Effective population size (x10^4)
20
15
10
5
0
10^4
10^5
10^6
10^7
Years (g=8, μ=7.242x10^-9)


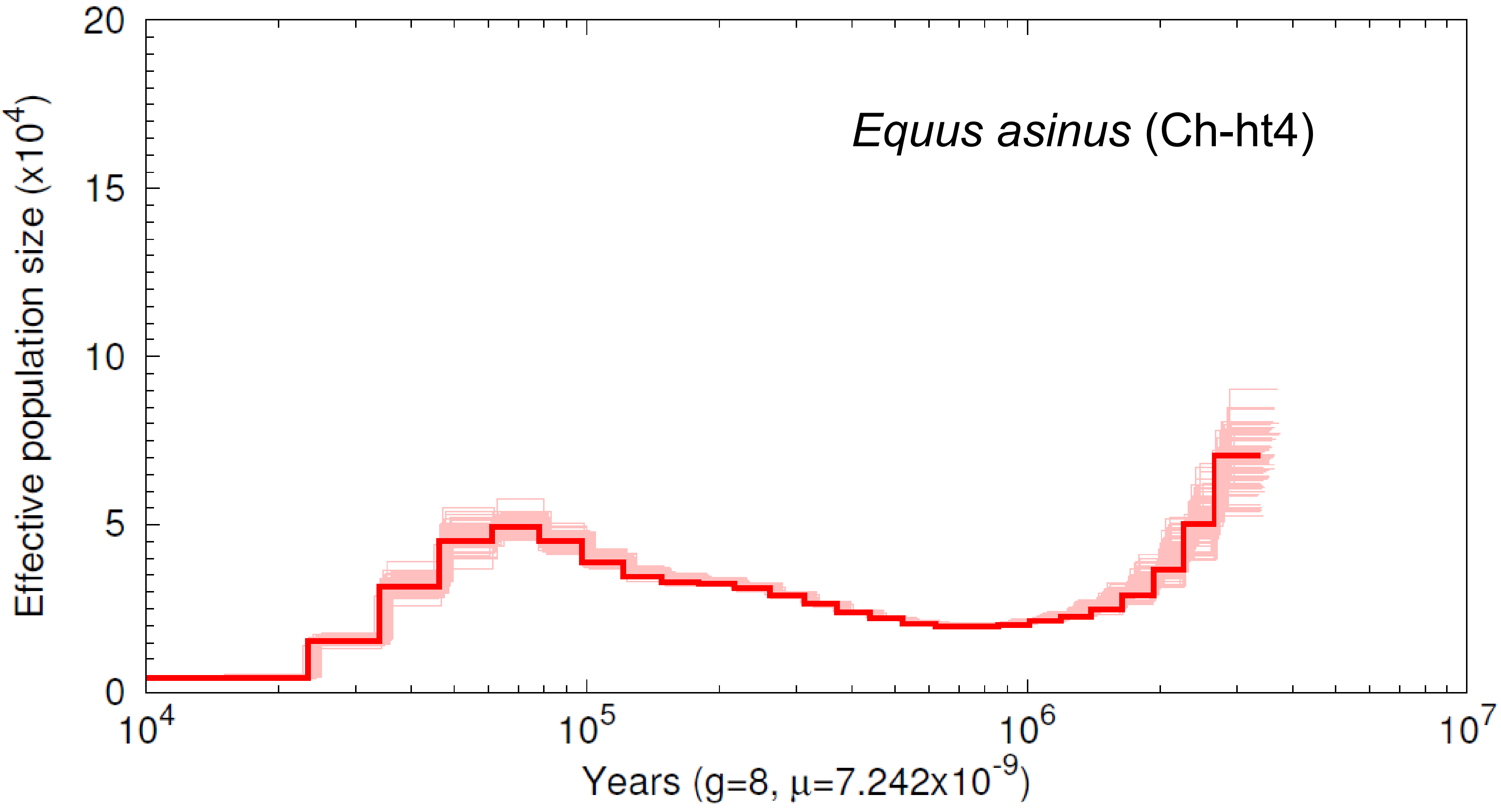

Equus asinus (Ch-ht4)
Effective population size (x10^4)
20
15
10
5
0
10^4
10^5
10^6
10^7
Years (g=8, μ=7.242x10^-9)

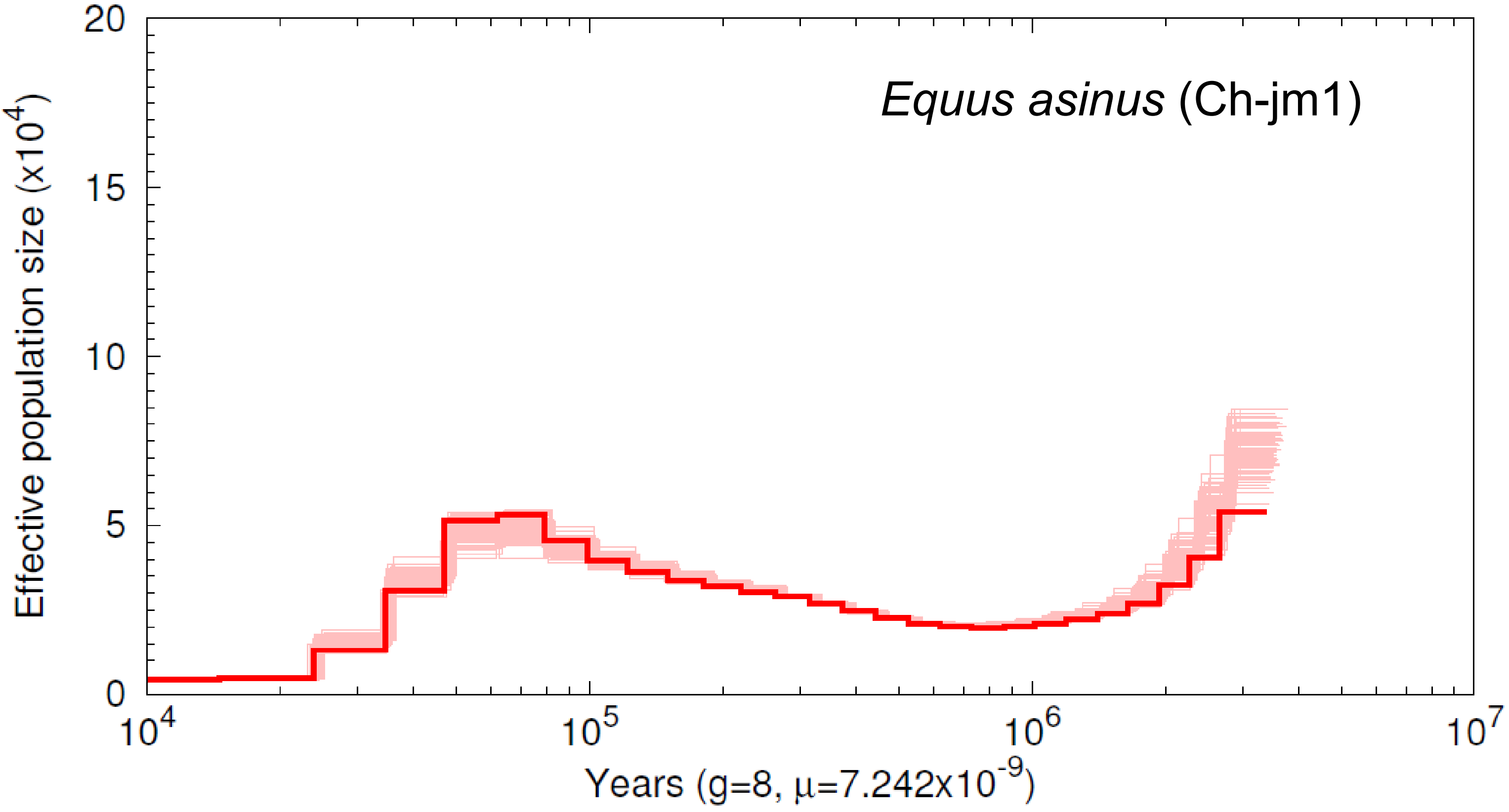
Equus asinus (Ch-jm1)
Effective population size (x10^4)
20
15
10
5
0
10^4
10^5
10^6
10^7
Years (g=8, μ=7.242x10^-9)

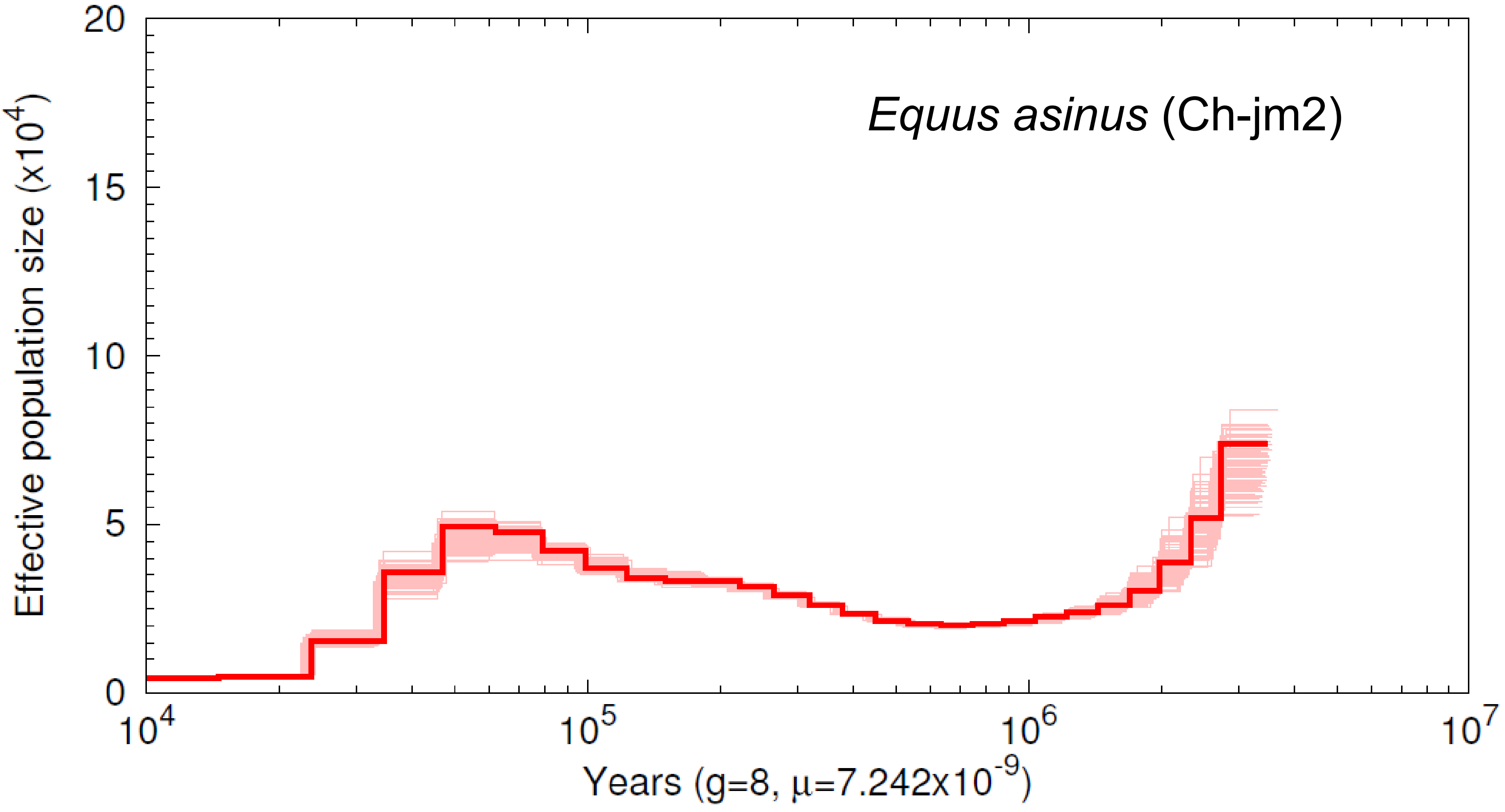
Equus asinus (Ch-jm2)
Effective population size (x10^4)
20
15
10
5
0
10^4
10^5
10^6
10^7
Years (g=8, μ=7.242x10^-9)

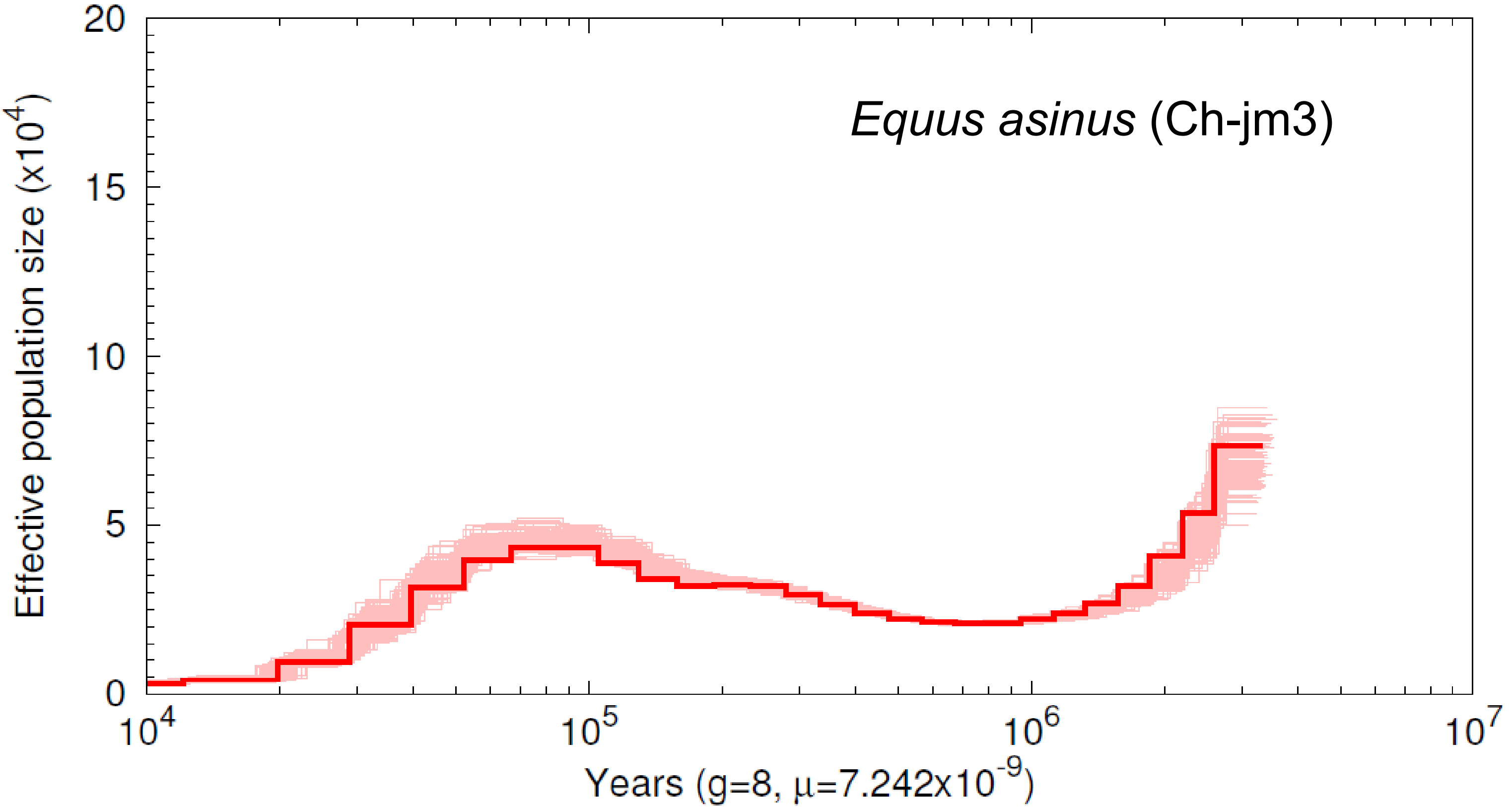

*Equus asinus* (Ch-jm3)
Effective population size (x10^4)
20
15
10
5
0
10^4
10^5
10^6
10^7
Years (g=8, μ=7.242x10^-9)


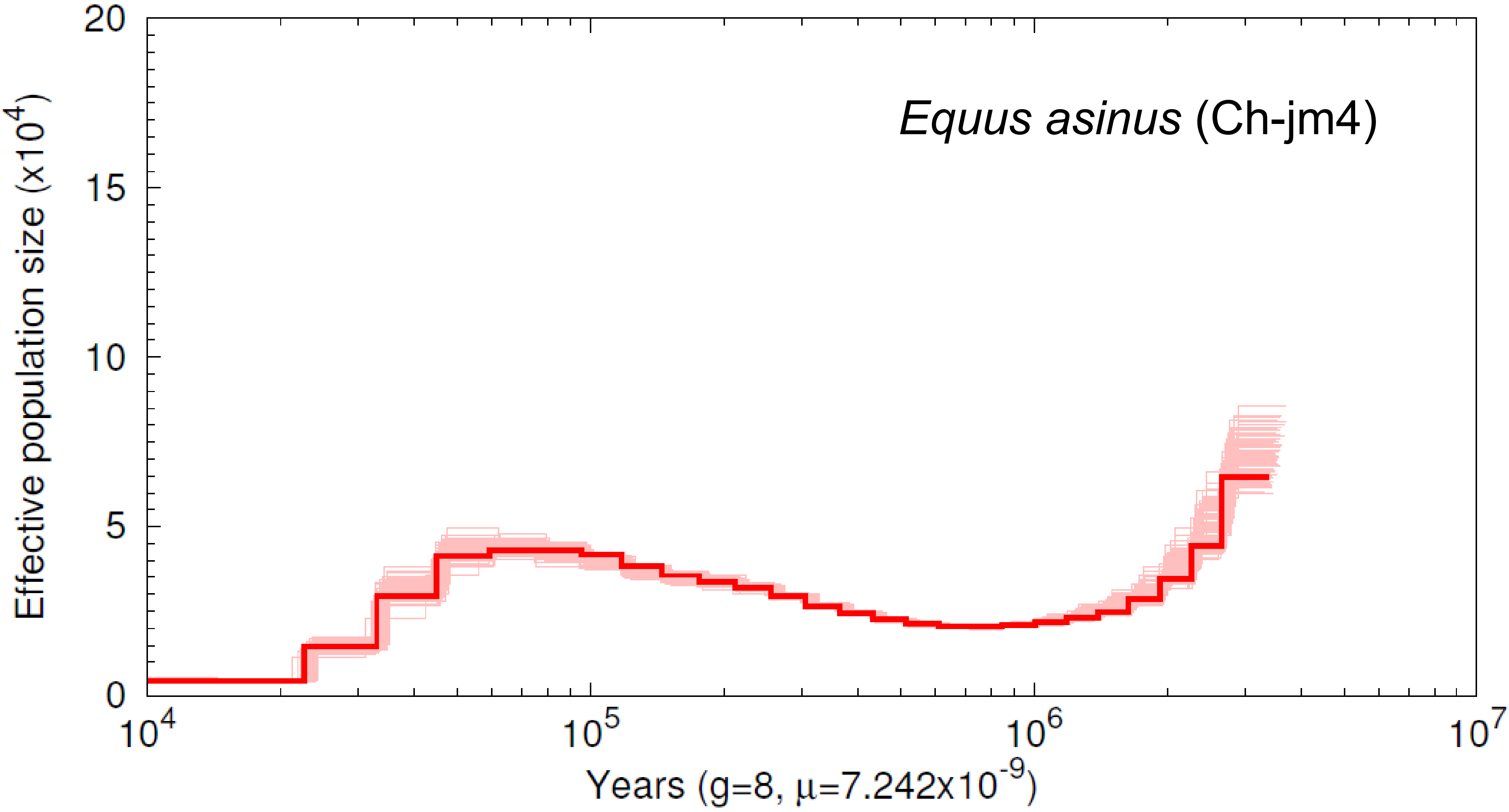

*Equus asinus* (Ch-jm4)
Effective population size (x10^4)
20
15
10
5
0
10^4
10^5
10^6
10^7
Years (g=8, μ=7.242x10^-9)

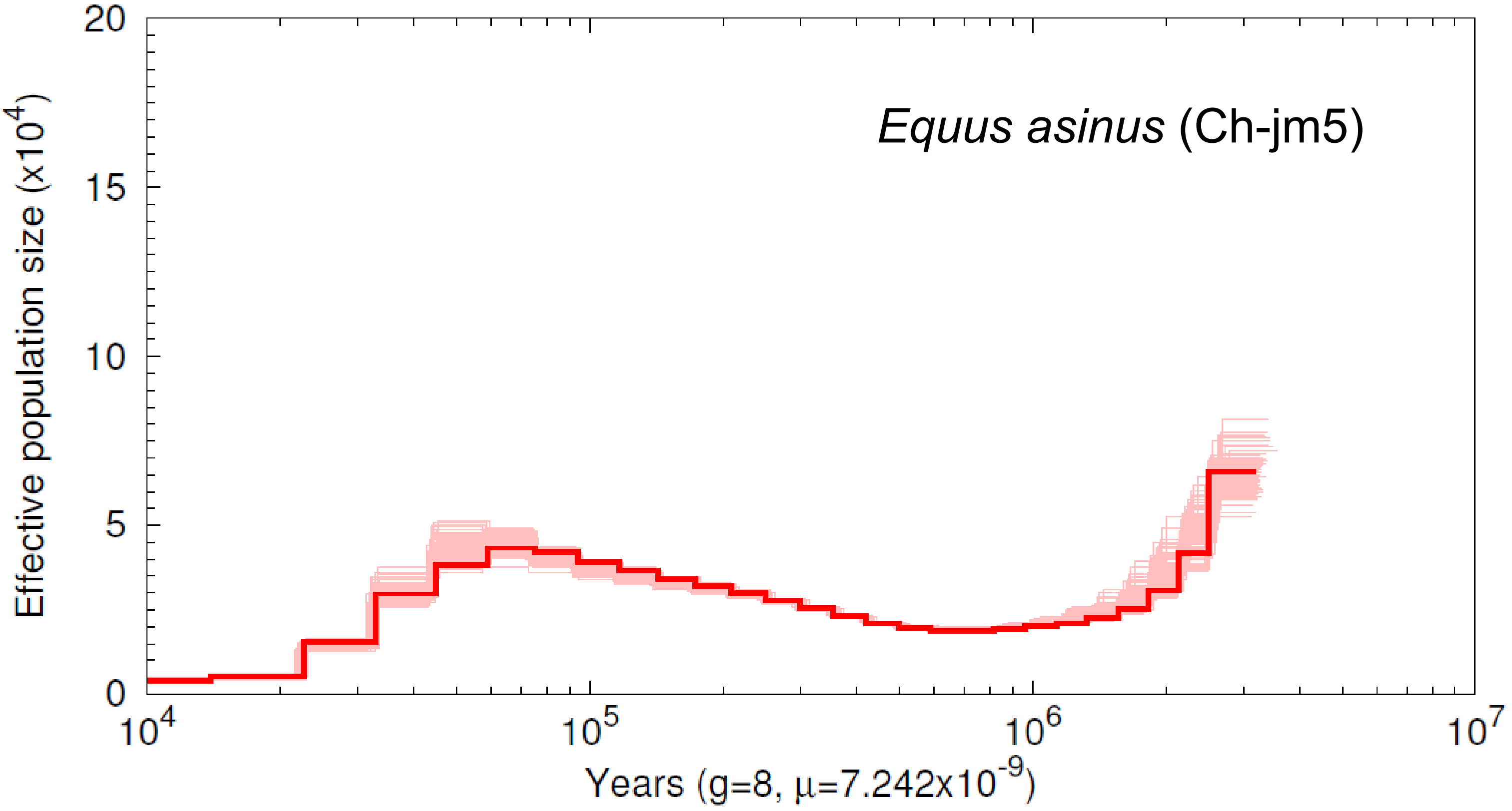

Equus asinus (Ch-jm5)
Effective population size (x10^4)
Years (g=8, μ=7.242x10^-9)


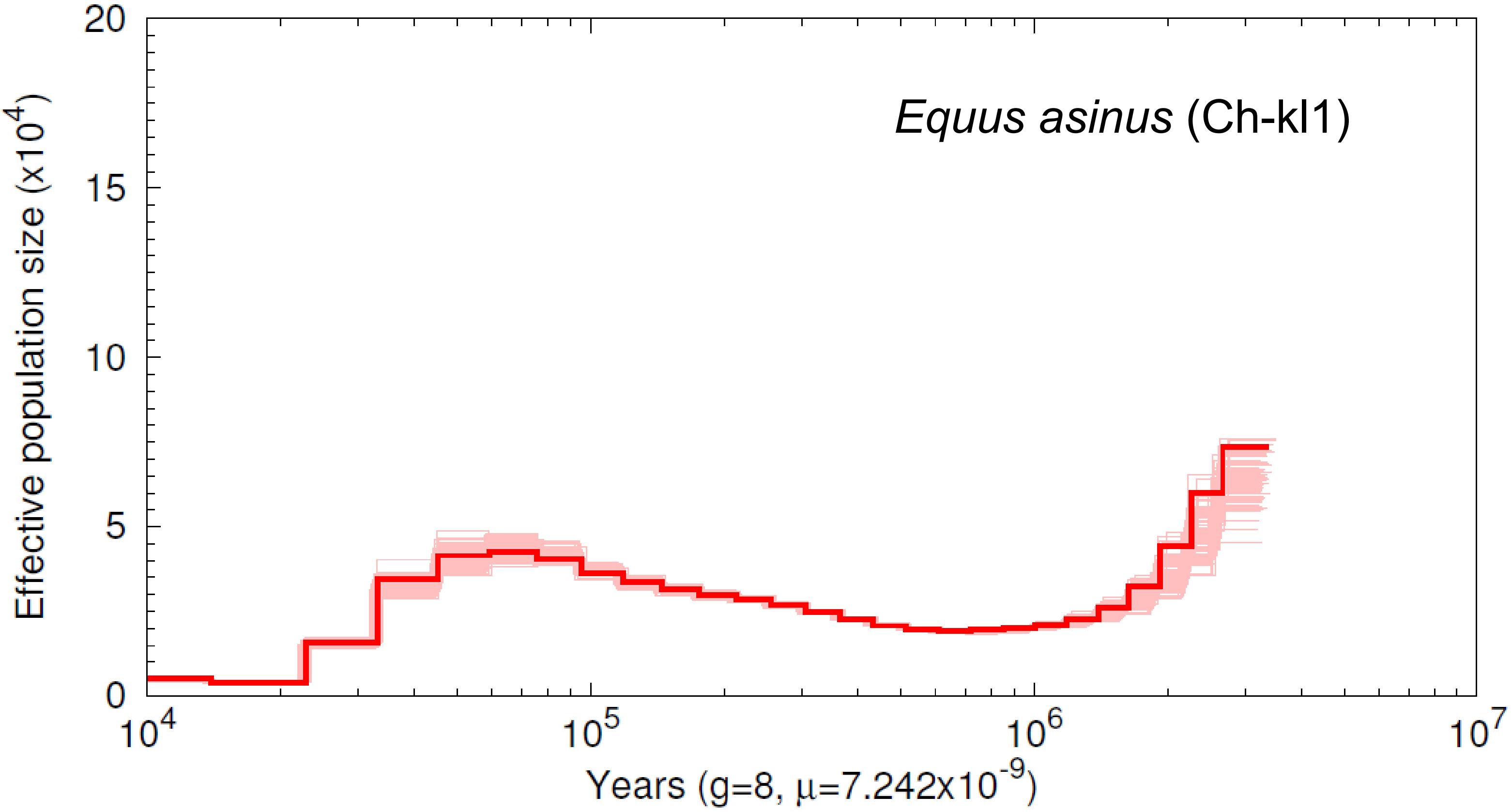

Equus asinus (Ch-kl1)
Effective population size (x10^4)
Years (g=8, μ=7.242x10^-9)

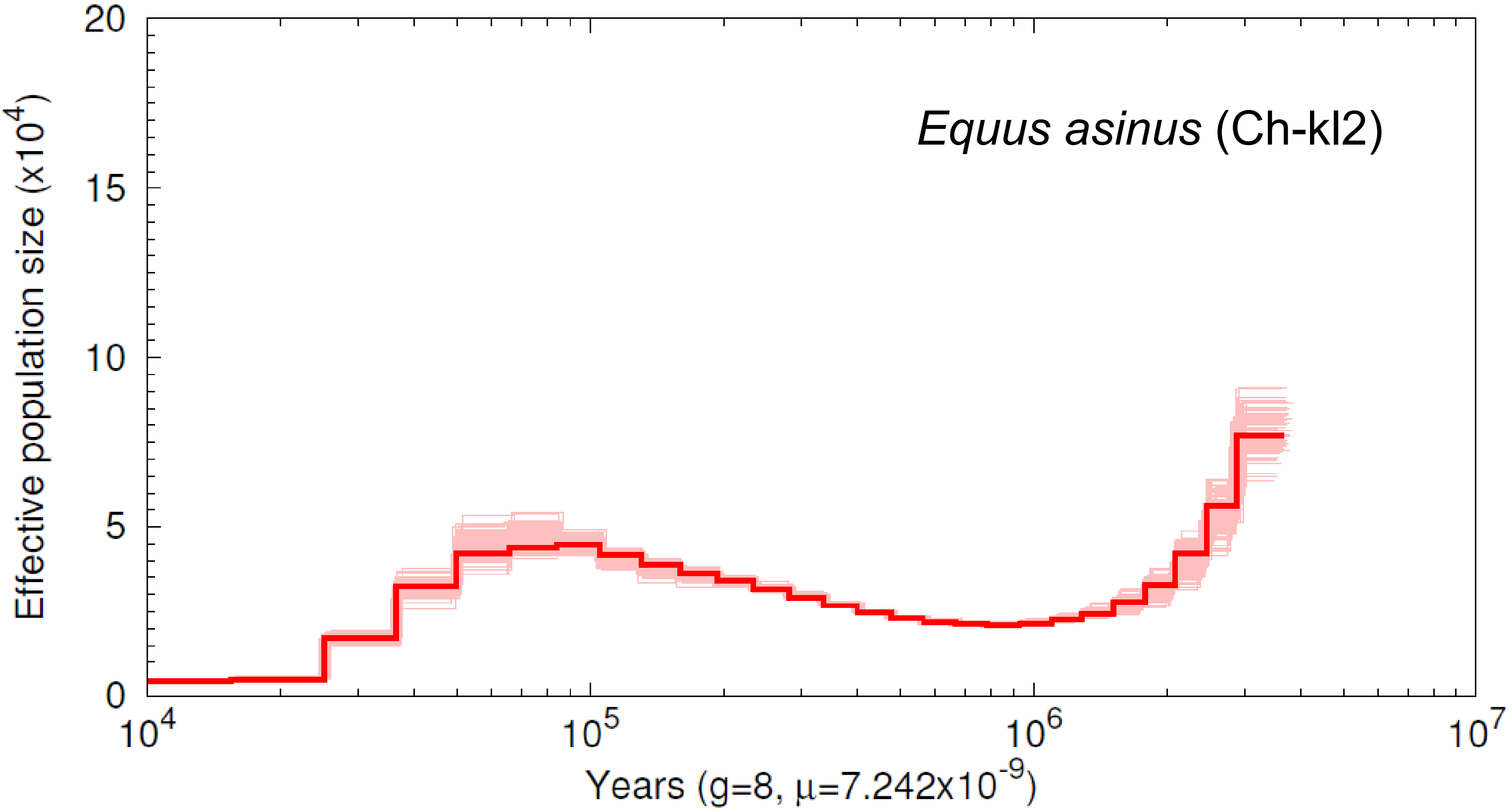
Equus asinus (Ch-kl2)
Effective population size (x10^4)
20
15
10
5
0
10^4
10^5
10^6
10^7
Years (g=8, μ=7.242x10^-9)

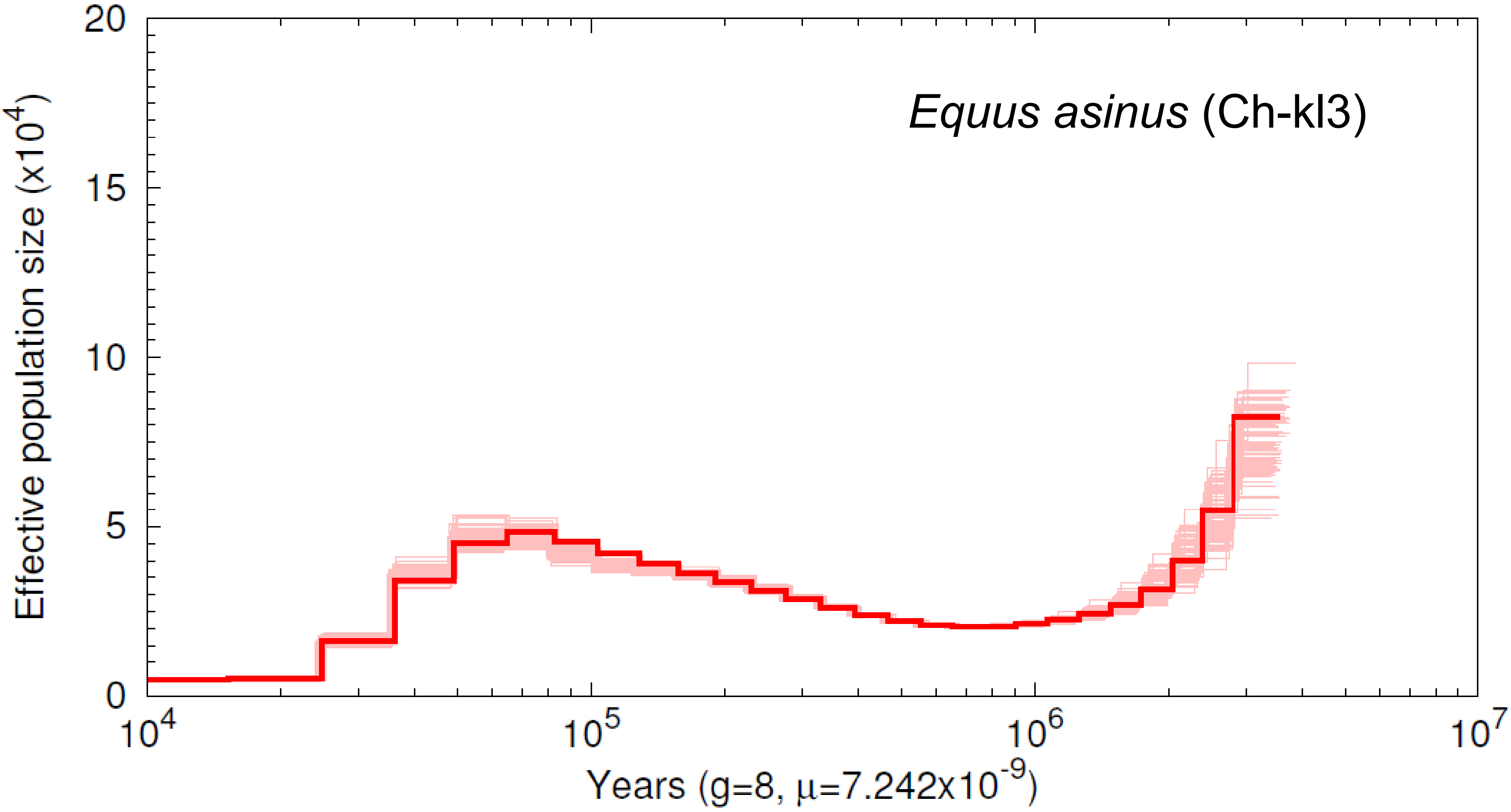
Equus asinus (Ch-kl3)
Effective population size (x10^4)
20
15
10
5
0
10^4
10^5
10^6
10^7
Years (g=8, μ=7.242x10^-9)

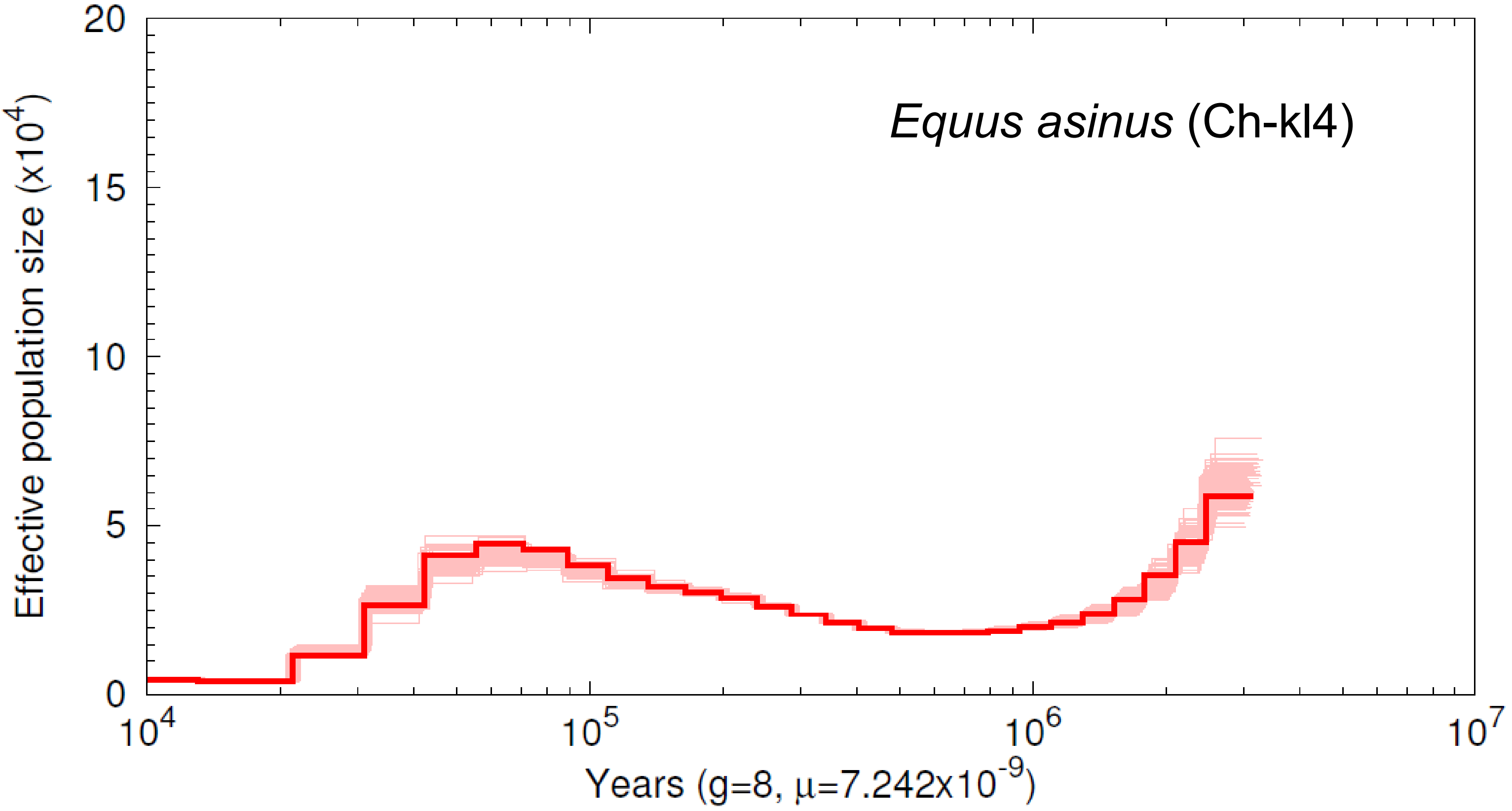
Equus asinus (Ch-kl4)
Effective population size (x10^4)
Years (g=8, μ=7.242x10^-9)
20
15
10
5
0
10^4
10^5
10^6
10^7

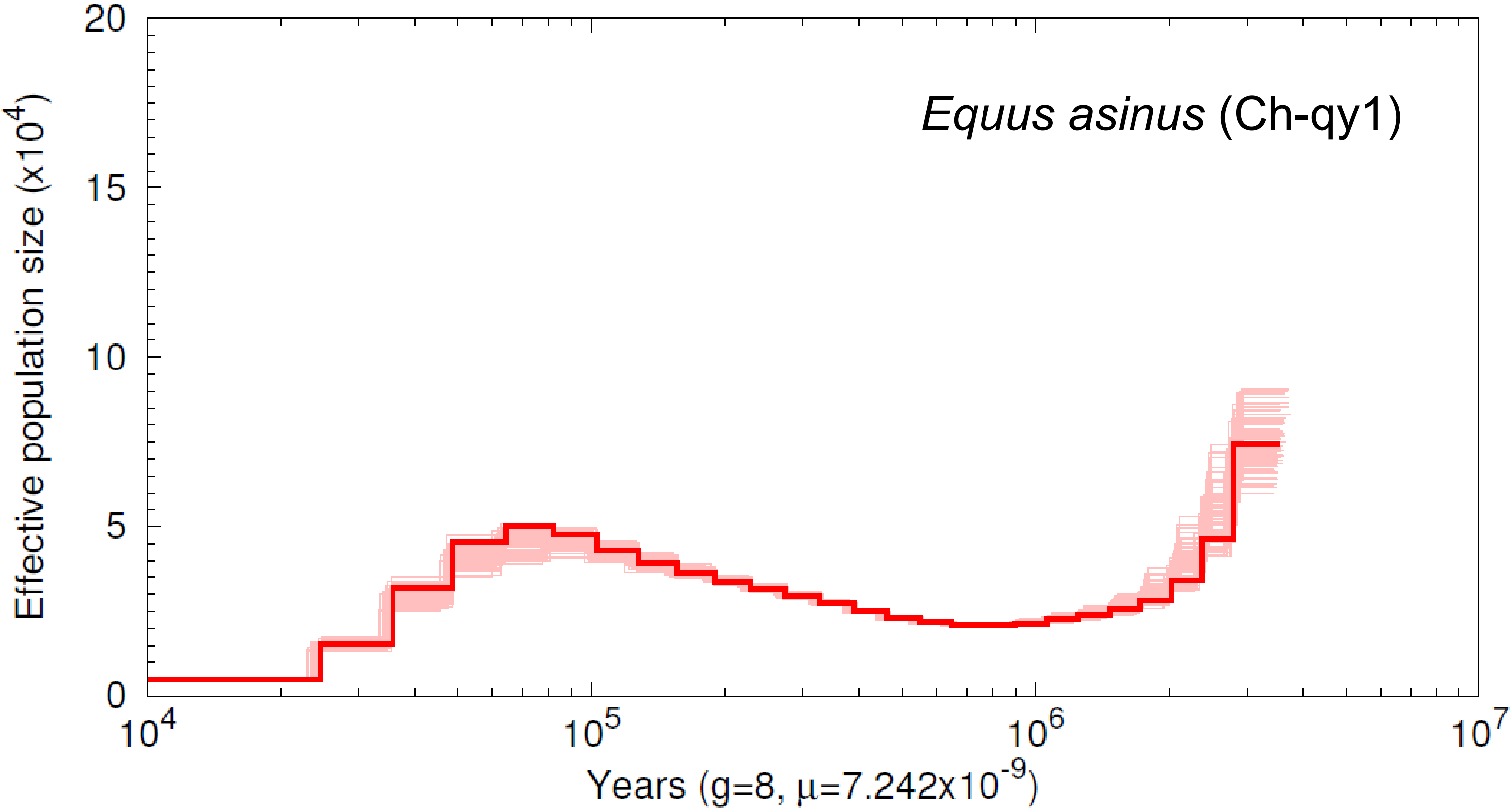
Equus asinus (Ch-qy1)
Effective population size (x10^4)
Years (g=8, μ=7.242x10^-9)
20
15
10
5
0
10^4
10^5
10^6
10^7

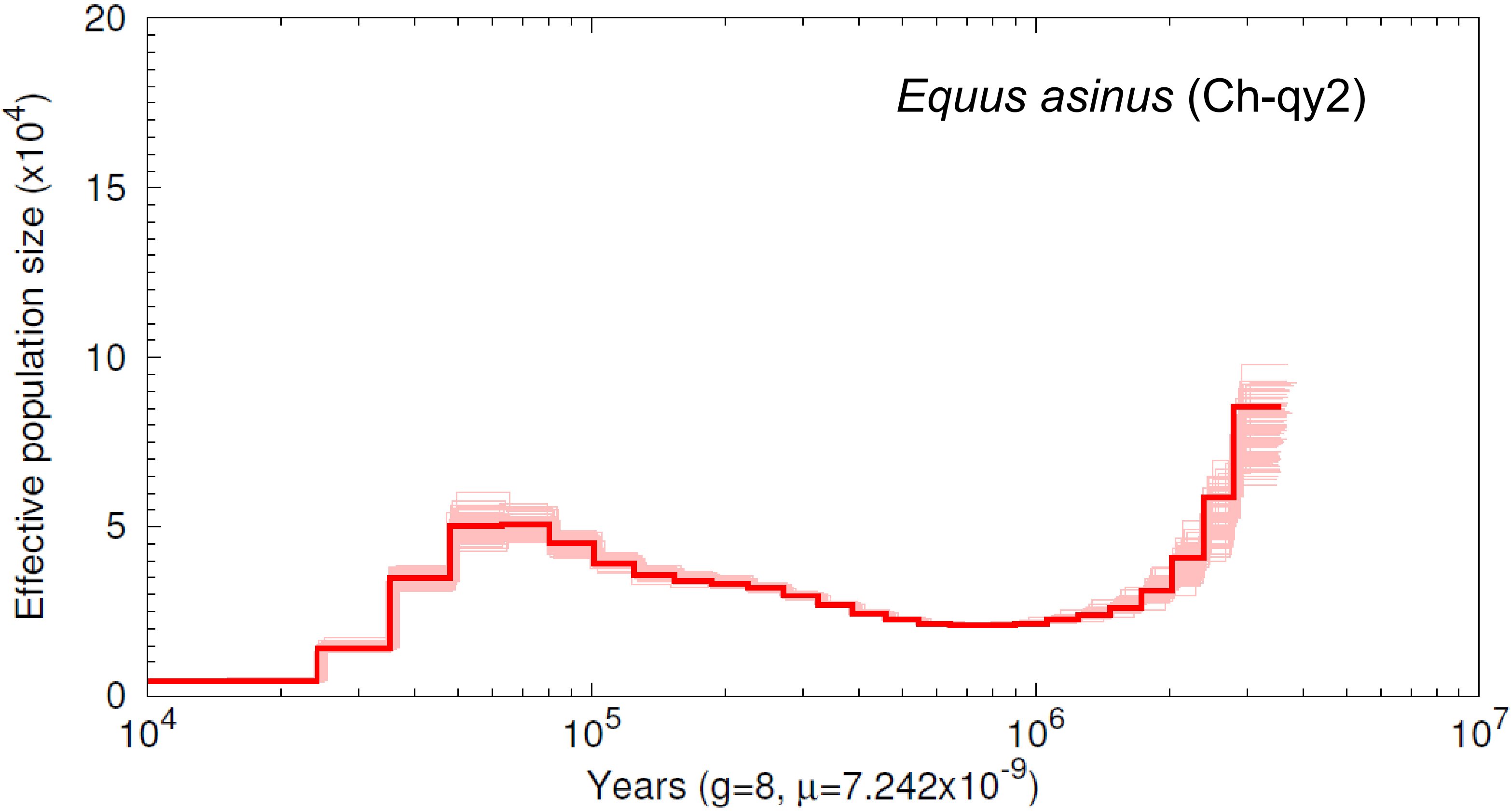
Equus asinus (Ch-qy2)
Effective population size (x10^4)
20
15
10
5
0
10^4
10^5
10^6
10^7
Years (g=8, μ=7.242x10^-9)

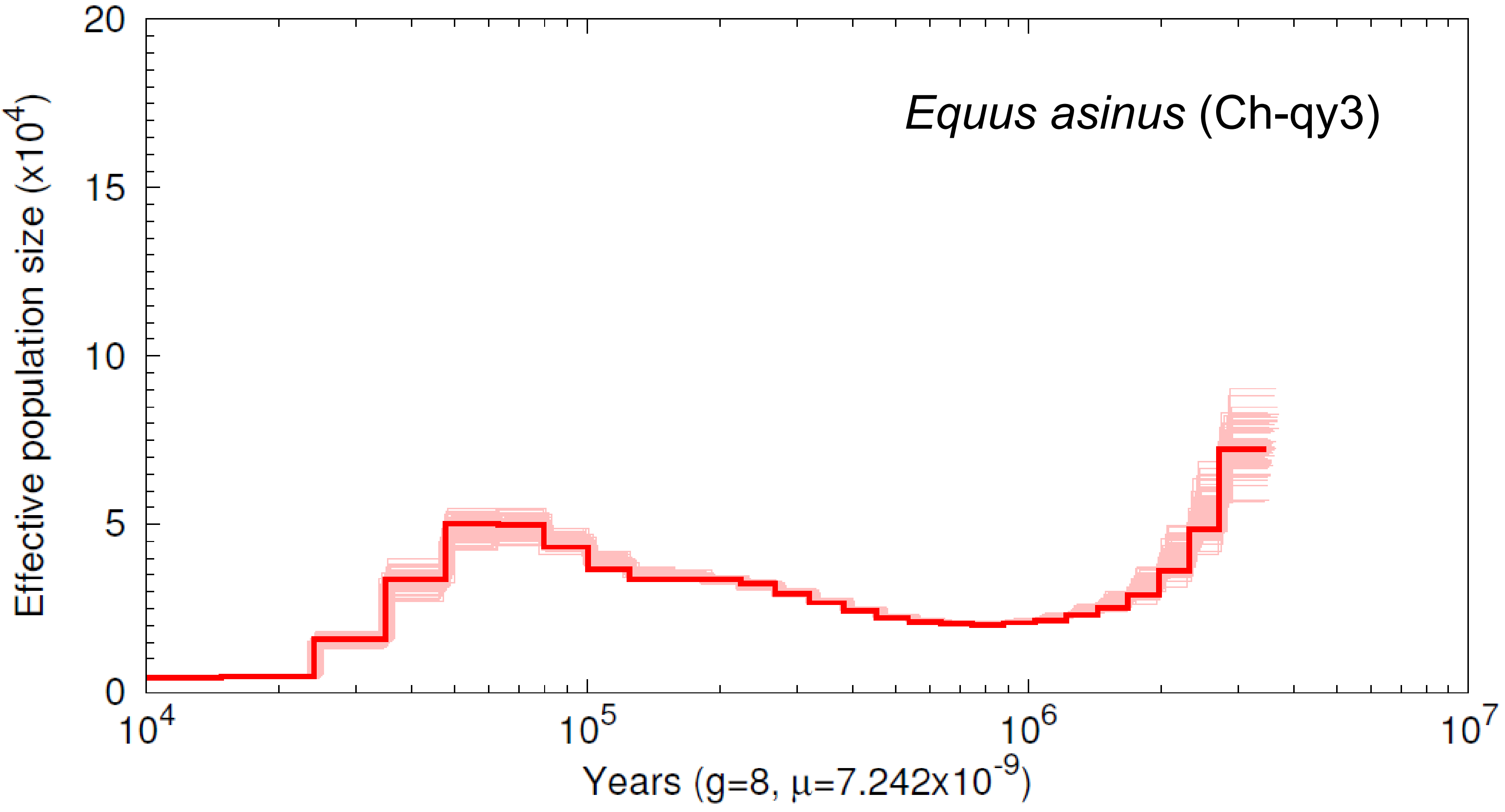
Equus asinus (Ch-qy3)
Effective population size (x10^4)
20
15
10
5
0
10^4
10^5
10^6
10^7
Years (g=8, μ=7.242x10^-9)

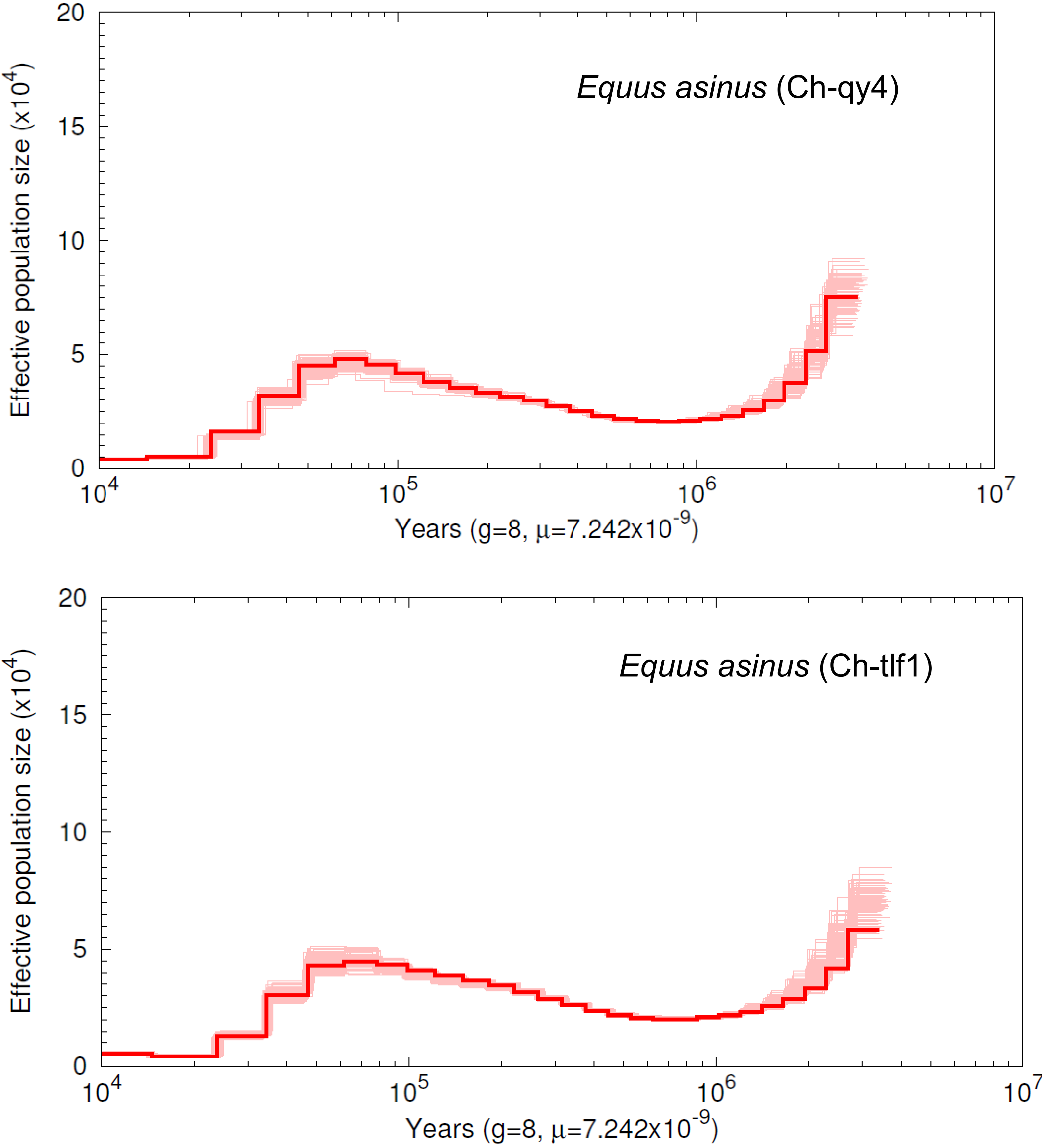

Equus asinus (Ch-qy4)
Effective population size (x10^4)
20
15
10
5
0
10^4
10^5
10^6
10^7
Years (g=8, μ=7.242x10^-9)
Equus asinus (Ch-tlf1)
Effective population size (x10^4)
20
15
10
5
0
10^4
10^5
10^6
10^7
Years (g=8, μ=7.242x10^-9)

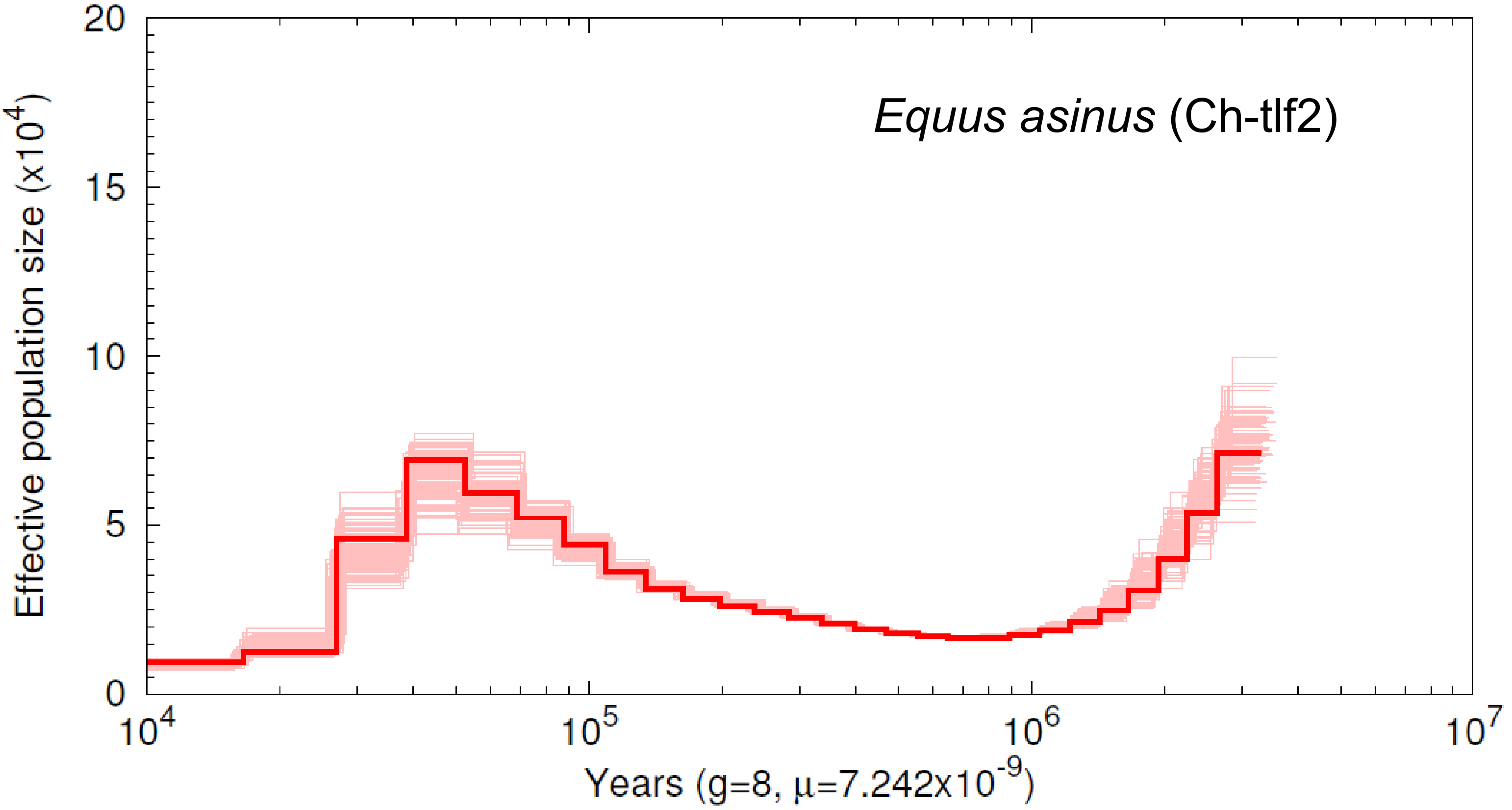
Equus asinus (Ch-tlf2)
Effective population size (x10^4)
Years (g=8, μ=7.242x10^-9)
20
15
10
5
0
10^4
10^5
10^6
10^7

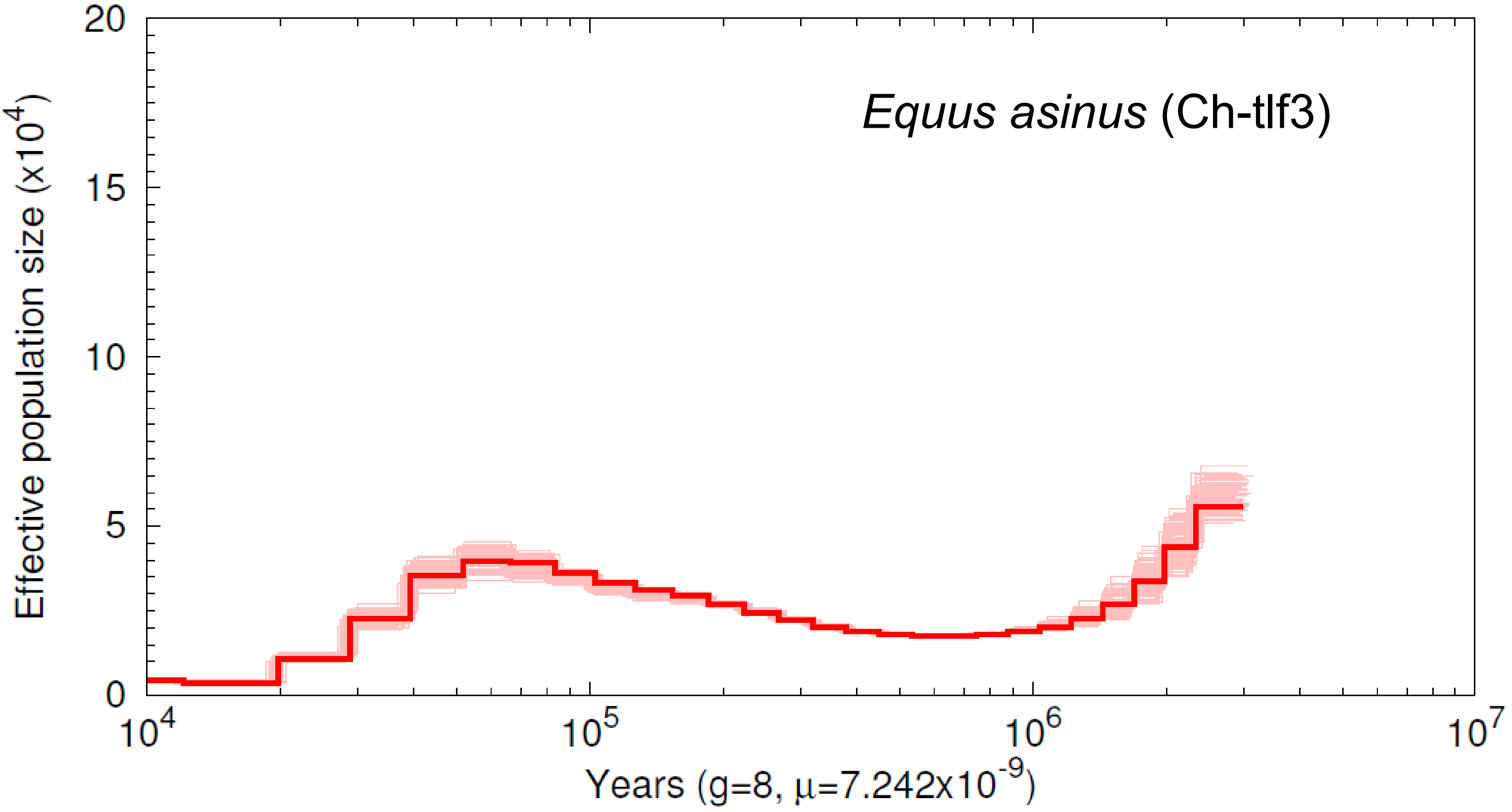
Equus asinus (Ch-tlf3)
Effective population size (x10^4)
Years (g=8, μ=7.242x10^-9)
20
15
10
5
0
10^4
10^5
10^6
10^7

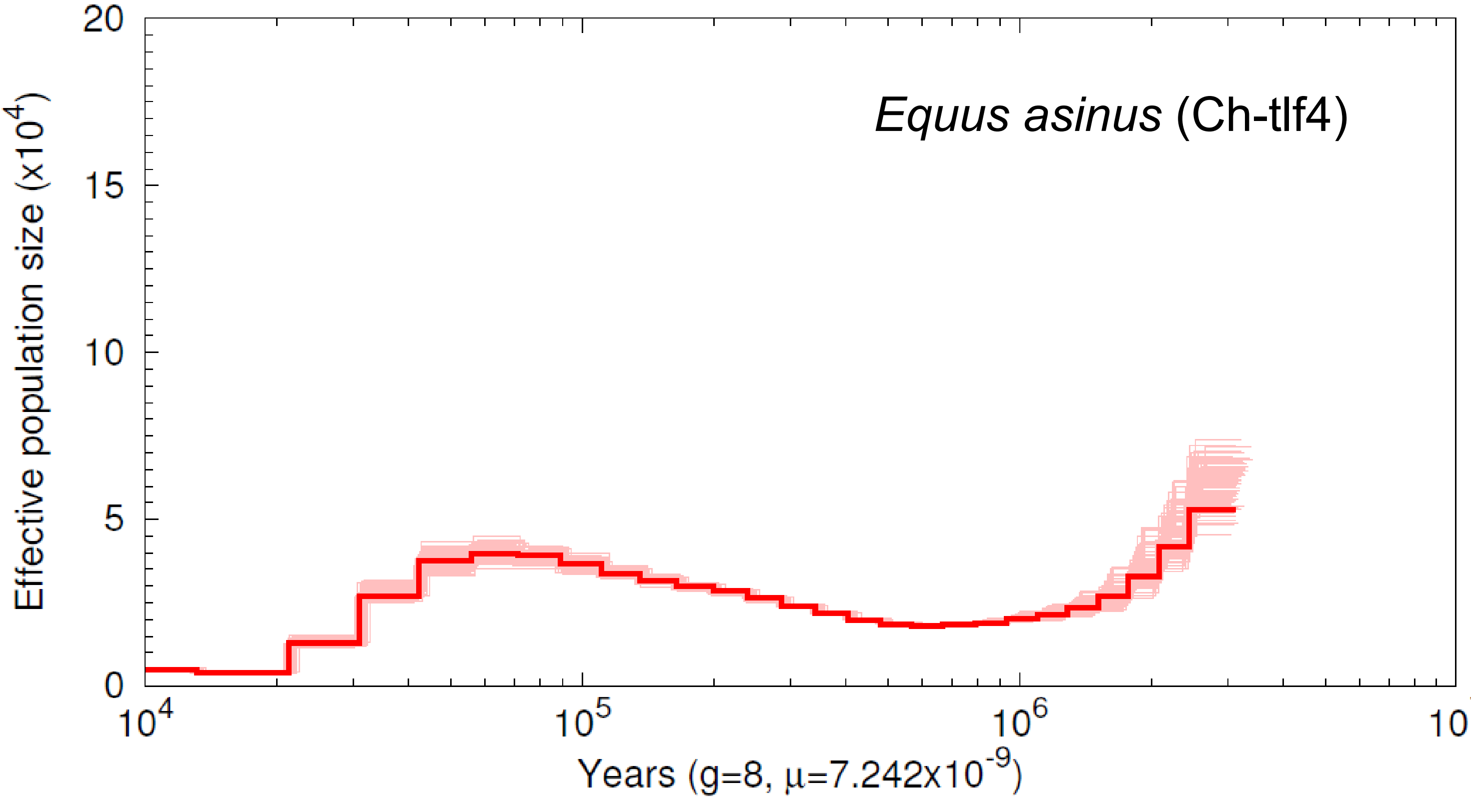
Equus asinus (Ch-tlf4)
Effective population size (x10^4)
20
15
10
5
0
10^4
10^5
10^6
10^7
Years (g=8, μ=7.242x10^-9)

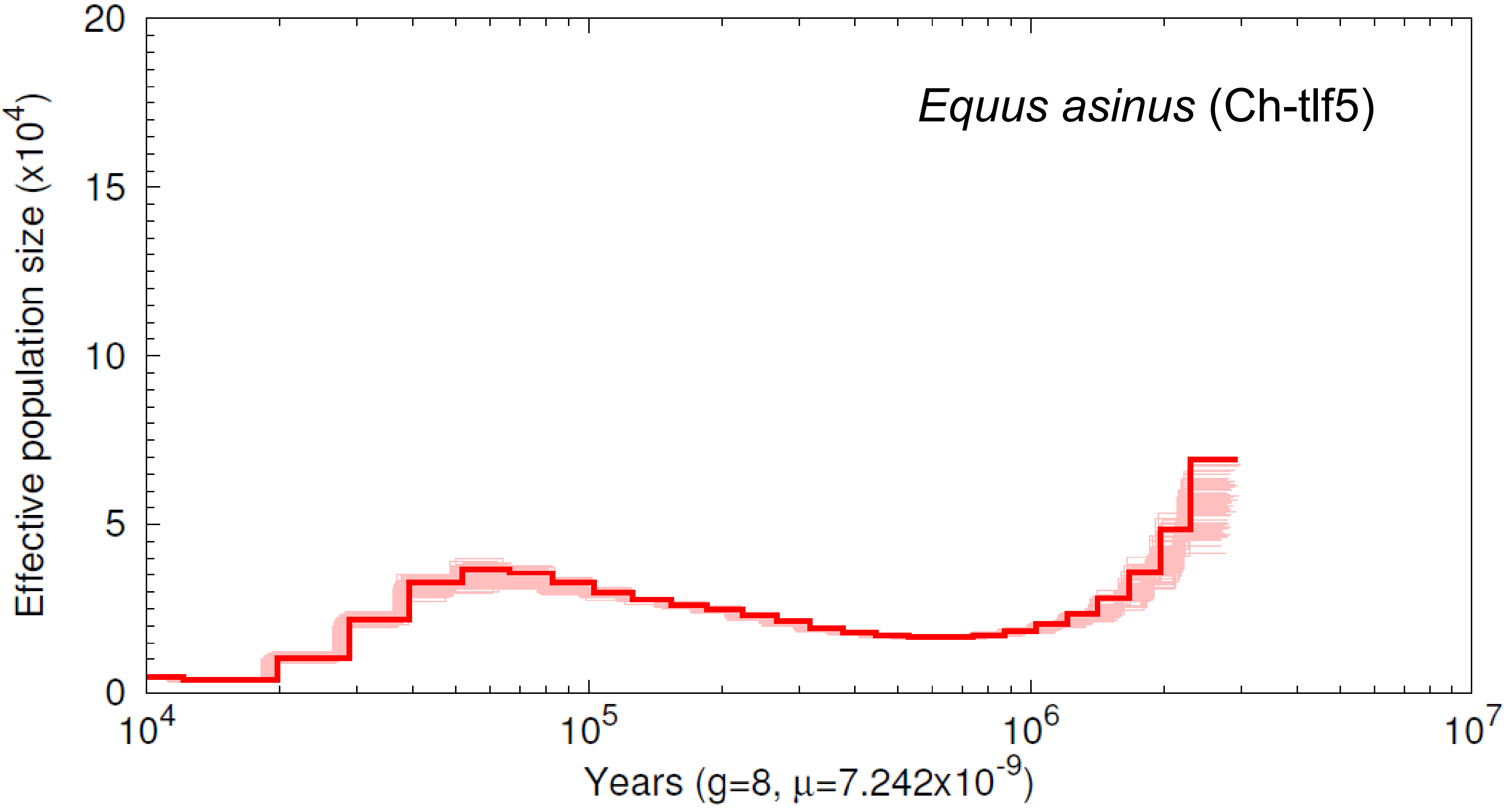
Equus asinus (Ch-tlf5)
Effective population size (x10^4)
20
15
10
5
0
10^4
10^5
10^6
10^7
Years (g=8, μ=7.242x10^-9)

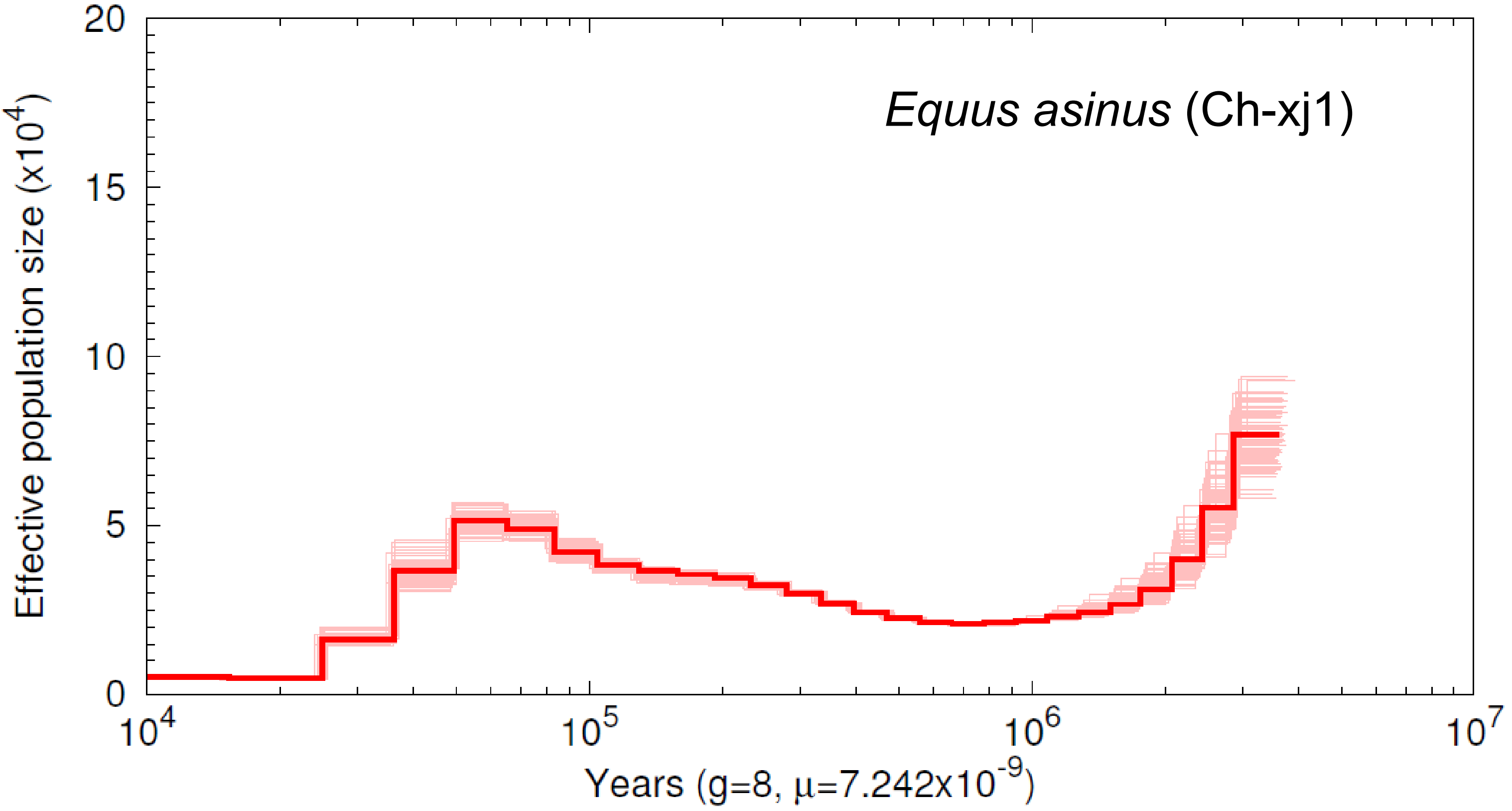
Equus asinus (Ch-xj1)
Effective population size (x10^4)
Years (g=8, μ=7.242x10^-9)
20
15
10
5
0
10^4
10^5
10^6
10^7

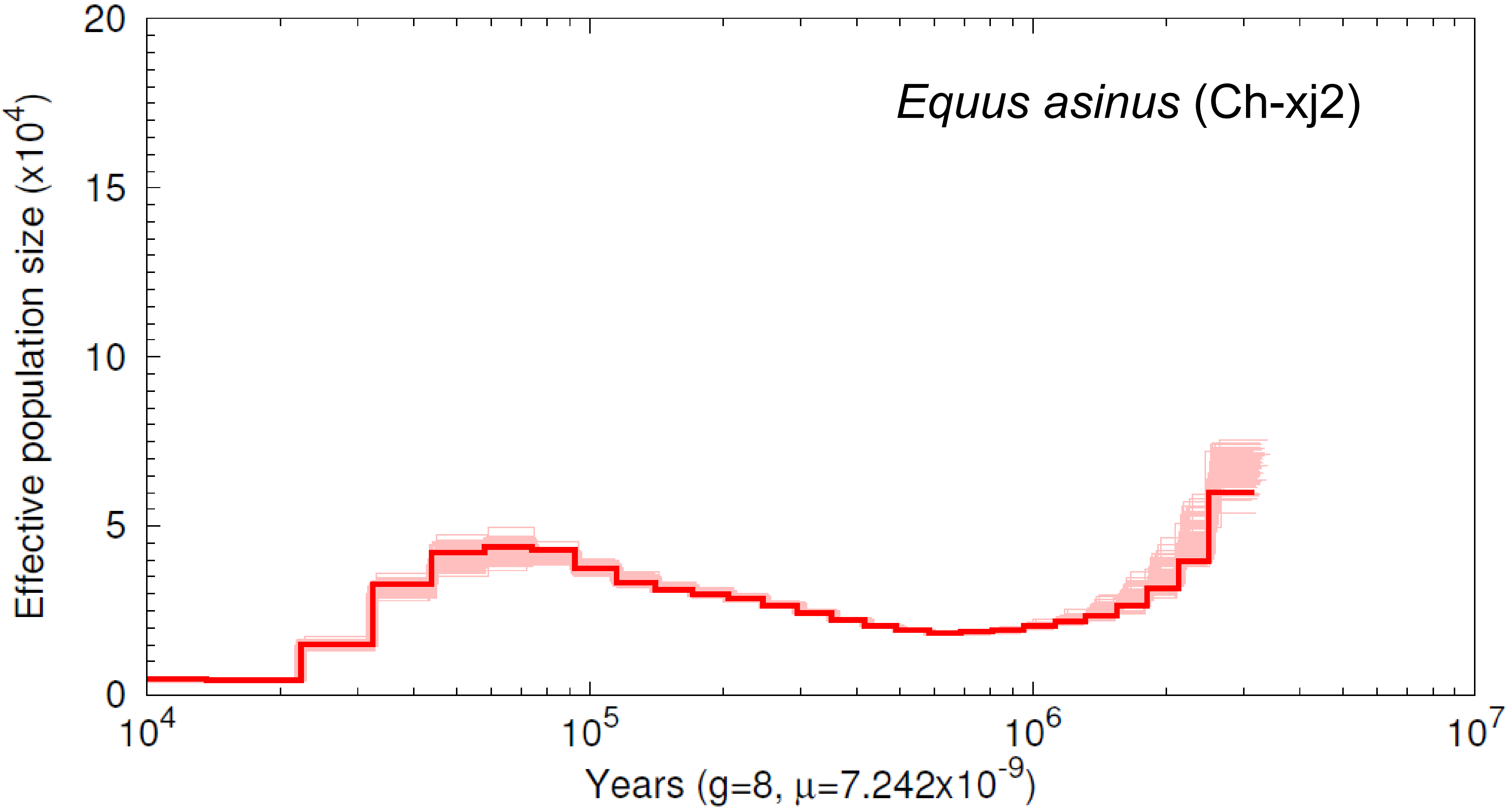
Equus asinus (Ch-xj2)
Effective population size (x10^4)
Years (g=8, μ=7.242x10^-9)
20
15
10
5
0
10^4
10^5
10^6
10^7

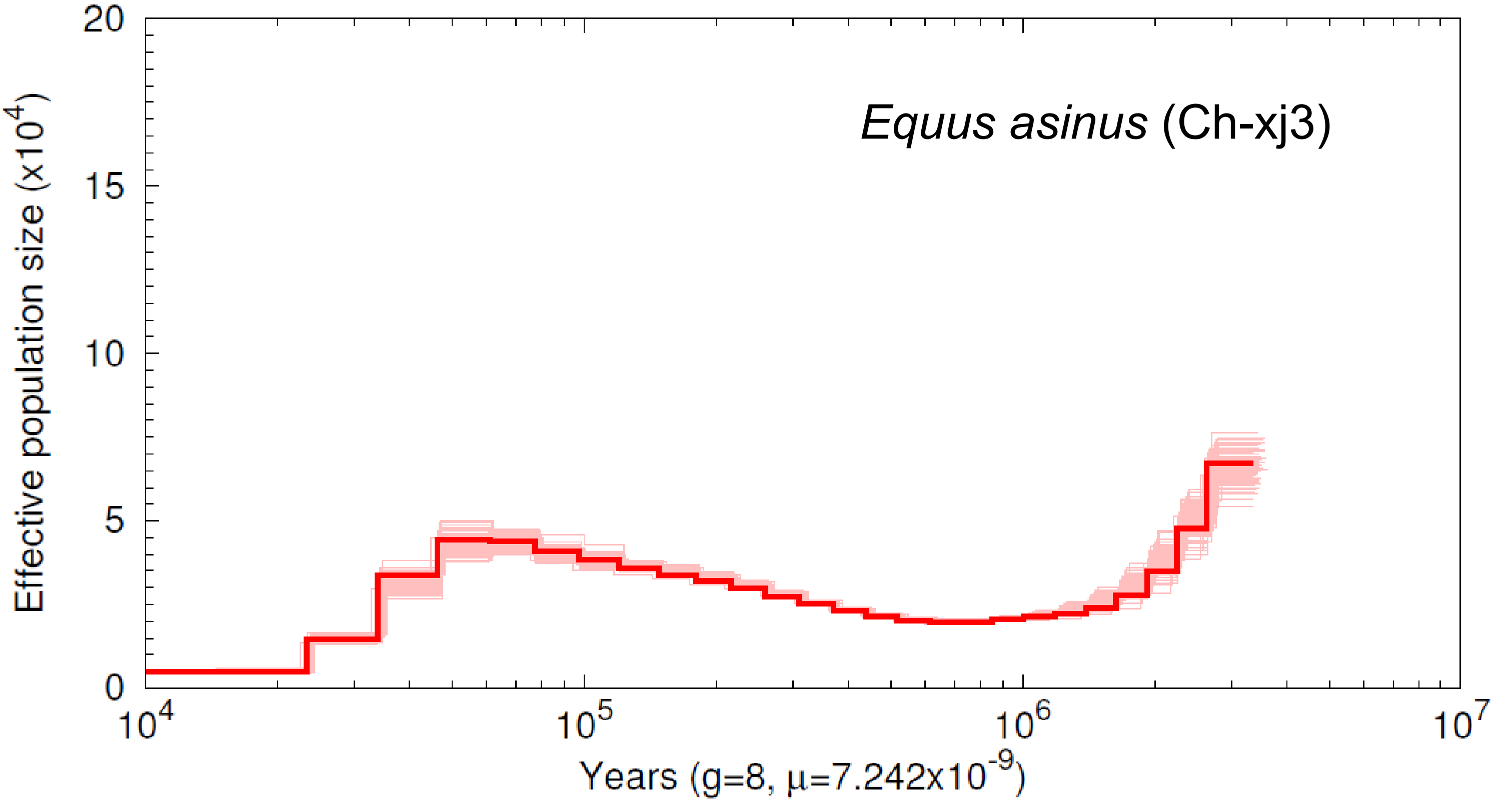
Equus asinus (Ch-xj3)
Effective population size (x10^4)
20
15
10
5
0
10^4
10^5
10^6
10^7
Years (g=8, μ=7.242x10^-9)

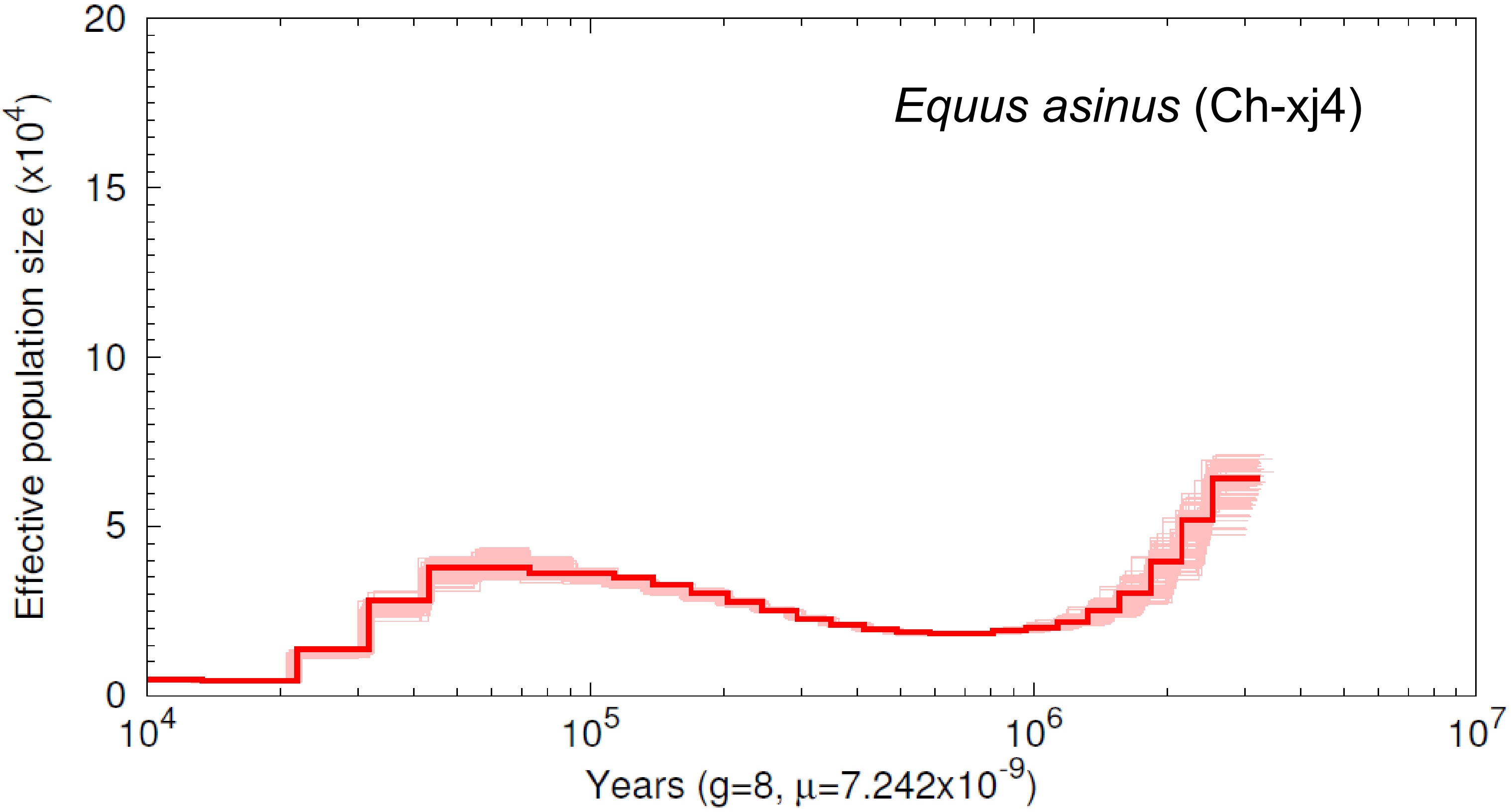
Equus asinus (Ch-xj4)
Effective population size (x10^4)
20
15
10
5
0
10^4
10^5
10^6
10^7
Years (g=8, μ=7.242x10^-9)

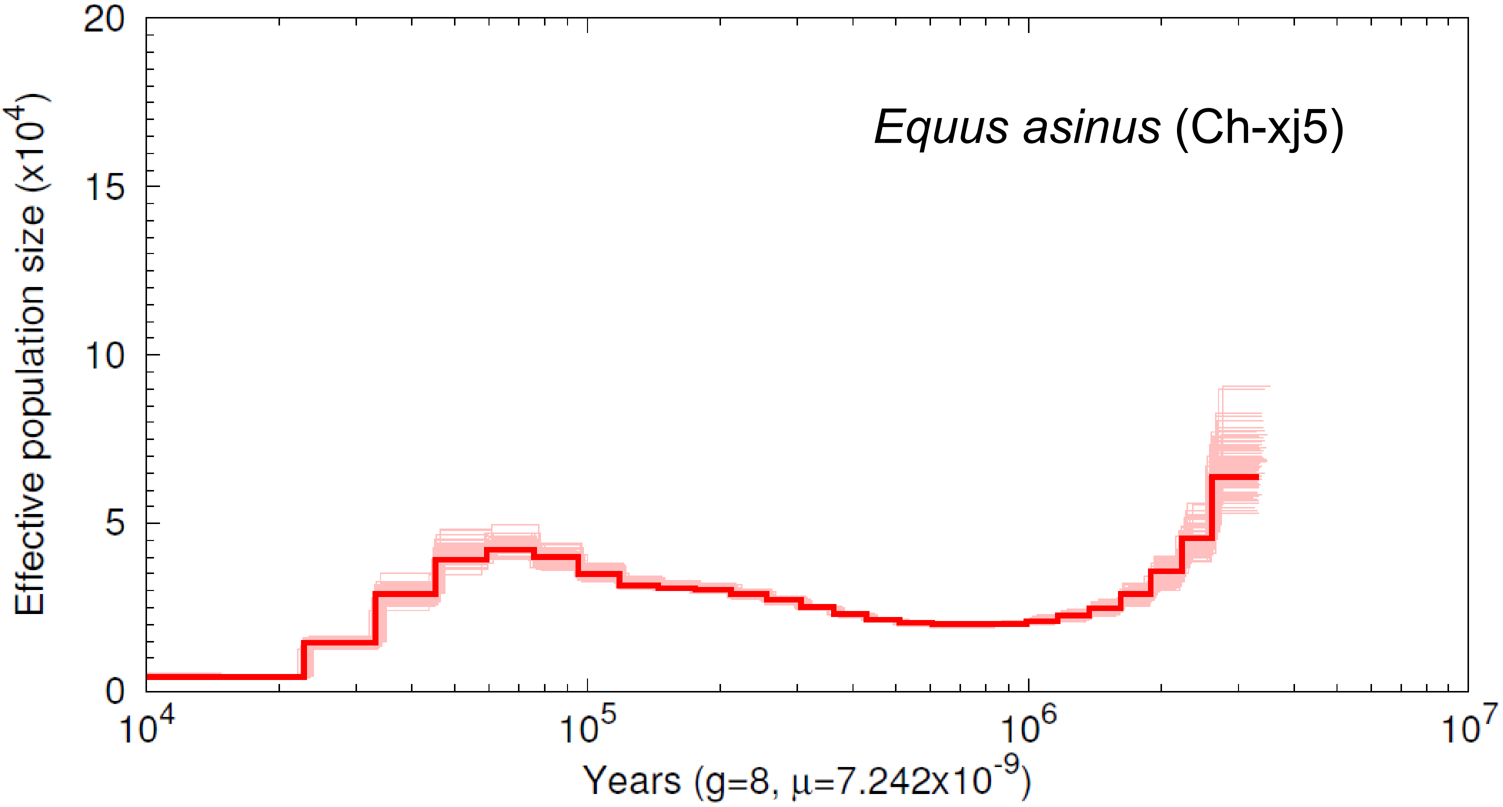
Equus asinus (Ch-xj5)
Effective population size (x10^4)
20
15
10
5
0
10^4
10^5
10^6
10^7
Years (g=8, μ=7.242x10^-9)

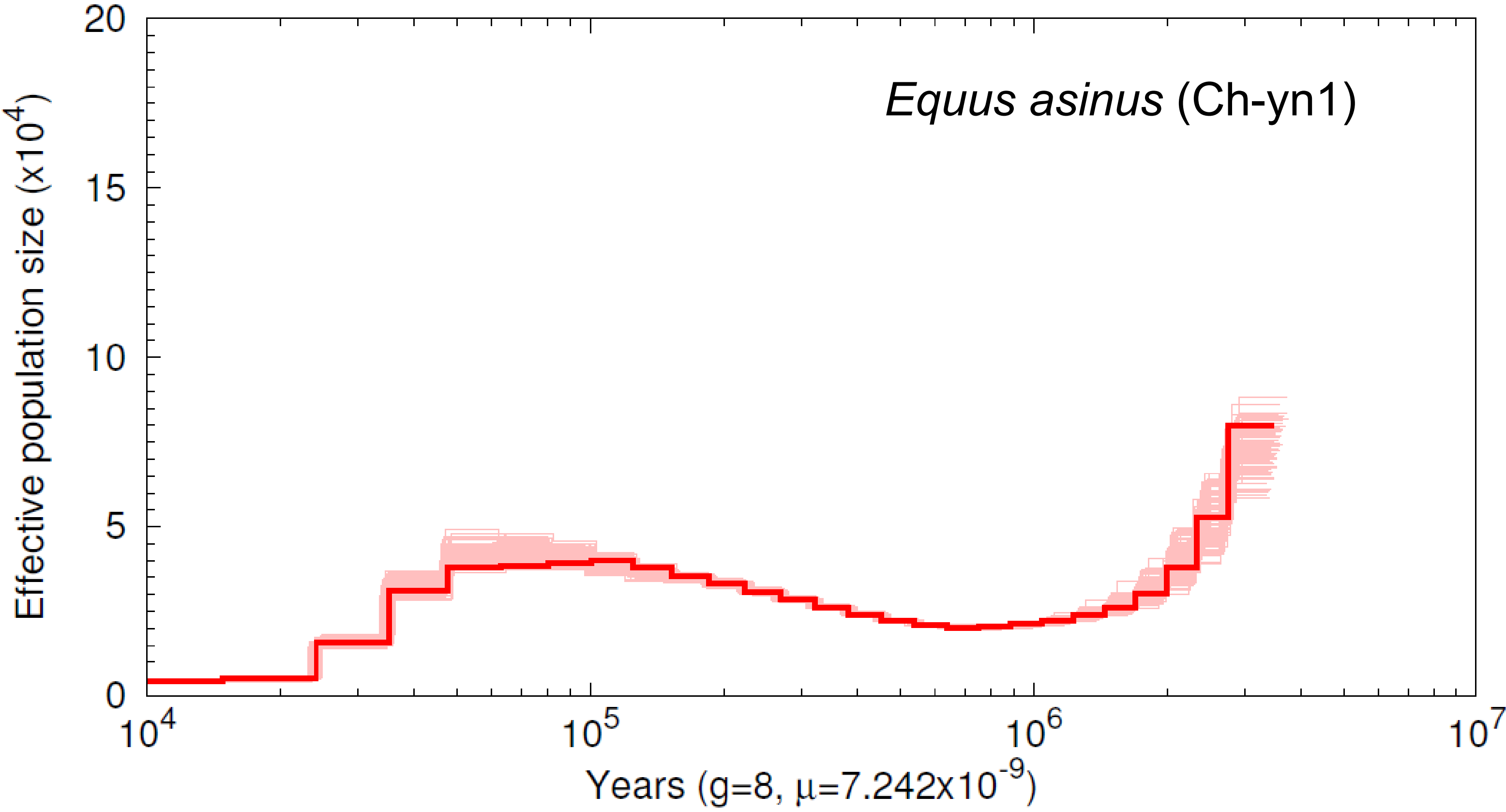
Equus asinus (Ch-yn1)
Effective population size (x10^4)
20
15
10
5
0
10^4
10^5
10^6
10^7
Years (g=8, μ=7.242x10^-9)

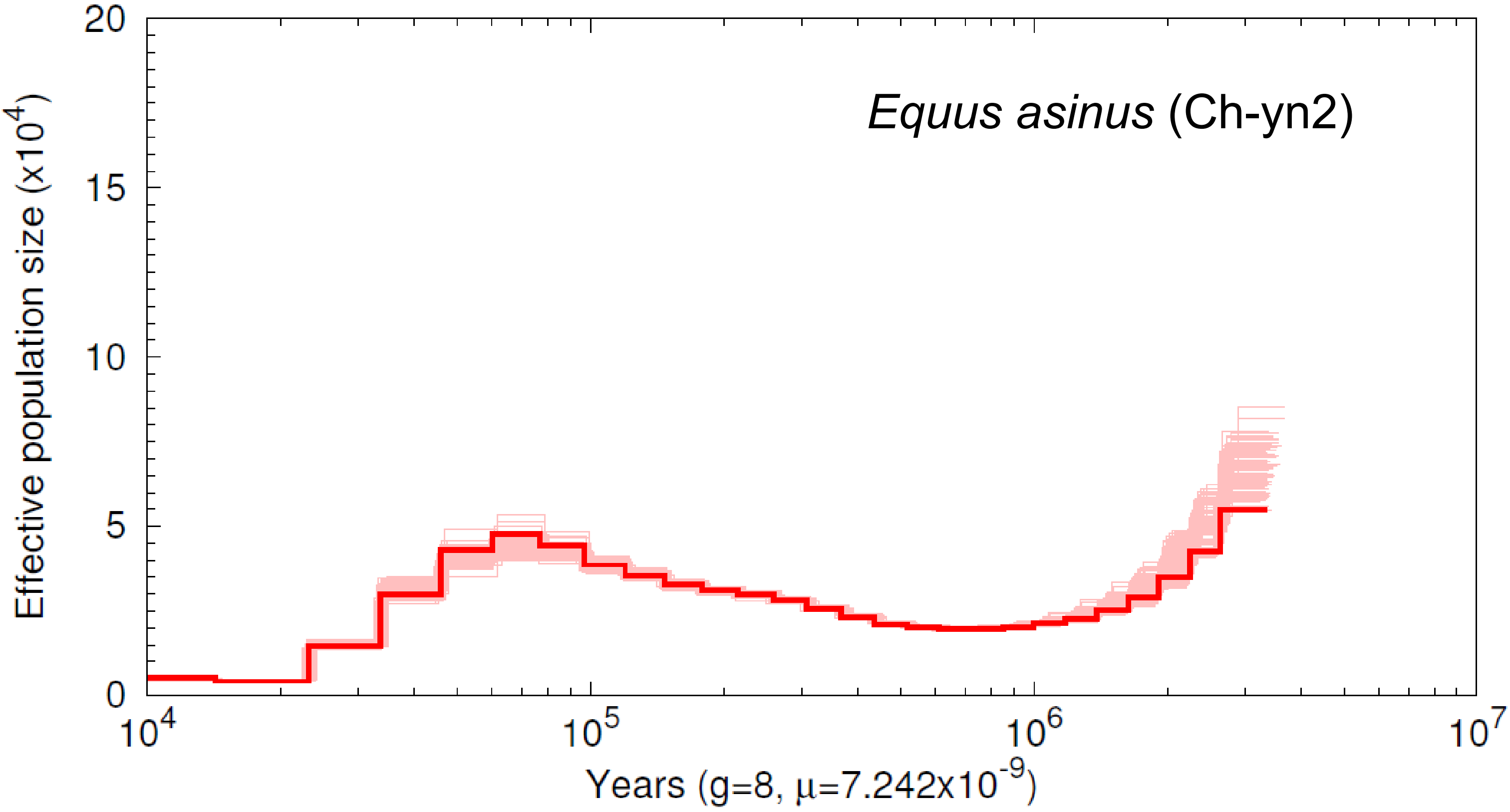
Equus asinus (Ch-yn2)
Effective population size (x10^4)
20
15
10
5
0
10^4
10^5
10^6
10^7
Years (g=8, μ=7.242x10^-9)

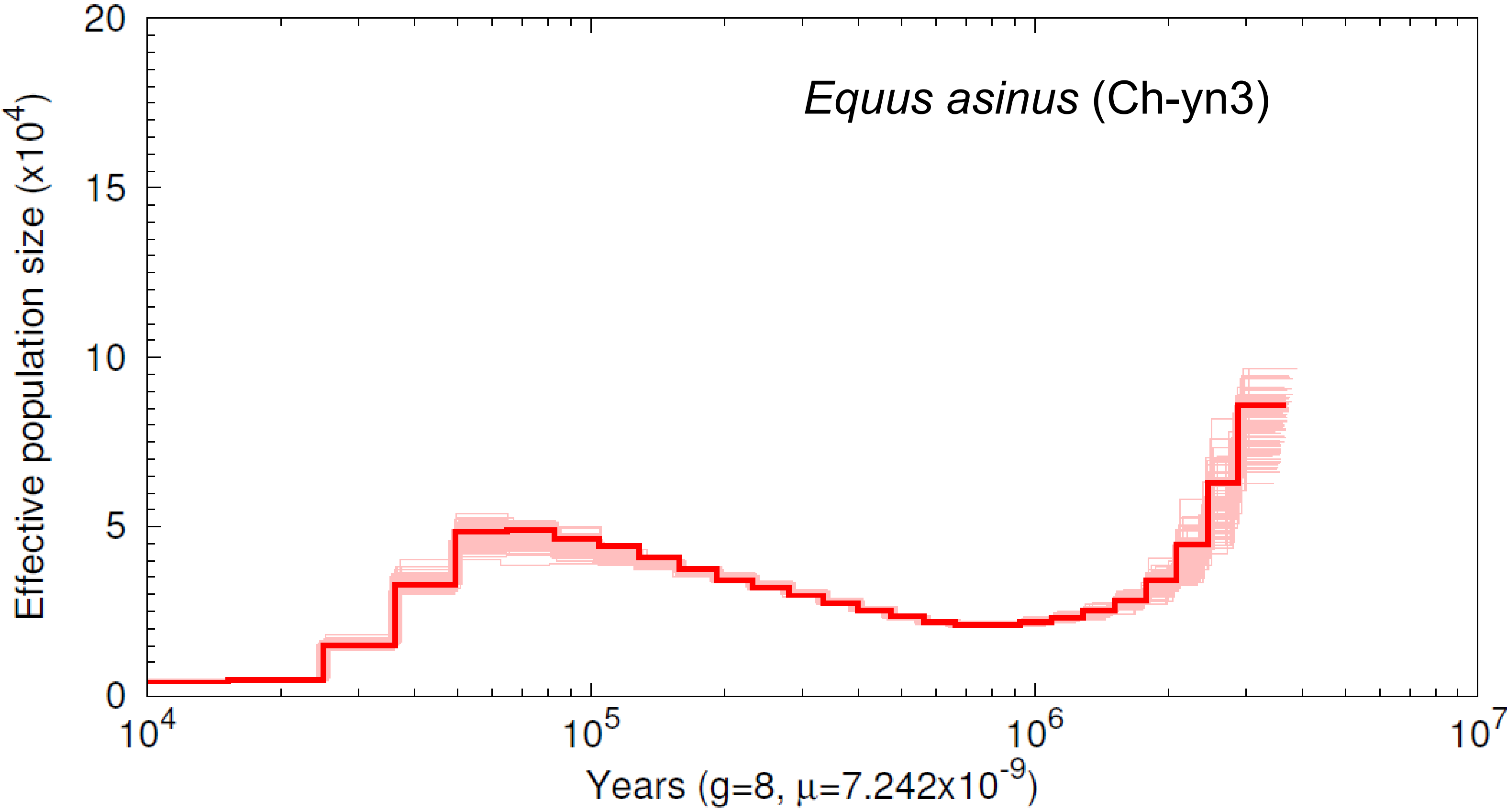
Equus asinus (Ch-yn3)
Effective population size (x10^4)
20
15
10
5
0
10^4
10^5
10^6
10^7
Years (g=8, μ=7.242x10^-9)

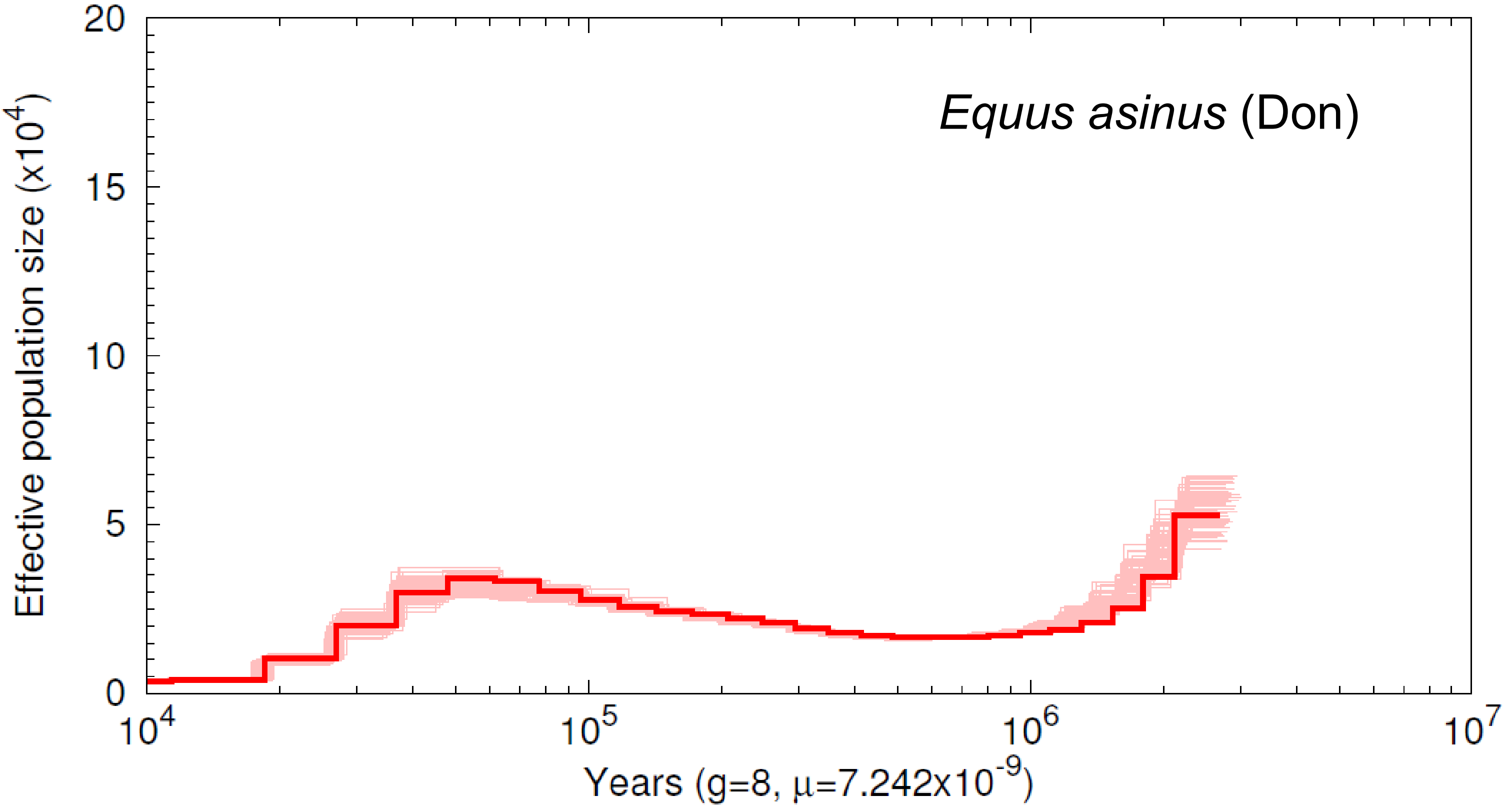

Equus asinus (Don)
Effective population size (x10^4)
20
15
10
5
0
10^4
10^5
10^6
10^7
Years (g=8, μ=7.242x10^-9)


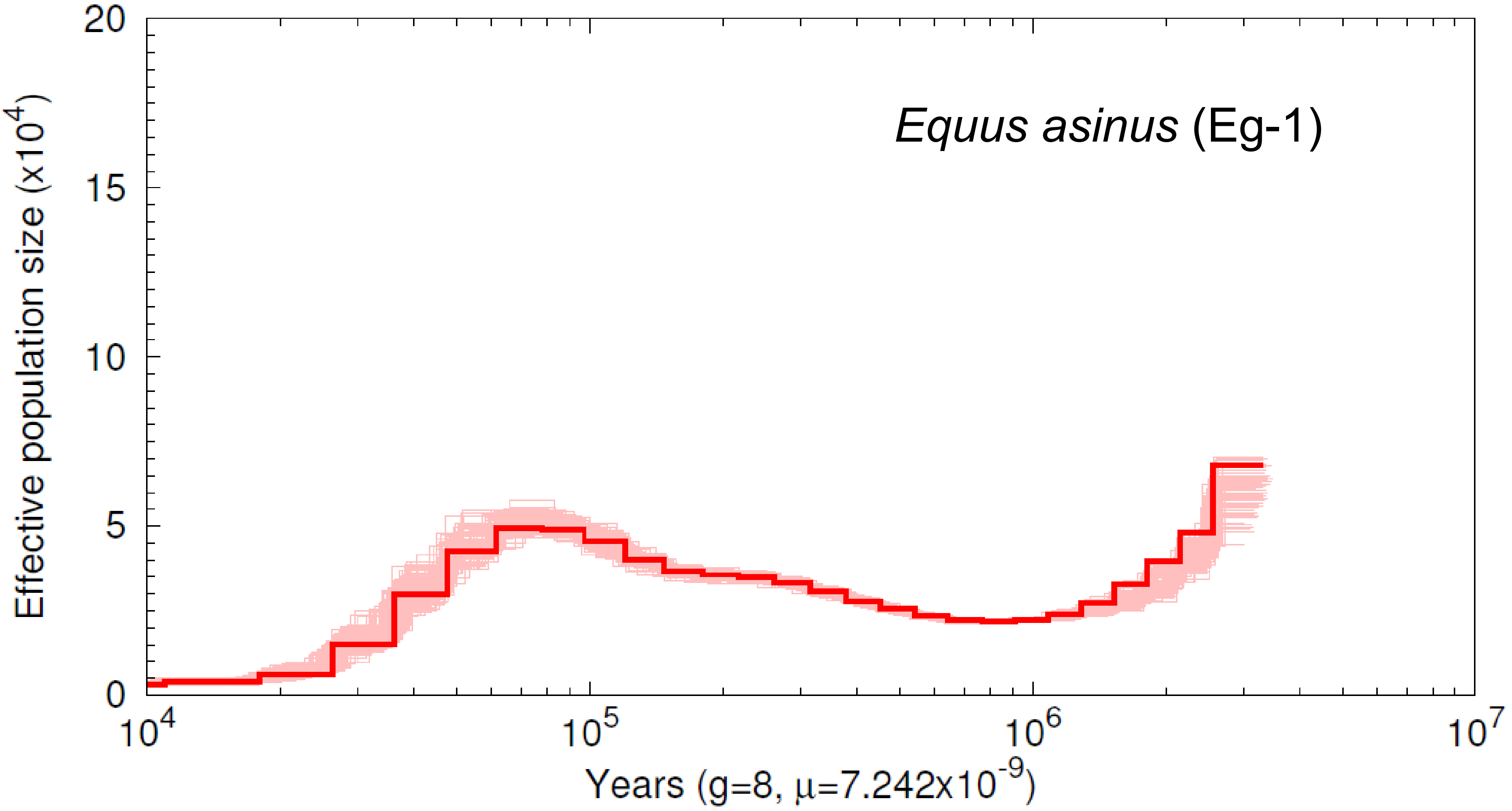

Equus asinus (Eg-1)
Effective population size (x10^4)
20
15
10
5
0
10^4
10^5
10^6
10^7
Years (g=8, μ=7.242x10^-9)

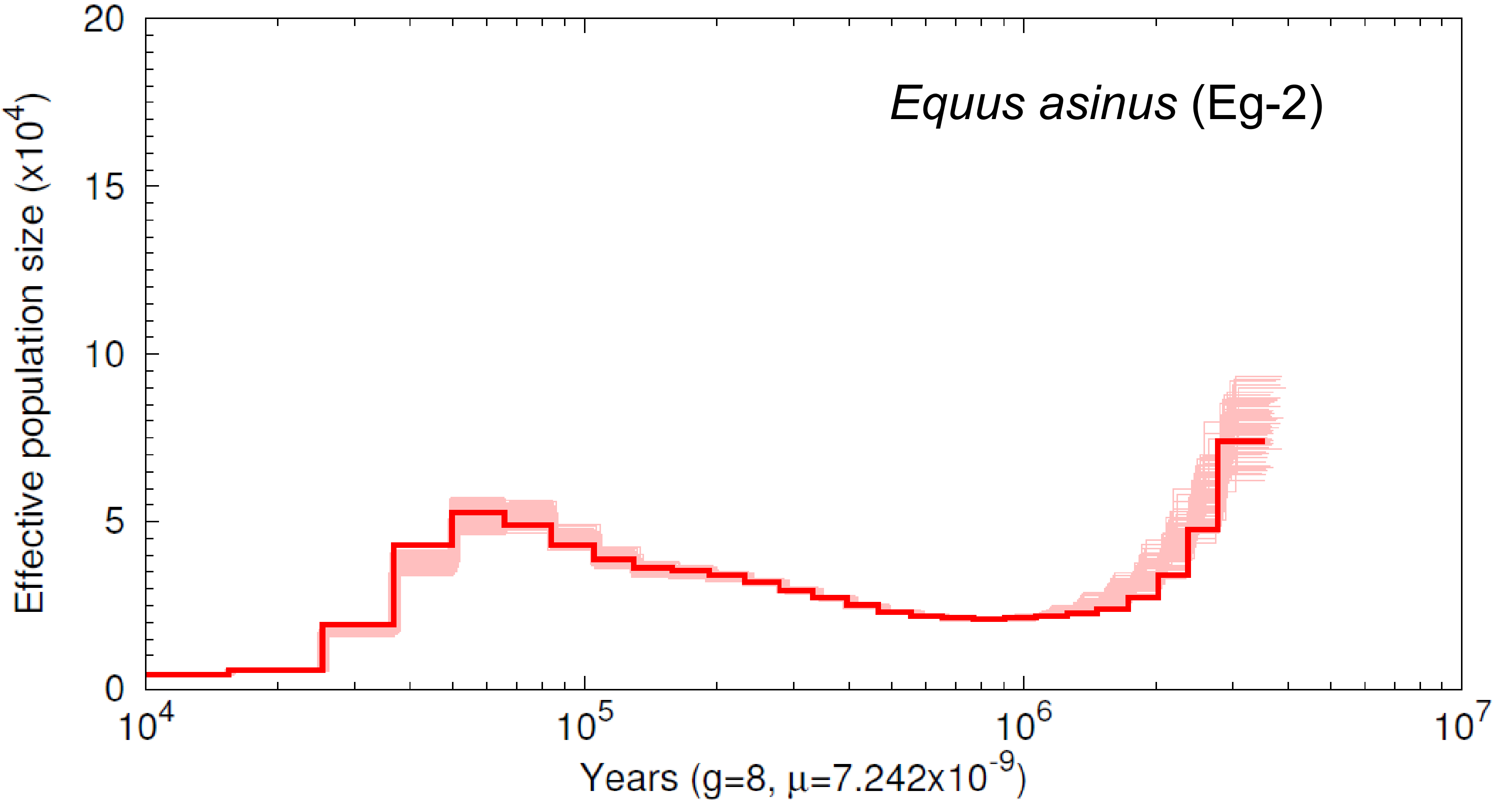
Equus asinus (Eg-2)
Effective population size (x10^4)
20
15
10
5
0
10^4
10^5
10^6
10^7
Years (g=8, μ=7.242x10^-9)

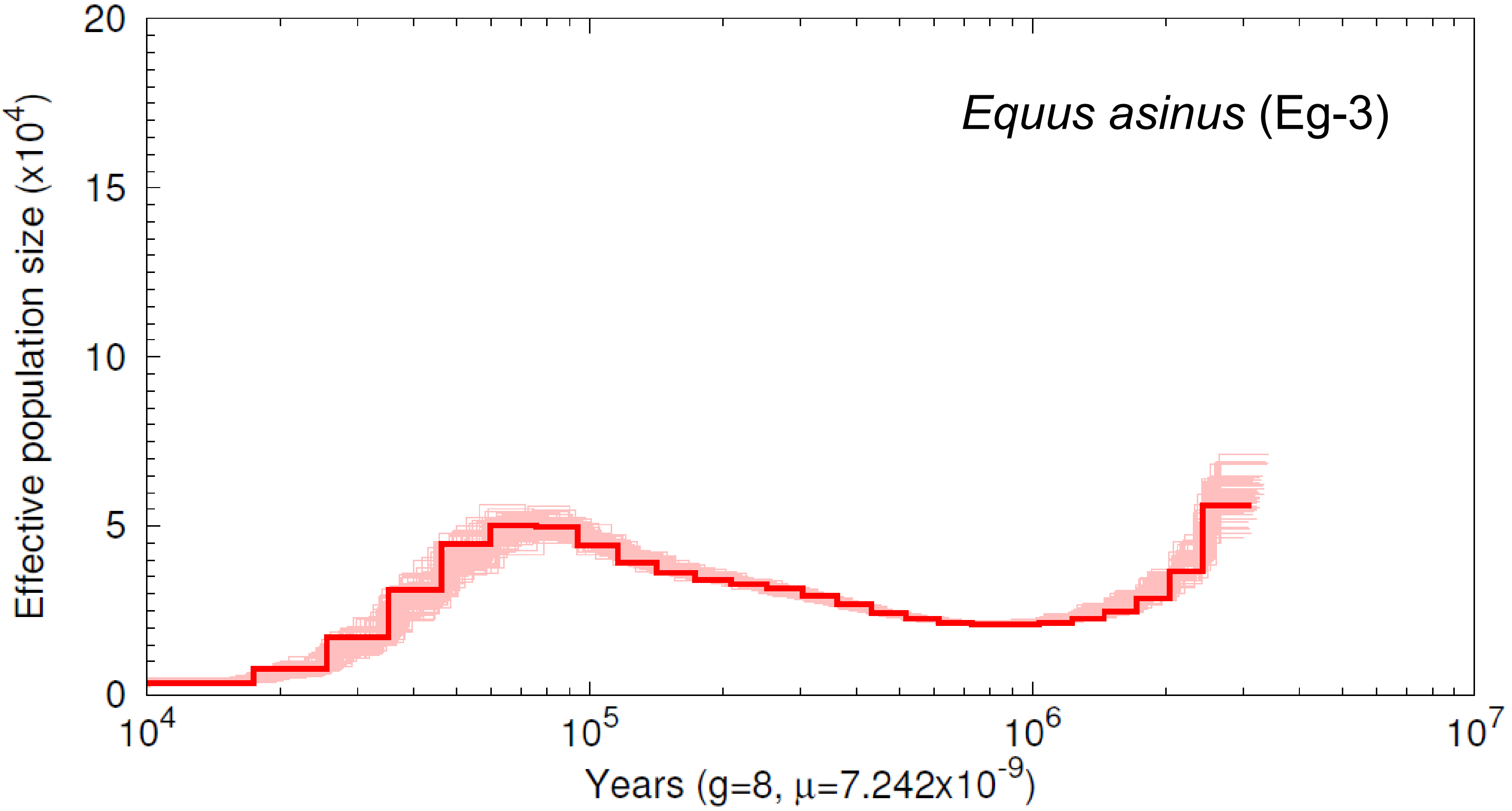
Equus asinus (Eg-3)
Effective population size (x10^4)
20
15
10
5
0
10^4
10^5
10^6
10^7
Years (g=8, μ=7.242x10^-9)

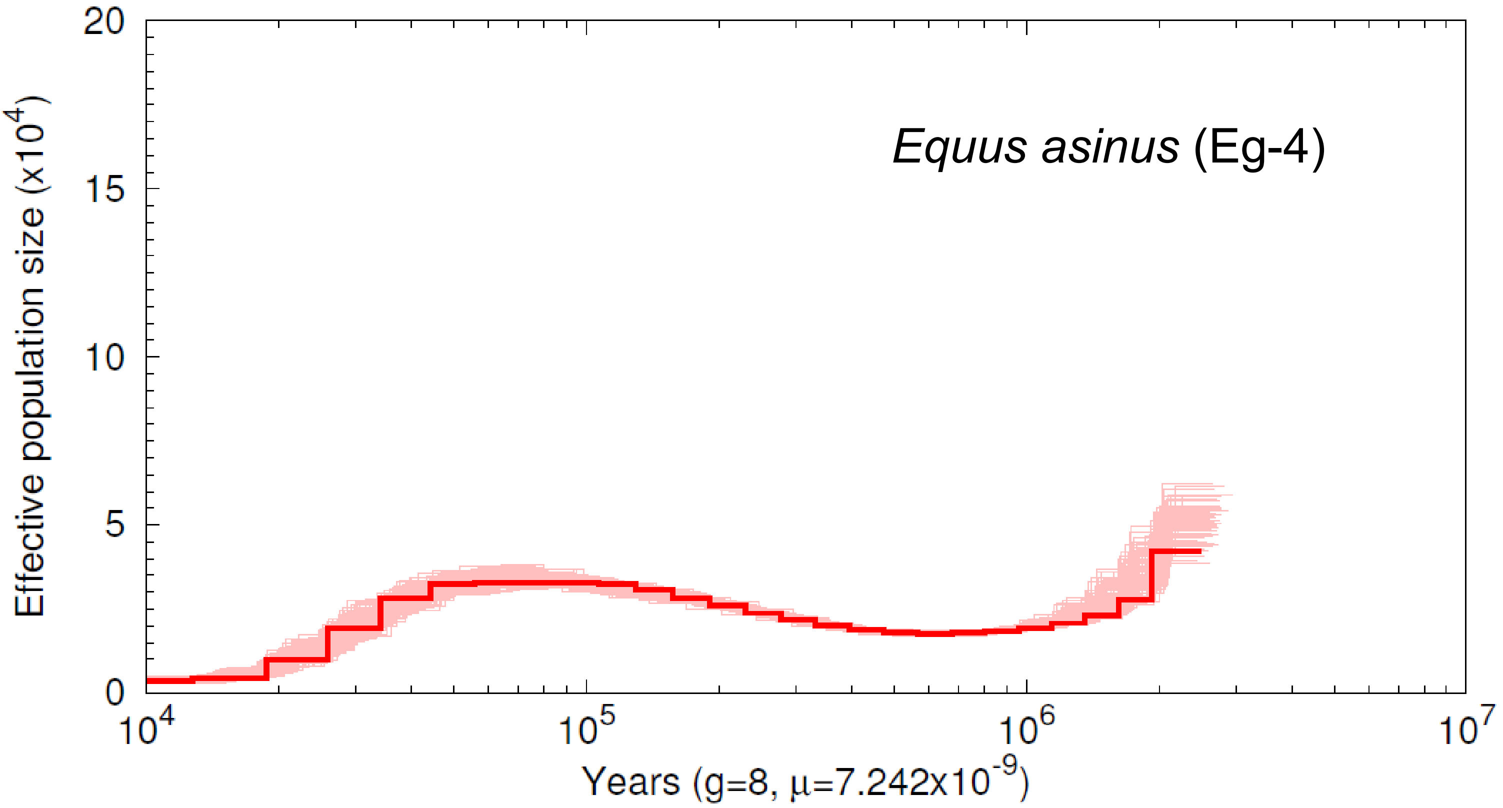

Equus asinus (Eg-4)
Effective population size (x10^4)
20
15
10
5
0
10^4
10^5
10^6
10^7
Years (g=8, μ=7.242x10^-9)


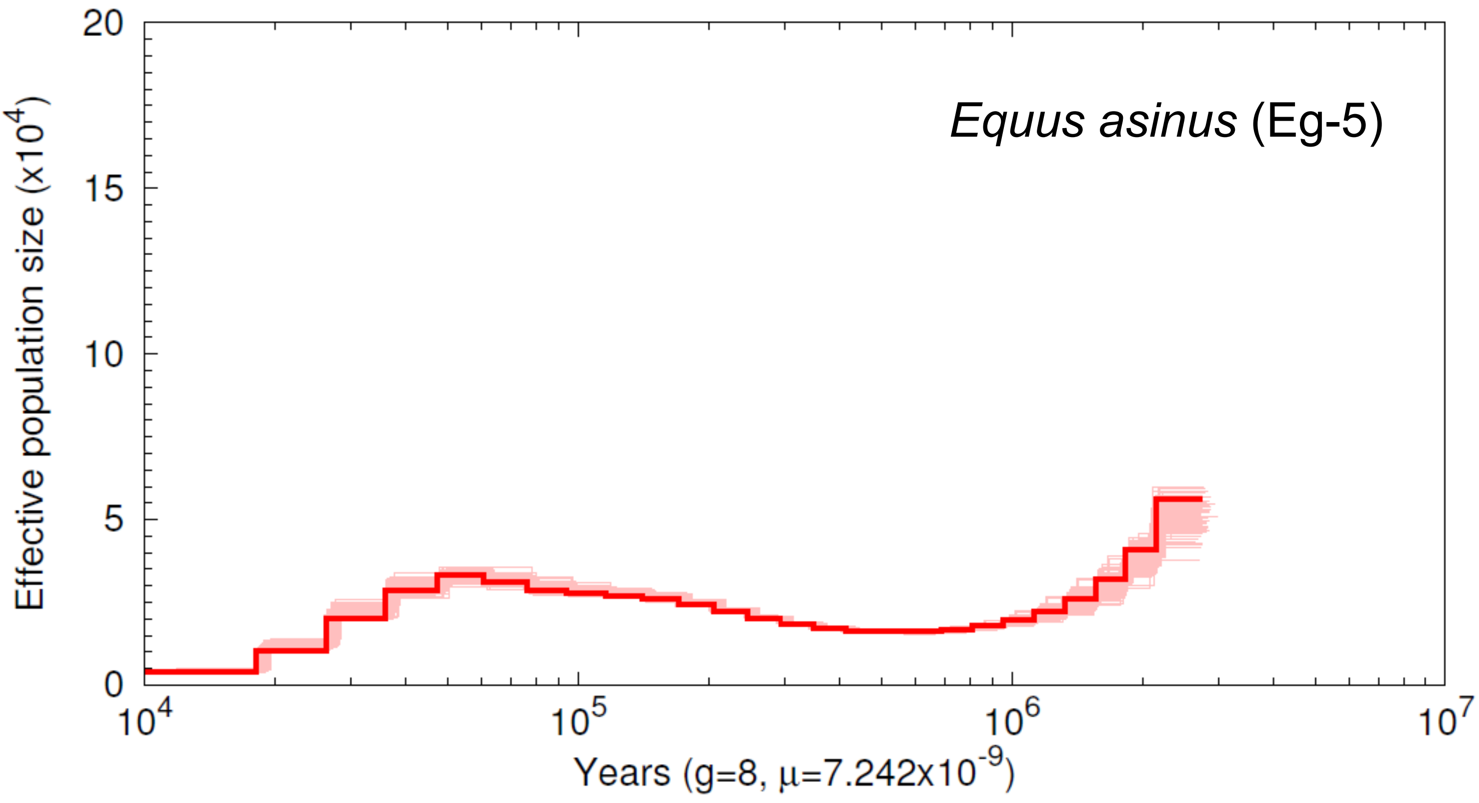

Equus asinus (Eg-5)
Effective population size (x10^4)
20
15
10
5
0
10^4
10^5
10^6
10^7
Years (g=8, μ=7.242x10^-9)

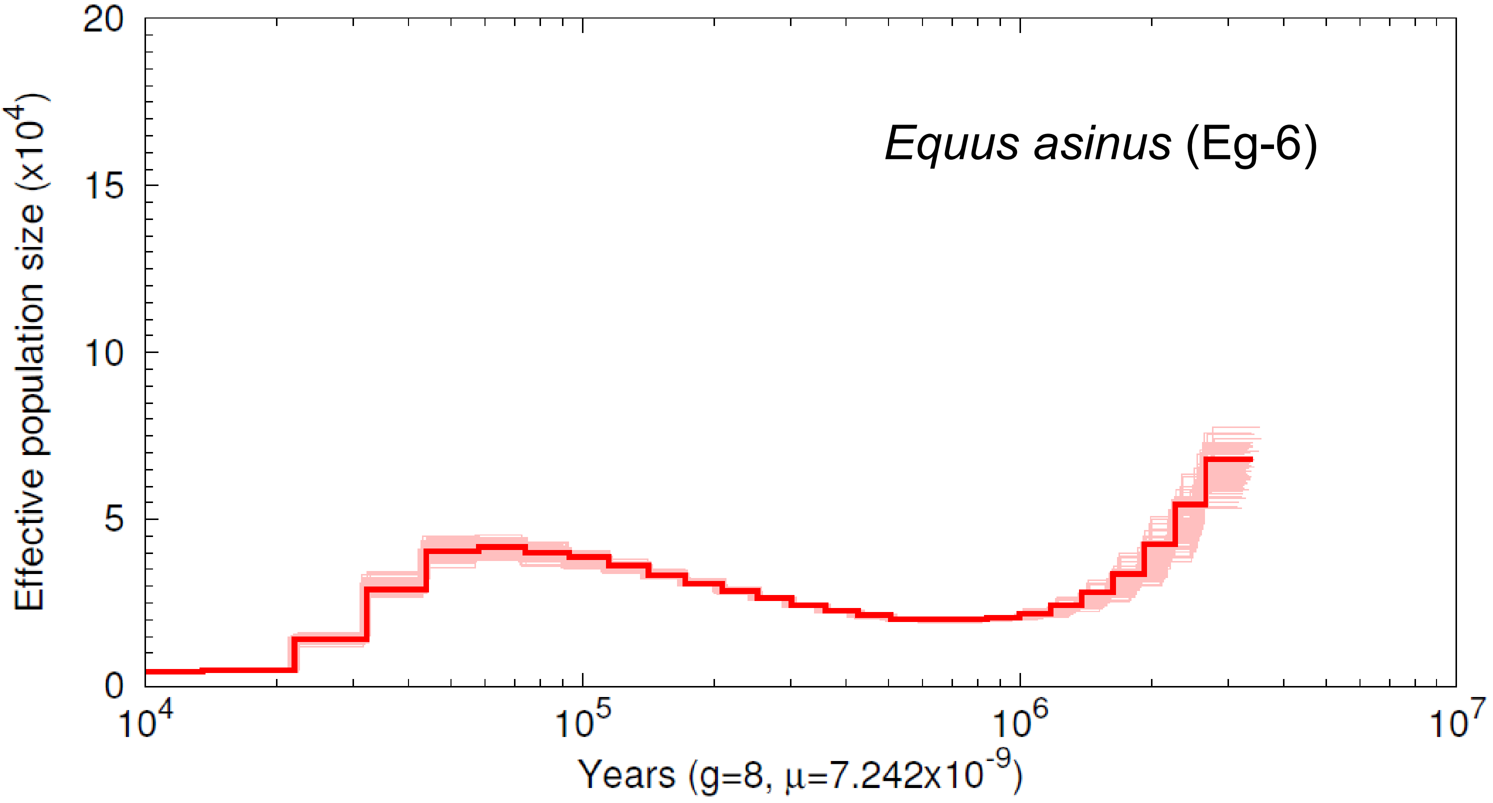

20
15
10
5
0
Effective population size (x10$^4$)
Equus asinus (Eg-6)
10$^4$
10$^5$
10$^6$
10$^7$
Years (g=8, μ=7.242x10$^{-9}$)


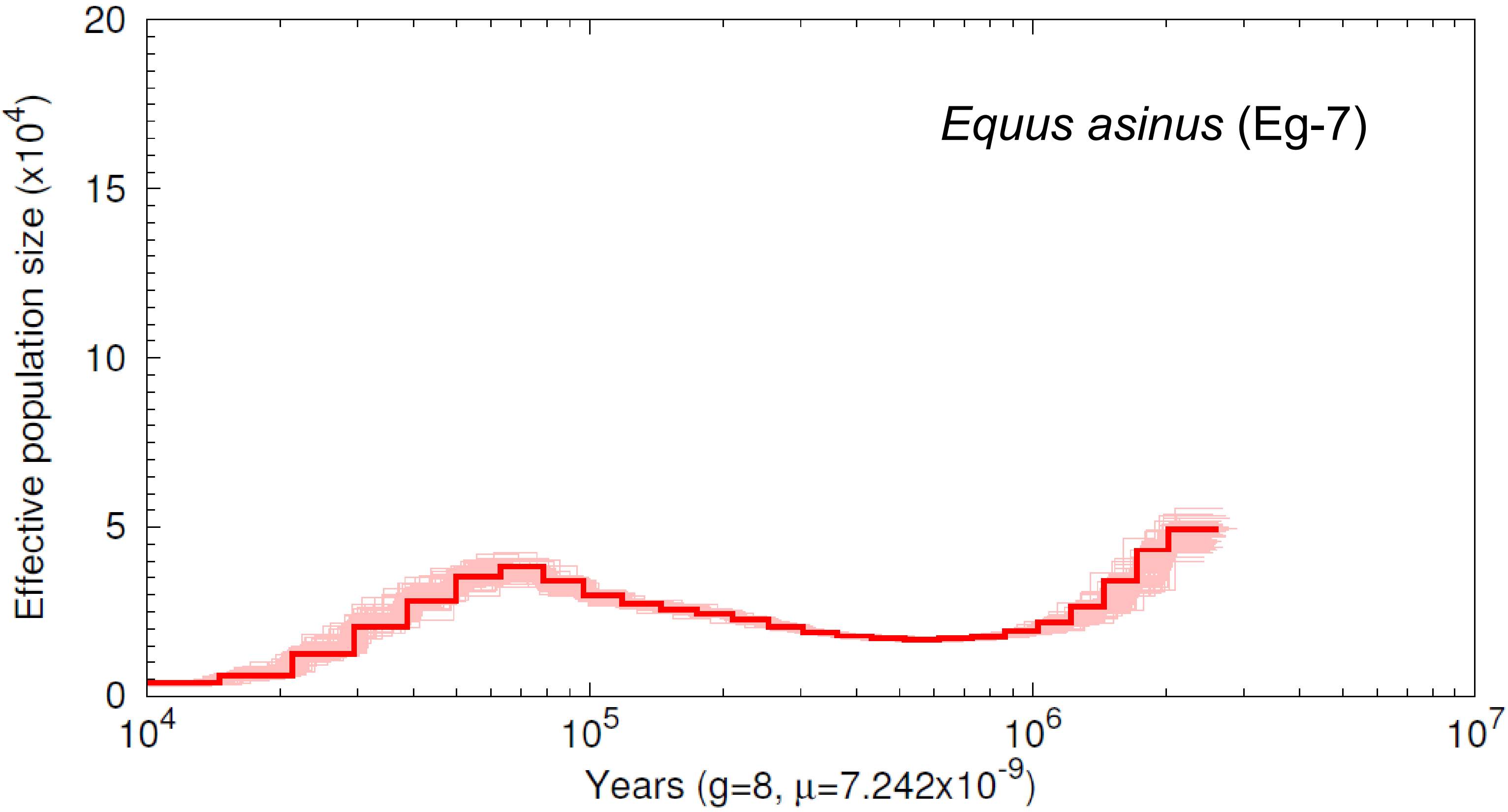

20
15
10
5
0
Effective population size (x10$^4$)
Equus asinus (Eg-7)
10$^4$
10$^5$
10$^6$
10$^7$
Years (g=8, μ=7.242x10$^{-9}$)

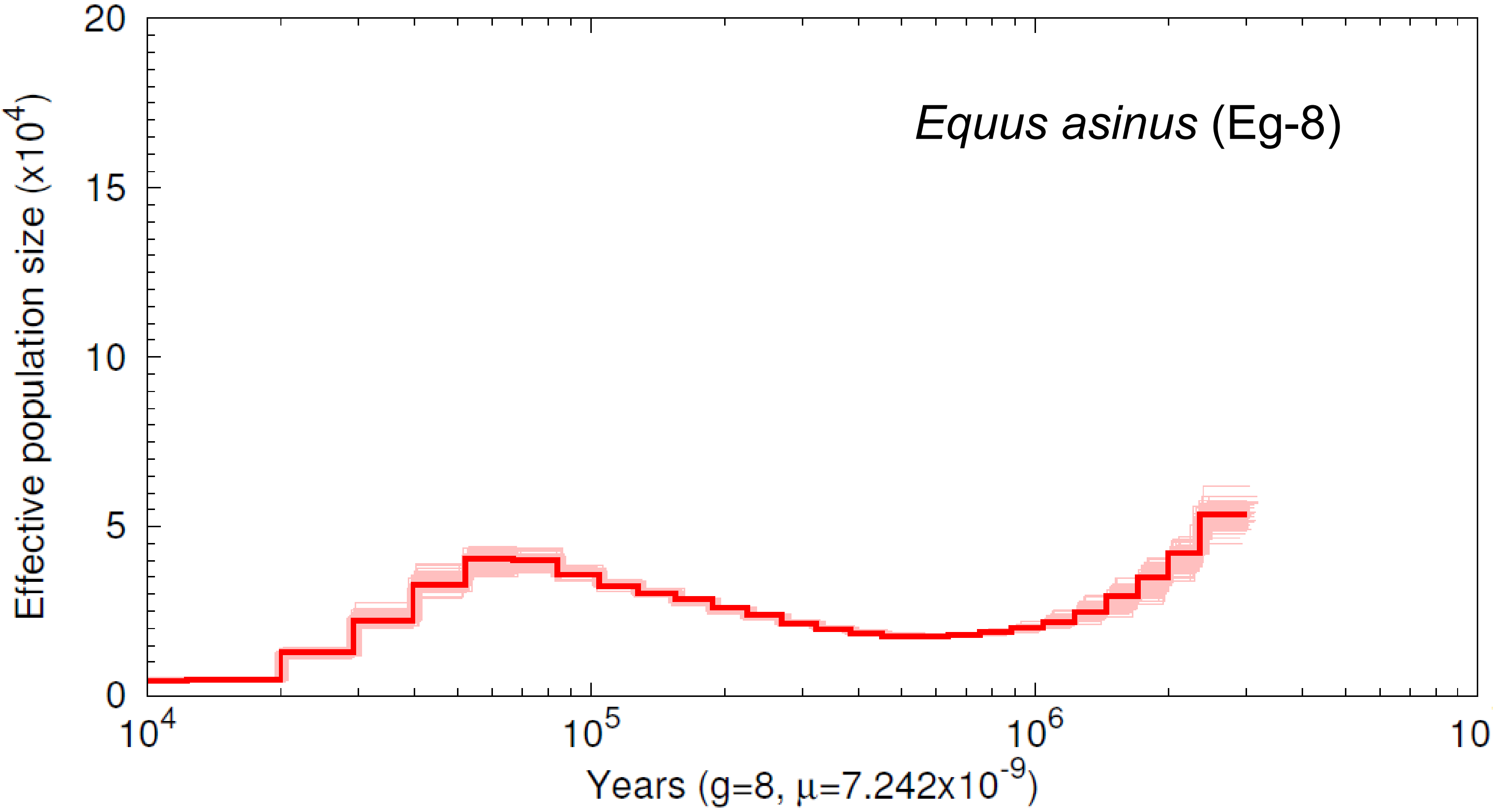

Equus asinus (Eg-8)
Effective population size (x10^4)
20
15
10
5
0
10^4
10^5
10^6
10^7
Years (g=8, μ=7.242x10^-9)


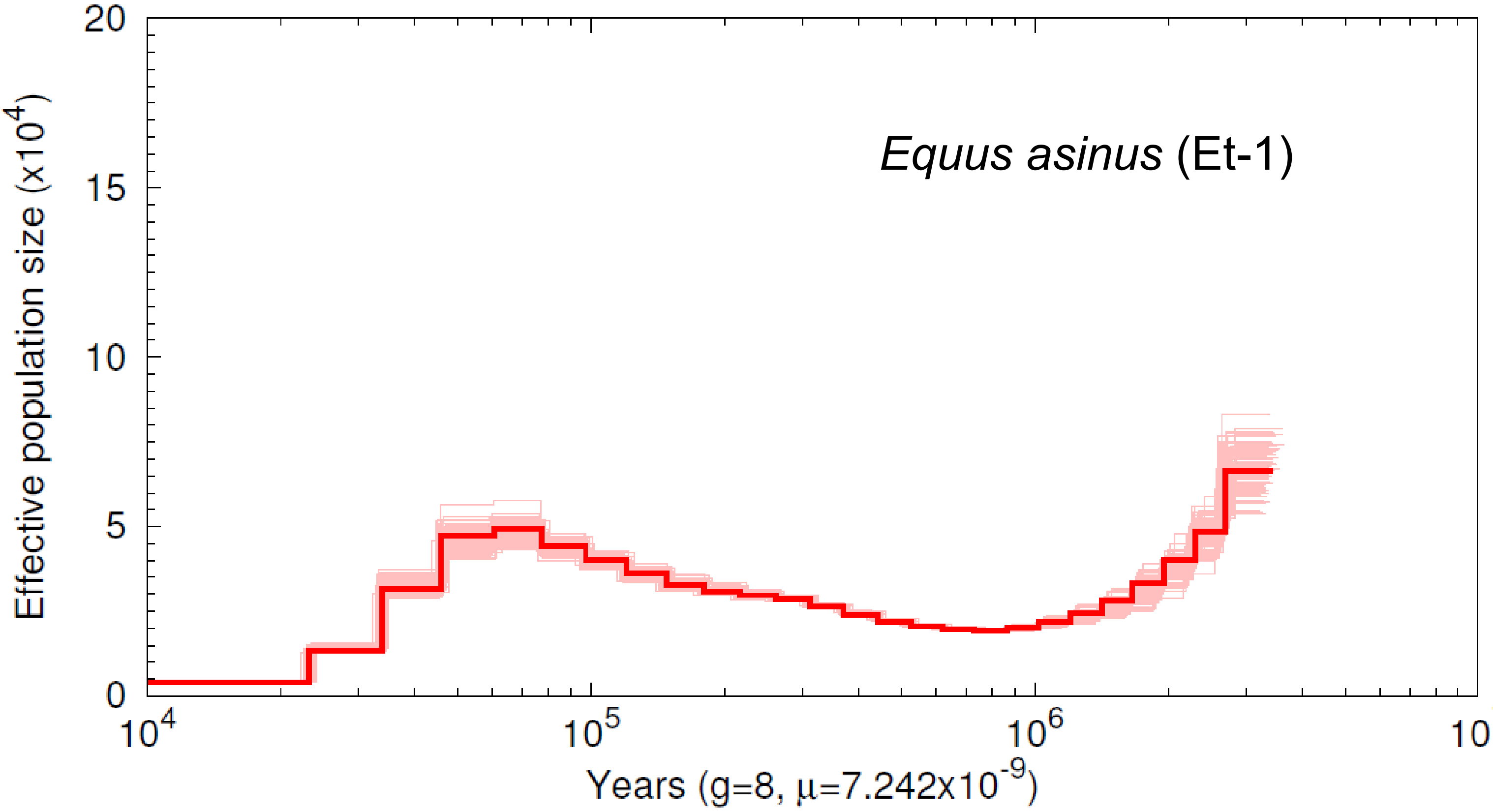

Equus asinus (Et-1)
Effective population size (x10^4)
20
15
10
5
0
10^4
10^5
10^6
10^7
Years (g=8, μ=7.242x10^-9)

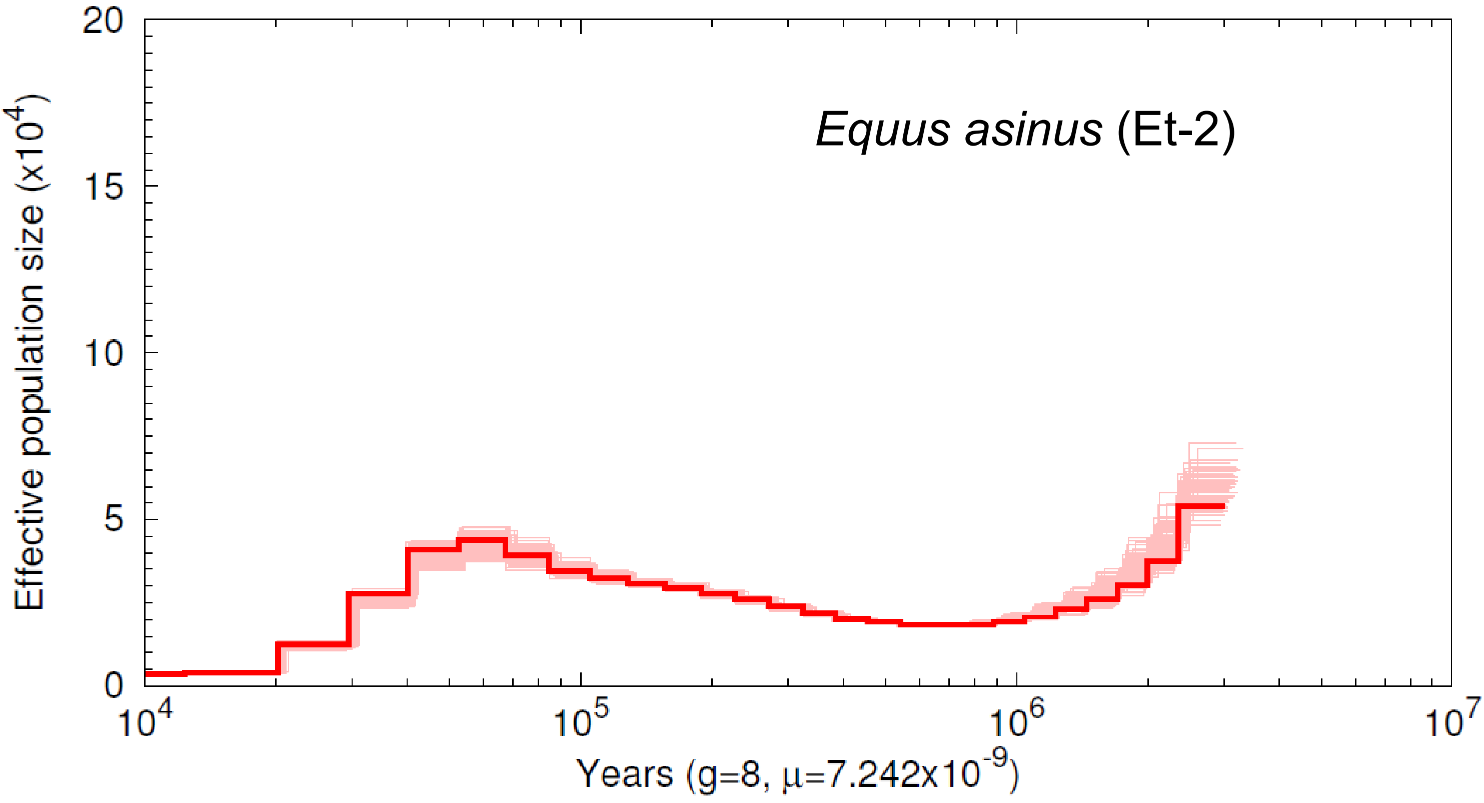
Equus asinus (Et-2)
Effective population size (x10^4)
20
15
10
5
0
10^4
10^5
10^6
10^7
Years (g=8, μ=7.242x10^-9)

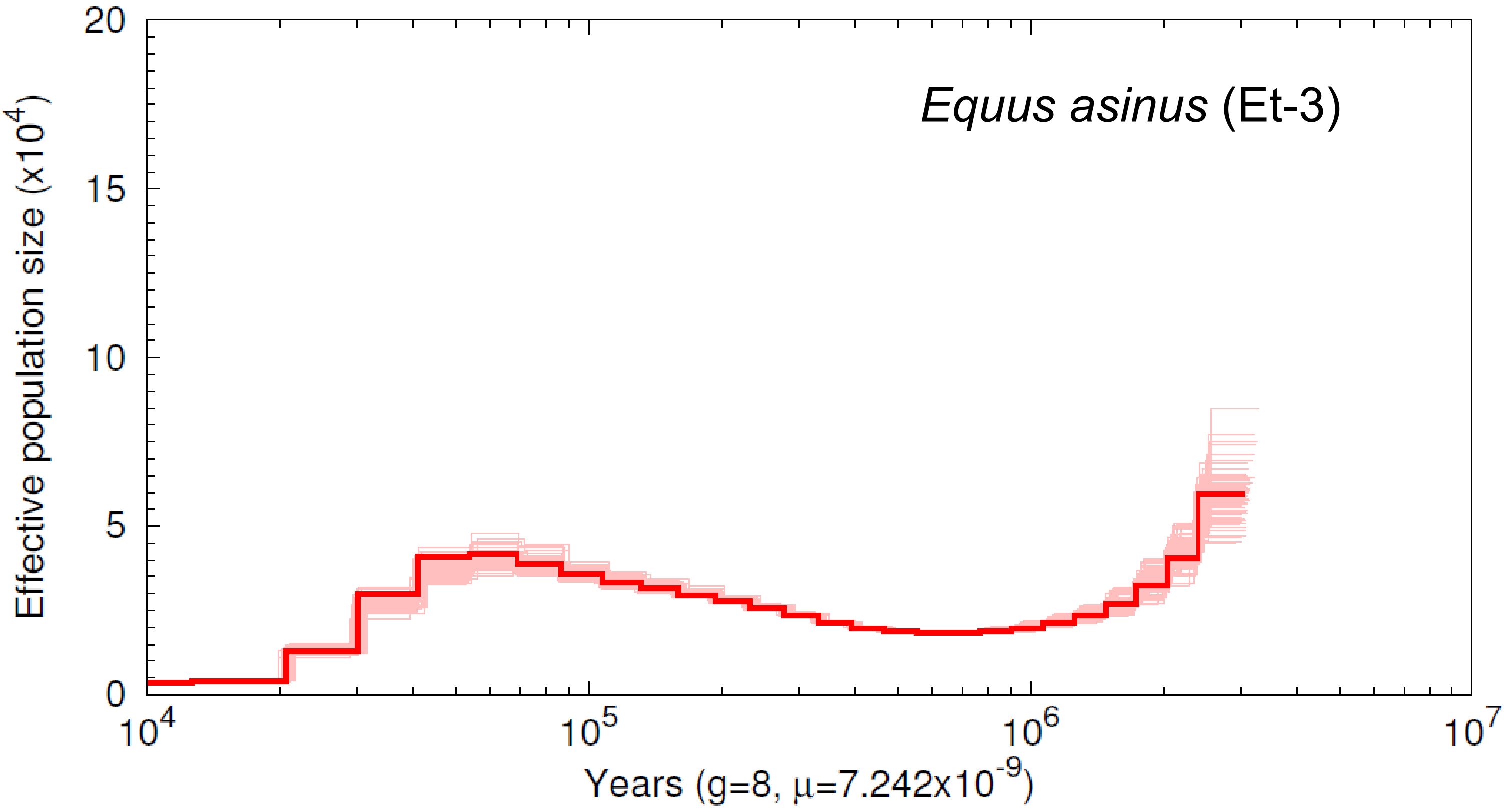
Equus asinus (Et-3)
Effective population size (x10^4)
20
15
10
5
0
10^4
10^5
10^6
10^7
Years (g=8, μ=7.242x10^-9)

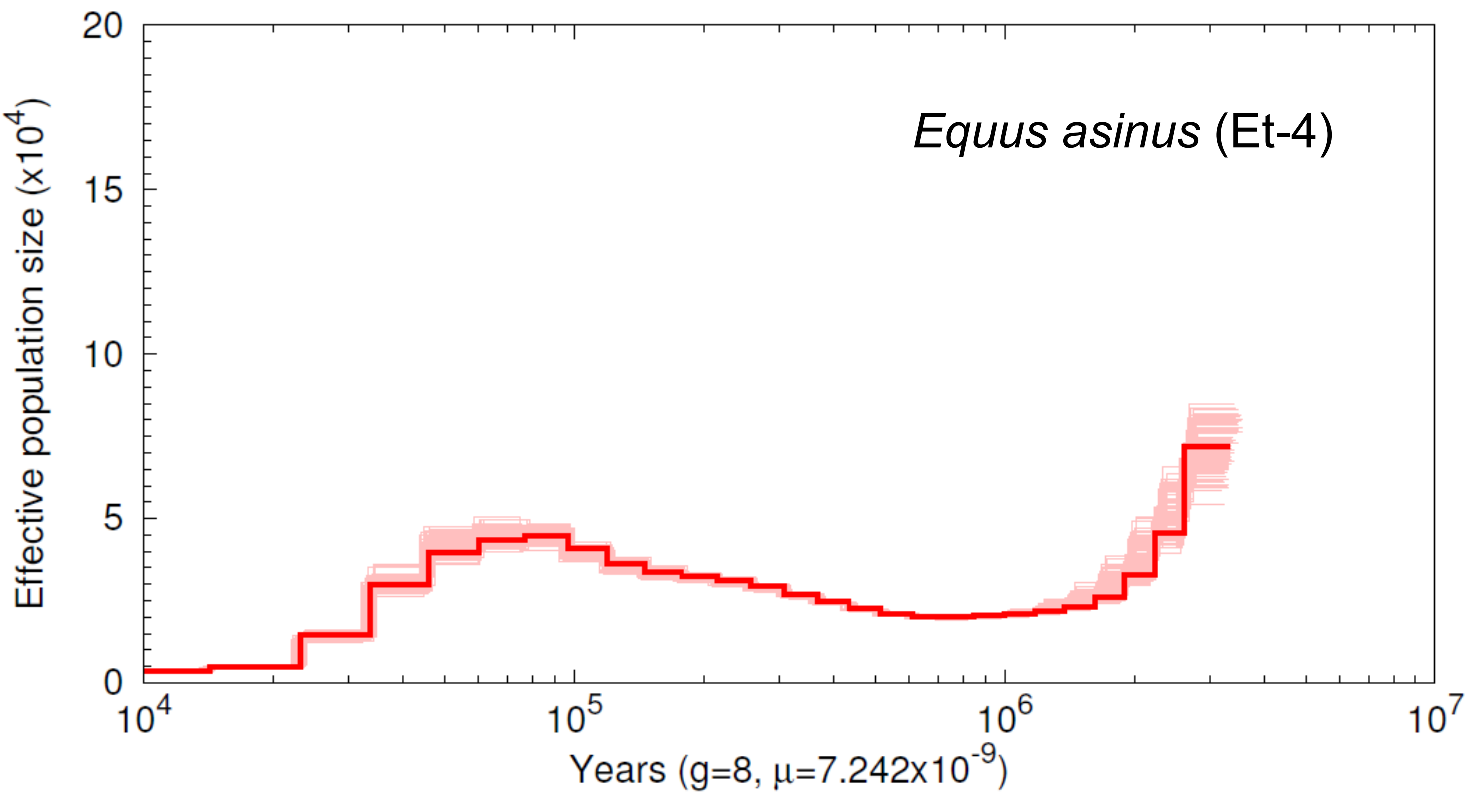
Equus asinus (Et-4)
Effective population size (x10^4)
20
15
10
5
0
10^4
10^5
10^6
10^7
Years (g=8, μ=7.242x10^-9)

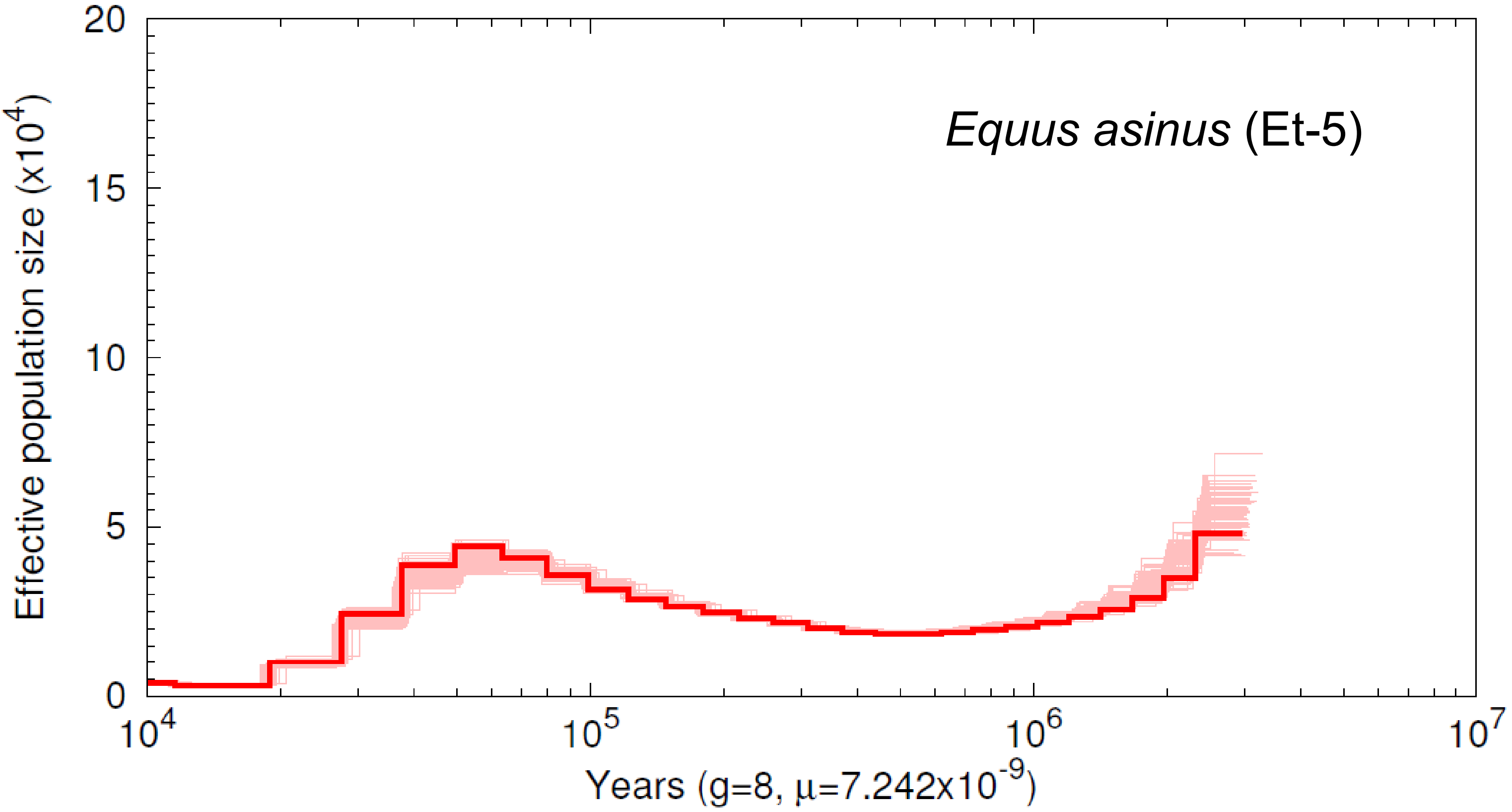
Equus asinus (Et-5)
Effective population size (x10^4)
20
15
10
5
0
10^4
10^5
10^6
10^7
Years (g=8, μ=7.242x10^-9)

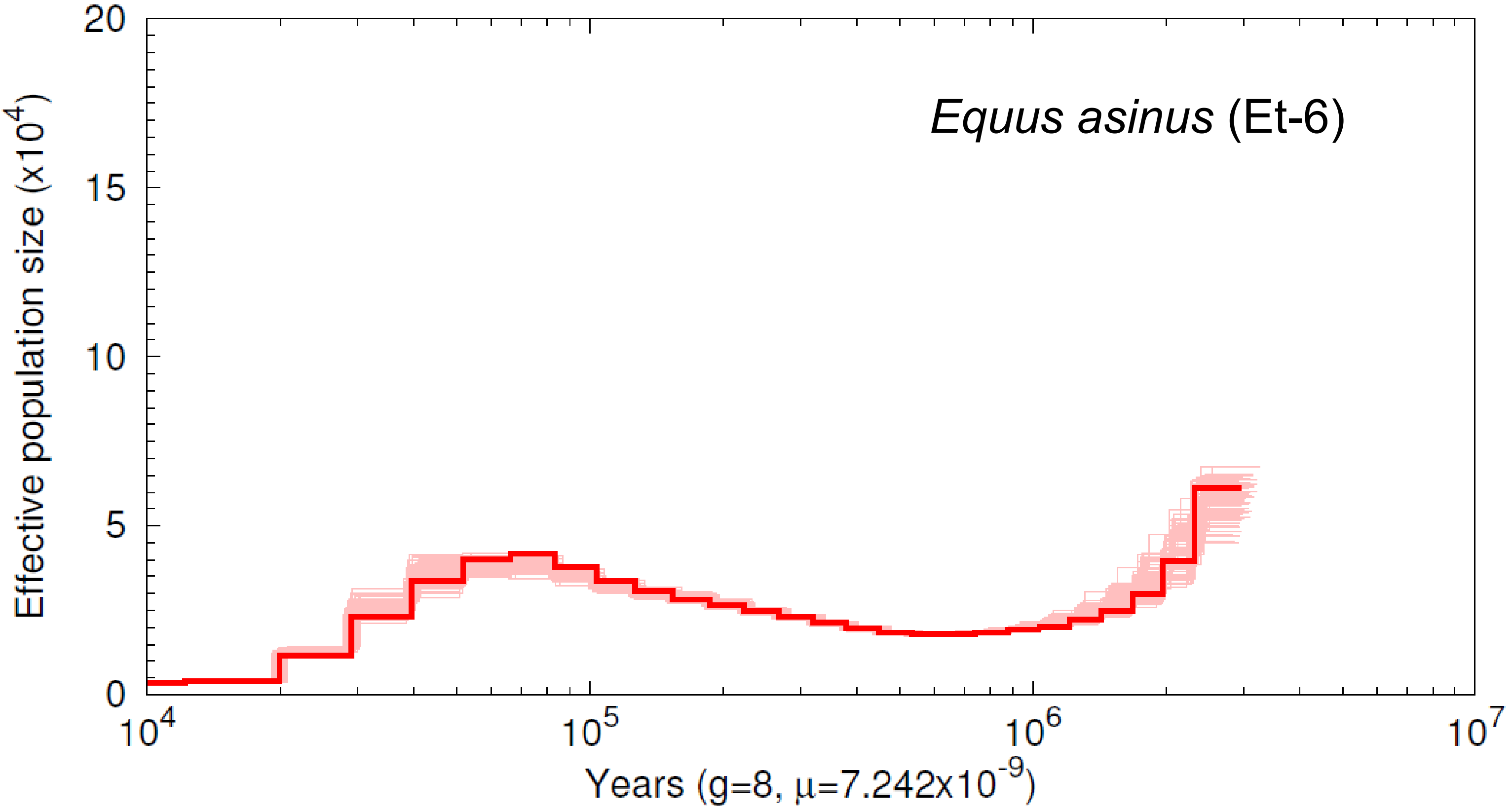

Equus asinus (Et-6)
Effective population size (x10^4)
20
15
10
5
0
10^4
10^5
10^6
10^7
Years (g=8, μ=7.242x10^-9)


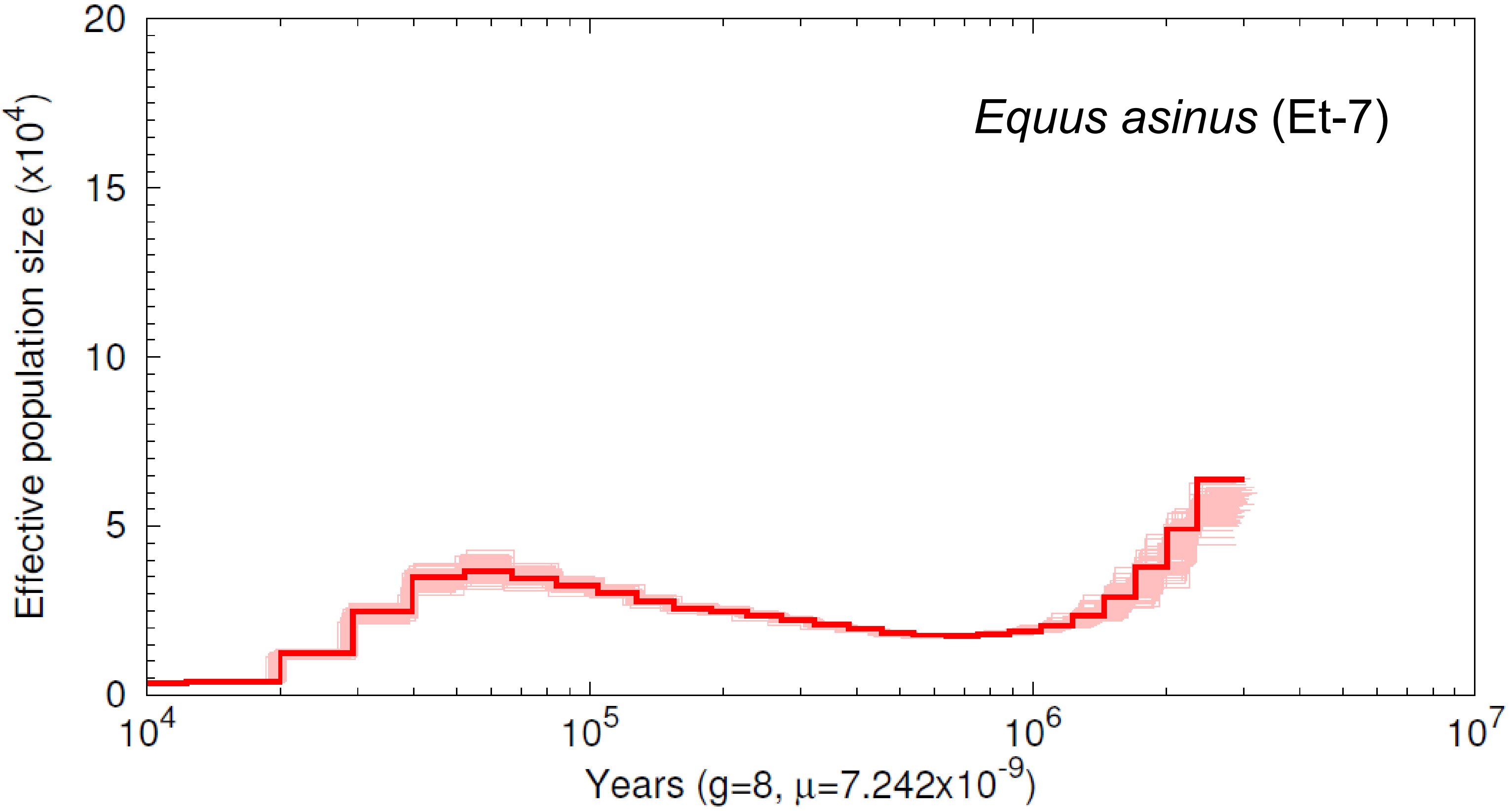

Equus asinus (Et-7)
Effective population size (x10^4)
20
15
10
5
0
10^4
10^5
10^6
10^7
Years (g=8, μ=7.242x10^-9)

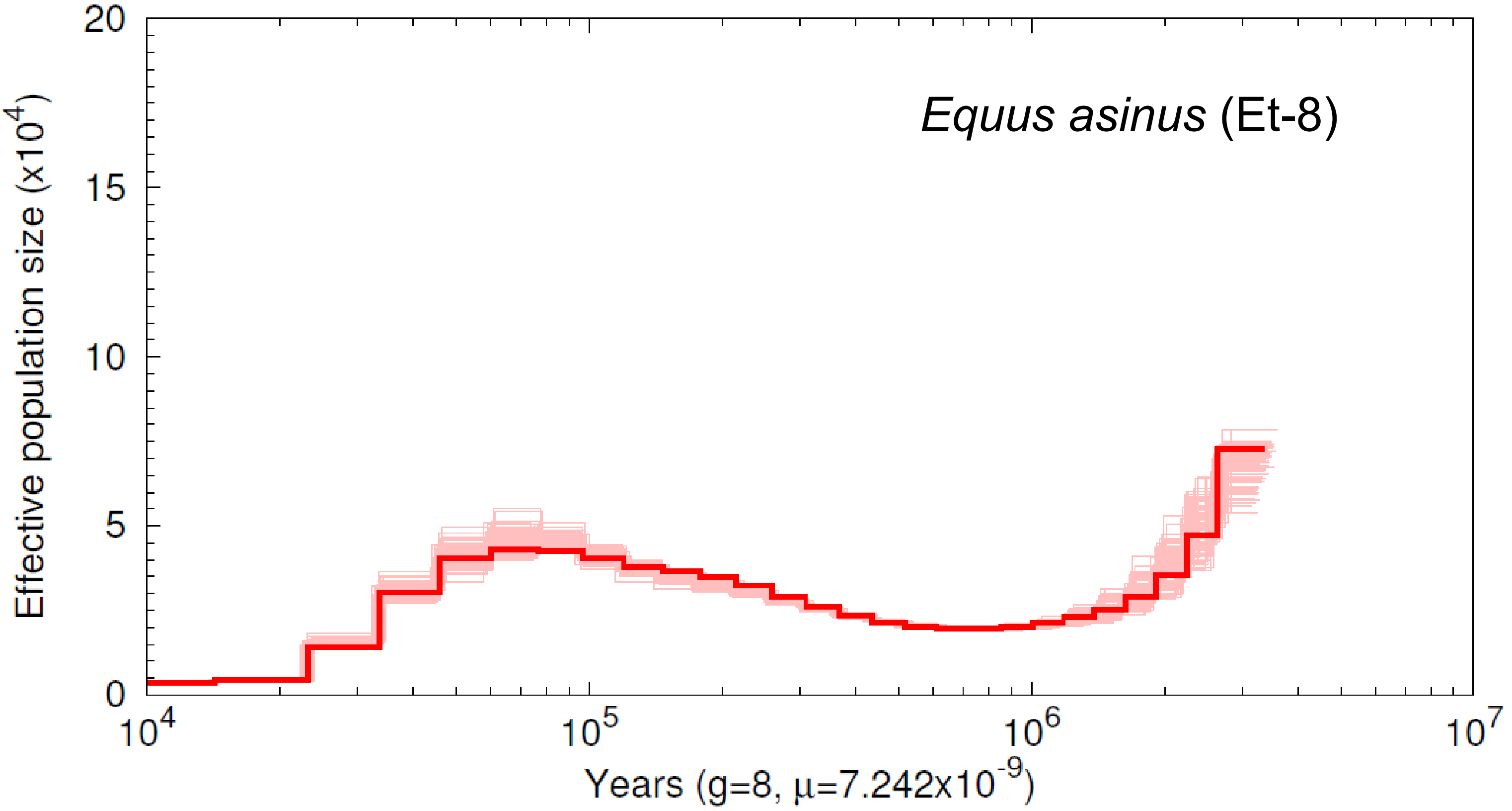
Equus asinus (Et-8)
Effective population size (x10^4)
Years (g=8, μ=7.242x10^-9)
20
15
10
5
0
10^4
10^5
10^6
10^7

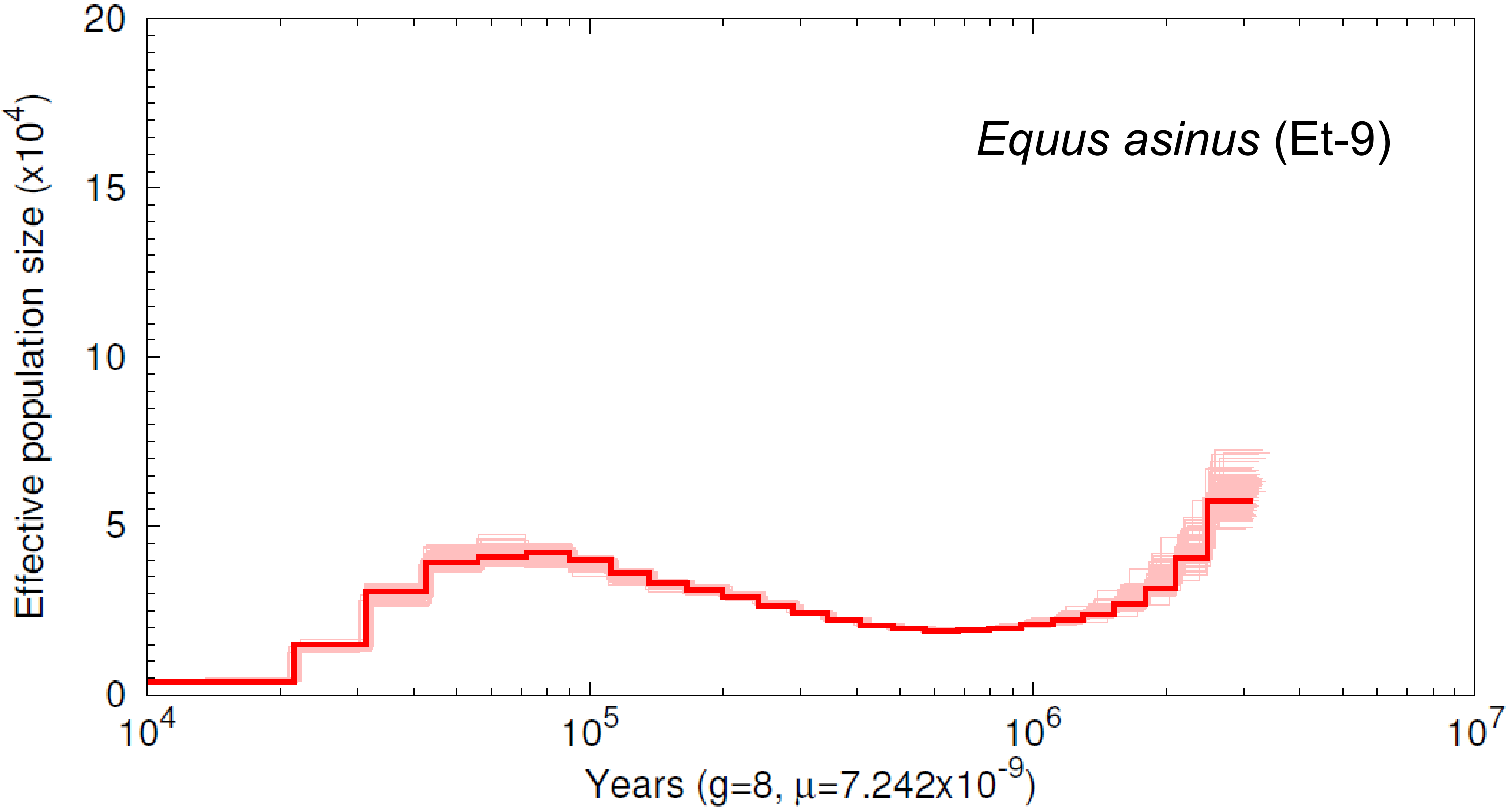
Equus asinus (Et-9)
Effective population size (x10^4)
Years (g=8, μ=7.242x10^-9)
20
15
10
5
0
10^4
10^5
10^6
10^7

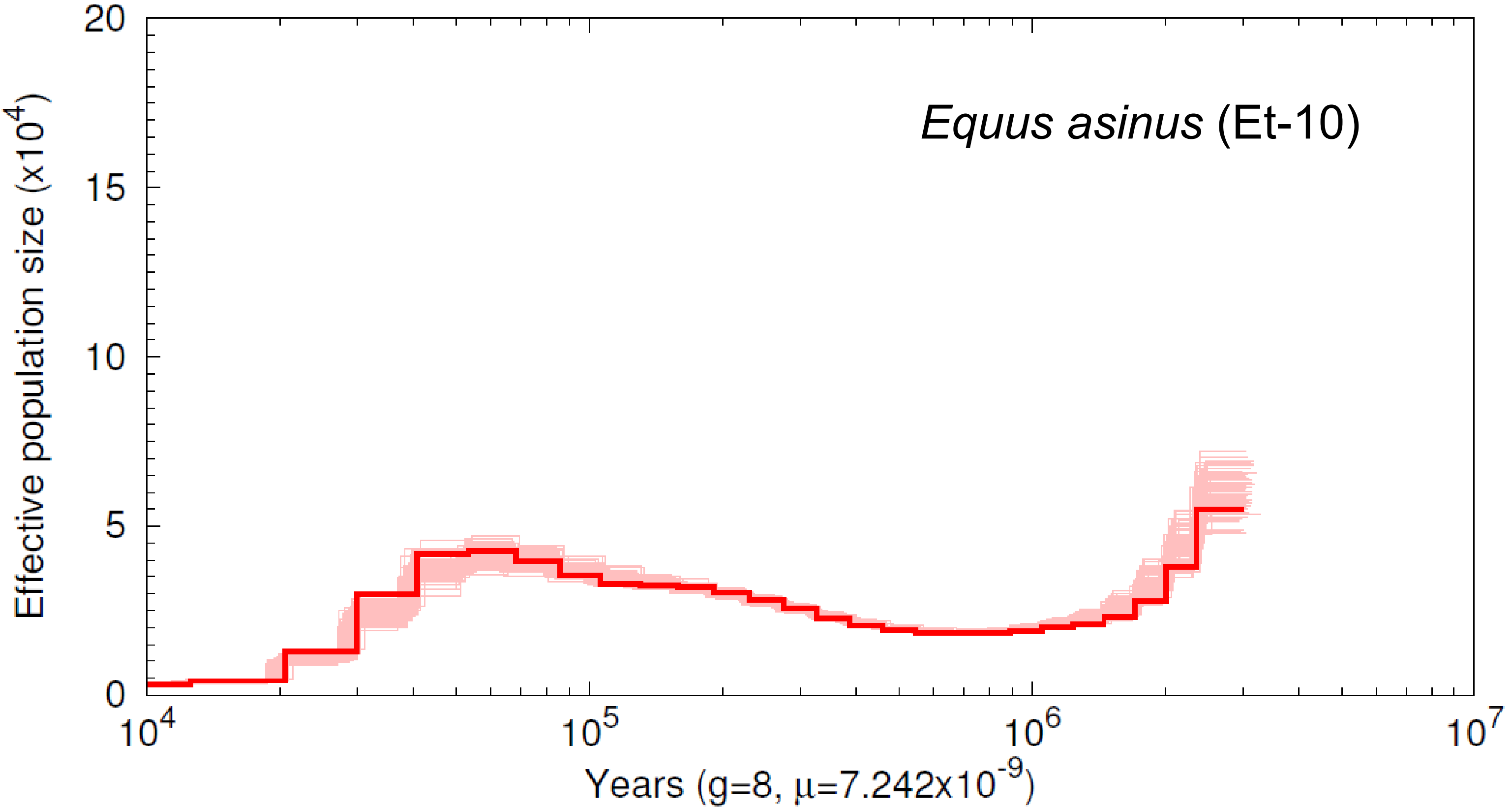
Equus asinus (Et-10)
Effective population size (x10^4)
Years (g=8, μ=7.242x10^-9)
20
15
10
5
0
10^4
10^5
10^6
10^7

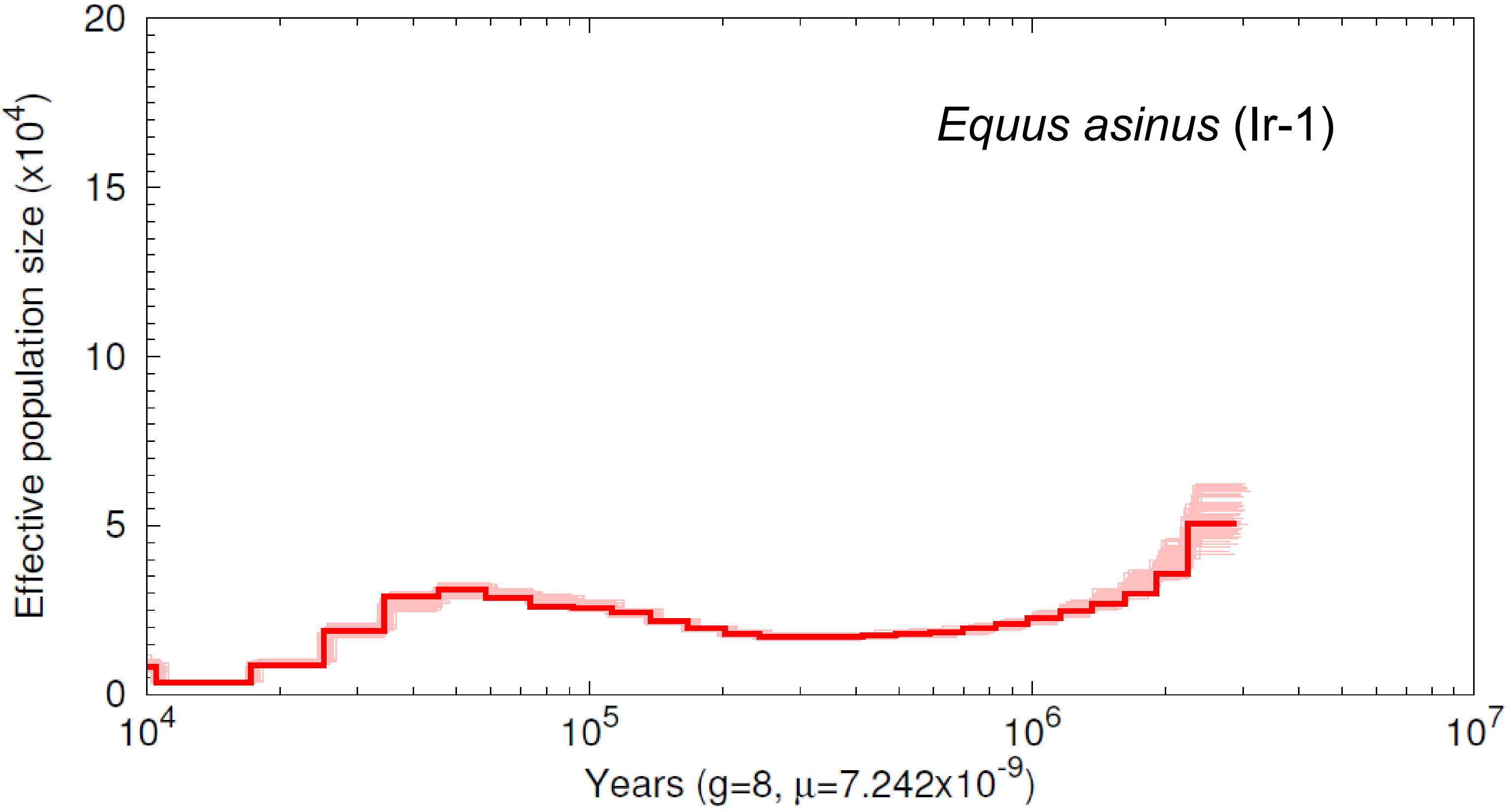
Equus asinus (Ir-1)
Effective population size (x10^4)
Years (g=8, μ=7.242x10^-9)
20
15
10
5
0
10^4
10^5
10^6
10^7

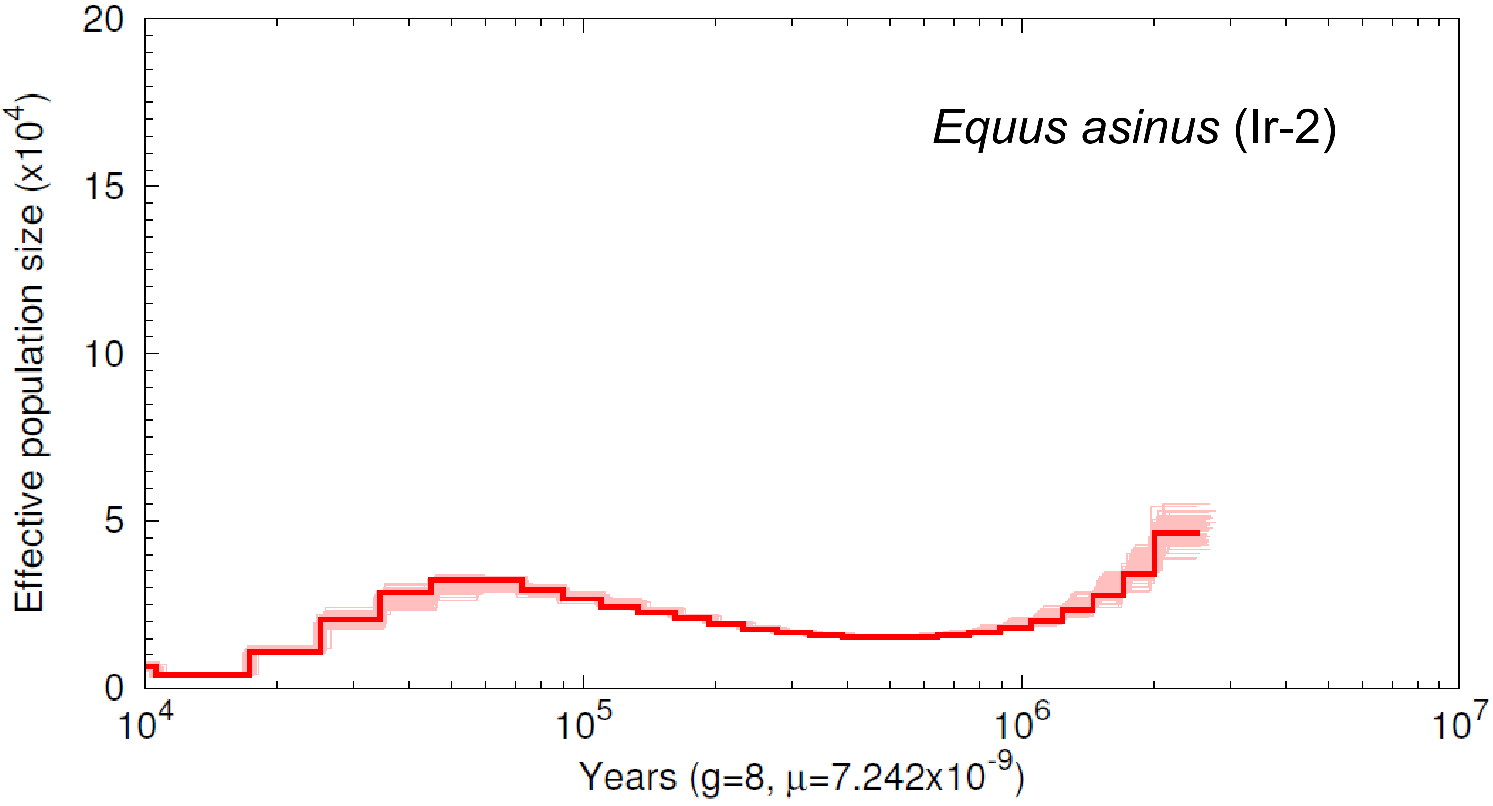
Equus asinus (Ir-2)
Effective population size (x10^4)
Years (g=8, μ=7.242x10^-9)
0
5
10
15
20
10^4
10^5
10^6
10^7

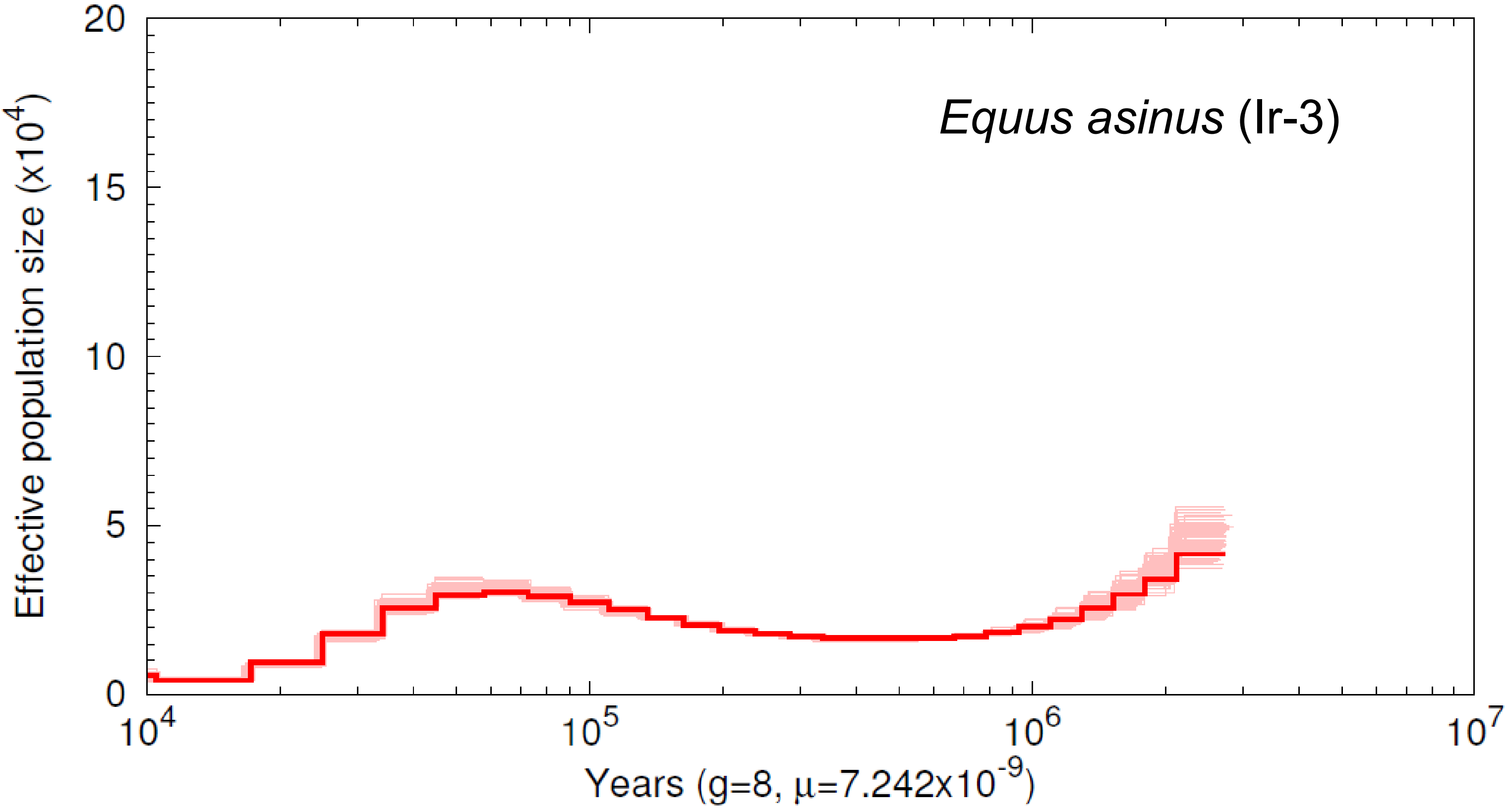
Equus asinus (Ir-3)
Effective population size (x10^4)
Years (g=8, μ=7.242x10^-9)
0
5
10
15
20
10^4
10^5
10^6
10^7

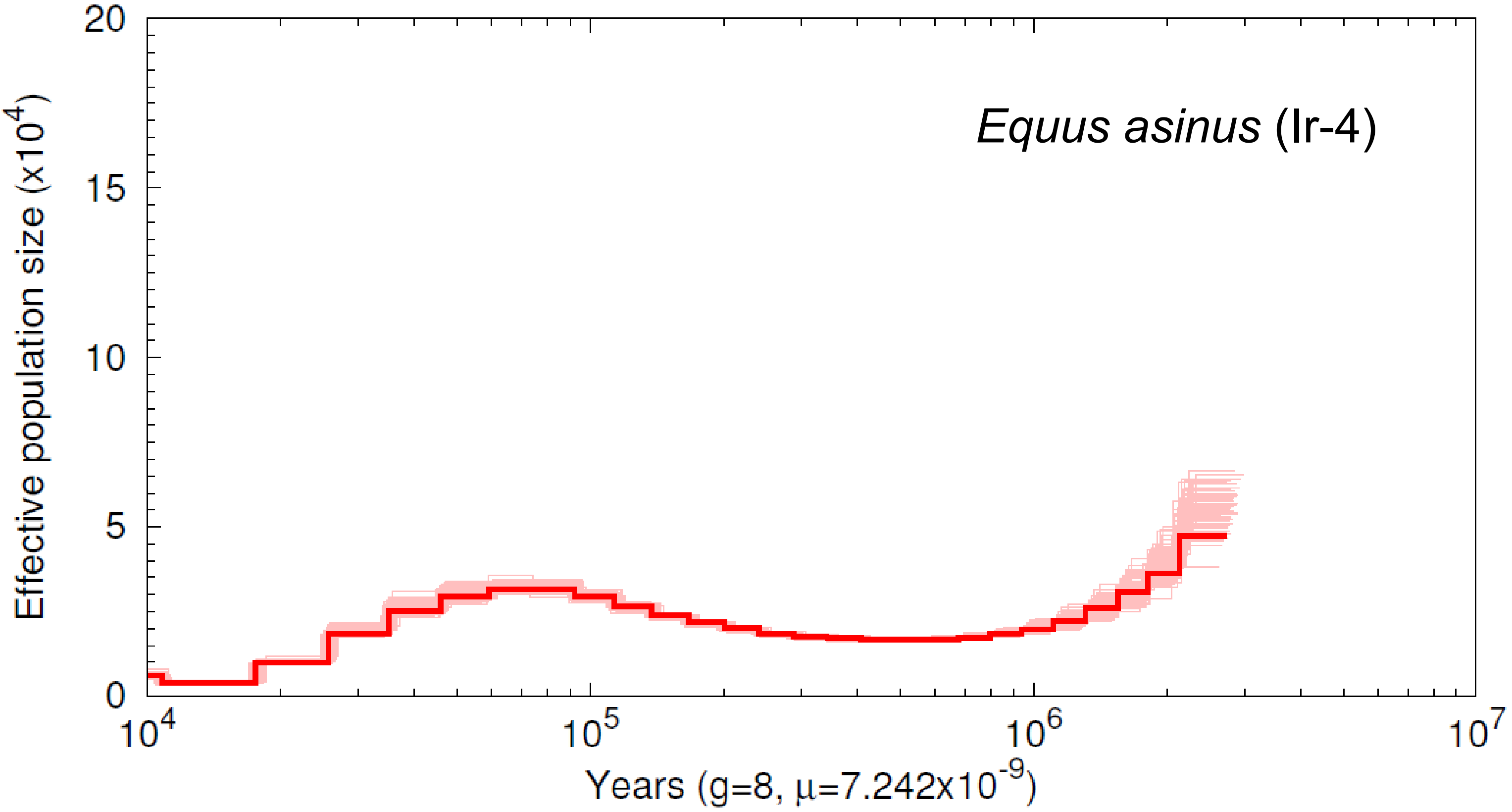
Equus asinus (Ir-4)
Effective population size (x10^4)
Years (g=8, μ=7.242x10^-9)
0
5
10
15
20
10^4
10^5
10^6
10^7

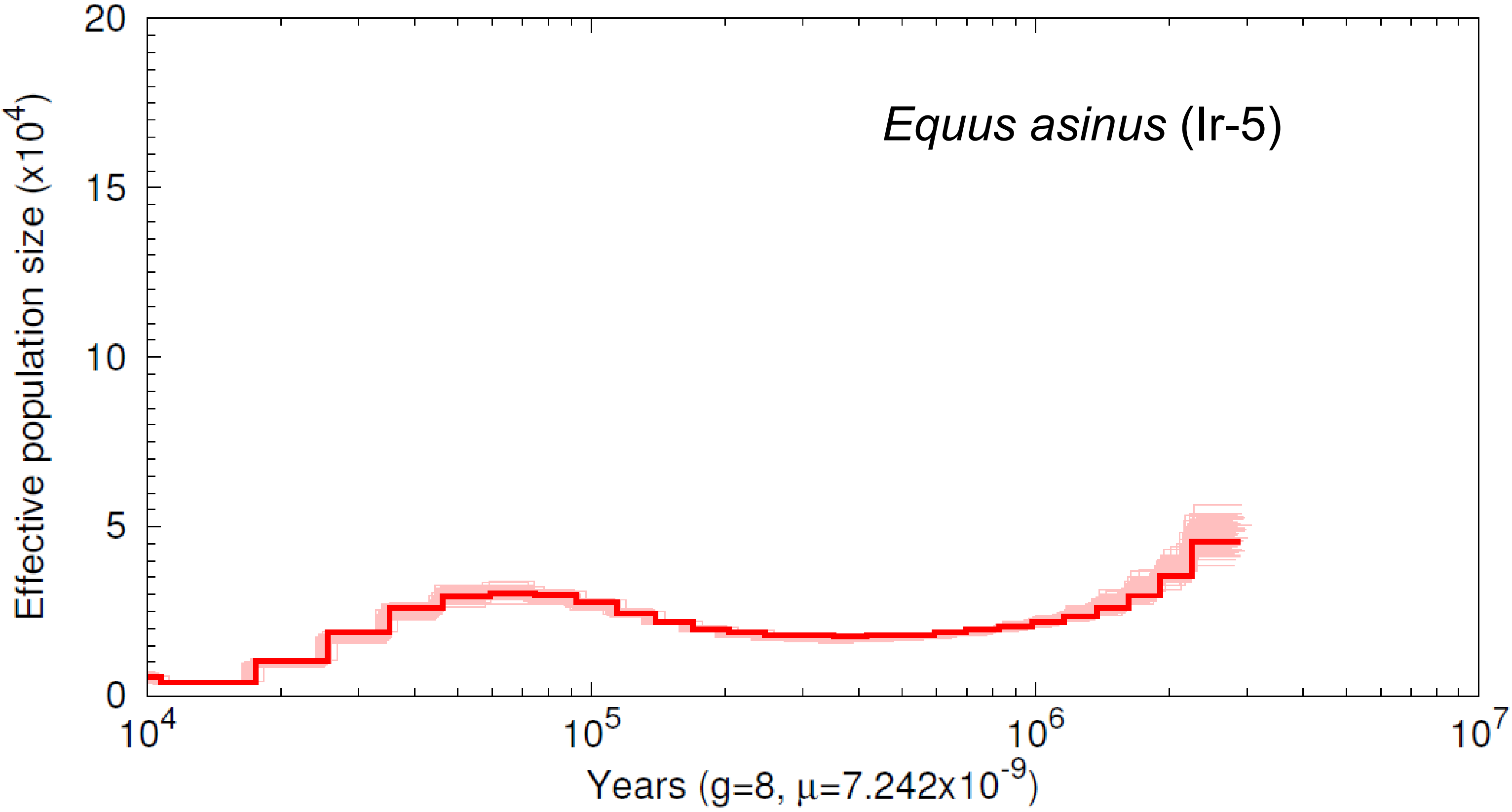
Equus asinus (Ir-5)
Effective population size (x10^4)
Years (g=8, μ=7.242x10^-9)
0
5
10
15
20
10^4
10^5
10^6
10^7

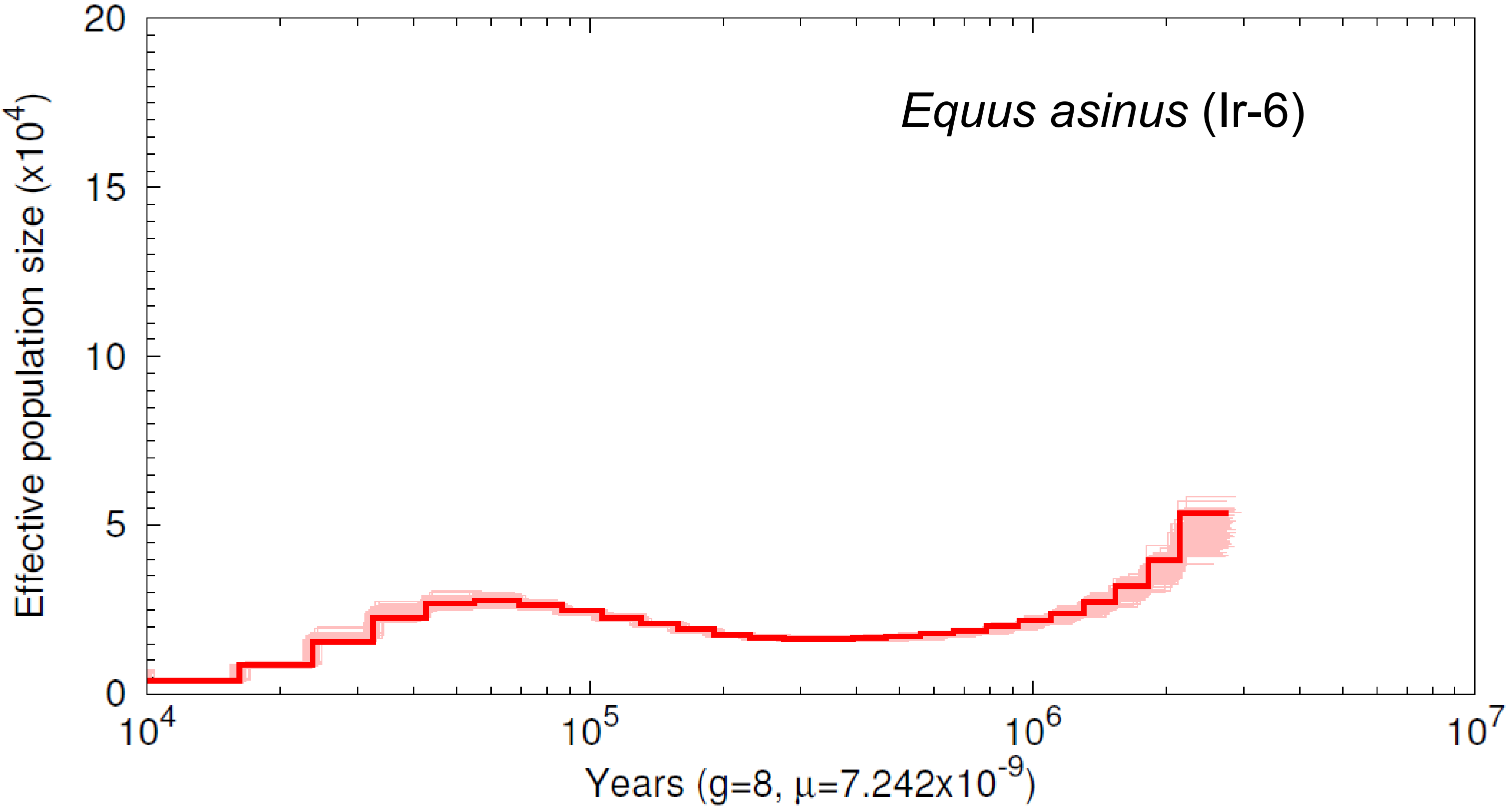

Equus asinus (Ir-6)
Effective population size (x10^4)
Years (g=8, μ=7.242x10^-9)


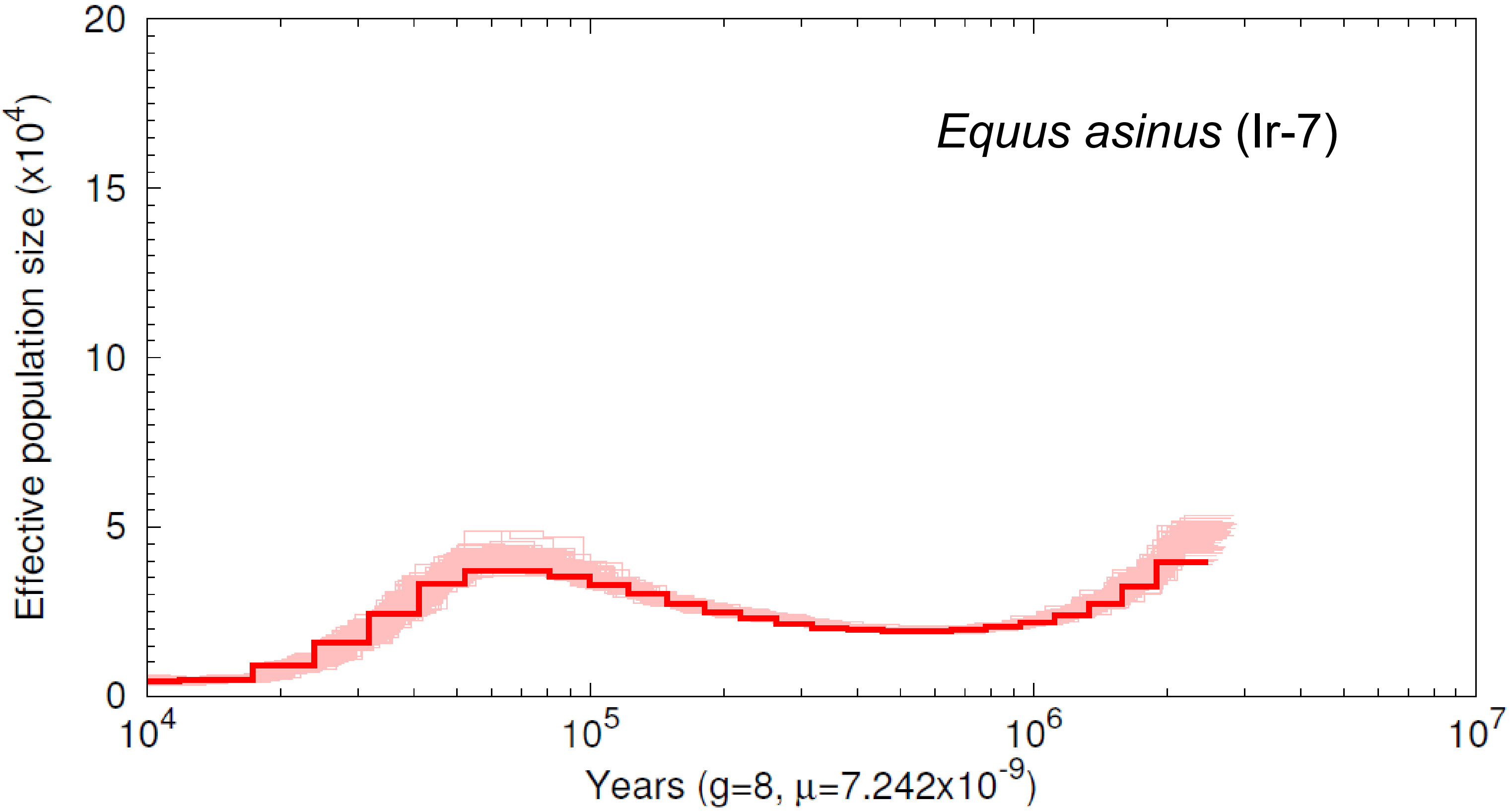

Equus asinus (Ir-7)
Effective population size (x10^4)
Years (g=8, μ=7.242x10^-9)

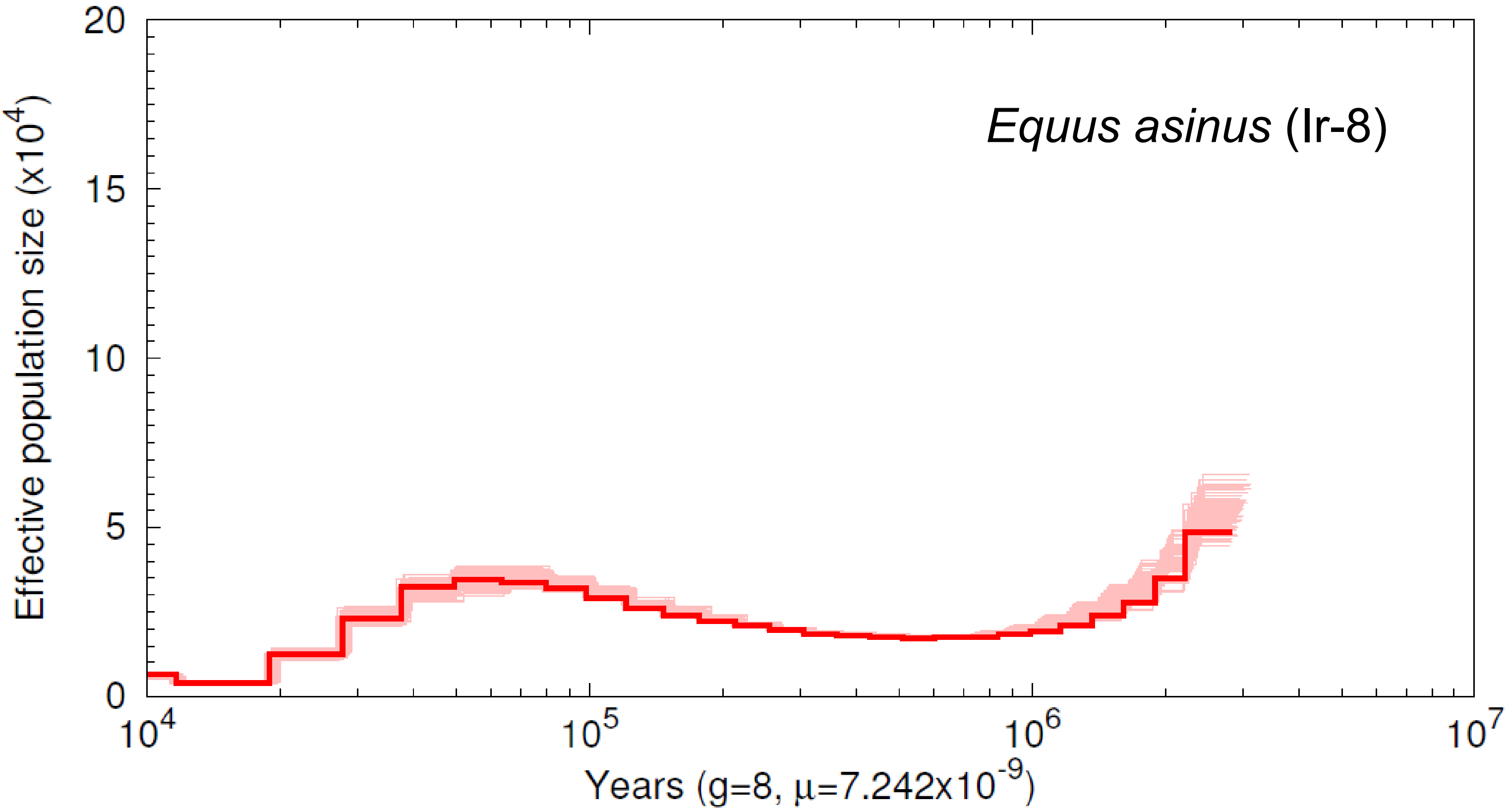

Equus asinus (Ir-8)
Effective population size (x10^4)
20
15
10
5
0
10^4
10^5
10^6
10^7
Years (g=8, μ=7.242x10^-9)


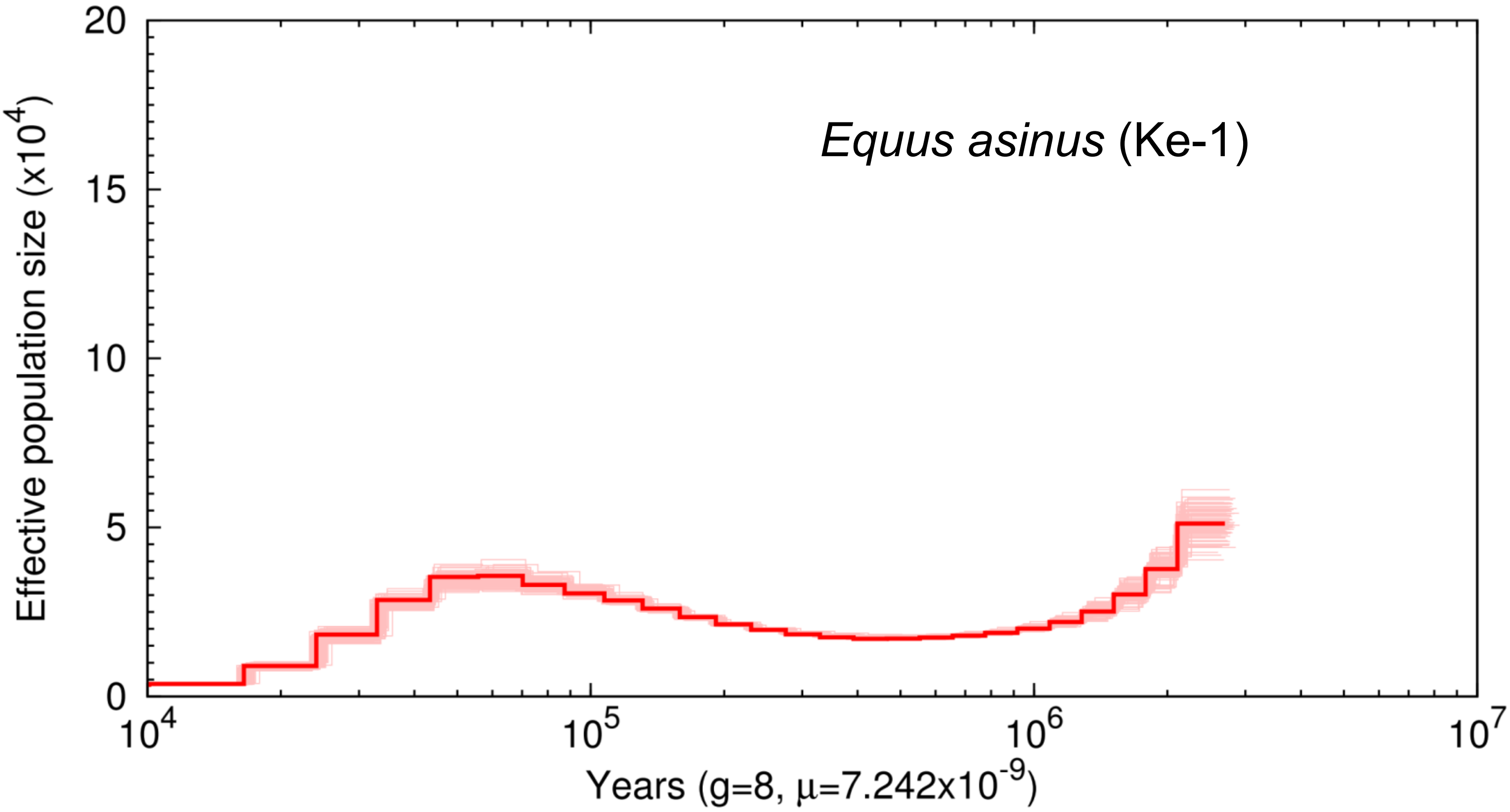

Equus asinus (Ke-1)
Effective population size (x10^4)
20
15
10
5
0
10^4
10^5
10^6
10^7
Years (g=8, μ=7.242x10^-9)

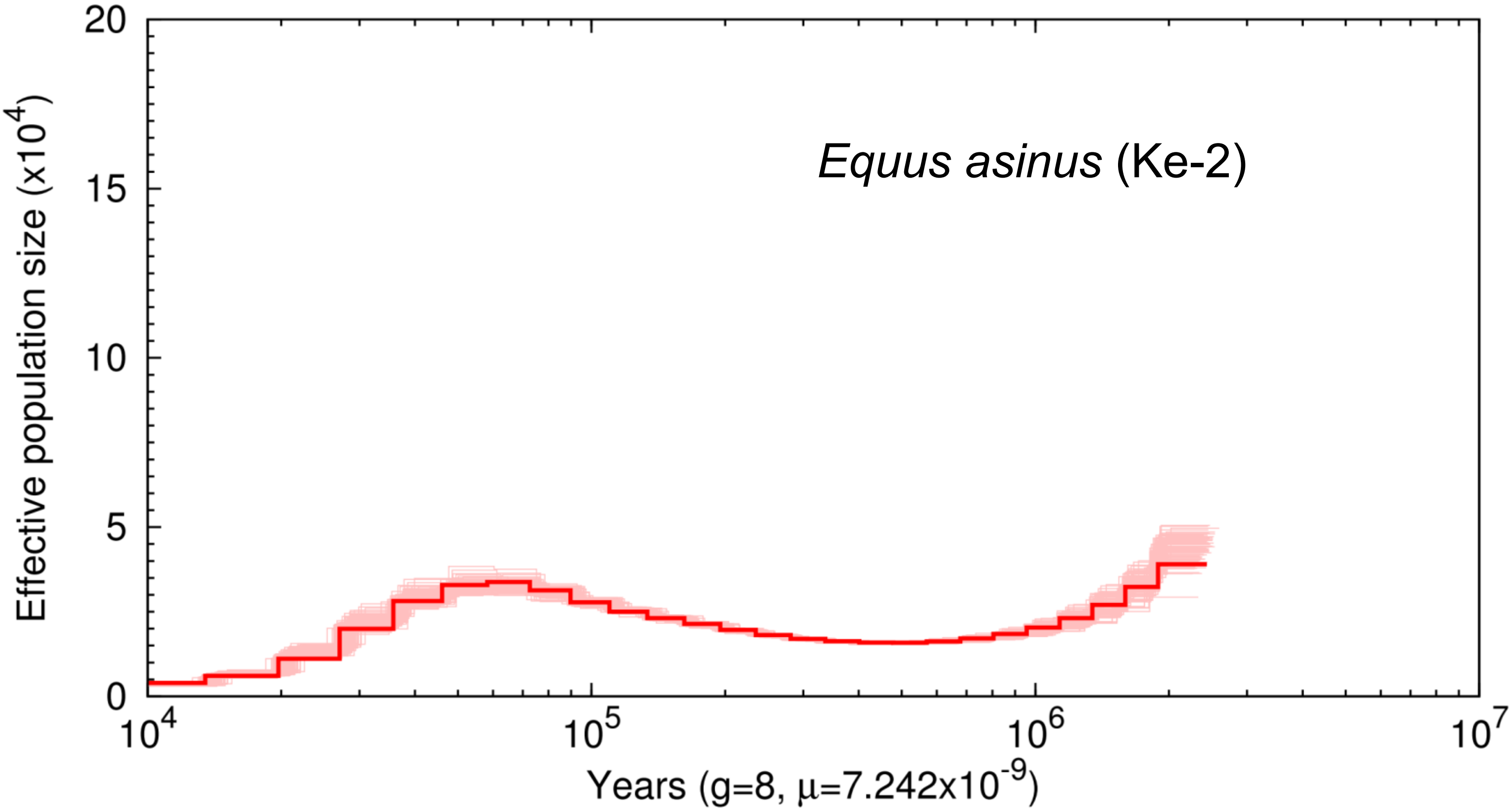
Equus asinus (Ke-2)
Effective population size (x10^4)
20
15
10
5
0
10^4
10^5
10^6
10^7
Years (g=8, μ=7.242x10^-9)

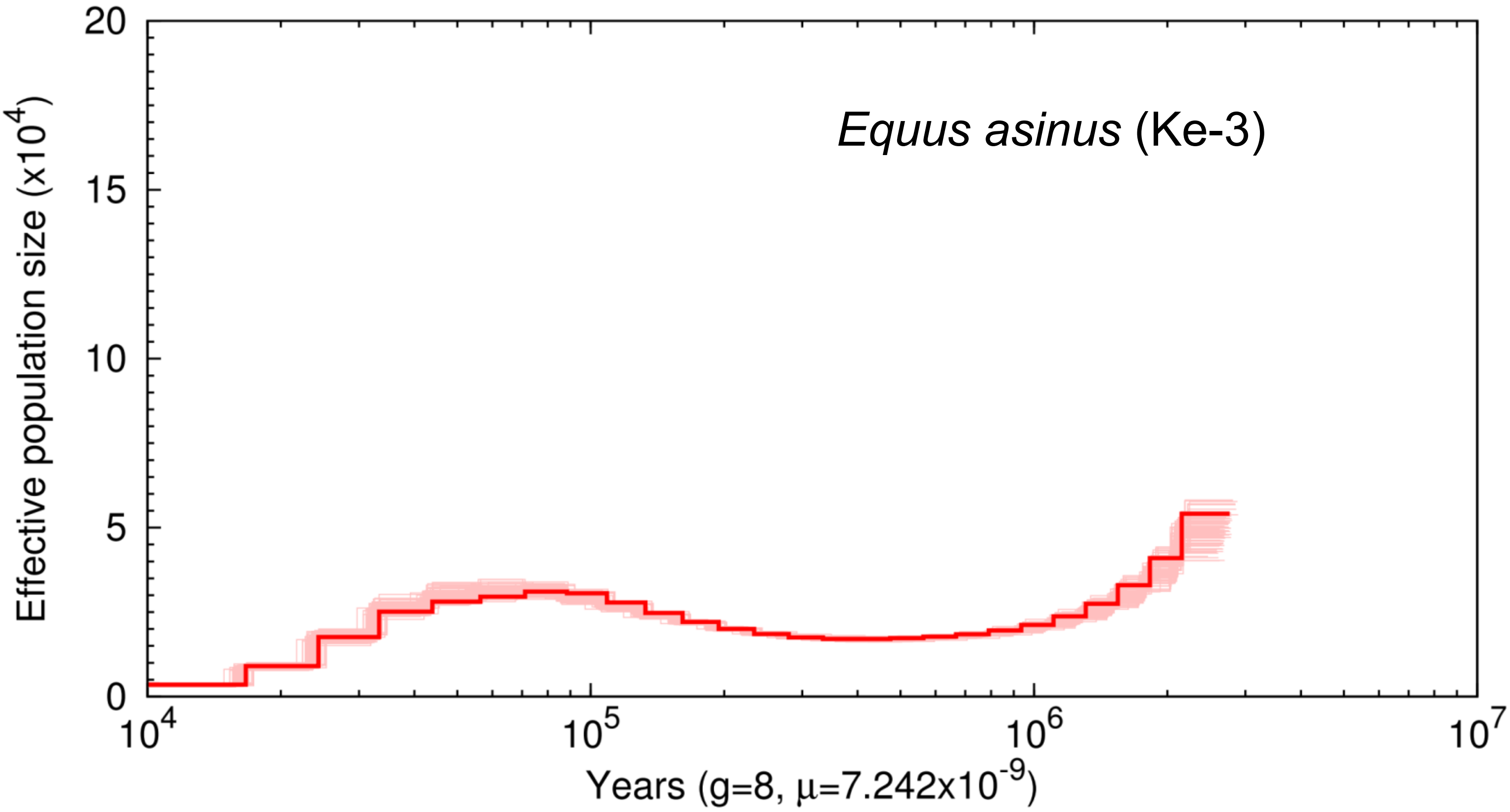
Equus asinus (Ke-3)
Effective population size (x10^4)
20
15
10
5
0
10^4
10^5
10^6
10^7
Years (g=8, μ=7.242x10^-9)

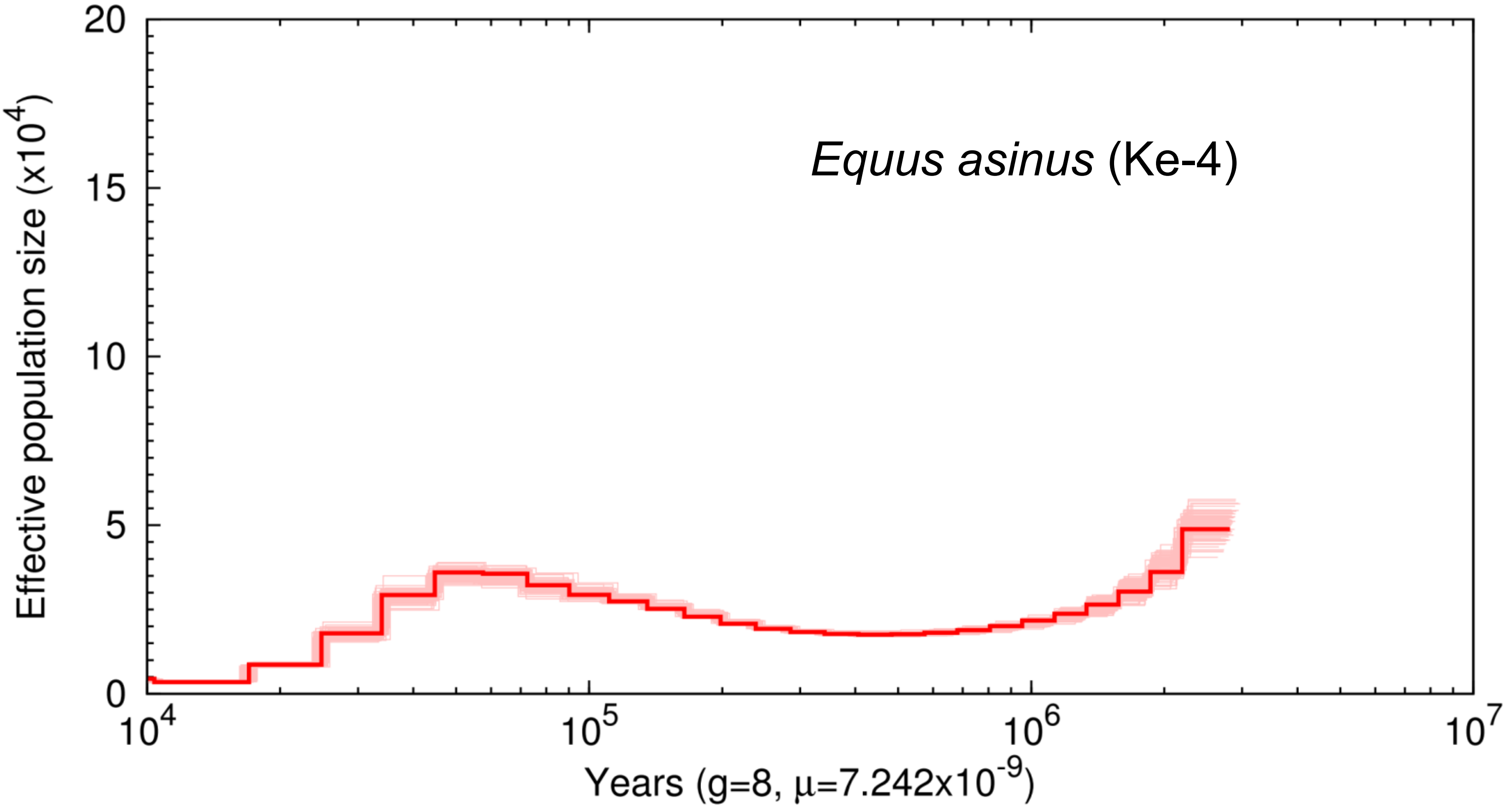

Equus asinus (Ke-4)
Effective population size (x10^4)
20
15
10
5
0
10^4
10^5
10^6
10^7
Years (g=8, μ=7.242x10^-9)


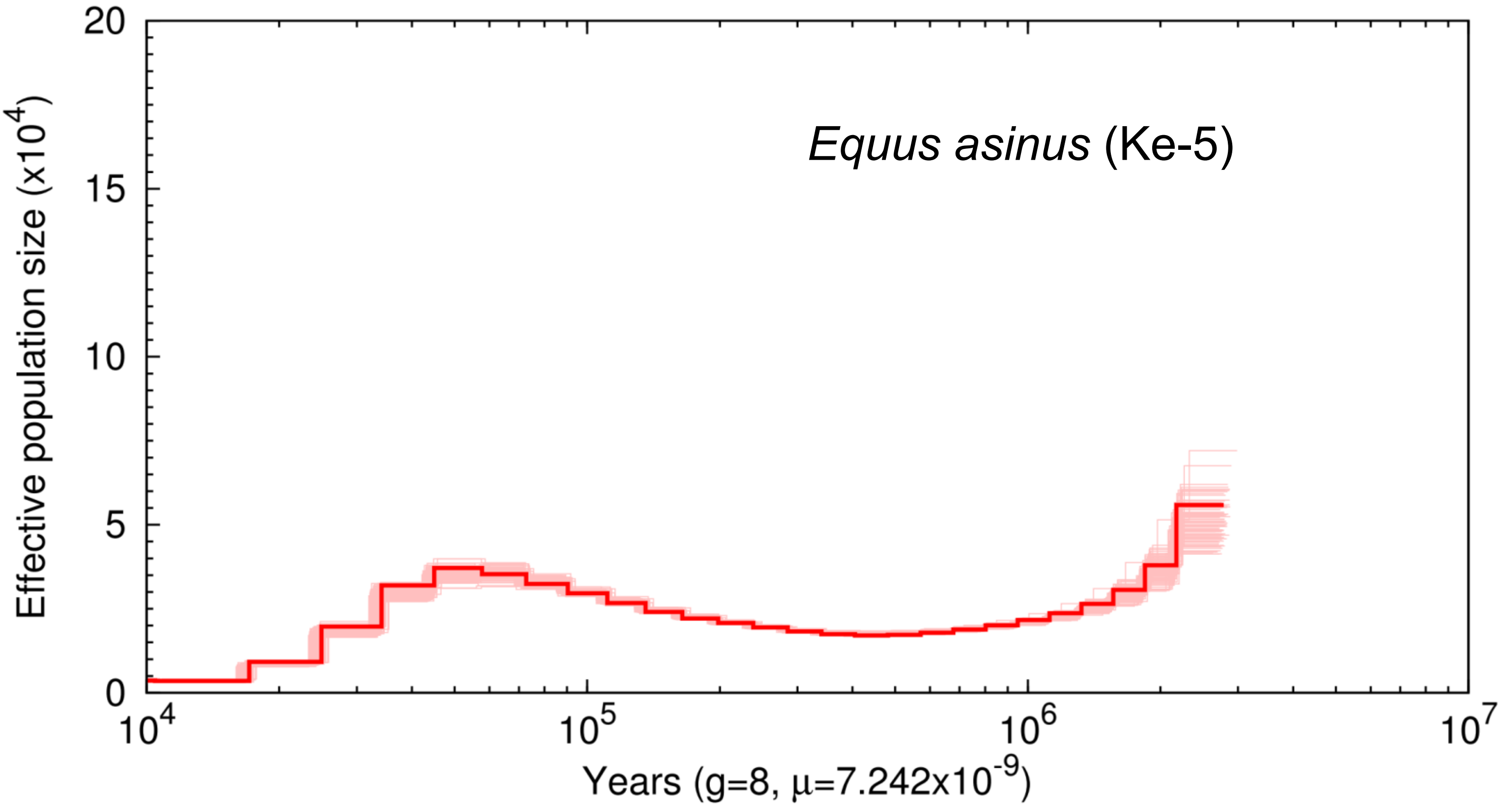

Equus asinus (Ke-5)
Effective population size (x10^4)
20
15
10
5
0
10^4
10^5
10^6
10^7
Years (g=8, μ=7.242x10^-9)

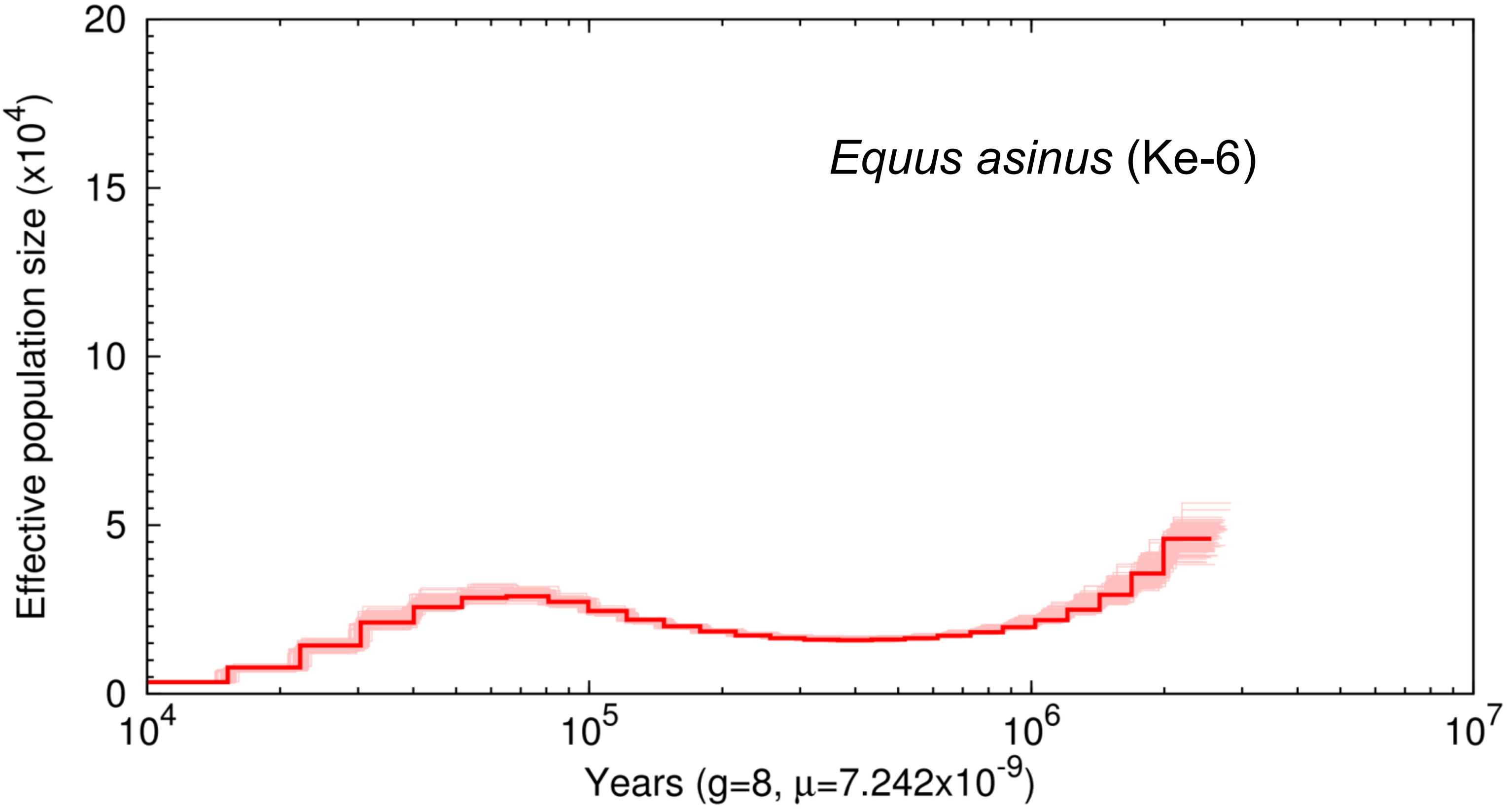

Equus asinus (Ke-6)
Effective population size (x10^4)
20
15
10
5
0
10^4
10^5
10^6
10^7
Years (g=8, μ=7.242x10^-9)


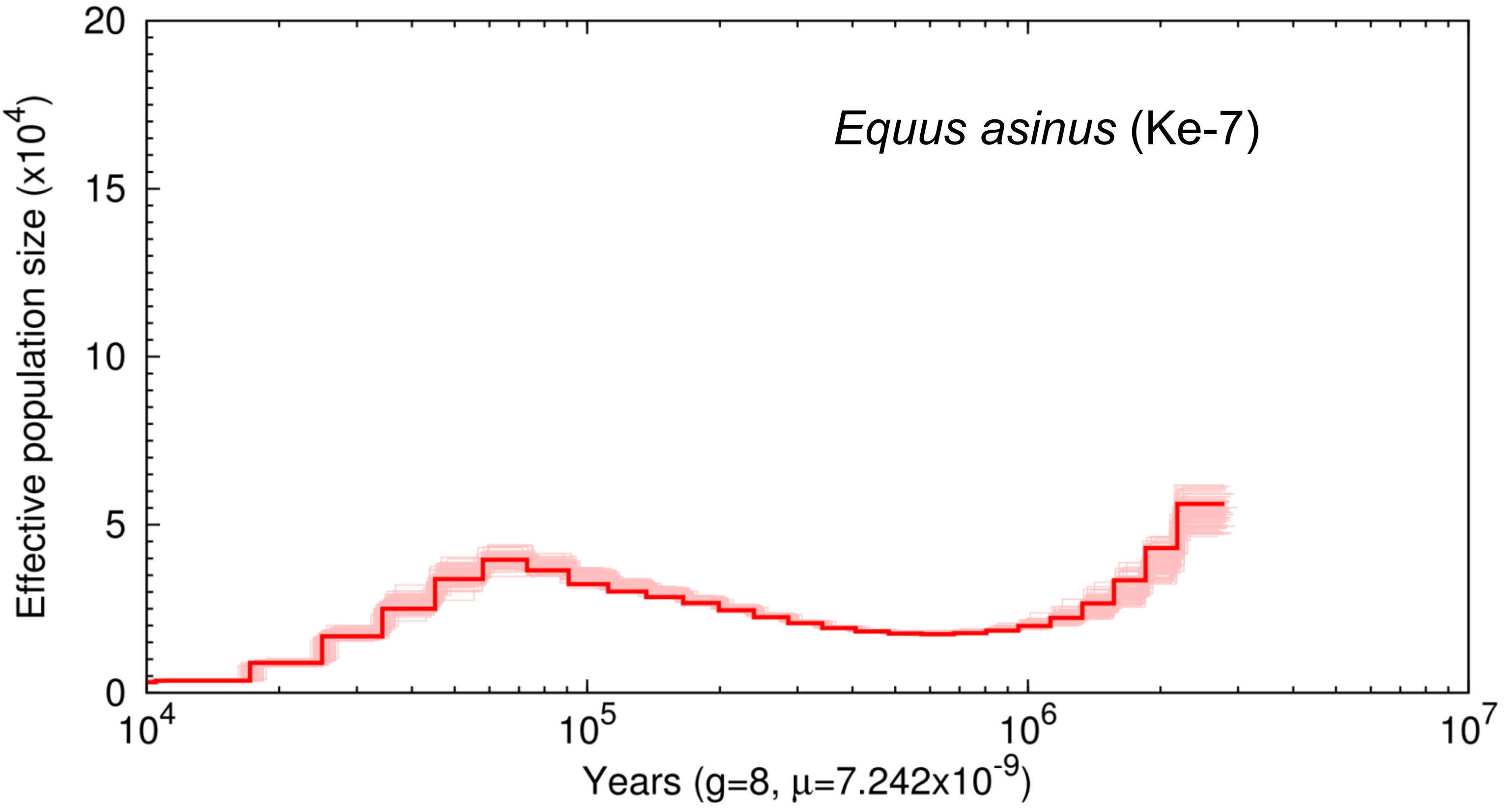

Equus asinus (Ke-7)
Effective population size (x10^4)
20
15
10
5
0
10^4
10^5
10^6
10^7
Years (g=8, μ=7.242x10^-9)

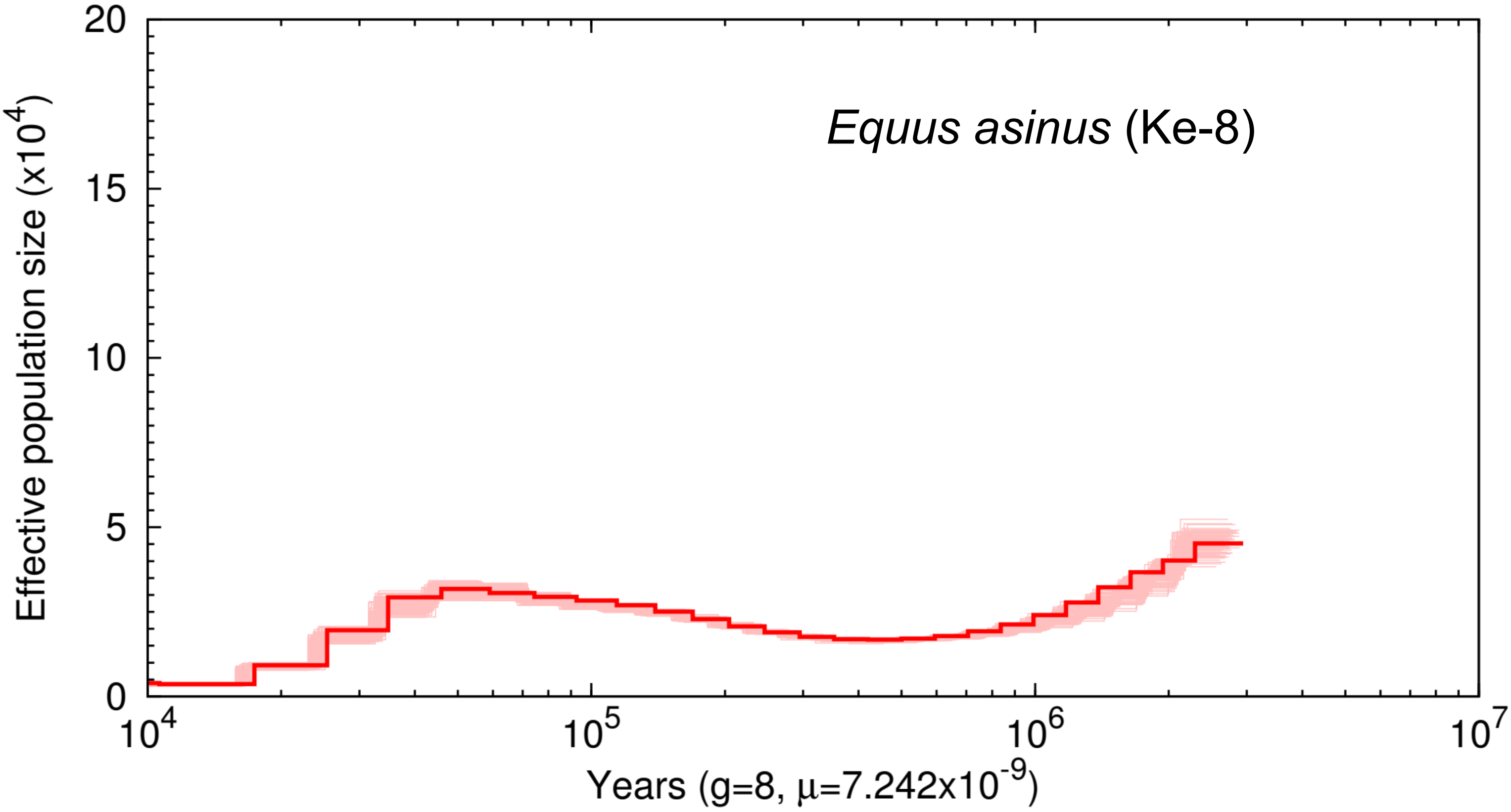
Equus asinus (Ke-8)
Effective population size (x10^4)
20
15
10
5
0
10^4
10^5
10^6
10^7
Years (g=8, μ=7.242x10^-9)

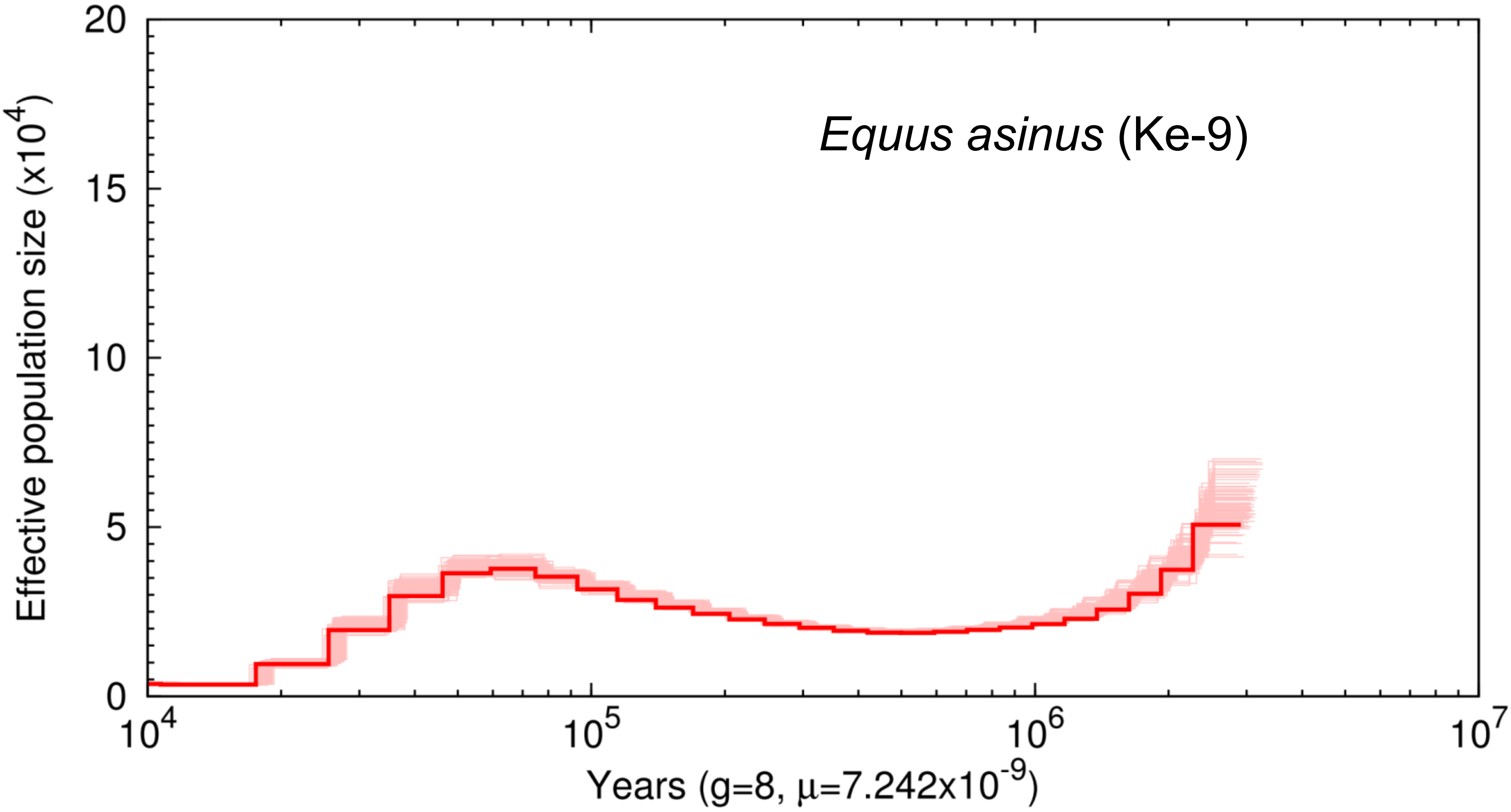
Equus asinus (Ke-9)
Effective population size (x10^4)
20
15
10
5
0
10^4
10^5
10^6
10^7
Years (g=8, μ=7.242x10^-9)

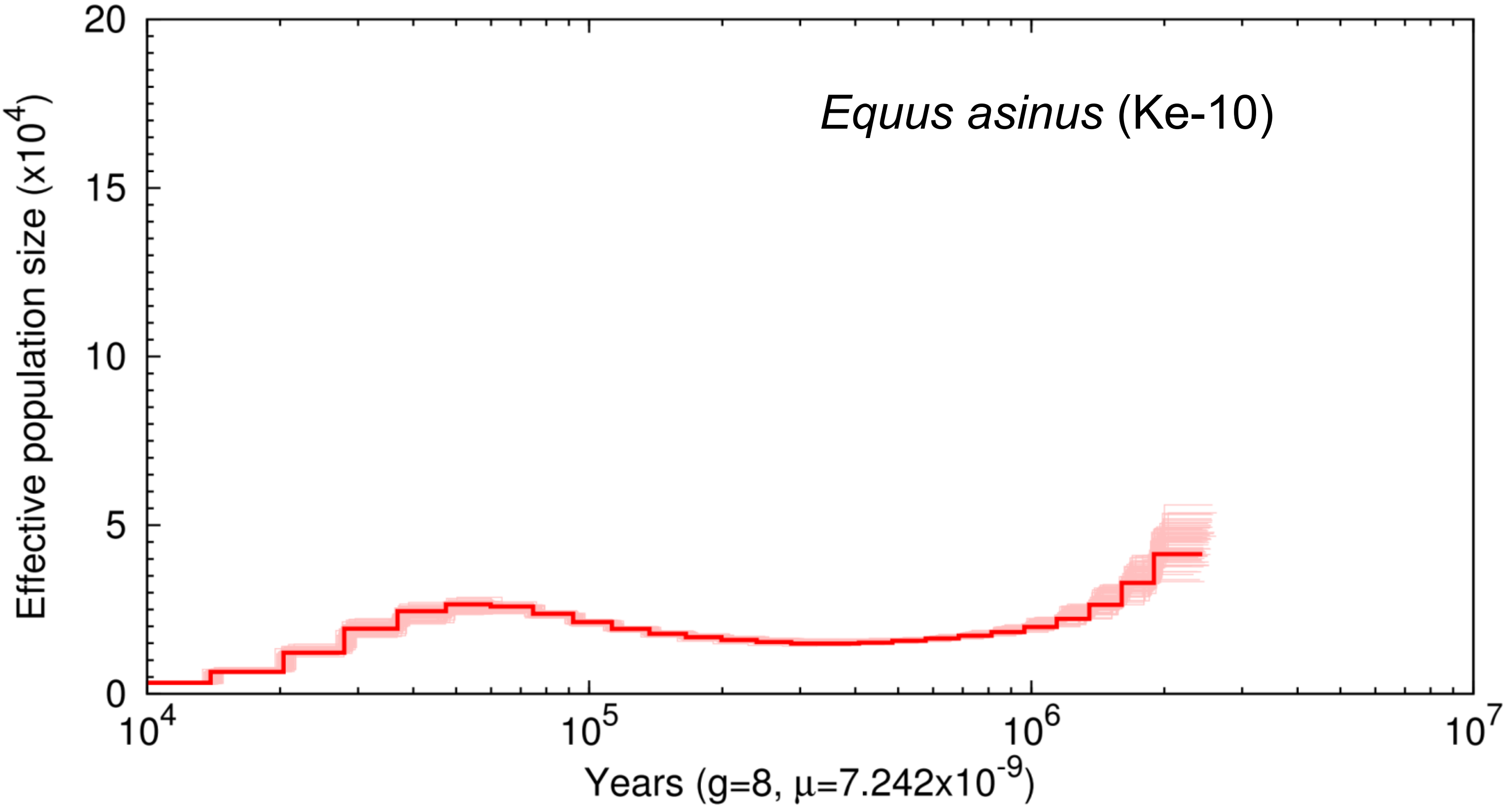

Equus asinus (Ke-10)
Effective population size (x10^4)
0
5
10
15
20
10^4
10^5
10^6
10^7
Years (g=8, μ=7.242x10^-9)


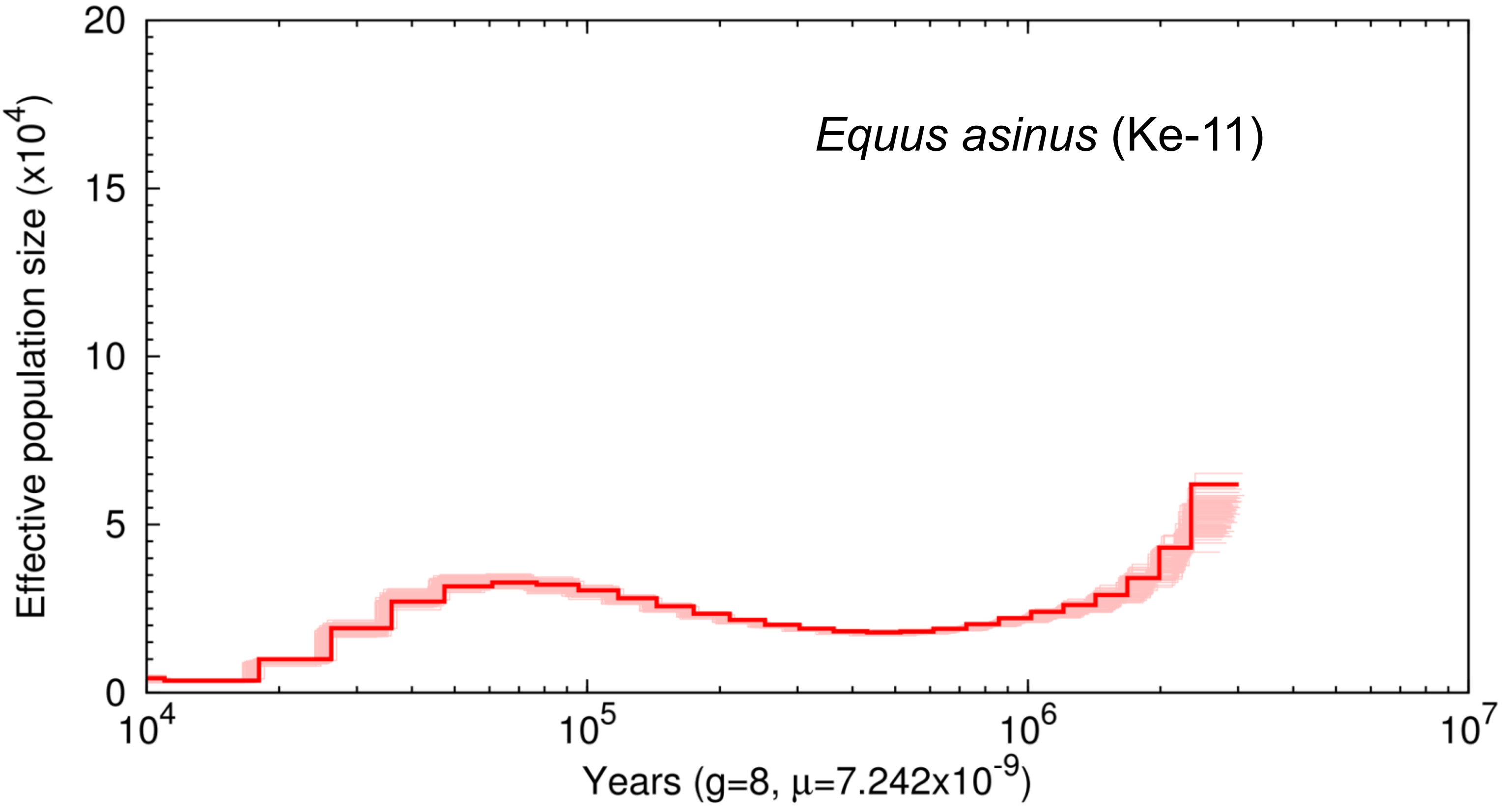

Equus asinus (Ke-11)
Effective population size (x10^4)
0
5
10
15
20
10^4
10^5
10^6
10^7
Years (g=8, μ=7.242x10^-9)

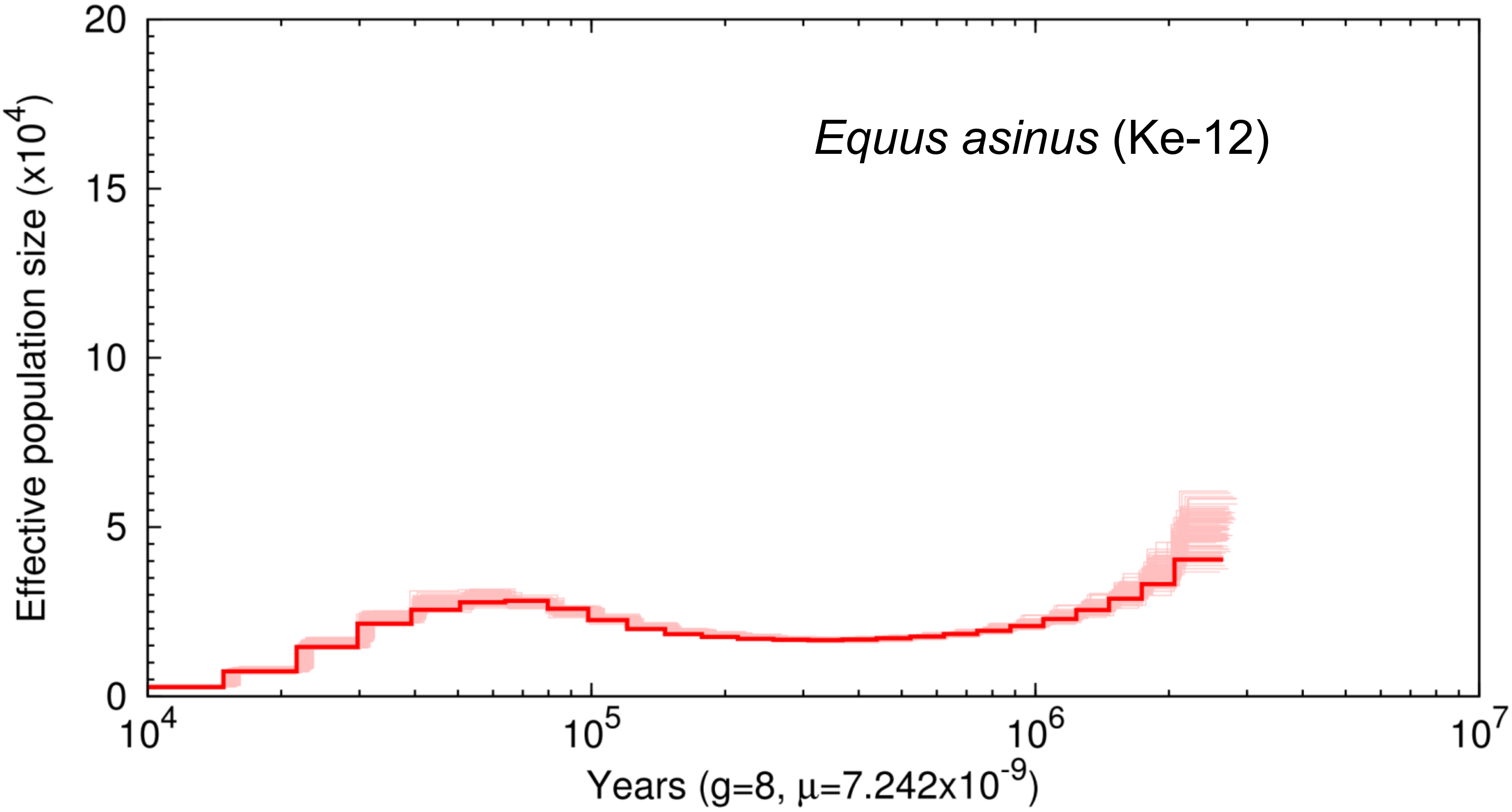
Equus asinus (Ke-12)
Effective population size (x10^4)
0
5
10
15
20
10^4
10^5
10^6
10^7
Years (g=8, μ=7.242x10^-9)

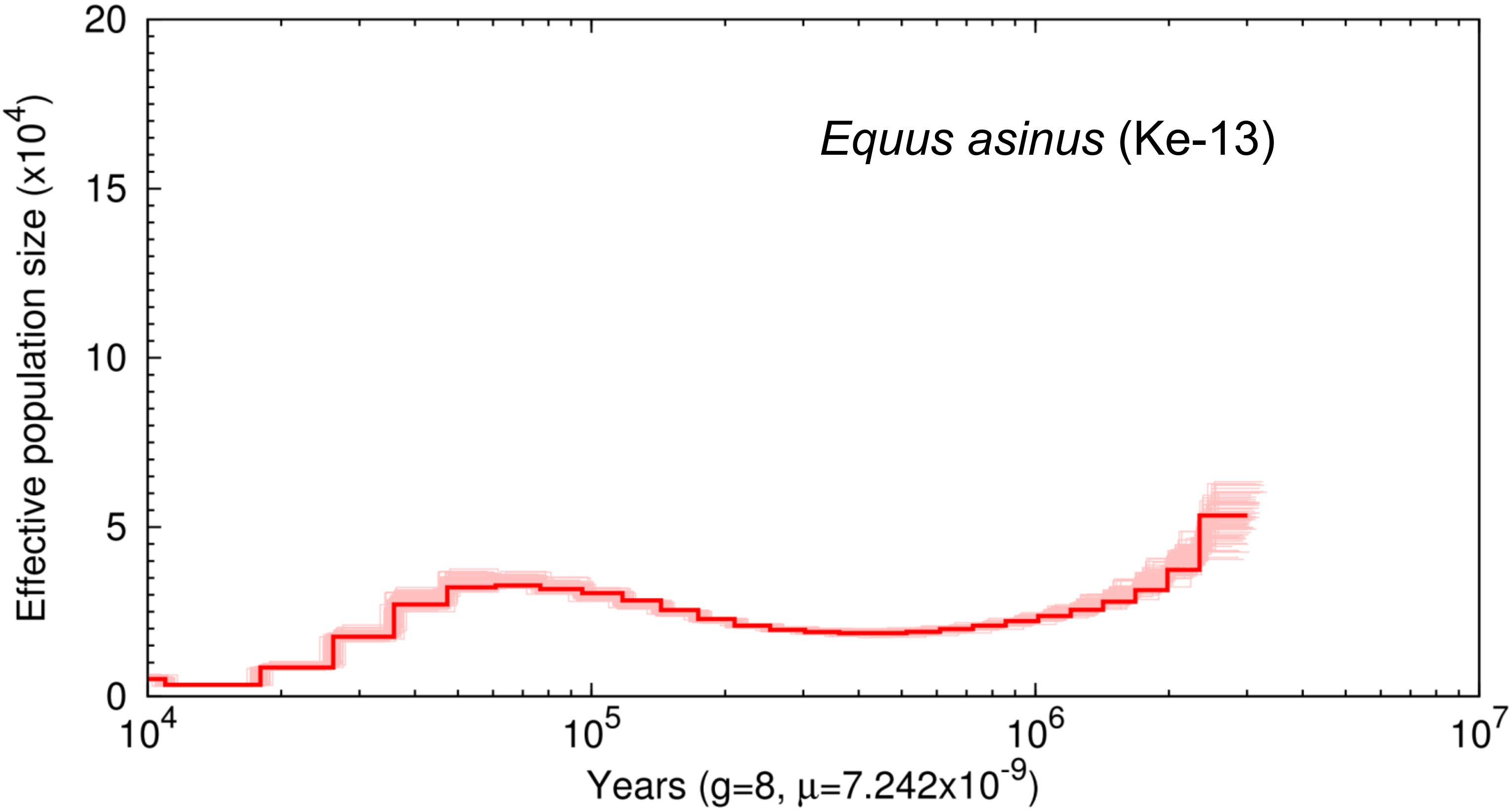
Equus asinus (Ke-13)
Effective population size (x10^4)
0
5
10
15
20
10^4
10^5
10^6
10^7
Years (g=8, μ=7.242x10^-9)

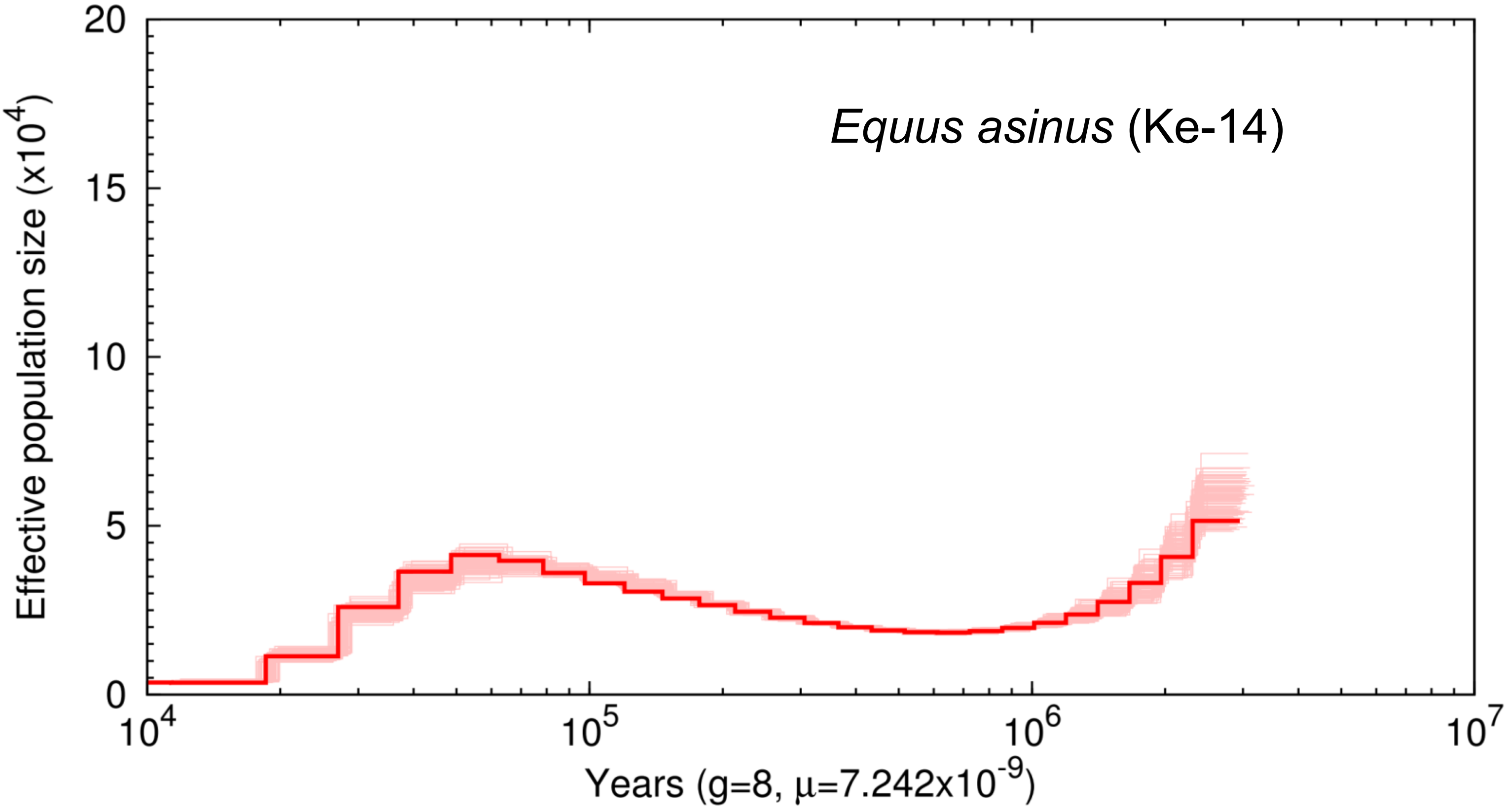
Equus asinus (Ke-14)
Effective population size (x10^4)
20
15
10
5
0
10^4
10^5
10^6
10^7
Years (g=8, μ=7.242x10^-9)

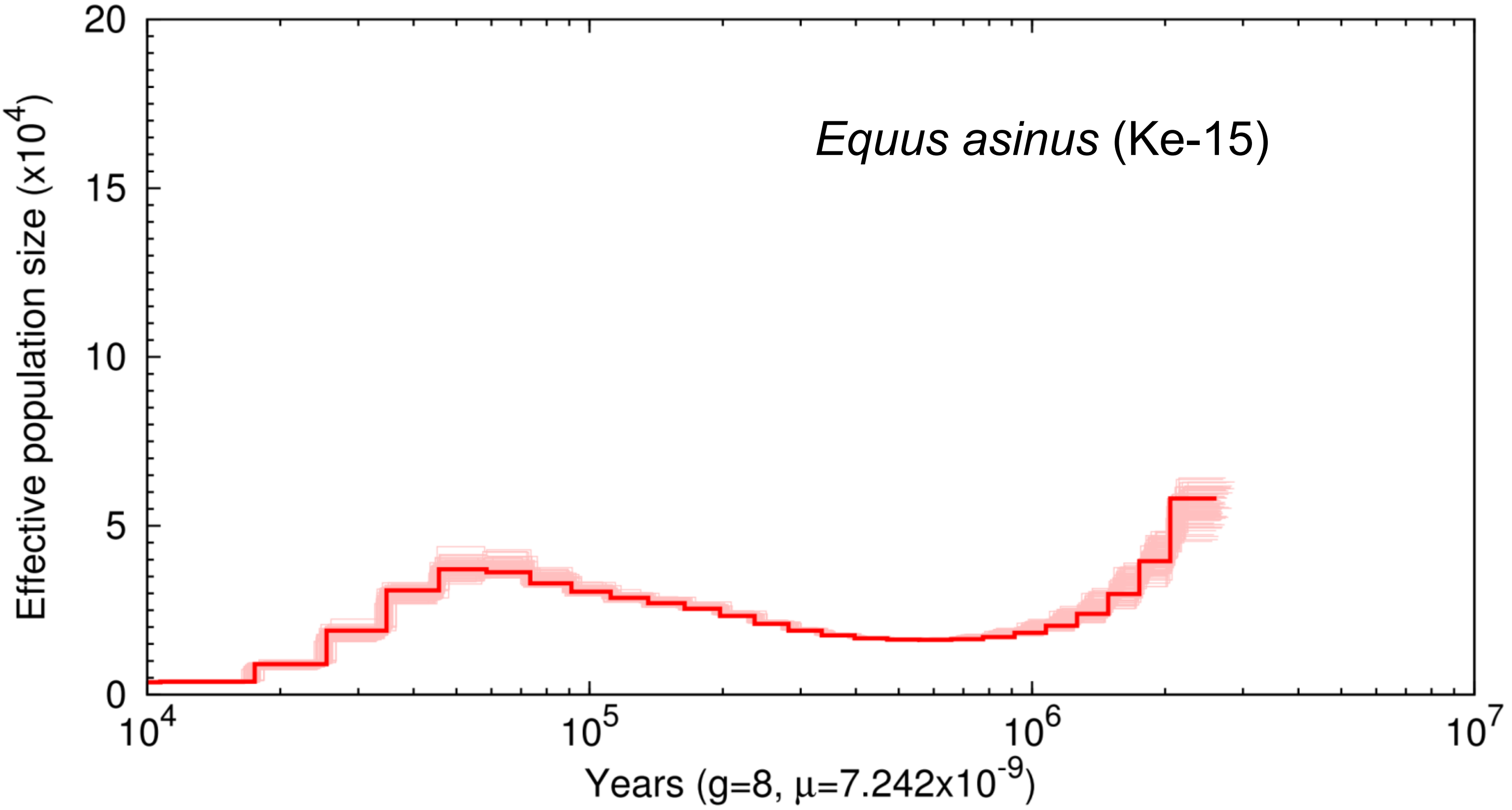
Equus asinus (Ke-15)
Effective population size (x10^4)
20
15
10
5
0
10^4
10^5
10^6
10^7
Years (g=8, μ=7.242x10^-9)

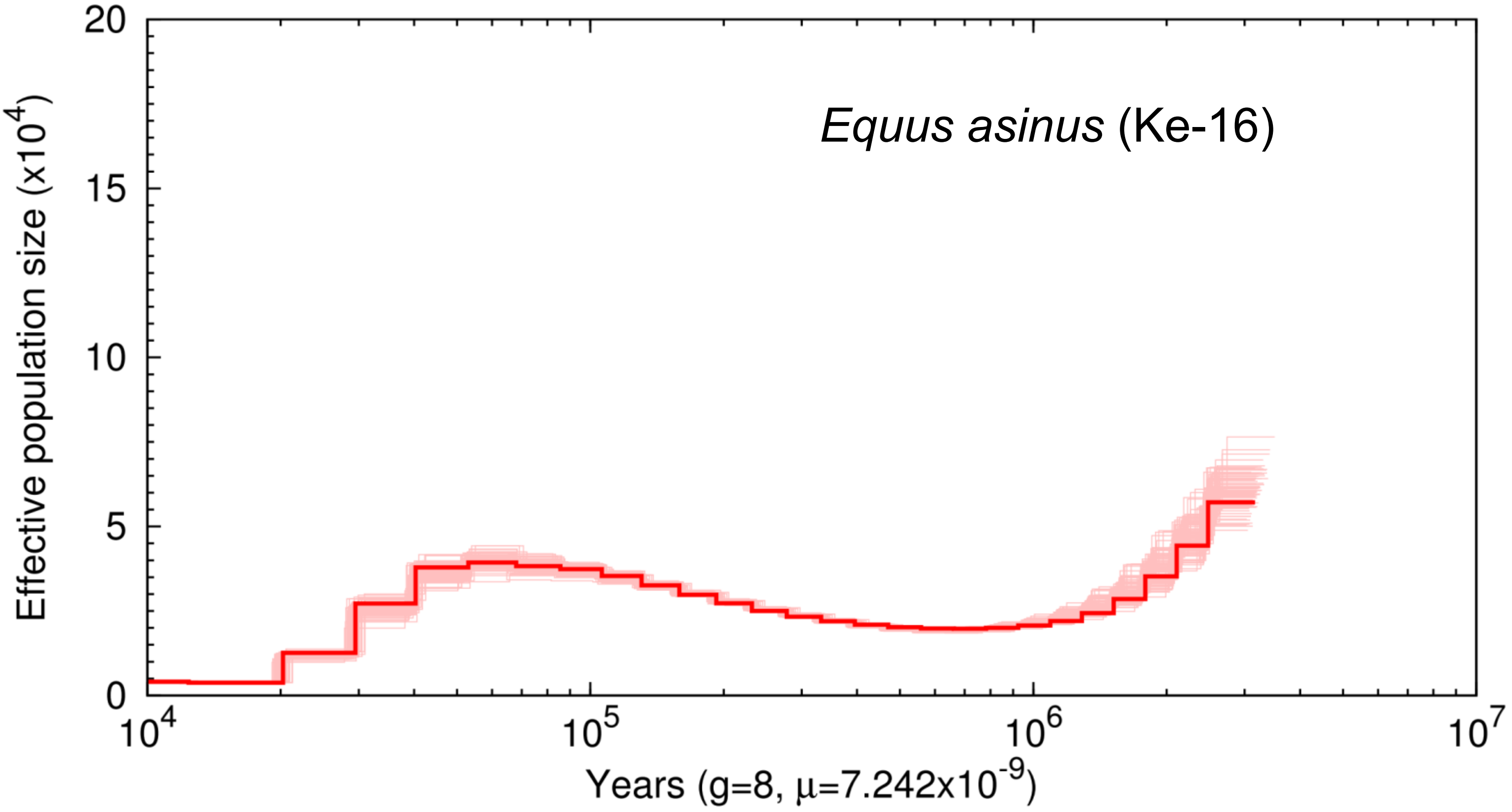
Equus asinus (Ke-16)
Effective population size (x10^4)
0
5
10
15
20
10^4
10^5
10^6
10^7
Years (g=8, μ=7.242x10^-9)

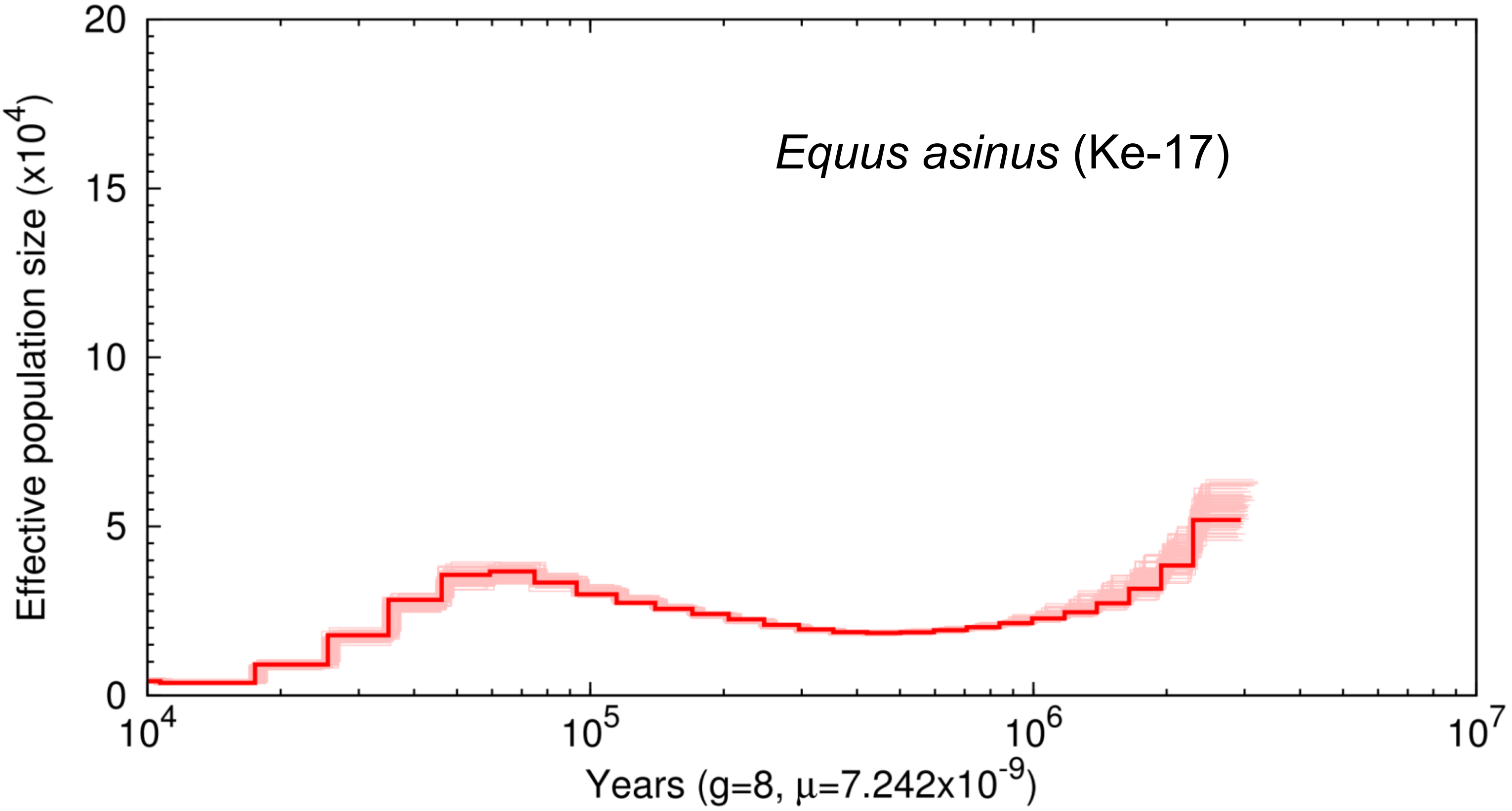
Equus asinus (Ke-17)
Effective population size (x10^4)
0
5
10
15
20
10^4
10^5
10^6
10^7
Years (g=8, μ=7.242x10^-9)

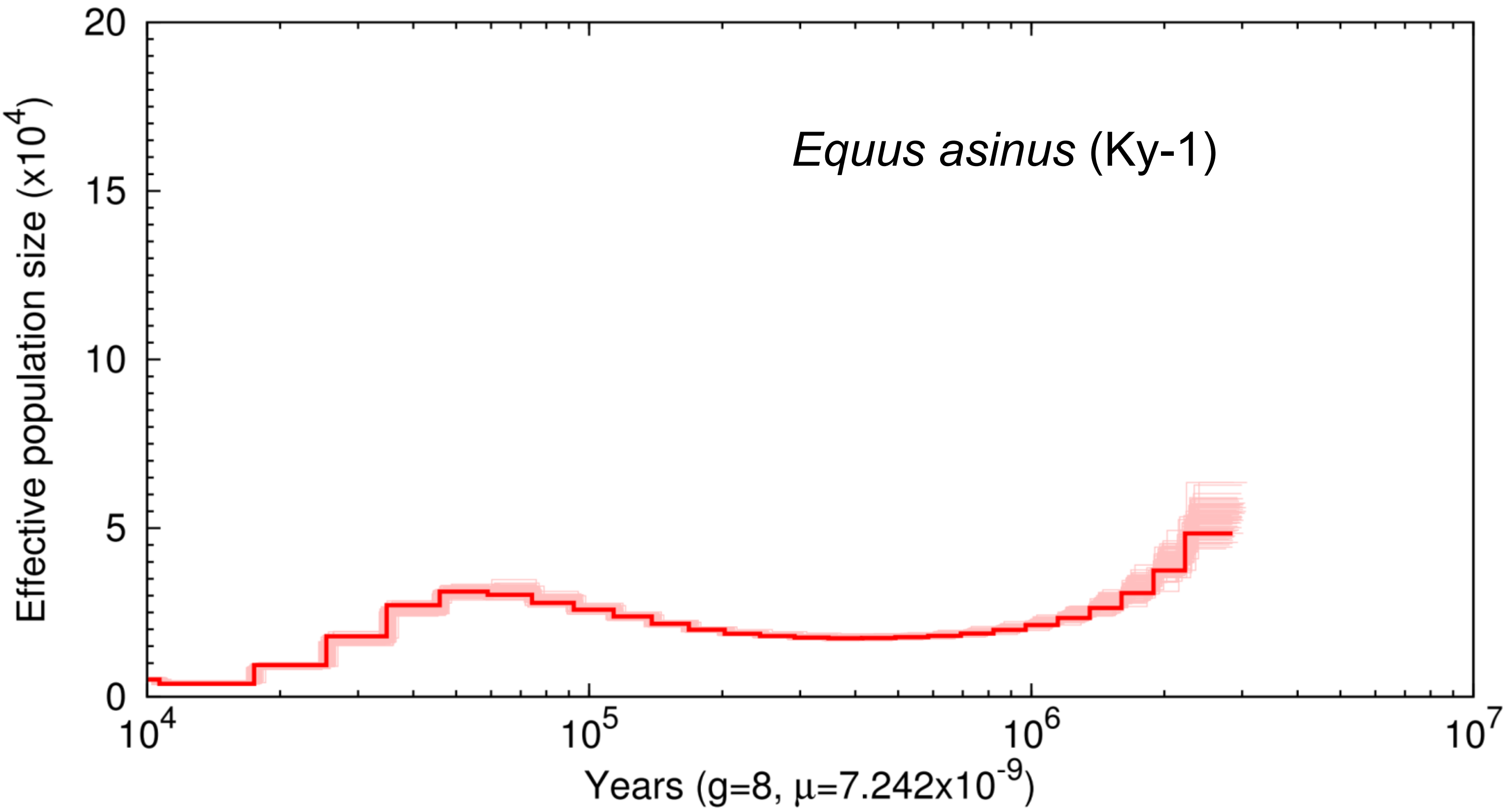
Equus asinus (Ky-1)
Effective population size (x10^4)
Years (g=8, μ=7.242x10^-9)
0
5
10
15
20
10^4
10^5
10^6
10^7

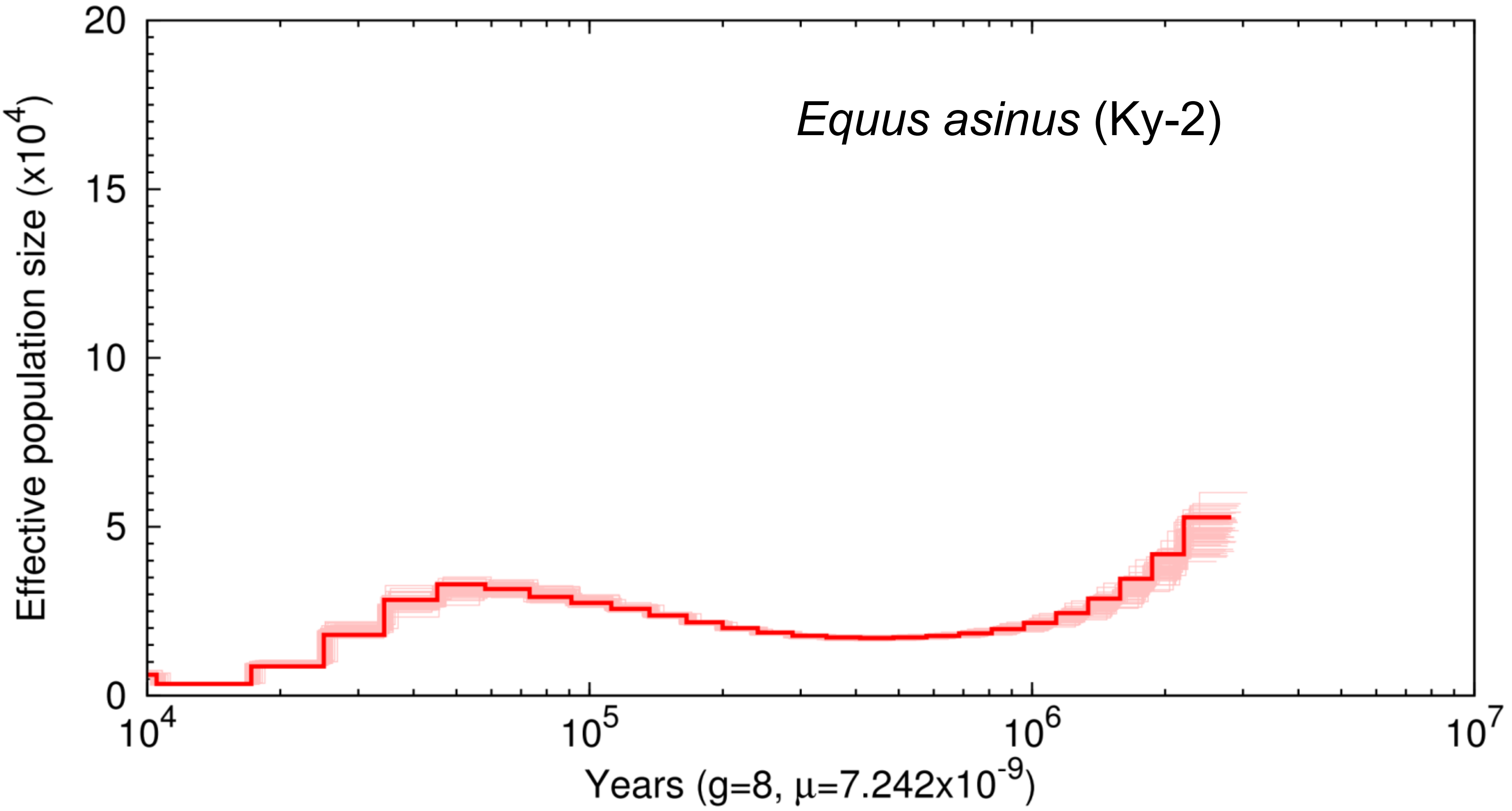
Equus asinus (Ky-2)
Effective population size (x10^4)
Years (g=8, μ=7.242x10^-9)
0
5
10
15
20
10^4
10^5
10^6
10^7

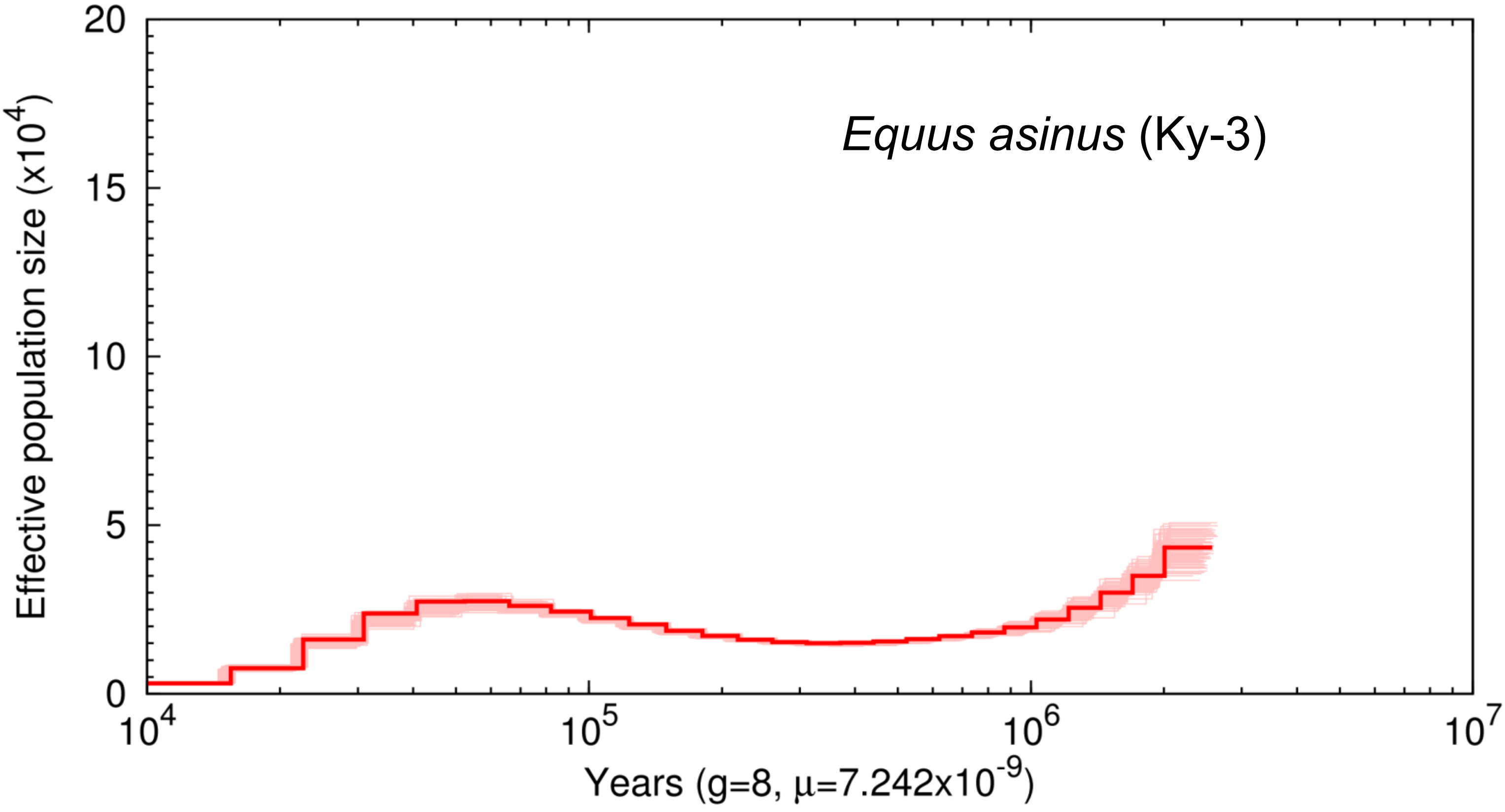

Equus asinus (Ky-3)
Effective population size (x10^4)
20
15
10
5
0
10^4
10^5
10^6
10^7
Years (g=8, μ=7.242x10^-9)

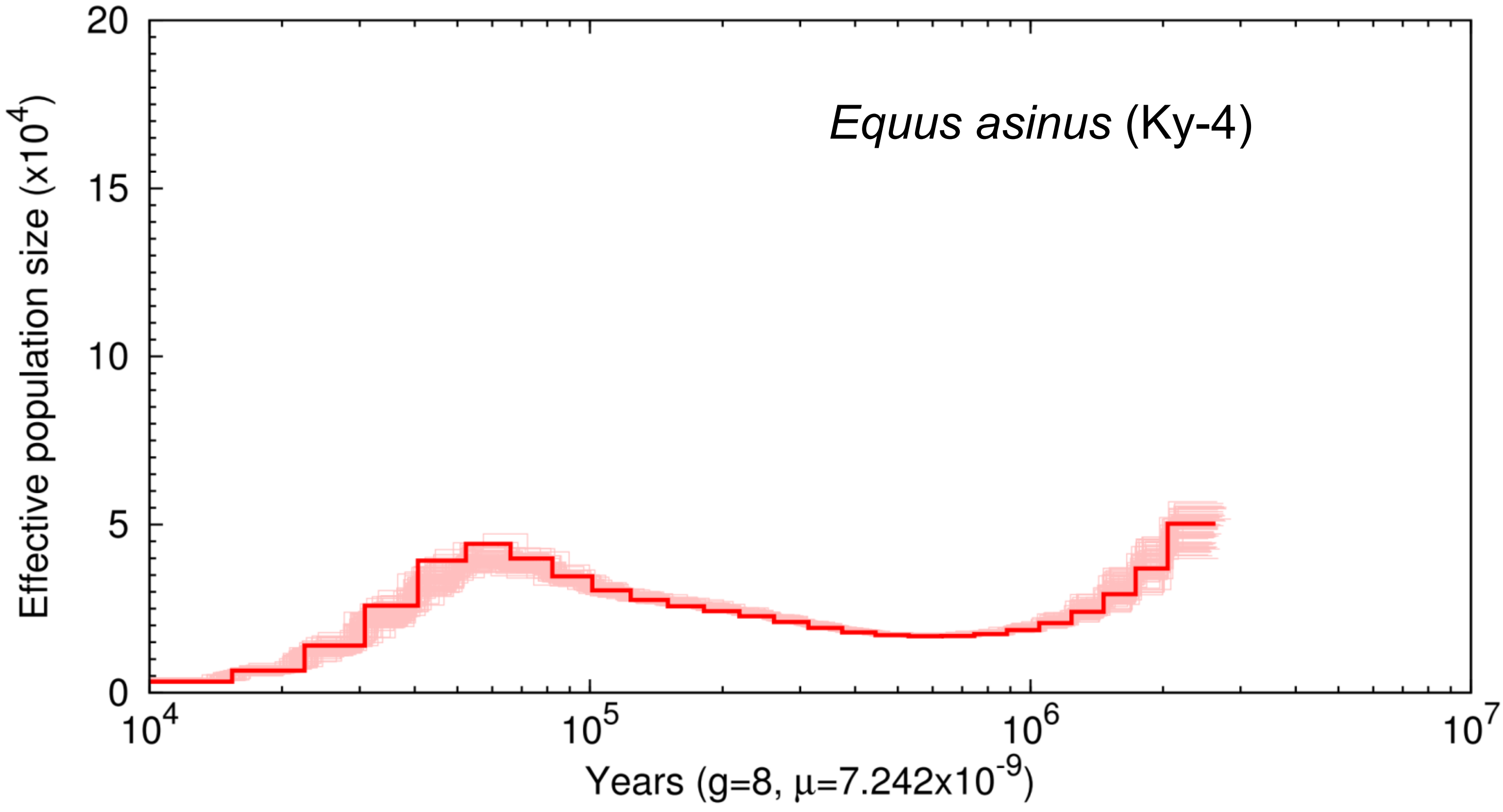

Equus asinus (Ky-4)
Effective population size (x10^4)
20
15
10
5
0
10^4
10^5
10^6
10^7
Years (g=8, μ=7.242x10^-9)

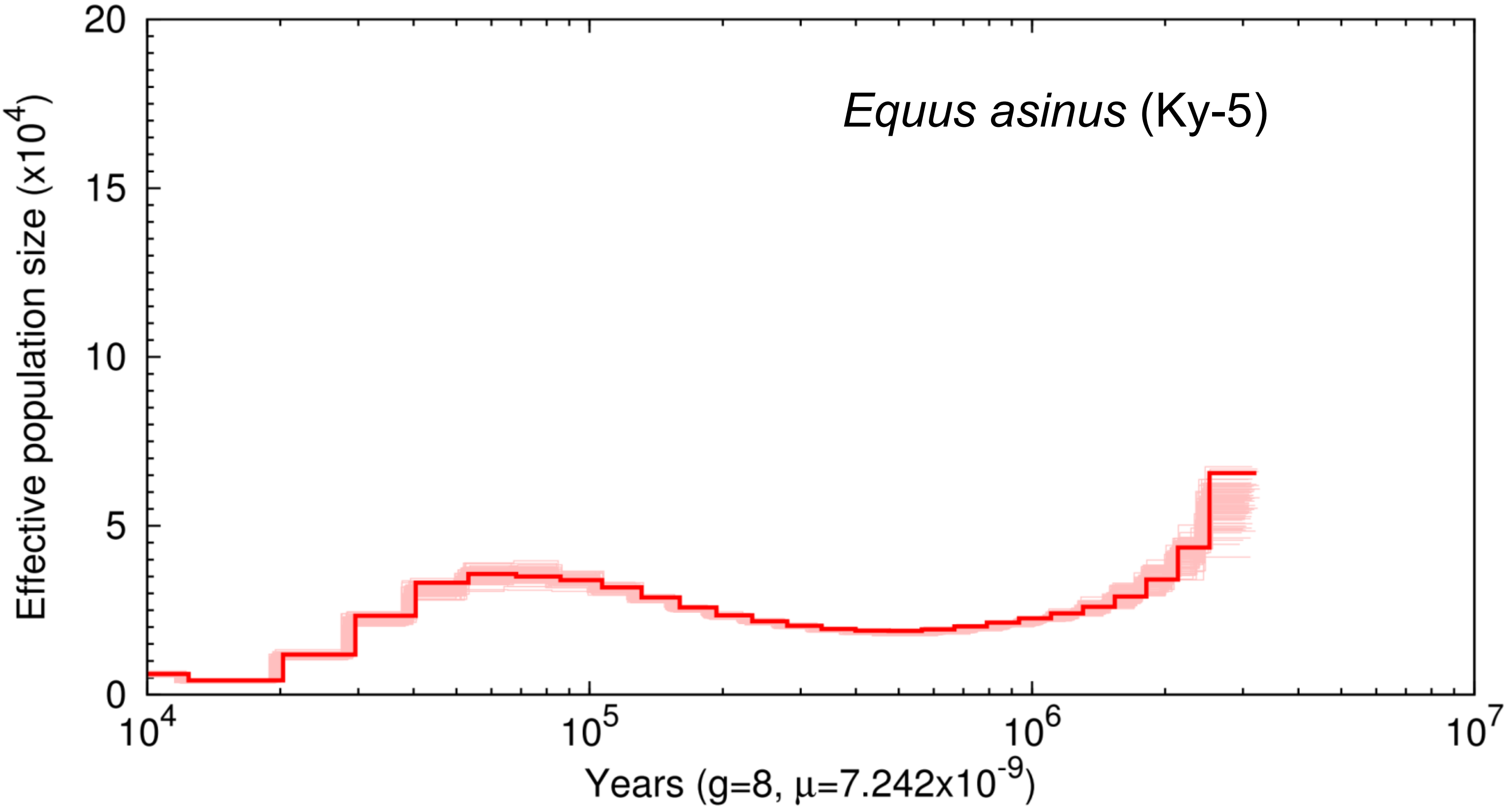
Equus asinus (Ky-5)
Effective population size (x10^4)
0
5
10
15
20
10^4
10^5
10^6
10^7
Years (g=8, μ=7.242x10^-9)

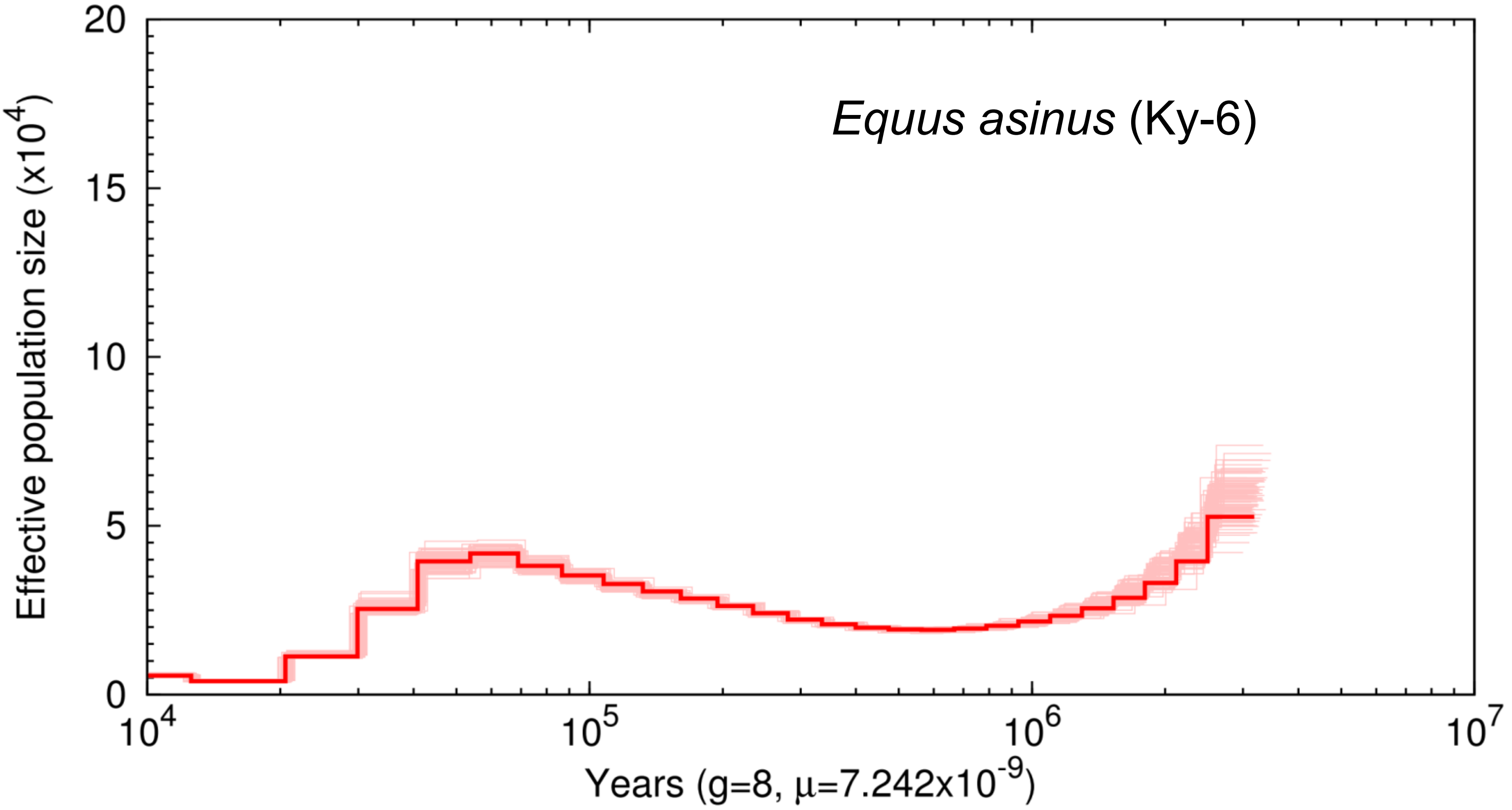
Equus asinus (Ky-6)
Effective population size (x10^4)
0
5
10
15
20
10^4
10^5
10^6
10^7
Years (g=8, μ=7.242x10^-9)

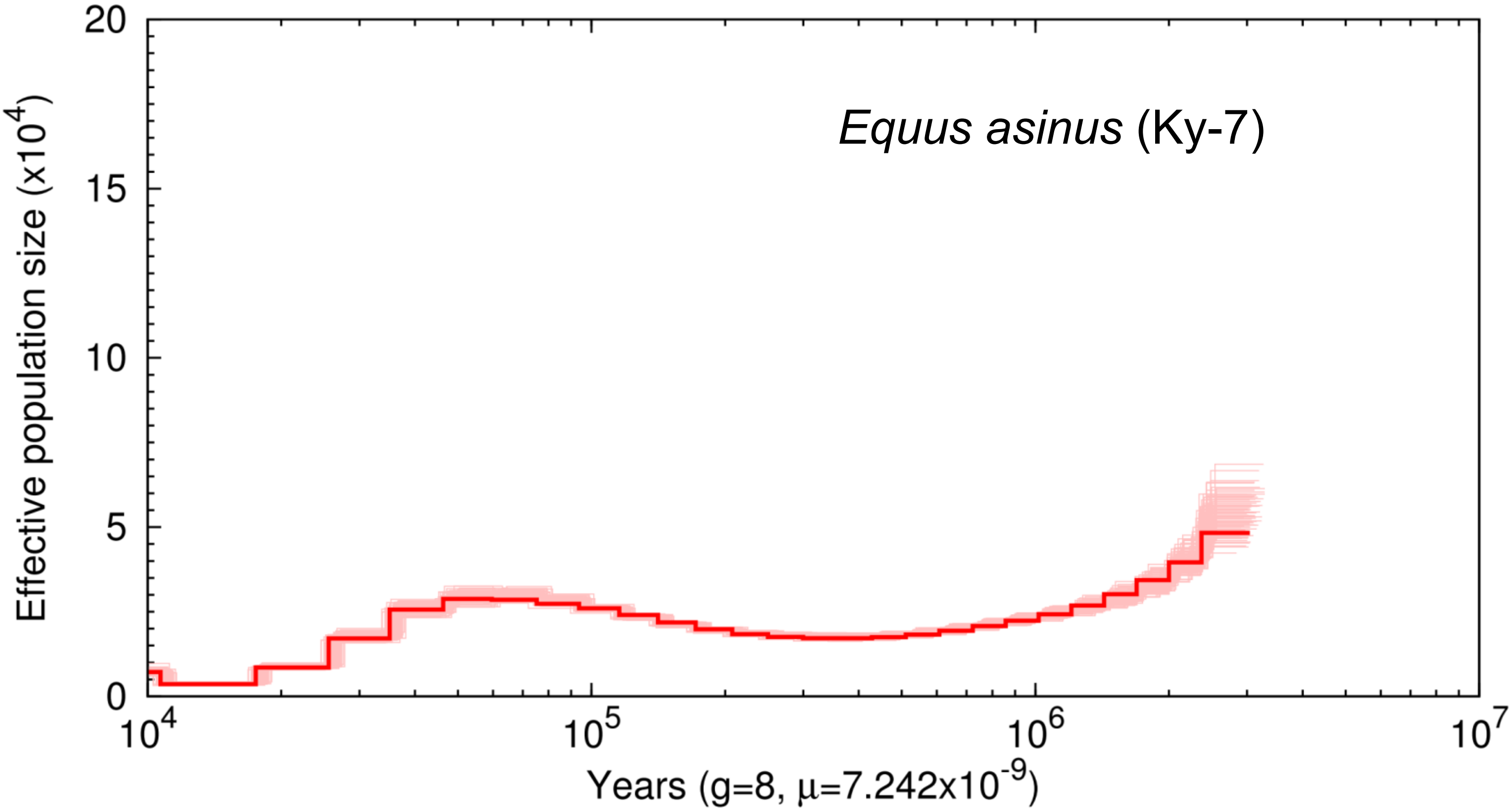
Equus asinus (Ky-7)
Effective population size (x10^4)
20
15
10
5
0
10^4
10^5
10^6
10^7
Years (g=8, μ=7.242x10^-9)

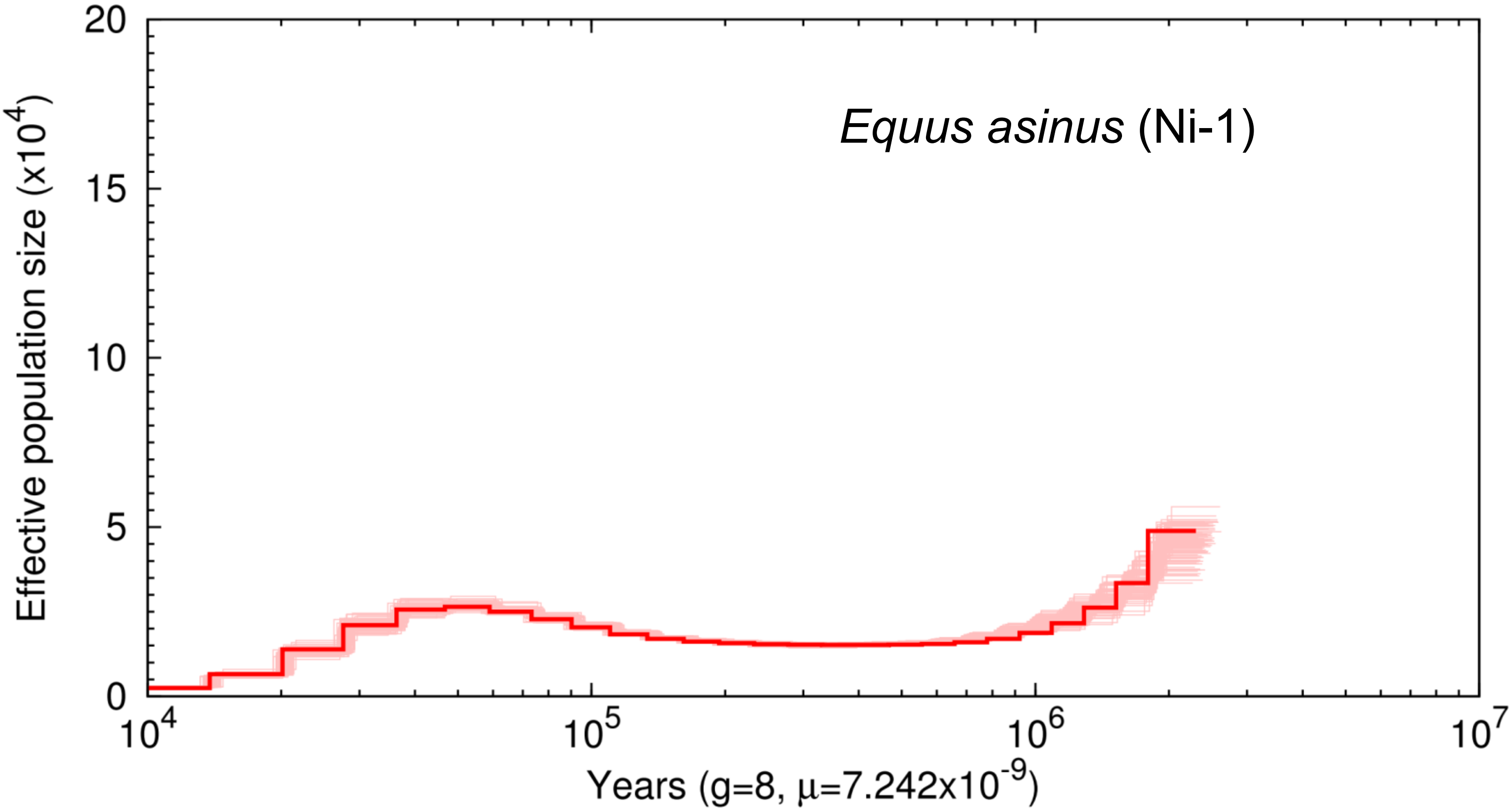
Equus asinus (Ni-1)
Effective population size (x10^4)
20
15
10
5
0
10^4
10^5
10^6
10^7
Years (g=8, μ=7.242x10^-9)

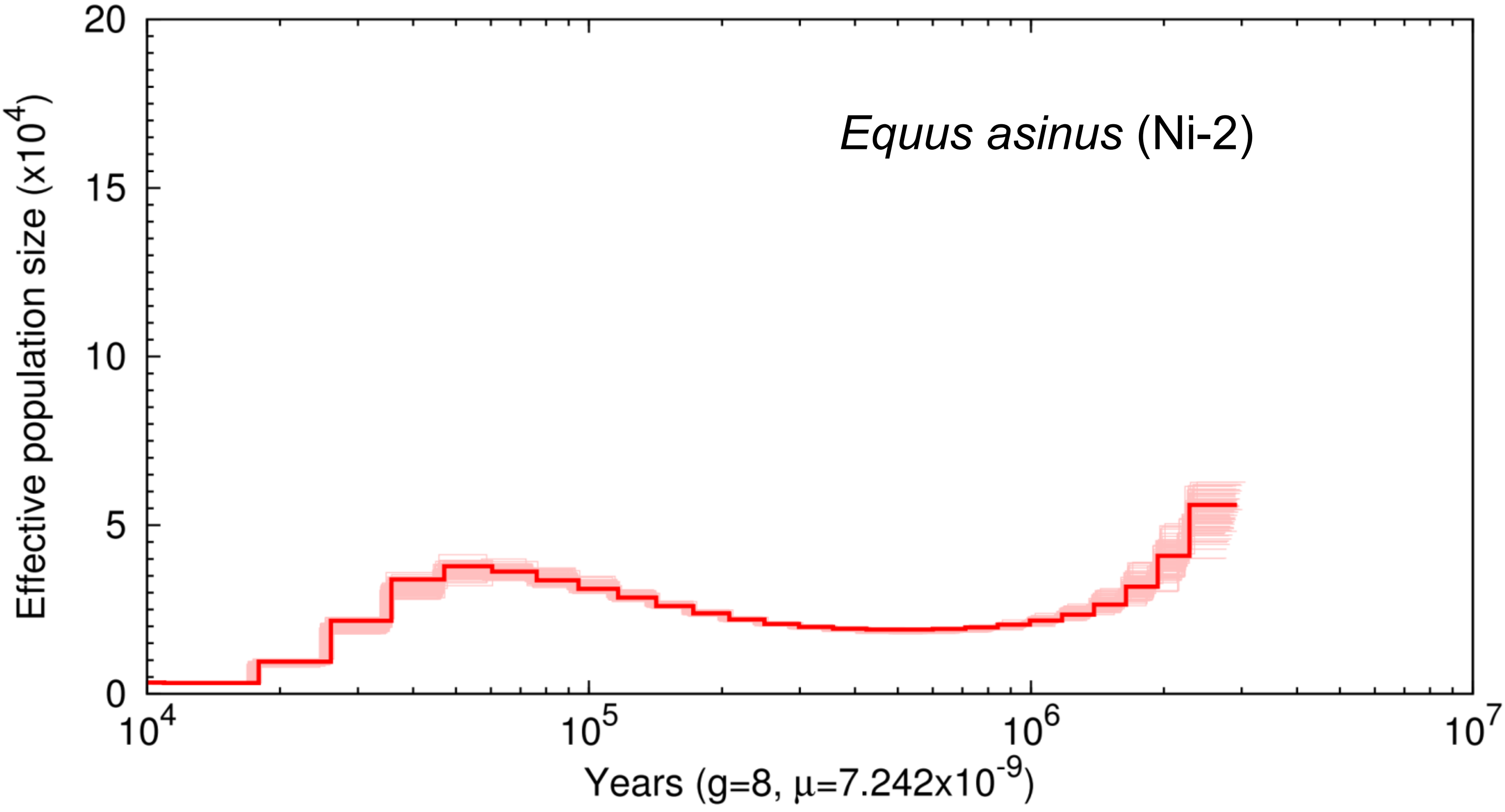

Equus asinus (Ni-2)
Effective population size (x10^4)
20
15
10
5
0
10^4
10^5
10^6
10^7
Years (g=8, μ=7.242x10^-9)


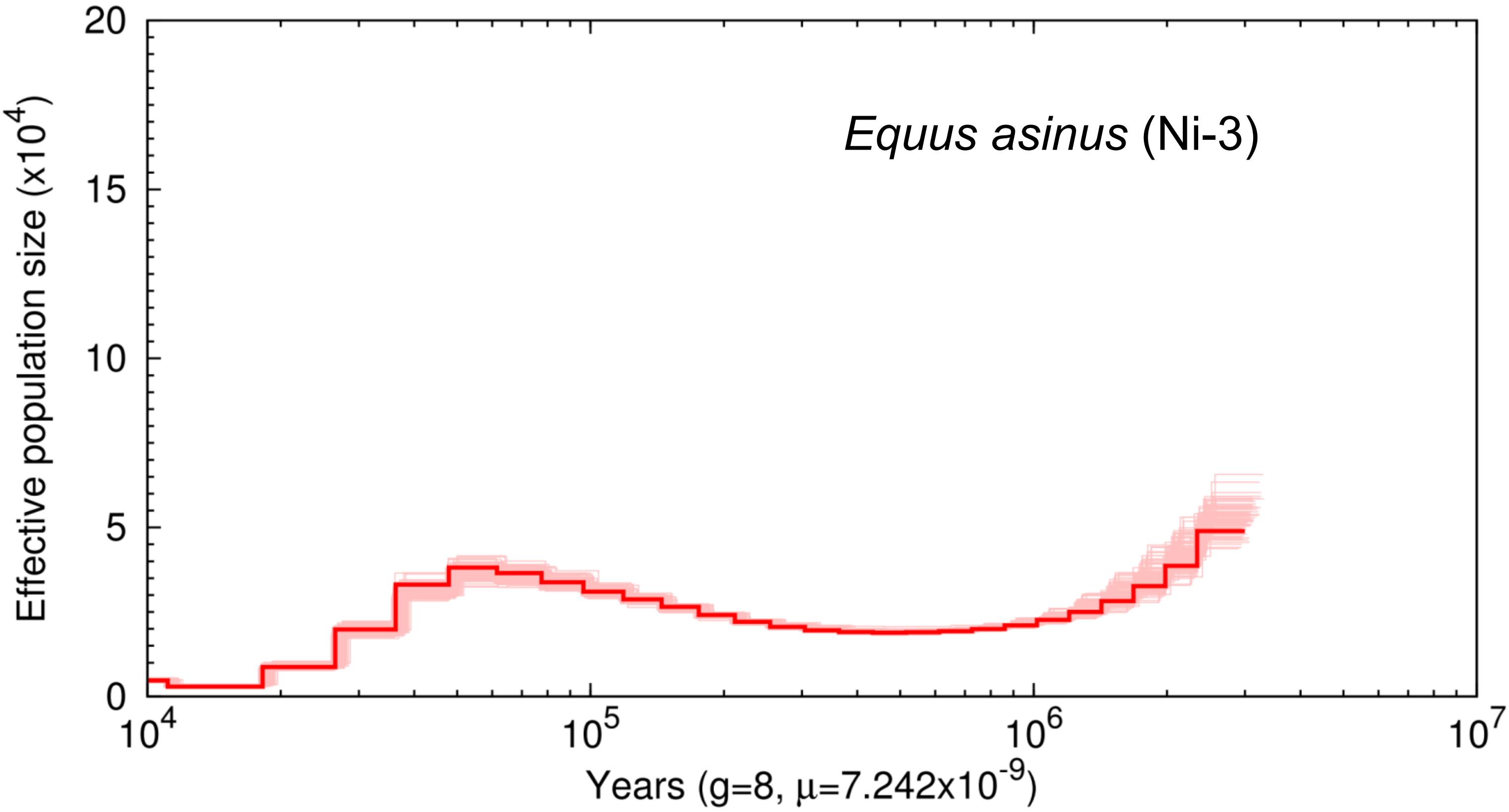

Equus asinus (Ni-3)
Effective population size (x10^4)
20
15
10
5
0
10^4
10^5
10^6
10^7
Years (g=8, μ=7.242x10^-9)

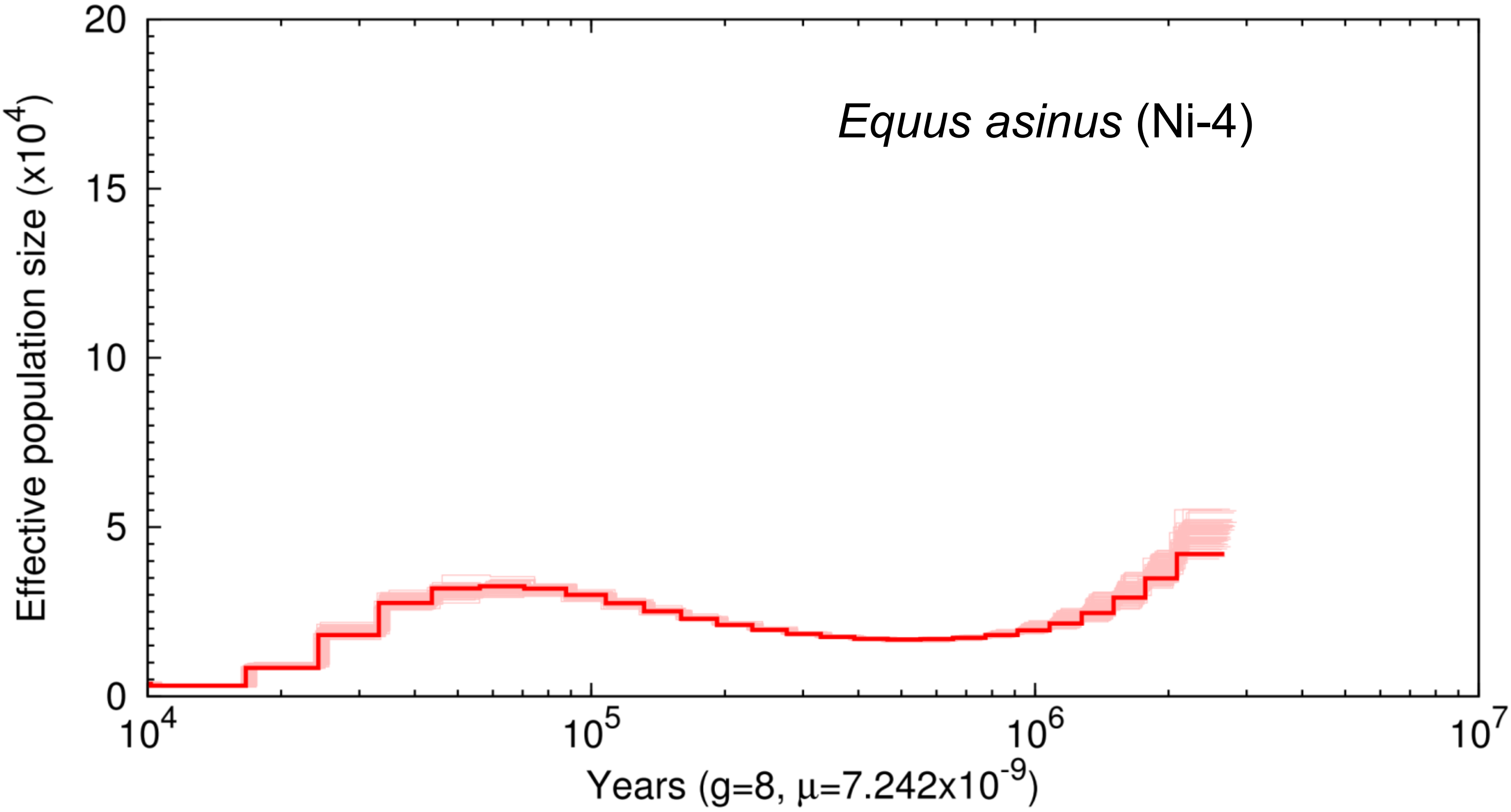
Equus asinus (Ni-4)
Effective population size (x10^4)
20
15
10
5
0
10^4
10^5
10^6
10^7
Years (g=8, μ=7.242x10^-9)

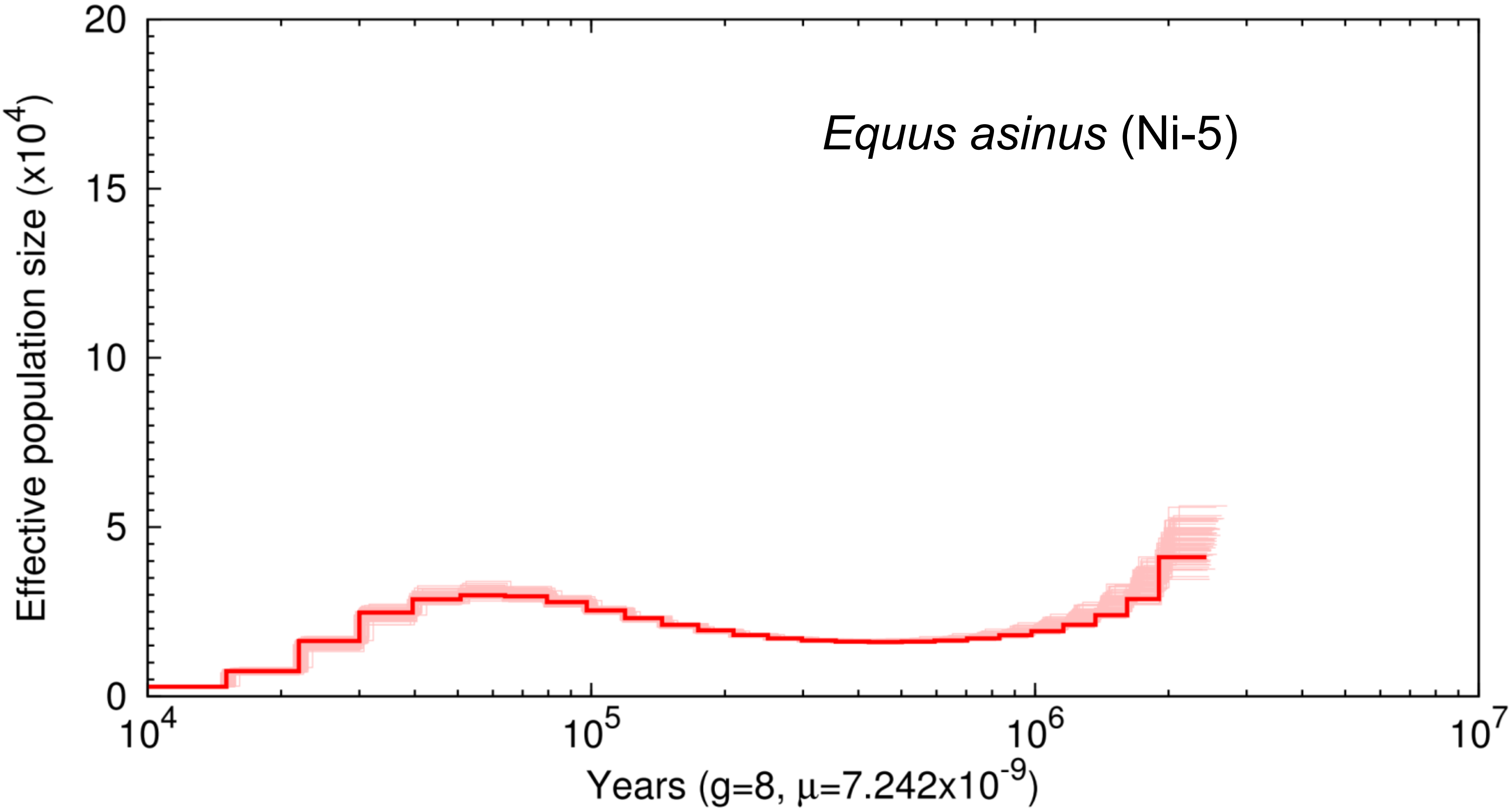
Equus asinus (Ni-5)
Effective population size (x10^4)
20
15
10
5
0
10^4
10^5
10^6
10^7
Years (g=8, μ=7.242x10^-9)

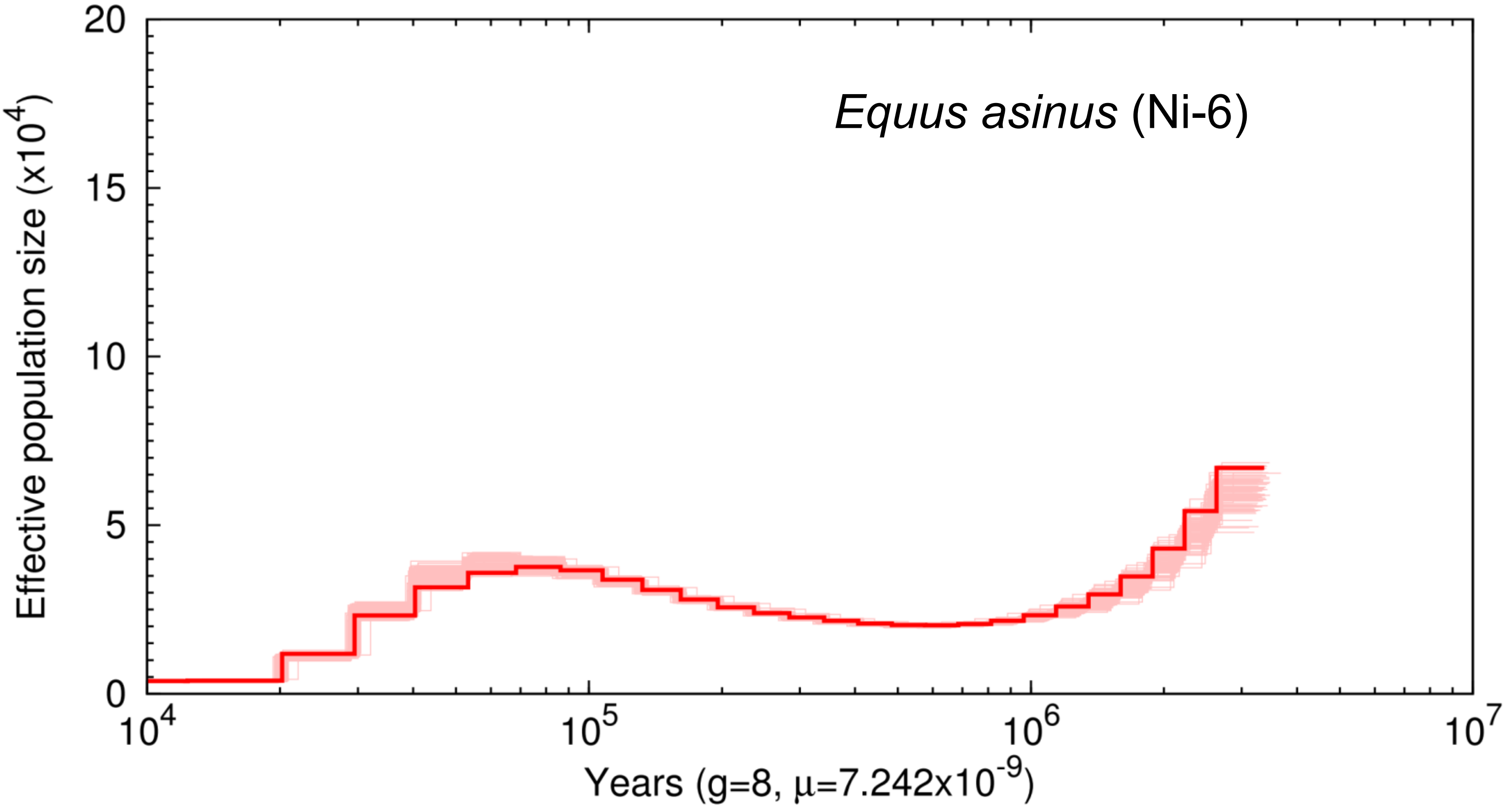
Equus asinus (Ni-6)
Effective population size (x10^4)
Years (g=8, μ=7.242x10^-9)
20
15
10
5
0
10^4
10^5
10^6
10^7

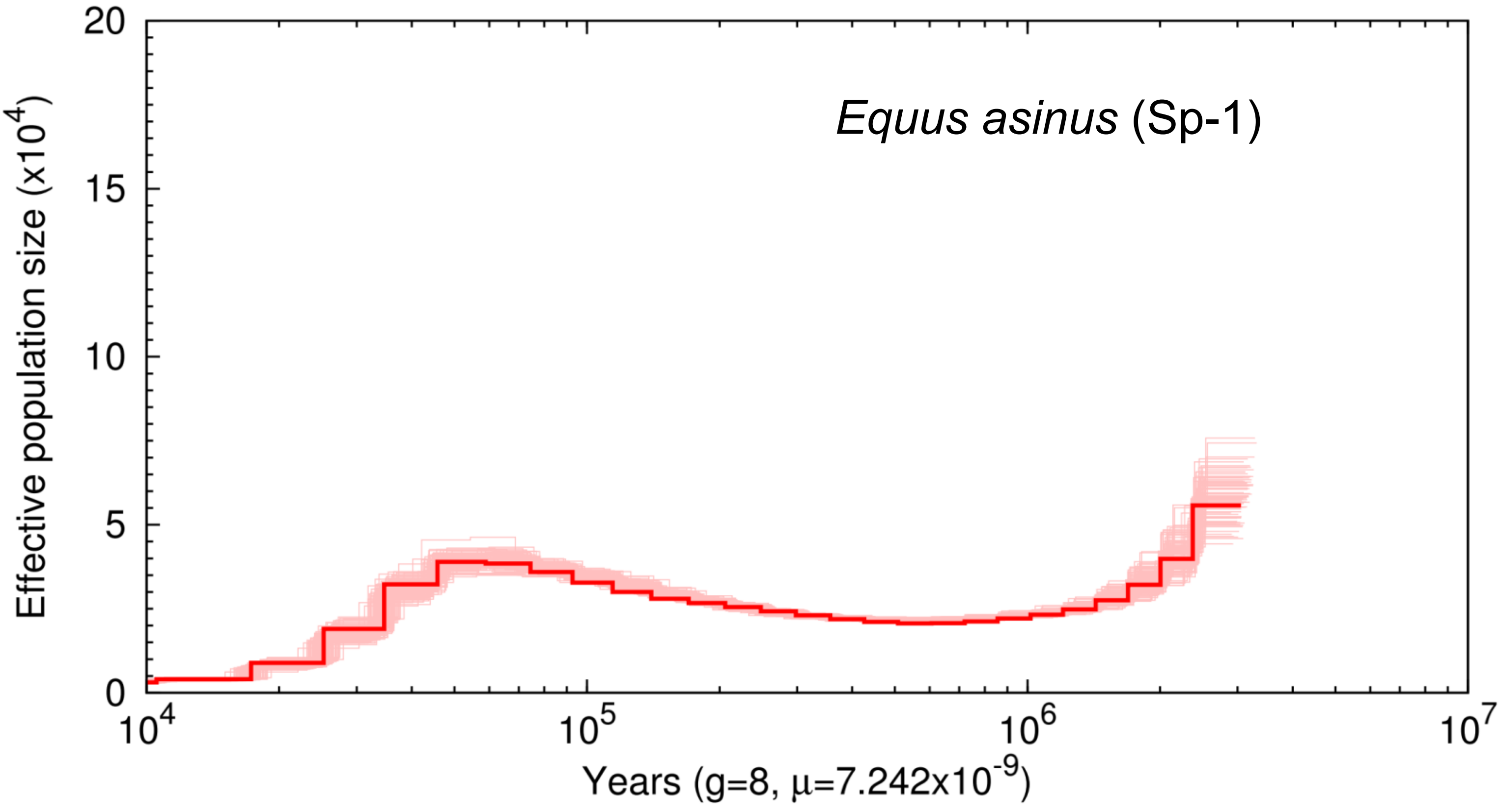
Equus asinus (Sp-1)
Effective population size (x10^4)
Years (g=8, μ=7.242x10^-9)
20
15
10
5
0
10^4
10^5
10^6
10^7

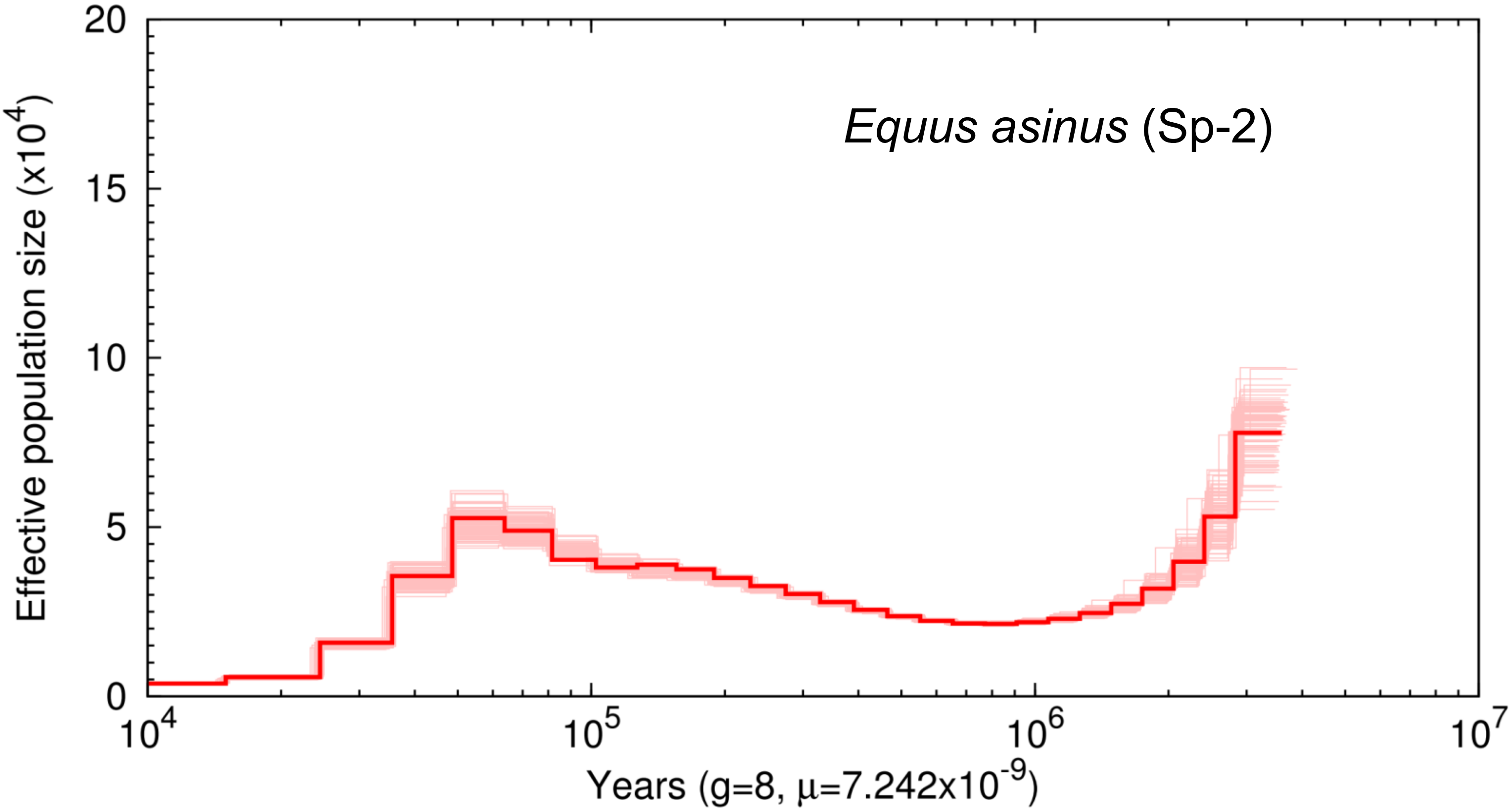
Equus asinus (Sp-2)
Effective population size (x10^4)
Years (g=8, μ=7.242x10^-9)
20
15
10
5
0
10^4
10^5
10^6
10^7

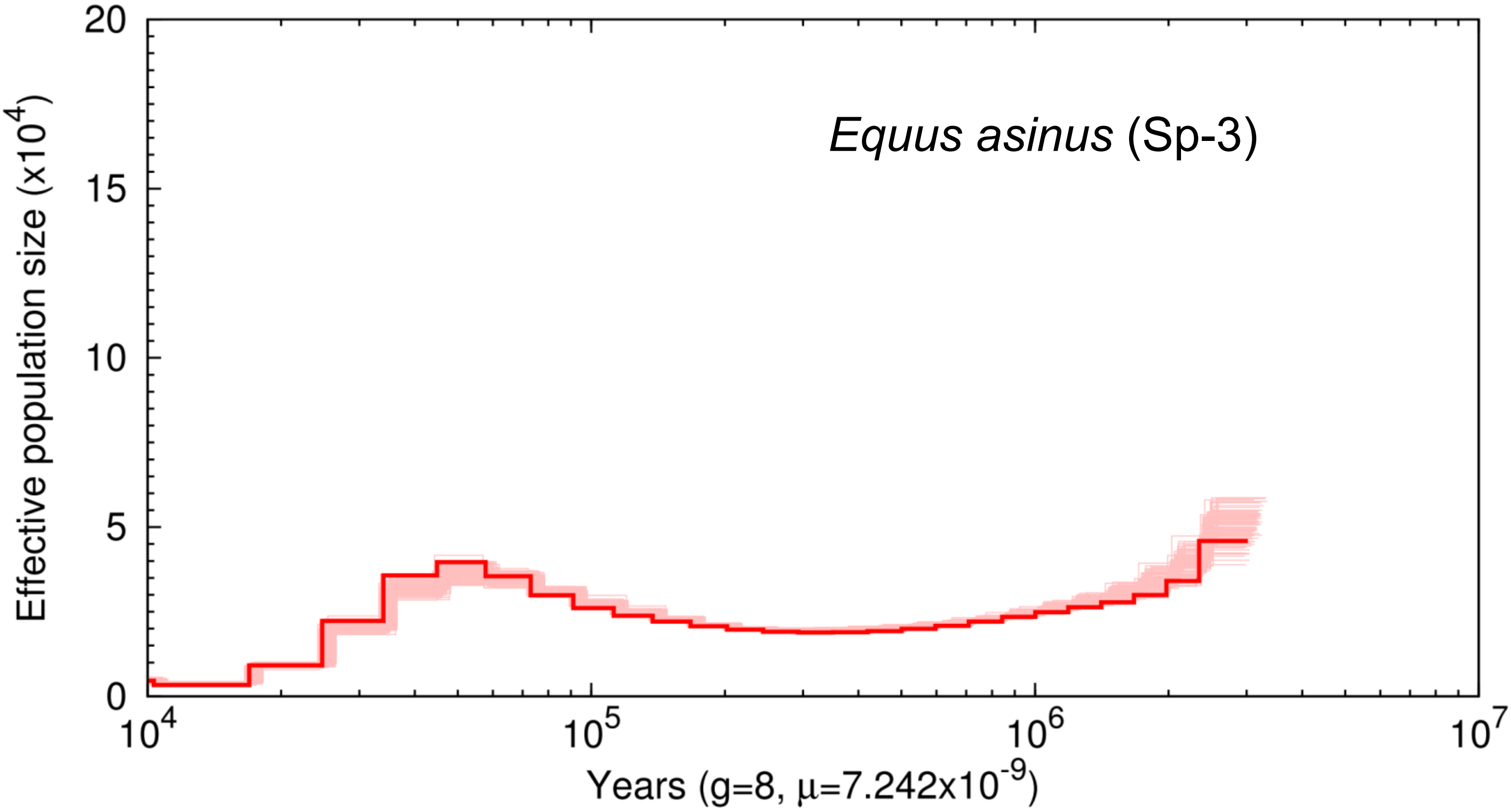
Equus asinus (Sp-3)
Effective population size (x10^4)
Years (g=8, μ=7.242x10^-9)
20
15
10
5
0
10^4
10^5
10^6
10^7

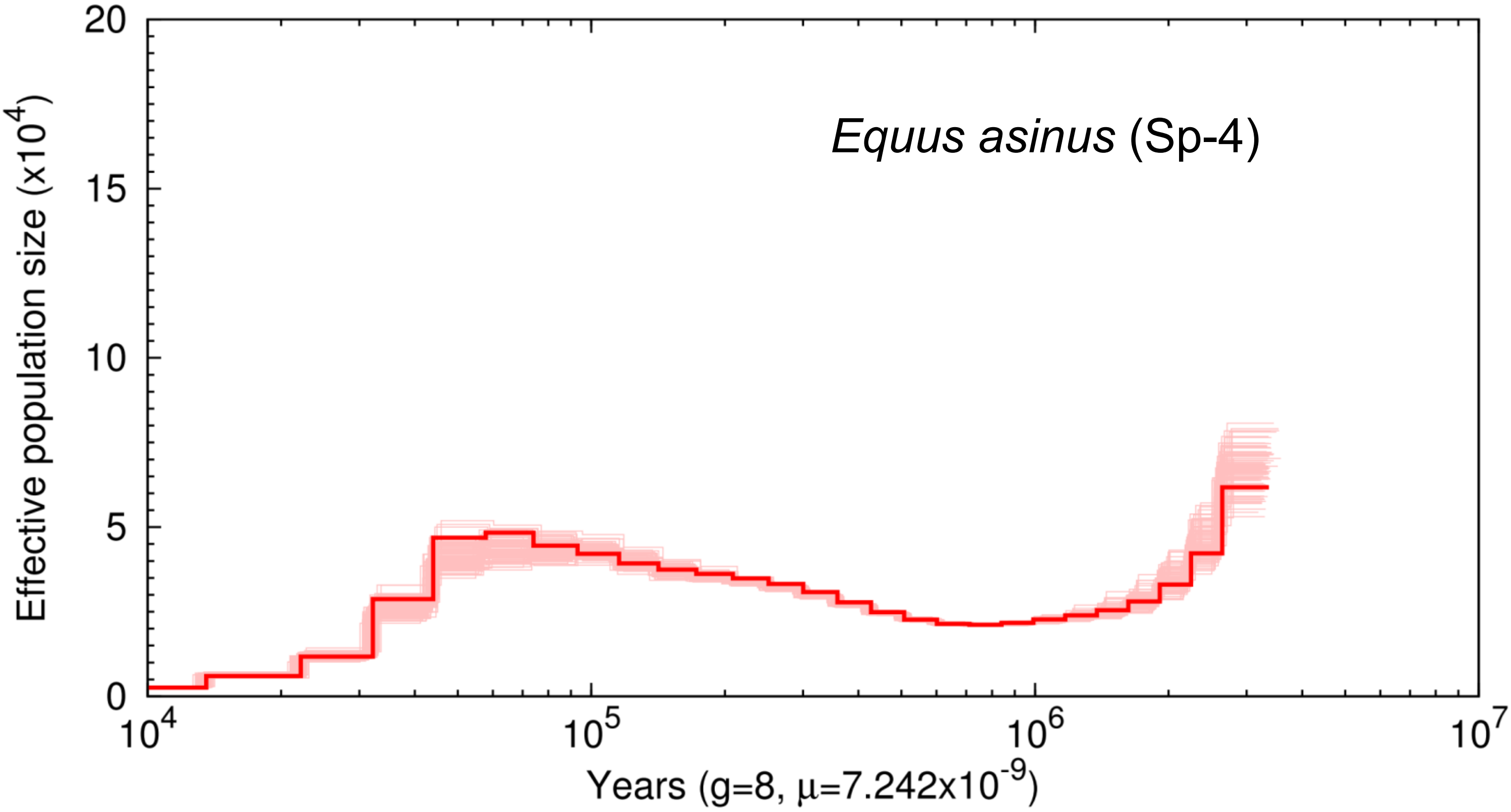
Equus asinus (Sp-4)
Effective population size (x10^4)
20
15
10
5
0
10^4
10^5
10^6
10^7
Years (g=8, μ=7.242x10^-9)

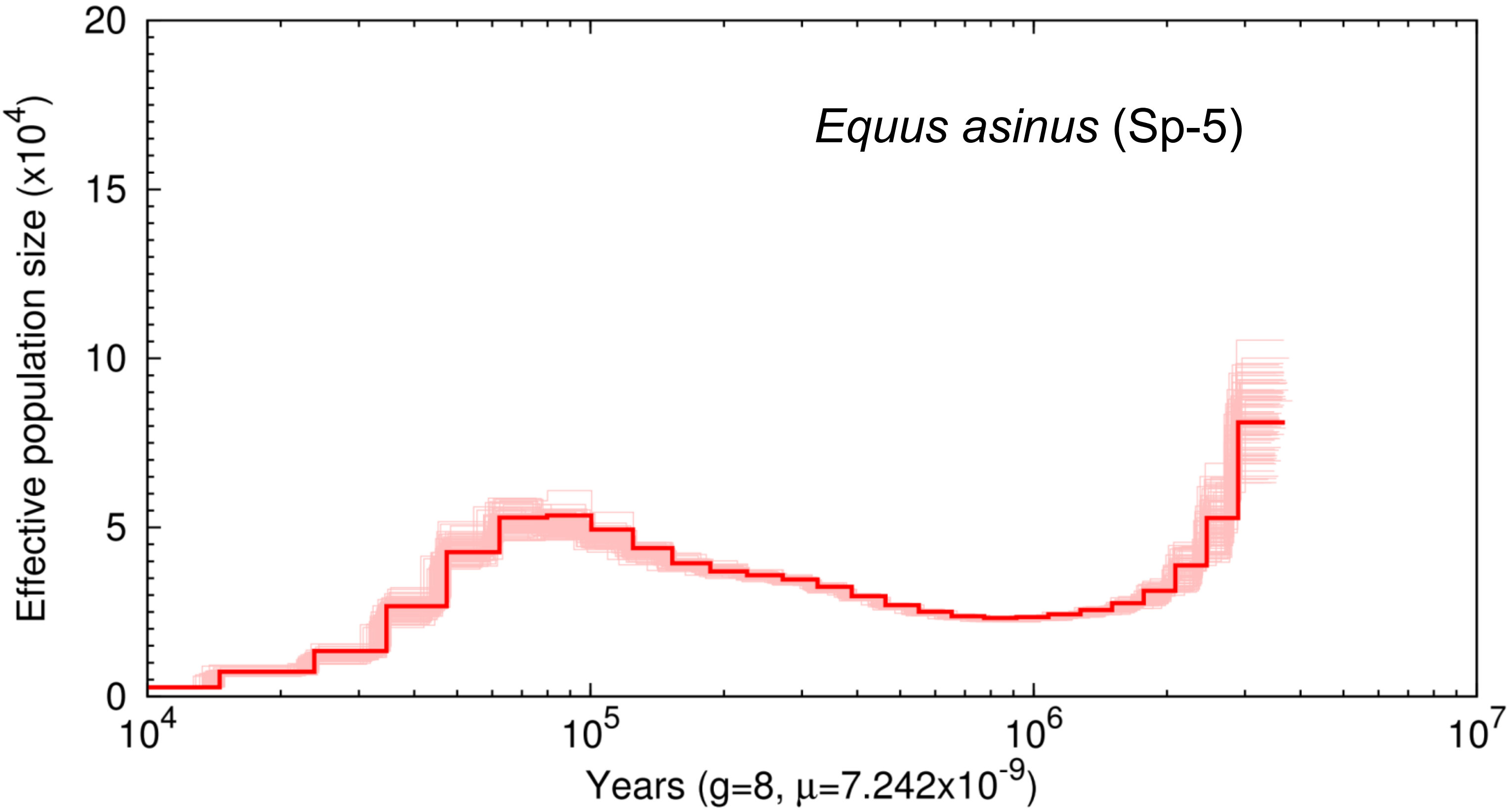
Equus asinus (Sp-5)
Effective population size (x10^4)
20
15
10
5
0
10^4
10^5
10^6
10^7
Years (g=8, μ=7.242x10^-9)

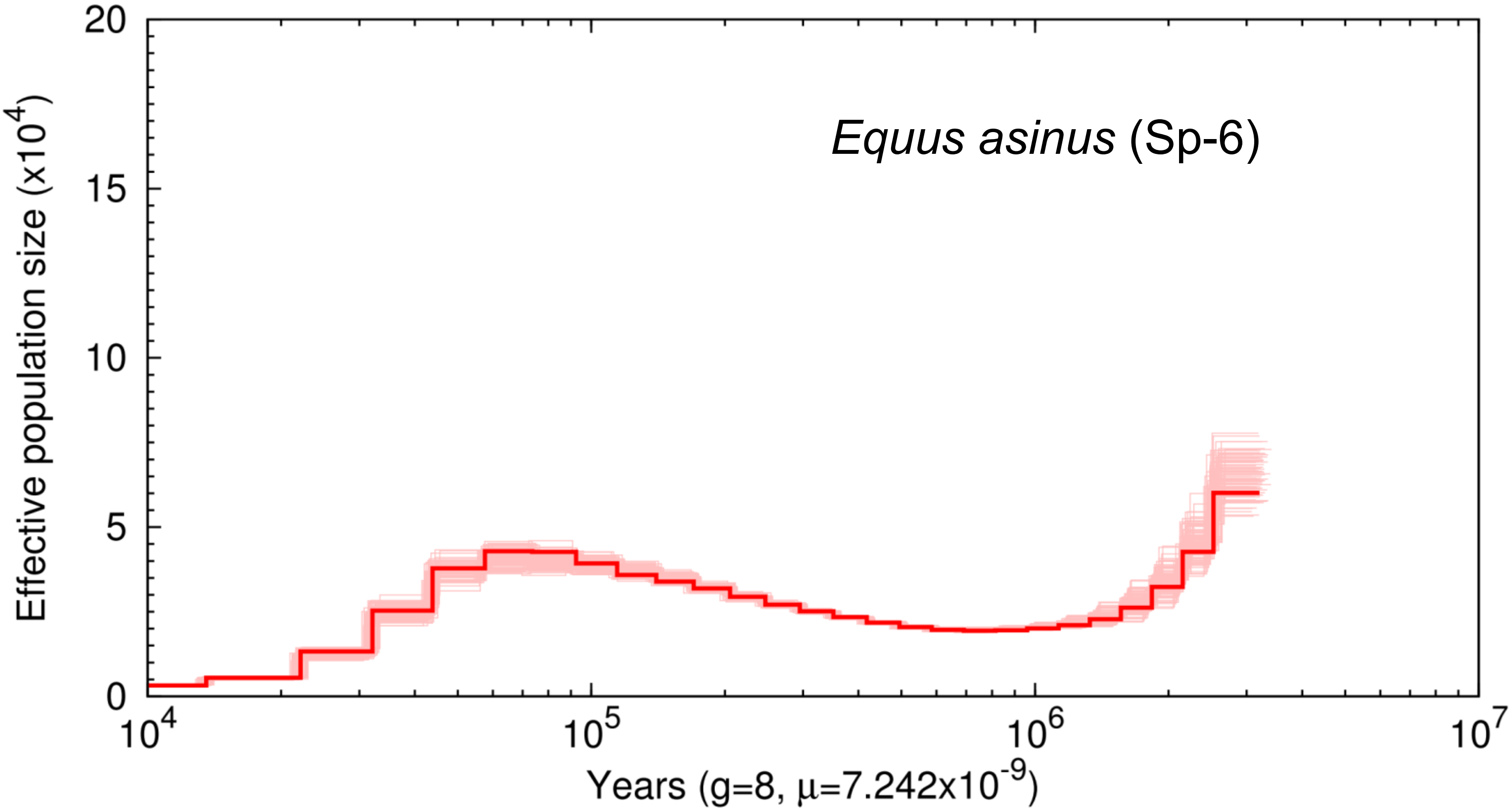

Equus asinus (Sp-6)
Effective population size (x10^4)
20
15
10
5
0
10^4
10^5
10^6
10^7
Years (g=8, μ=7.242x10^-9)


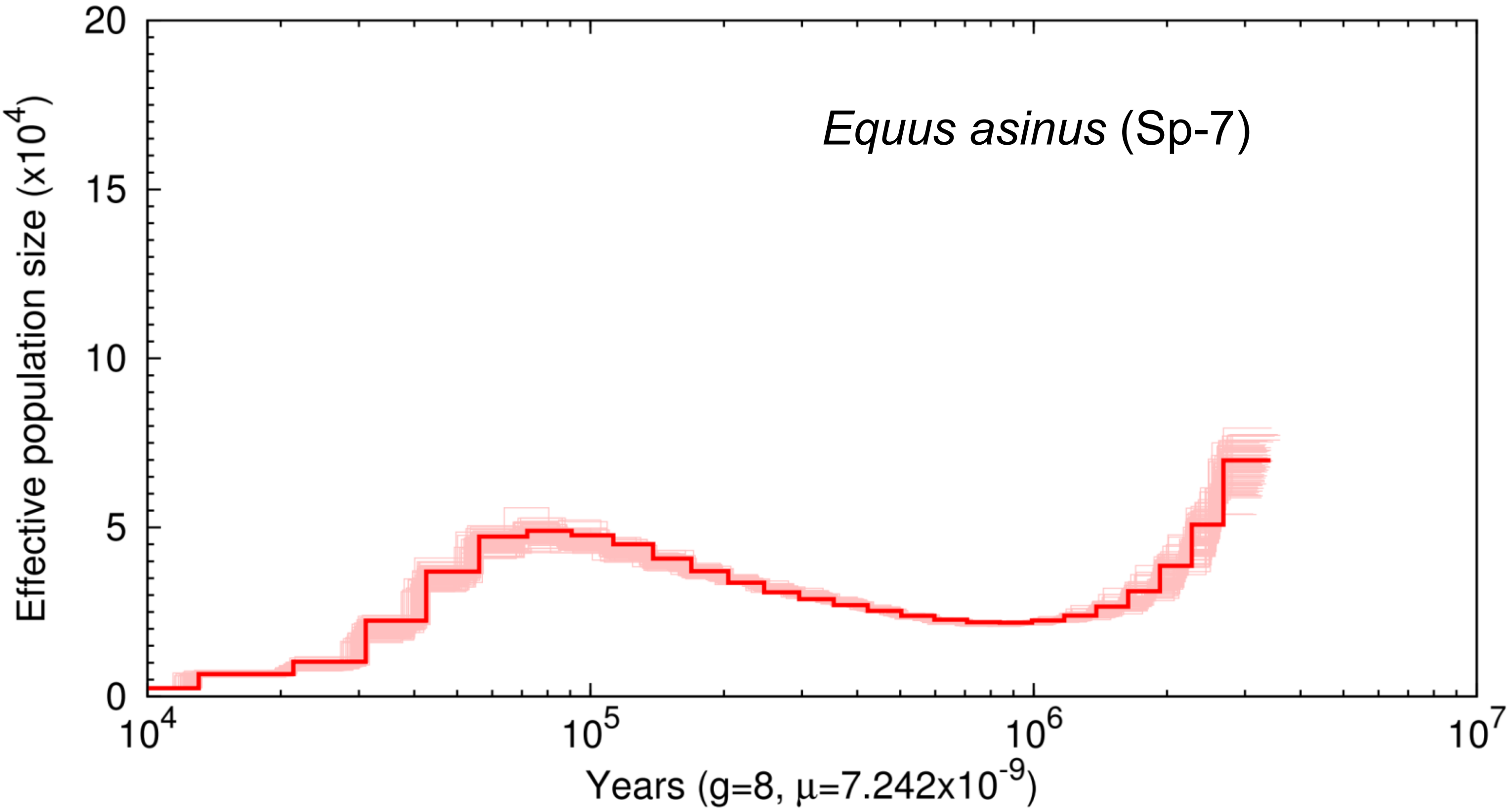

Equus asinus (Sp-7)
Effective population size (x10^4)
20
15
10
5
0
10^4
10^5
10^6
10^7
Years (g=8, μ=7.242x10^-9)

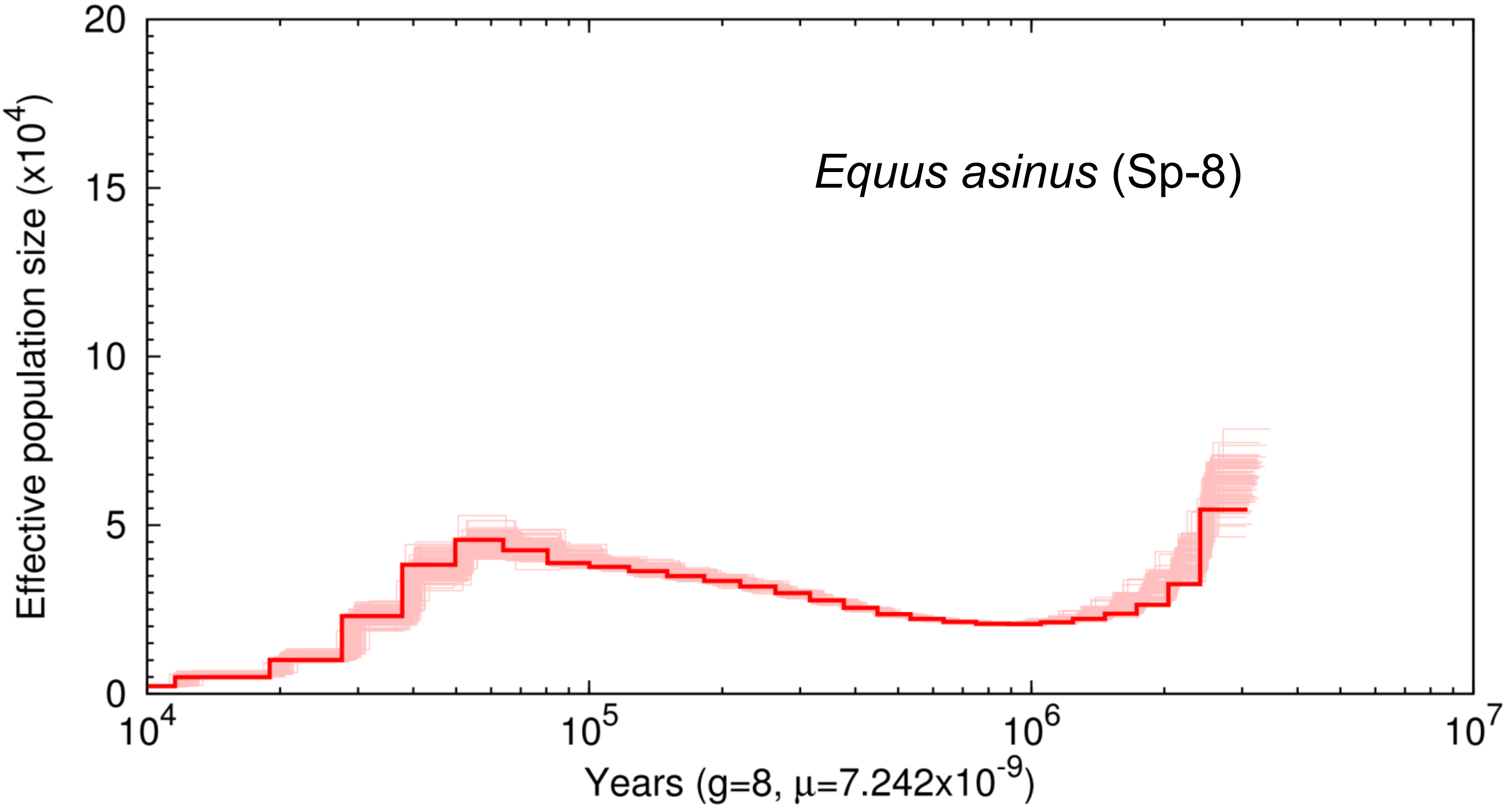
Equus asinus (Sp-8)
Effective population size (x10^4)
0
5
10
15
20
10^4
10^5
10^6
10^7
Years (g=8, μ=7.242x10^-9)

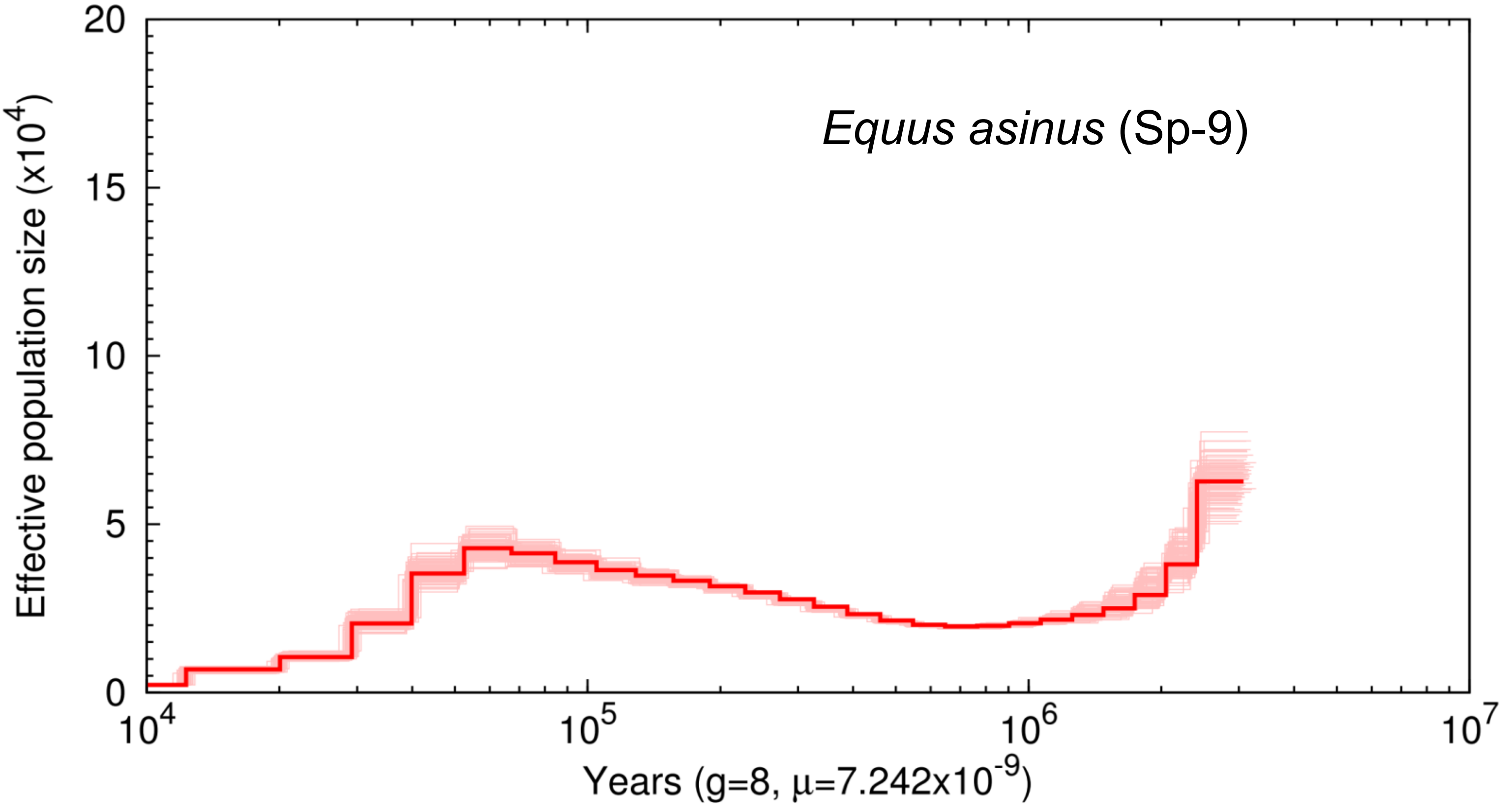
Equus asinus (Sp-9)
Effective population size (x10^4)
0
5
10
15
20
10^4
10^5
10^6
10^7
Years (g=8, μ=7.242x10^-9)

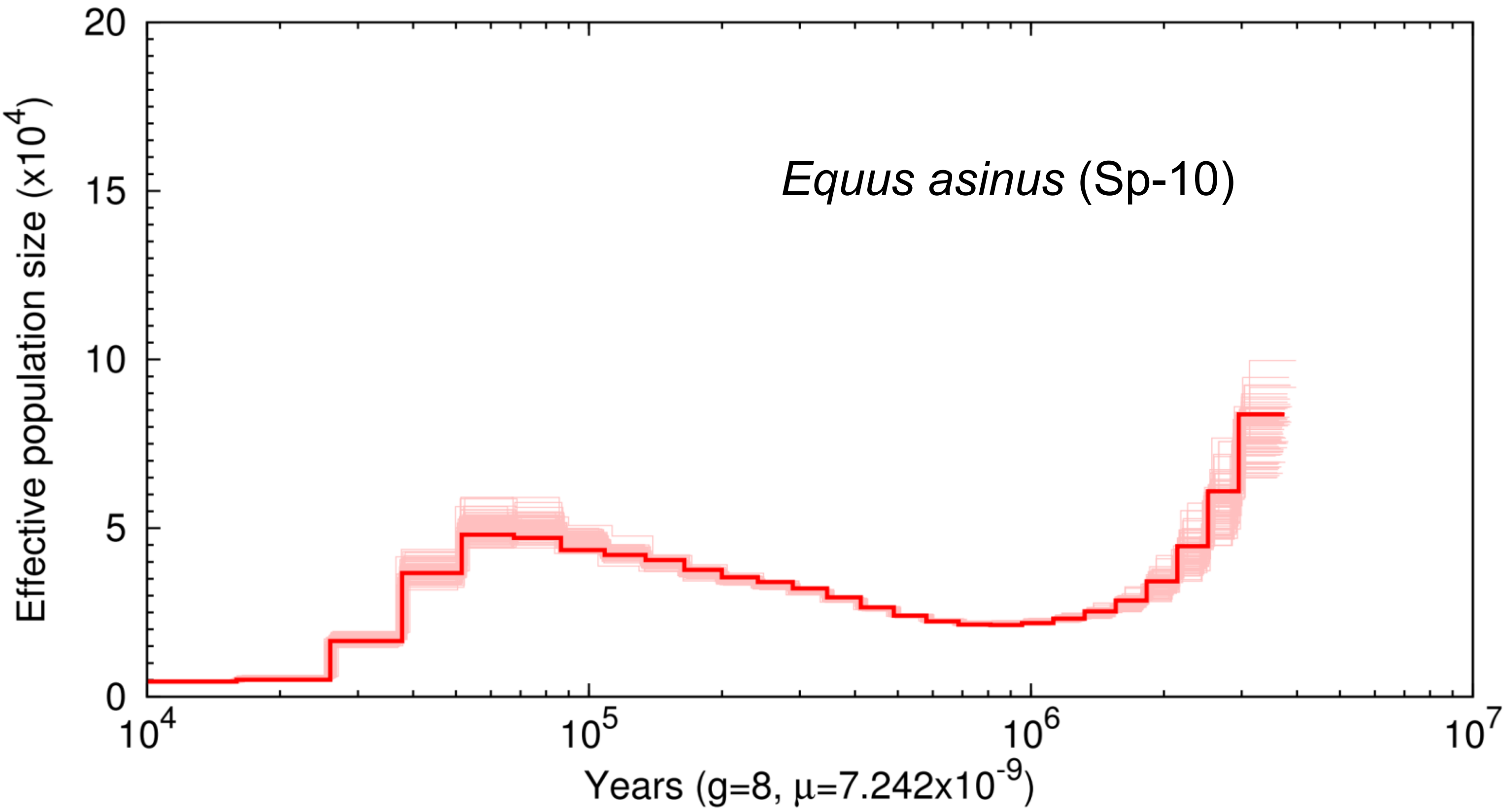
Equus asinus (Sp-10)
Effective population size (x10^4)
20
15
10
5
0
10^4
10^5
10^6
10^7
Years (g=8, μ=7.242x10^-9)

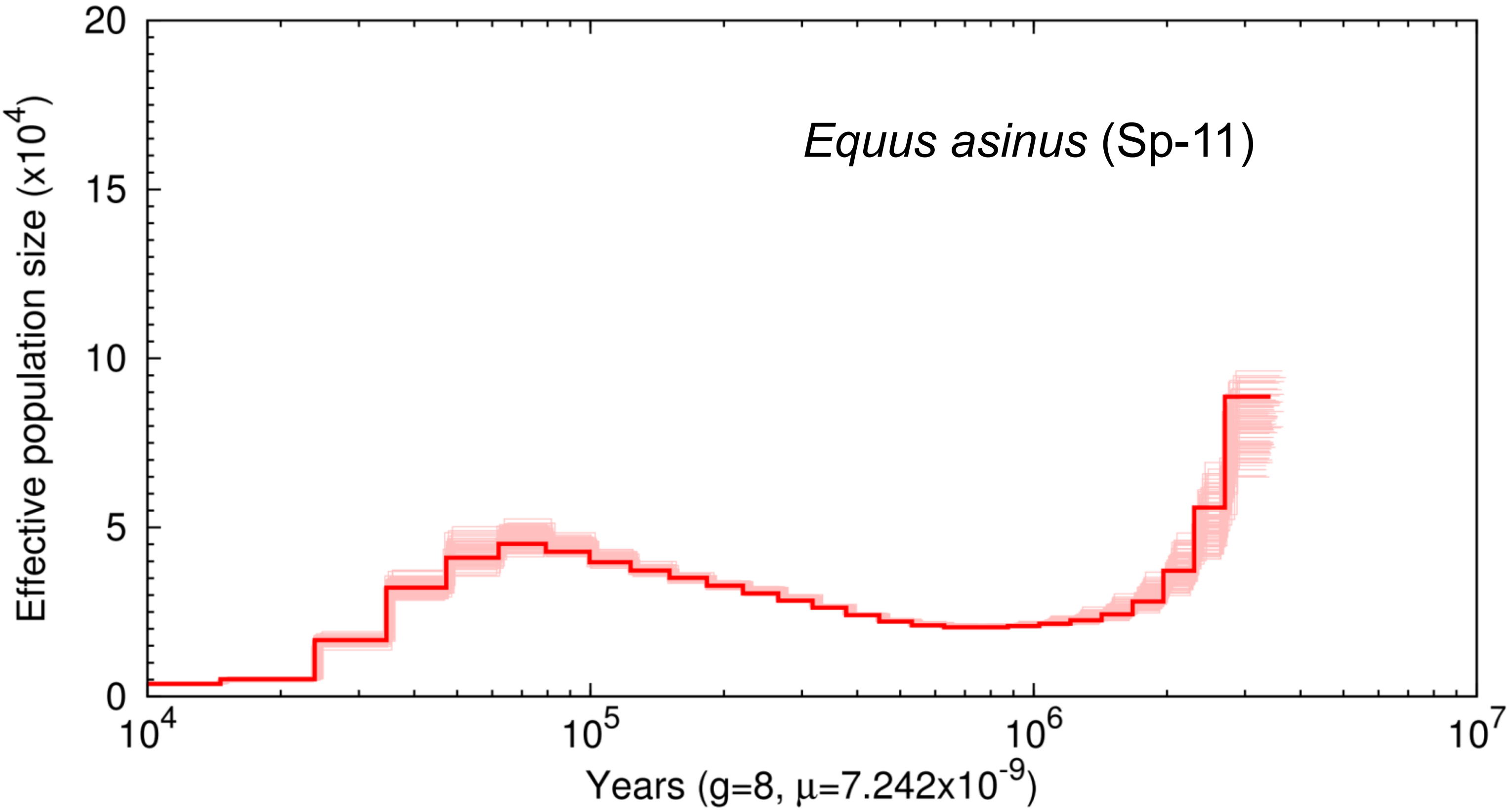
Equus asinus (Sp-11)
Effective population size (x10^4)
20
15
10
5
0
10^4
10^5
10^6
10^7
Years (g=8, μ=7.242x10^-9)

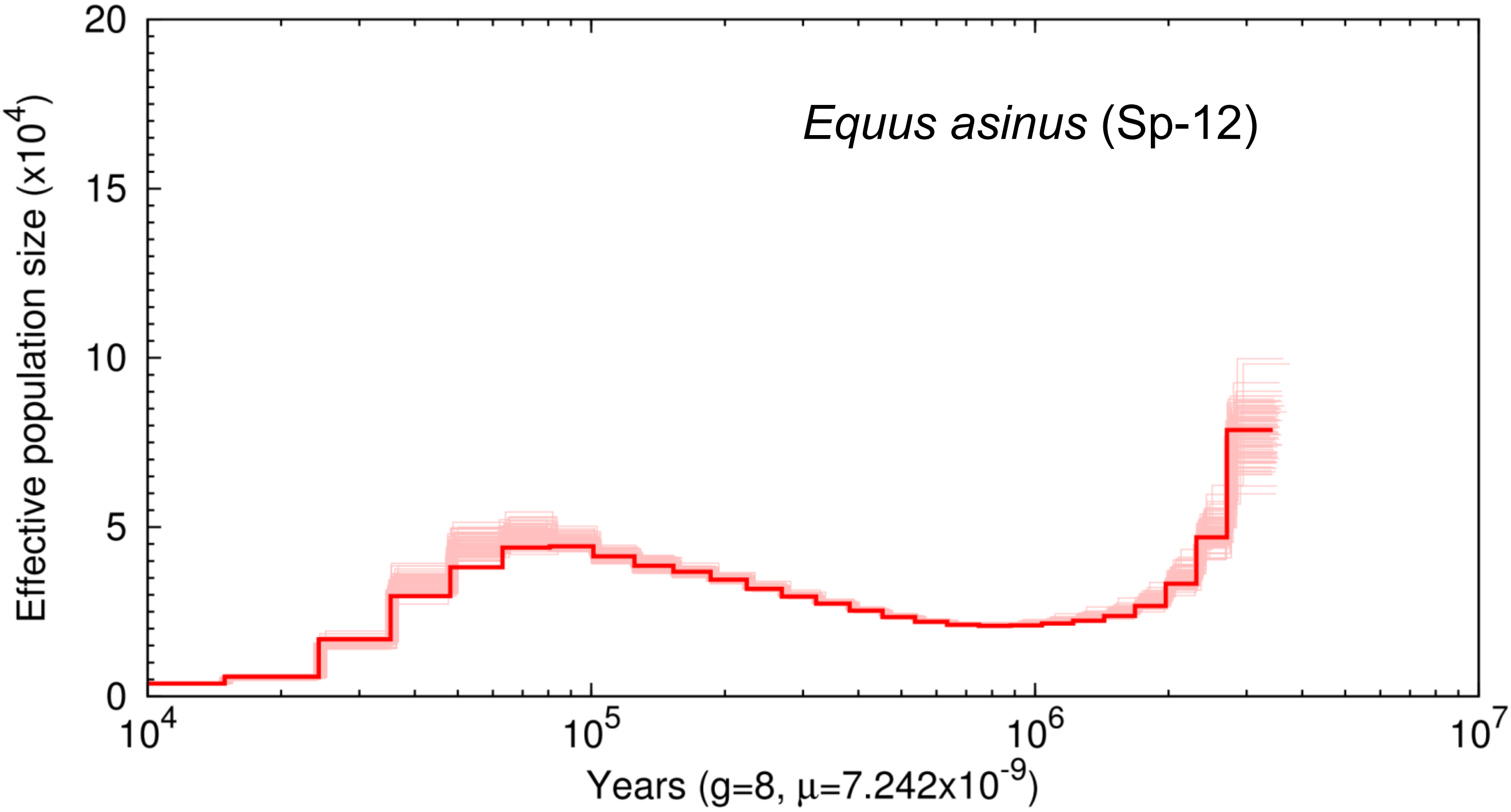
Equus asinus (Sp-12)
Effective population size (x10^4)
Years (g=8, μ=7.242x10^-9)
0
5
10
15
20
10^4
10^5
10^6
10^7

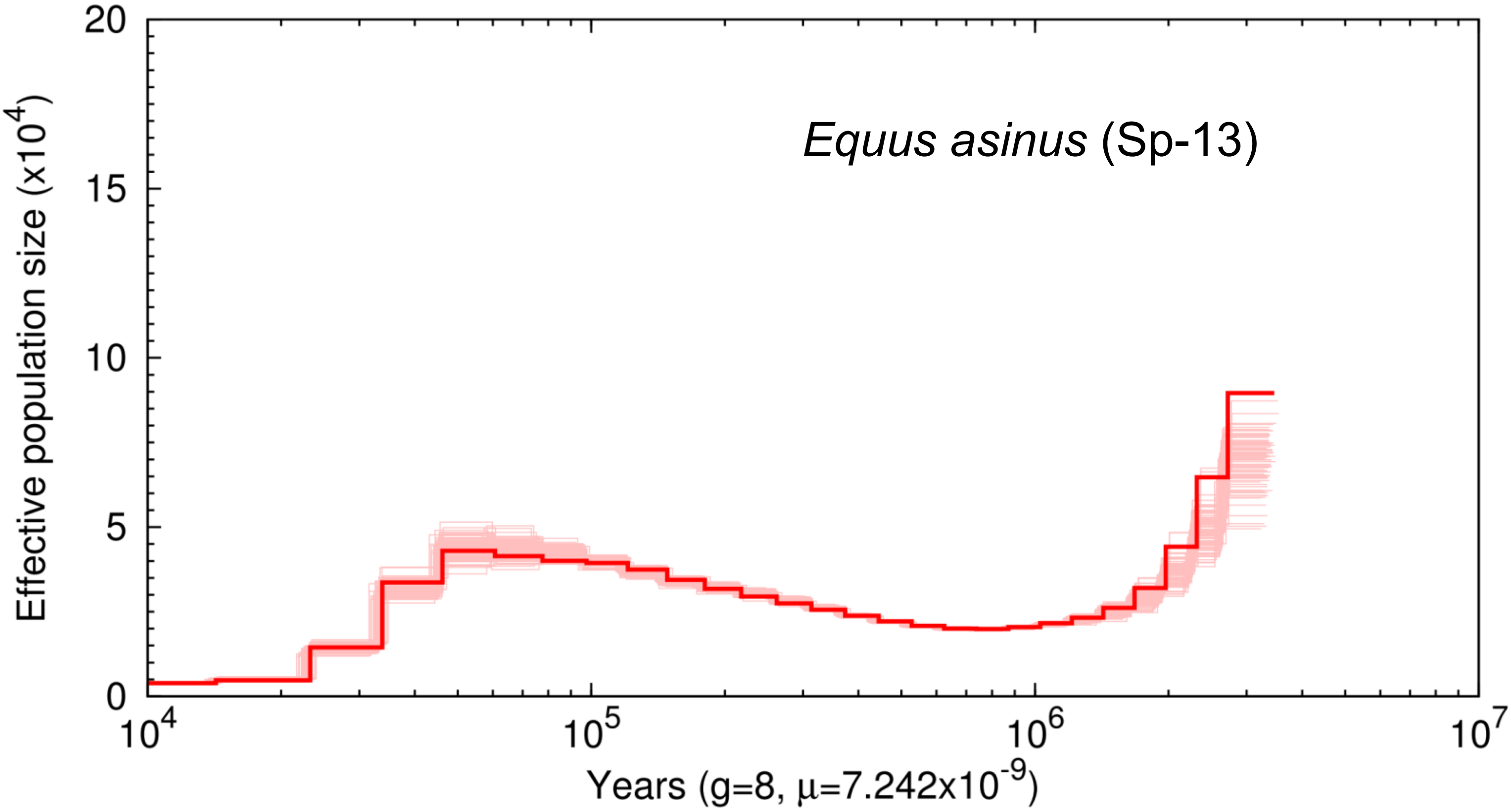
Equus asinus (Sp-13)
Effective population size (x10^4)
Years (g=8, μ=7.242x10^-9)
0
5
10
15
20
10^4
10^5
10^6
10^7

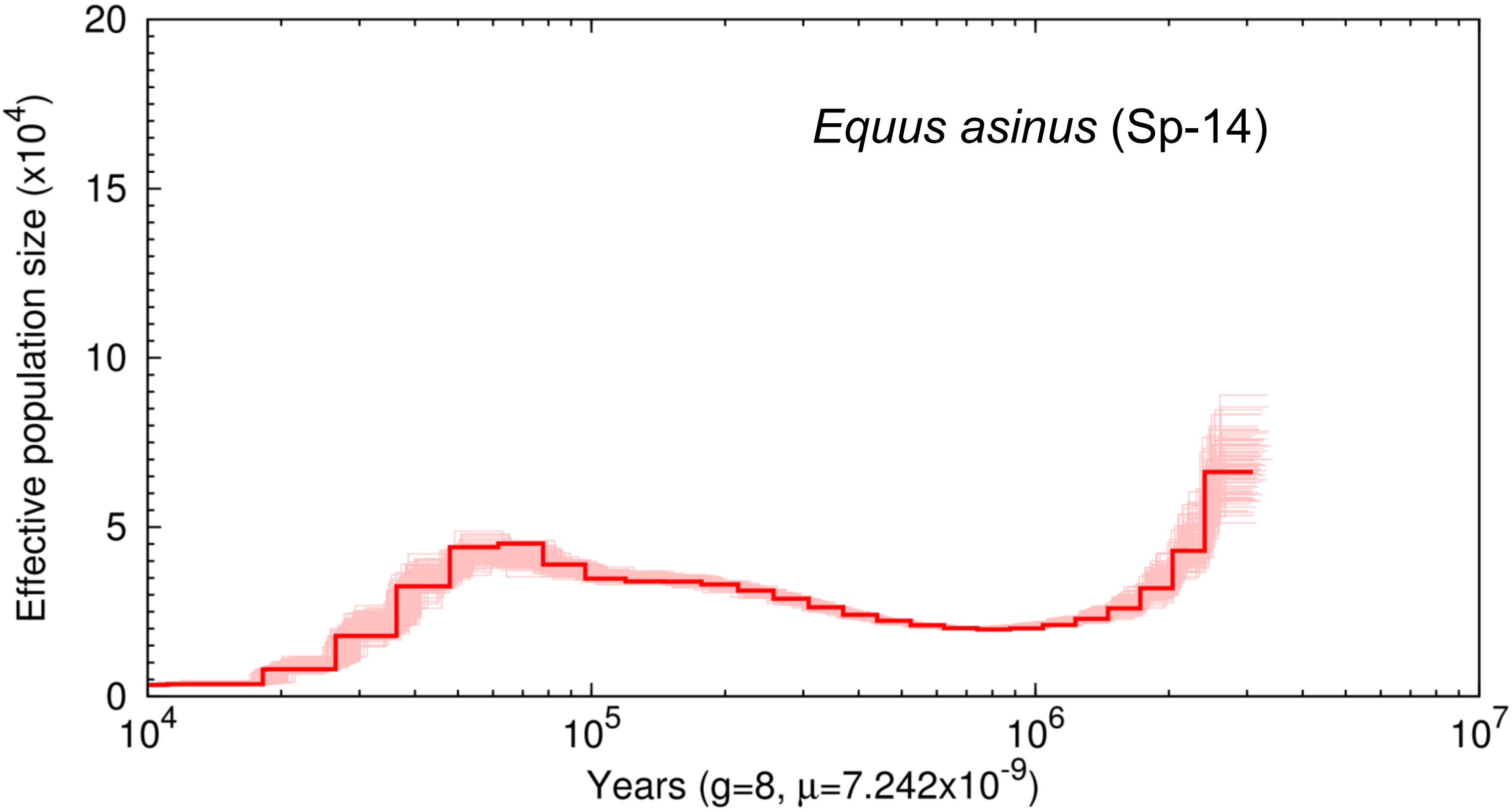
Equus asinus (Sp-14)
Effective population size (x10^4)
20
15
10
5
0
10^4
10^5
10^6
10^7
Years (g=8, μ=7.242x10^-9)

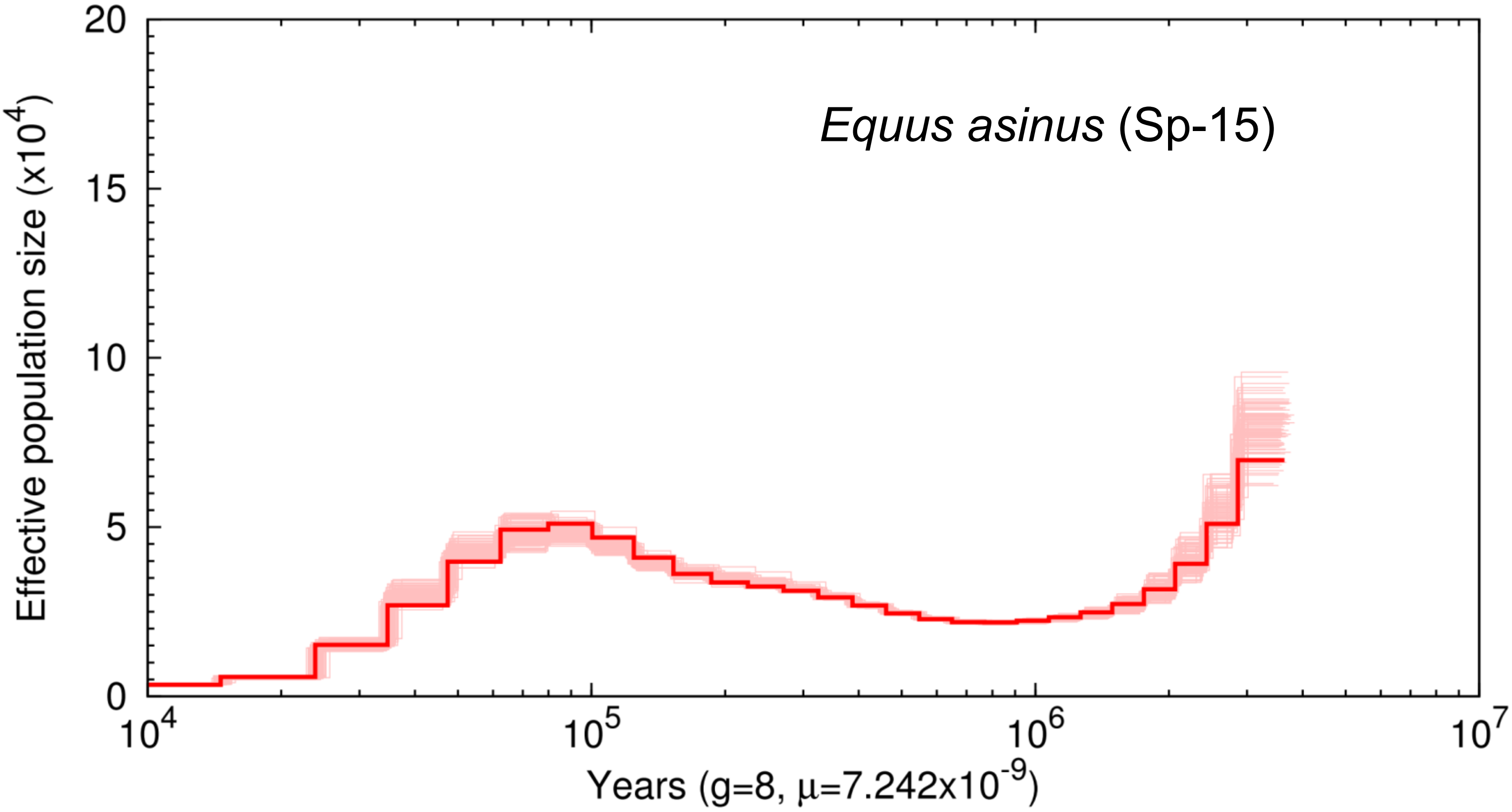
Equus asinus (Sp-15)
Effective population size (x10^4)
20
15
10
5
0
10^4
10^5
10^6
10^7
Years (g=8, μ=7.242x10^-9)

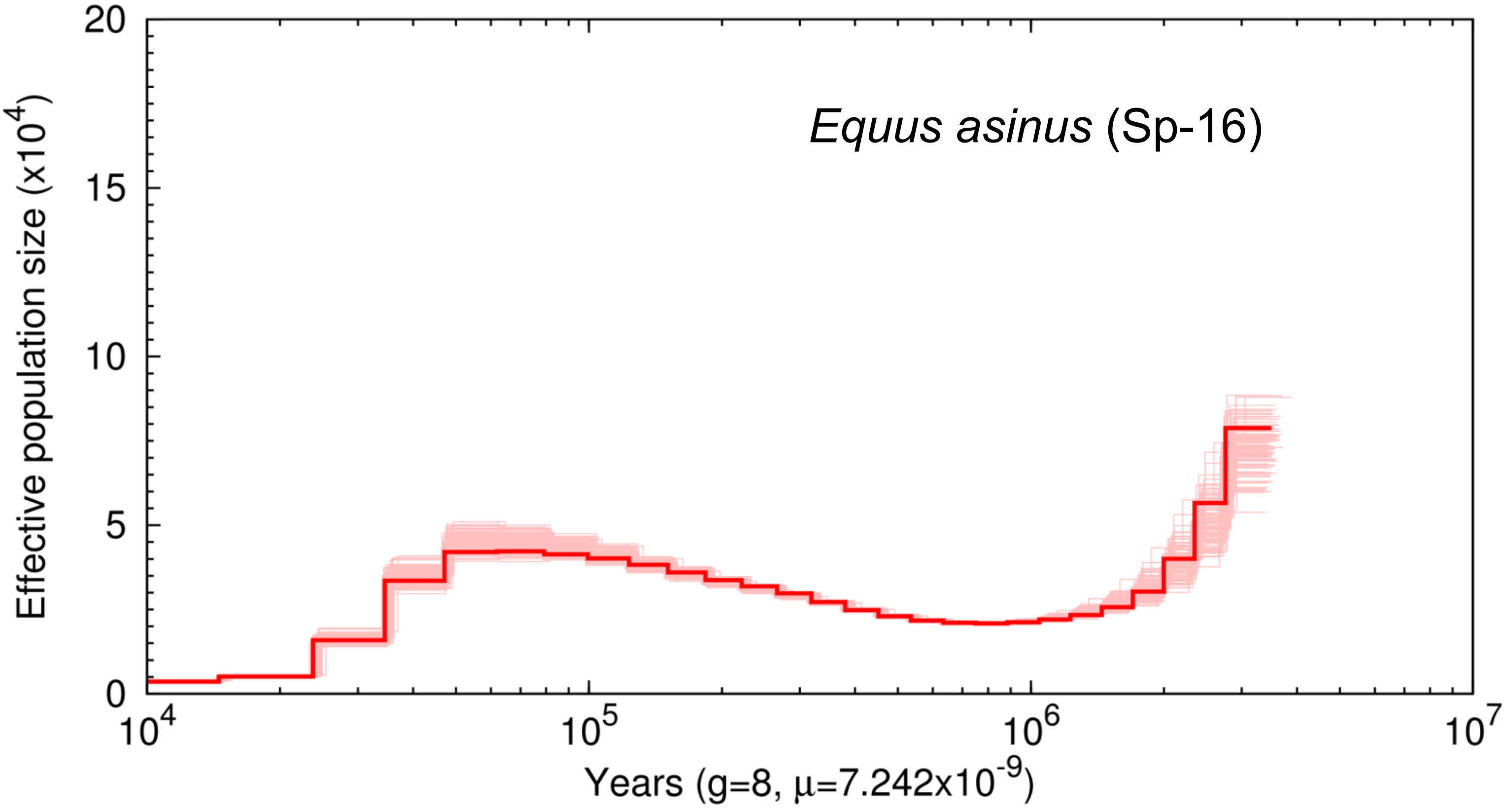
Equus asinus (Sp-16)
Effective population size (x10^4)
Years (g=8, μ=7.242x10^-9)
0
5
10
15
20
10^4
10^5
10^6
10^7

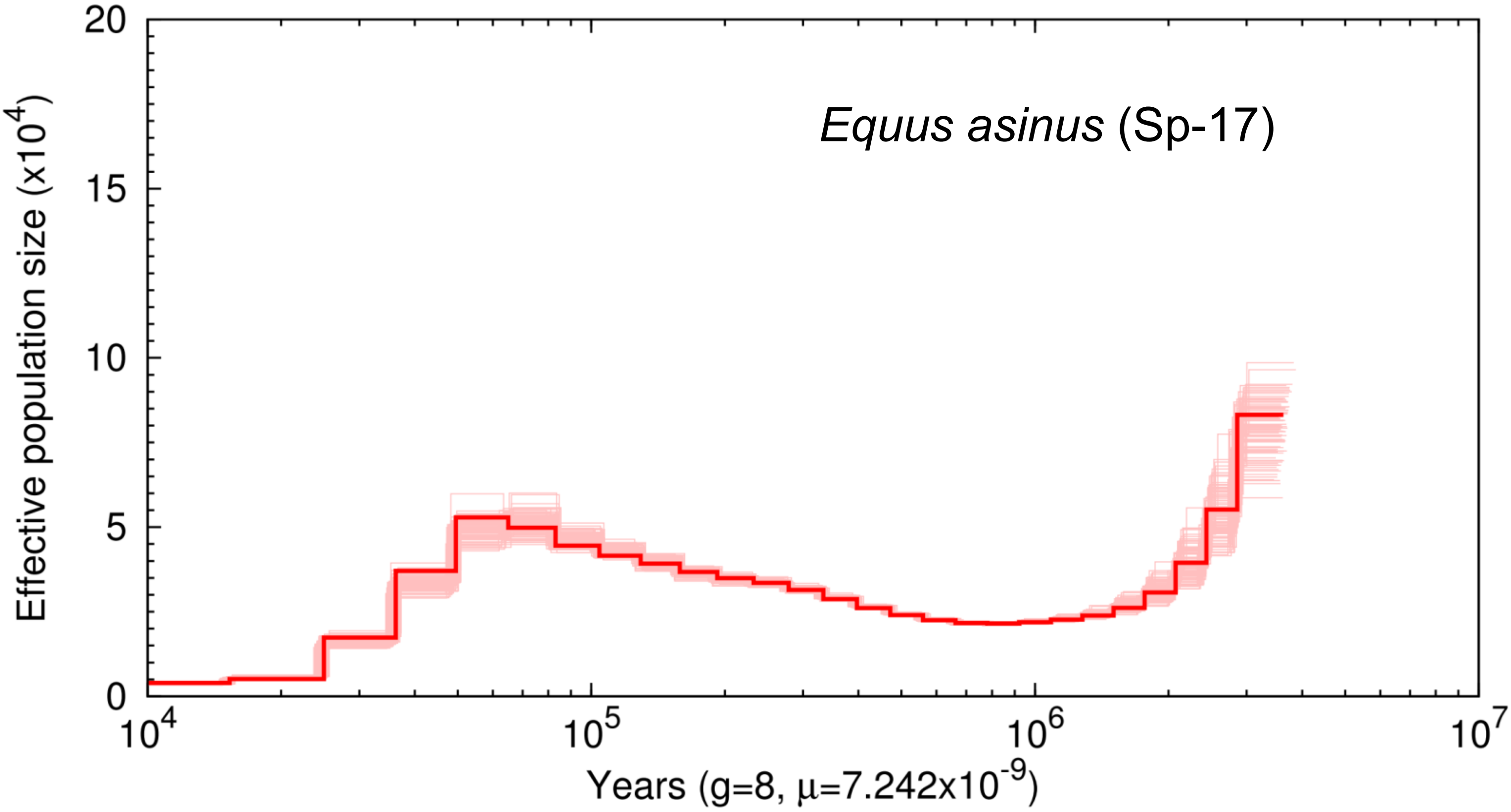
Equus asinus (Sp-17)
Effective population size (x10^4)
Years (g=8, μ=7.242x10^-9)
0
5
10
15
20
10^4
10^5
10^6
10^7

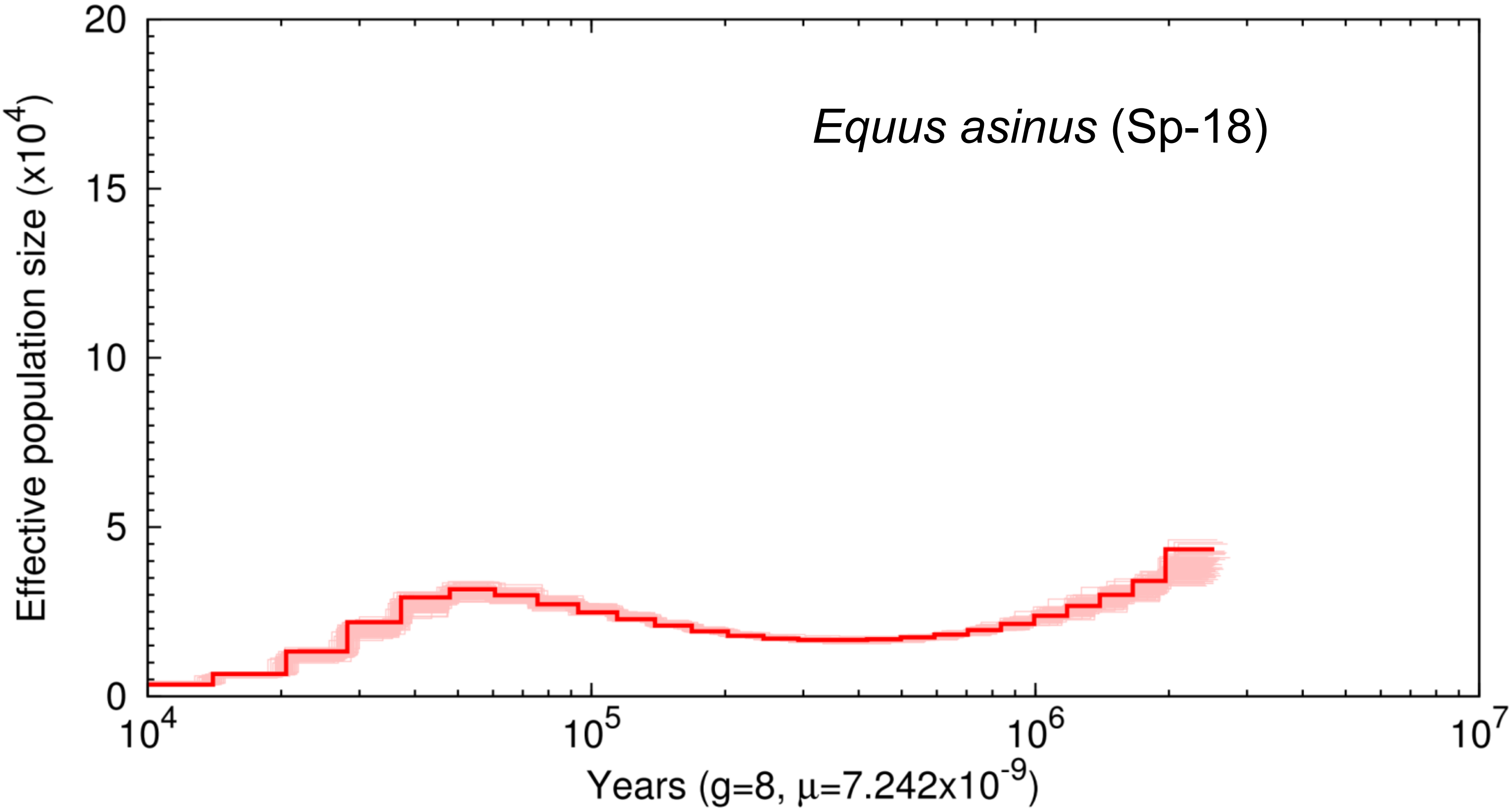

Equus asinus (Sp-18)
Effective population size (x10^4)
20
15
10
5
0
10^4
10^5
10^6
10^7
Years (g=8, μ=7.242x10^-9)


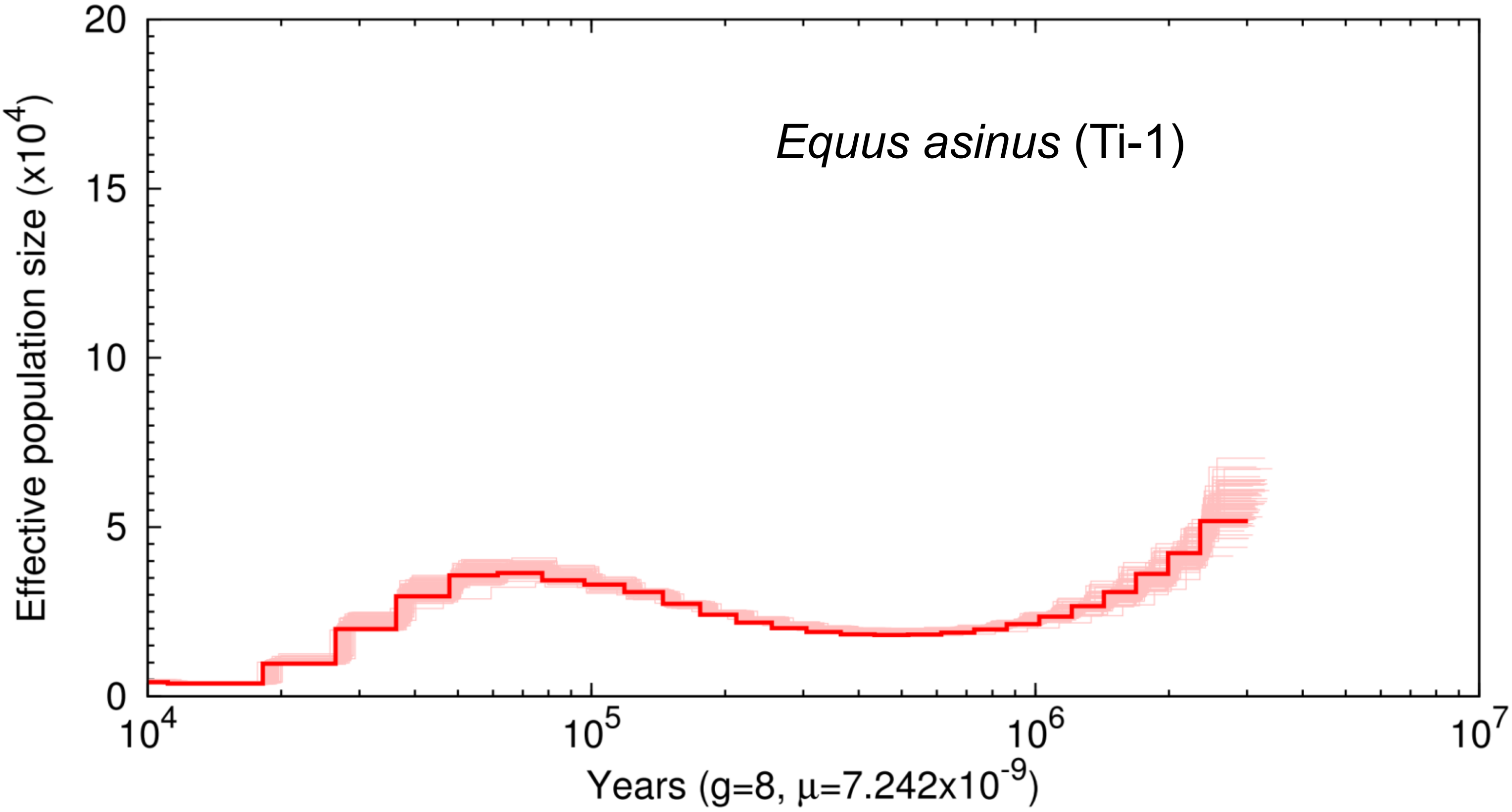

Equus asinus (Ti-1)
Effective population size (x10^4)
20
15
10
5
0
10^4
10^5
10^6
10^7
Years (g=8, μ=7.242x10^-9)

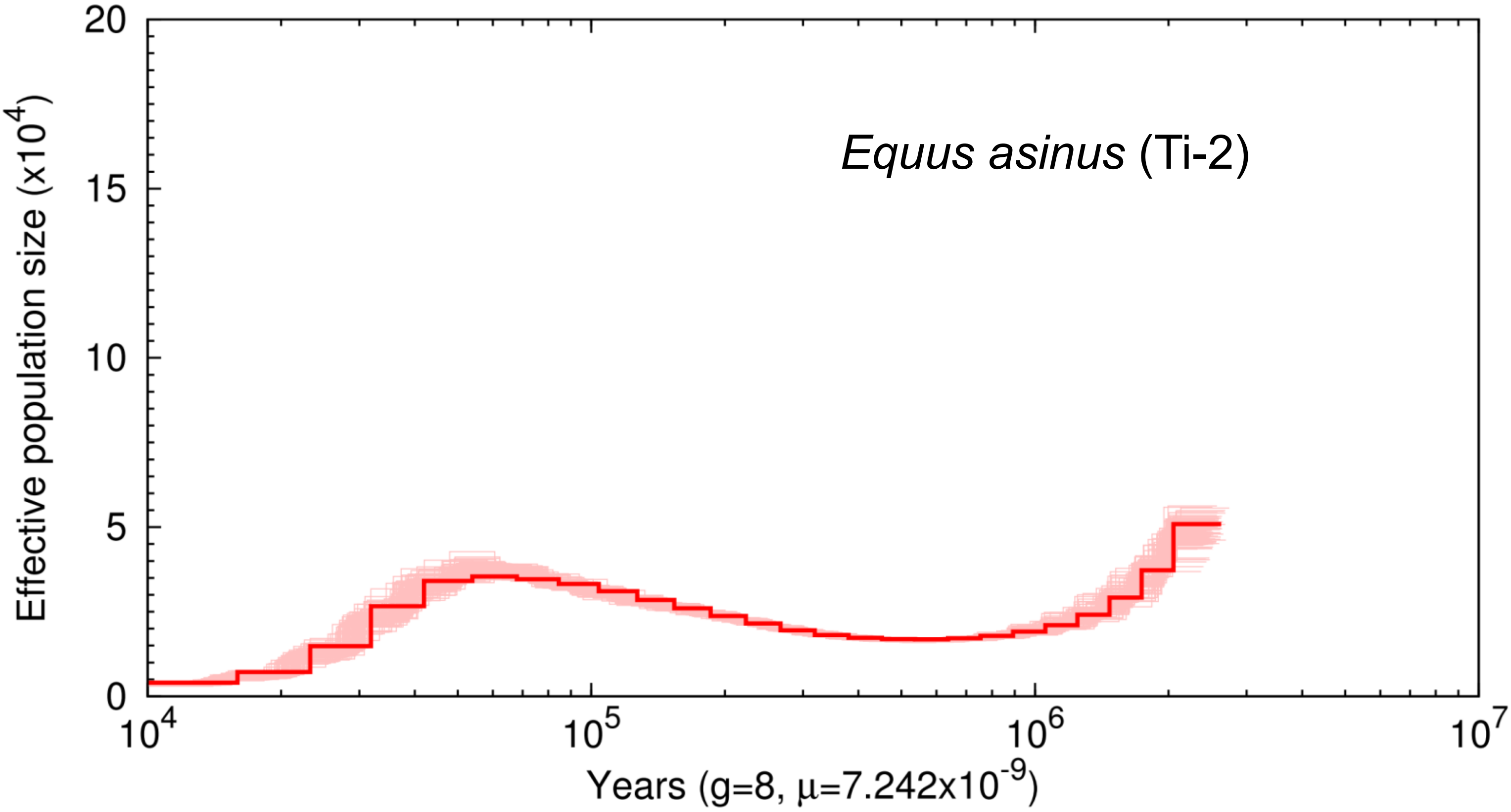
Equus asinus (Ti-2)
Effective population size (x10^4)
20
15
10
5
0
10^4
10^5
10^6
10^7
Years (g=8, μ=7.242x10^-9)

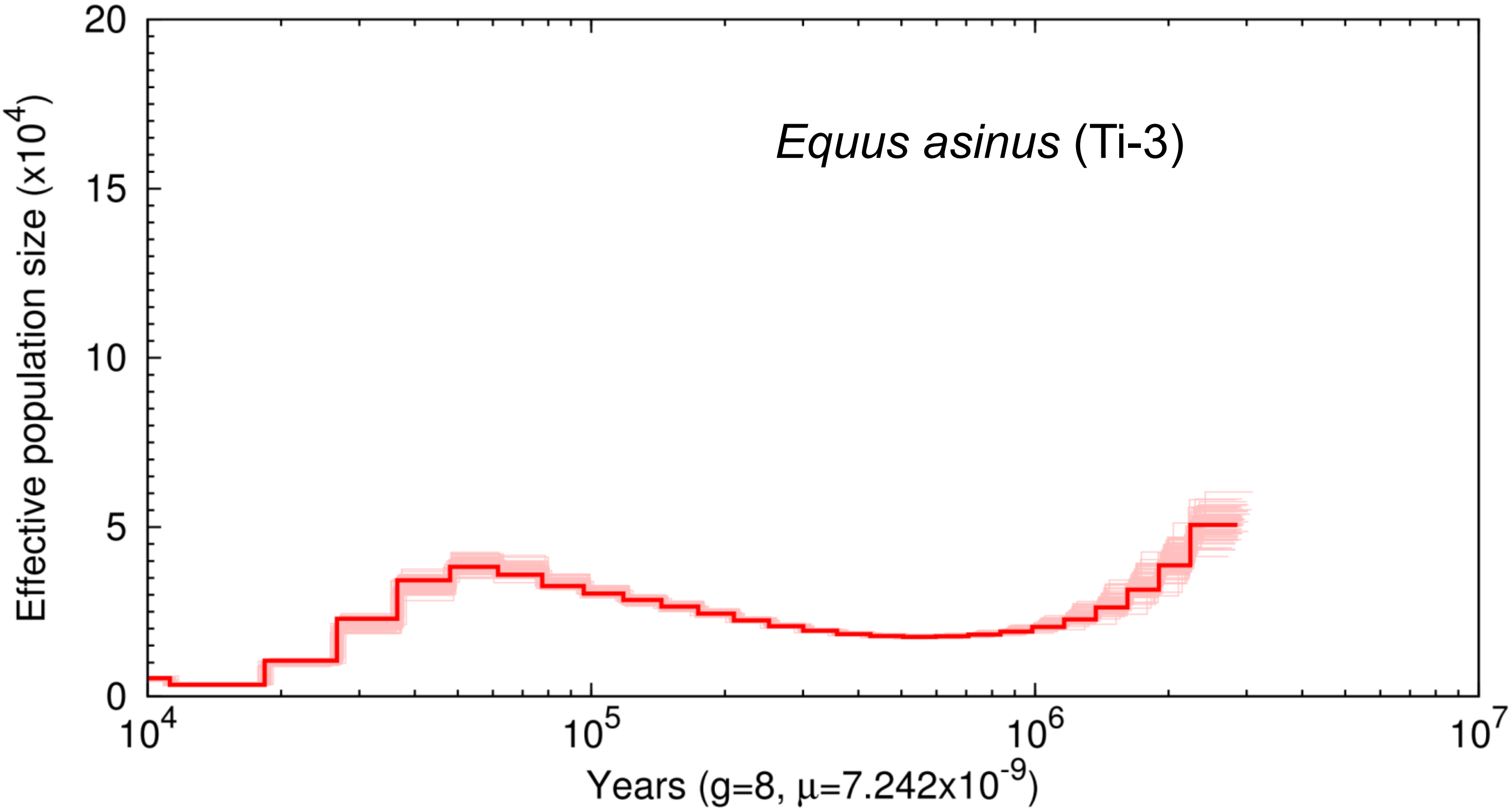
Equus asinus (Ti-3)
Effective population size (x10^4)
20
15
10
5
0
10^4
10^5
10^6
10^7
Years (g=8, μ=7.242x10^-9)

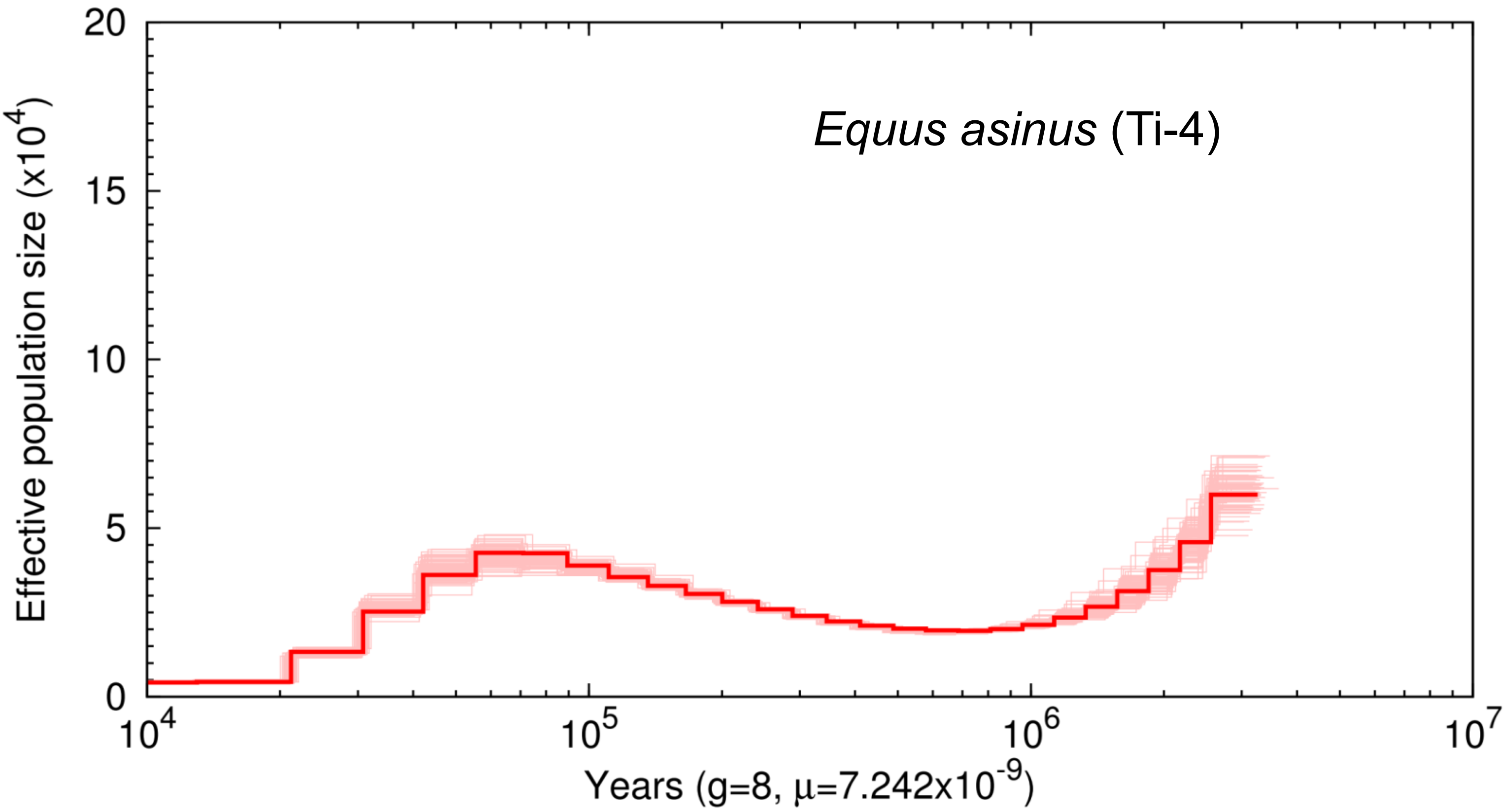

Effective population size (x10^4)
20
15
10
5
0
*Equus asinus* (Ti-4)
10^4
10^5
10^6
10^7
Years (g=8, μ=7.242x10^-9)
