# Supplementary material for: Donkey genomes provide new insights into domestication and selection for coat color
Source: Nat Commun. 2020 Dec 8;11:6014. doi: 10.1038/s41467-020-19813-7 (PMC7723042; doi:10.1038/s41467-020-19813-7)
Supplement: Supplementary file 12 — Supplementary Data 9 [file 41467_2020_19813_MOESM12_ESM.pdf]

Supplementary Data 9. Phylogenetic tree based on Y chromosome SNPs from wild asses and domestic donkeys. Sample names in gold color represent North Africa & Eurasia donkeys while sample names in red represent Tropical Africa donkeys. A total of 13,032 SNPs mapping to the Y chromosome were used to construct the tree. FigTree v1.4.4 (http://tree.bio.ed.ac.uk/software/figtree/) was used to display the tree. To make the tree more readable, the branches of the tree were transformed to be cladogram. BEAST2 was applied in this phylogenetic analysis. The parameters for generating the maximum clade credibility (MCC) tree are: HKY for site model, strict clock model for clock model (clock rate = 1), Yule model for tree priors. The phylogenetic tree was generated by Bayesian Markov chain Monte Carlo (MCMC) with 1000 simulations. Statistical support of each node was assessed by MCMC posterior probability indicated by the number aside each node. The following acronyms have been used: Ke (Kenya), Ch (China), Ni (Nigeria), Ir (Iran), Sp (Spain), Eg (Egypt), Et (Ethiopia), Ti (Tibetan), Au (Australia), and Don (the European donkey). Both AW2 and Ona refers to Asian wild asses.

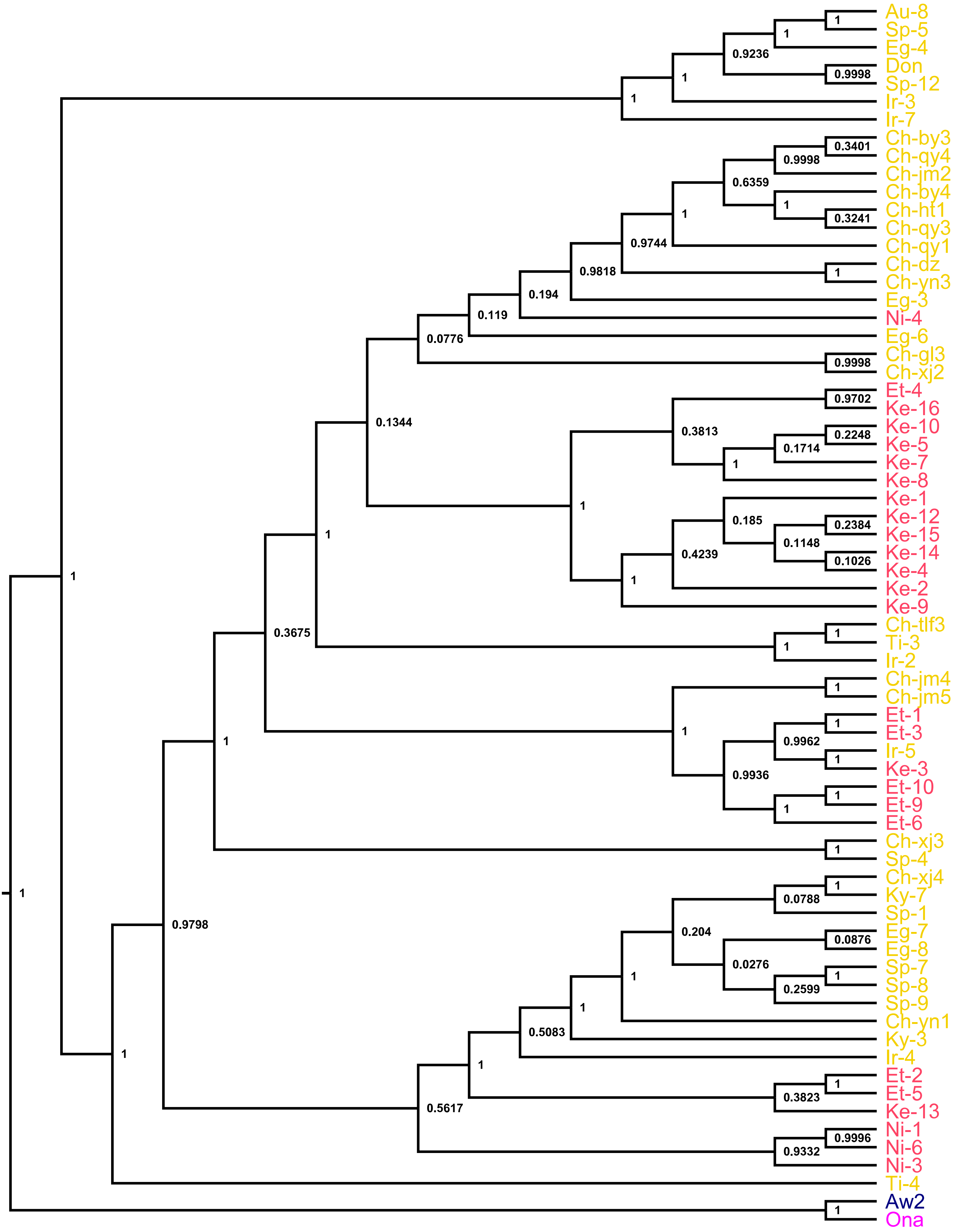
Au-8
Sp-5
Eg-4
Don
Sp-12
Ir-3
Ir-7
Ch-by3
Ch-qy4
Ch-jm2
Ch-by4
Ch-ht1
Ch-qy3
Ch-qy1
Ch-dz
Ch-yn3
Eg-3
Ni-4
Eg-6
Ch-gl3
Ch-xj2
Et-4
Ke-16
Ke-10
Ke-5
Ke-7
Ke-8
Ke-1
Ke-12
Ke-15
Ke-14
Ke-4
Ke-2
Ke-9
Ch-tlf3
Ti-3
Ir-2
Ch-jm4
Ch-jm5
Et-1
Et-3
Ir-5
Ke-3
Et-10
Et-9
Et-6
Ch-xj3
Sp-4
Ch-xj4
Ky-7
Sp-1
Eg-7
Eg-8
Sp-7
Sp-8
Sp-9
Ch-yn1
Ky-3
Ir-4
Et-2
Et-5
Ke-13
Ni-1
Ni-6
Ni-3
Ti-4
Aw2
Ona
1
0.9236
0.9998
0.3401
0.6359
0.3241
0.9744
0.9818
0.194
0.119
0.0776
0.1344
0.9702
0.3813
0.2248
0.1714
0.185
0.2384
0.1148
0.4239
0.1026
0.3675
0.9962
0.9936
0.0788
0.204
0.0876
0.0276
0.2599
0.5083
0.3823
0.5617
0.9996
0.9332
0.9798
