# Supplementary material for: Donkey genomes provide new insights into domestication and selection for coat color
Source: Nat Commun. 2020 Dec 8;11:6014. doi: 10.1038/s41467-020-19813-7 (PMC7723042; doi:10.1038/s41467-020-19813-7)
Supplement: Supplementary file 13 — Supplementary Data 10 [file 41467_2020_19813_MOESM13_ESM.pdf]

Supplementary Data 10. Phylogenetic tree based on SNPs mapping to the mitochondrial genome of Somali wild ass and domestic donkeys. Sample names in gold color represent North Africa & Eurasia donkeys while sample names in red represent Tropical Africa donkeys. A total of 953 mitochondrial SNPs were used to construct the tree. FigTree v1.4.4 (http://tree.bio.ed.ac.uk/software/figtree/) was used to display the tree. To make the tree more readable, the branches of the tree were transformed to be cladogram. BEAST2 software (*90*) was applied in this phylogenetic analysis. The parameters for generating the maximum clade credibility (MCC) tree are: HKY for site model, strict clock model for clock model (clock rate = 1), Yule model for tree priors. The phylogenetic tree was generated by Bayesian Markov chain Monte Carlo (MCMC) with 1000 simulations. Statistical support of each node was assessed by MCMC posterior probability indicated by the number aside each node. The following acronyms have been used: Ke (Kenya), Ch (China), Ni (Nigeria), Ir (Iran), Sp (Spain), Eg (Egypt), Et (Ethiopia), Ti (Tibetan), Au (Australia), and Don (the European donkey).

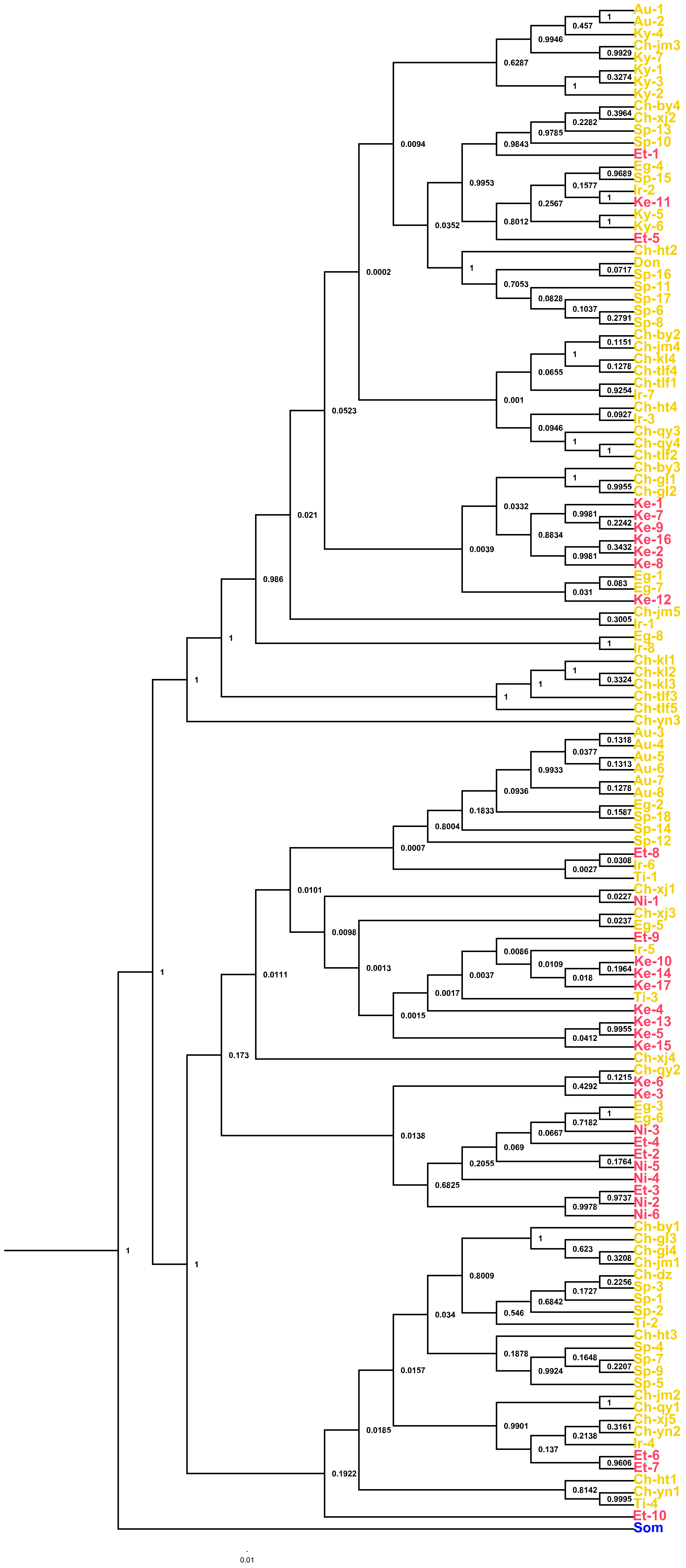
Au-1
Au-2
Ky-4
Ch-jm3
Ky-7
Ky-1
Ky-3
Ky-2
Ch-by4
Ch-xj2
Sp-13
Sp-10
Et-1
Eg-4
Sp-15
Ir-2
Ke-11
Ky-5
Ky-6
Et-5
Ch-ht2
Don
Sp-16
Sp-11
Sp-17
Sp-6
Sp-8
Ch-by2
Ch-jm4
Ch-kl4
Ch-tlf4
Ch-tlf1
Ir-7
Ch-ht4
Ir-3
Ch-qy3
Ch-qy4
Ch-tlf2
Ch-by3
Ch-gl1
Ch-gl2
Ke-1
Ke-7
Ke-9
Ke-16
Ke-2
Ke-8
Eg-1
Eg-7
Ke-12
Ch-jm5
Ir-1
Eg-8
Ir-8
Ch-kl1
Ch-kl2
Ch-kl3
Ch-tlf3
Ch-tlf5
Ch-yn3
Au-3
Au-4
Au-5
Au-6
Au-7
Au-8
Eg-2
Sp-18
Sp-14
Sp-12
Et-8
Ir-6
Ti-1
Ch-xj1
Ni-1
Ch-xj3
Eg-5
Et-9
Ir-5
Ke-10
Ke-14
Ke-17
Ti-3
Ke-4
Ke-13
Ke-5
Ke-15
Ch-xj4
Ch-qy2
Ke-6
Ke-3
Eg-3
Eg-6
Ni-3
Et-4
Et-2
Ni-5
Ni-4
Et-3
Ni-2
Ni-6
Ch-by1
Ch-gl3
Ch-gl4
Ch-jm1
Ch-dz
Sp-3
Sp-1
Sp-2
Ti-2
Ch-ht3
Sp-4
Sp-7
Sp-9
Sp-5
Ch-jm2
Ch-qy1
Ch-xj5
Ch-yn2
Ir-4
Et-6
Et-7
Ch-ht1
Ch-yn1
Ti-4
Et-10
Som
0.01
